# Modulable Photocatalyzed Strategies for the Synthesis of α-*C*-Glycosyl Alanine Analogues via the Giese Reaction with Dehydroalanine Derivates

**DOI:** 10.1021/acs.orglett.3c01660

**Published:** 2023-06-22

**Authors:** Lorenzo Poletti, Alessandro Massi, Daniele Ragno, Federico Droghetti, Mirco Natali, Carmela De Risi, Olga Bortolini, Graziano Di Carmine

**Affiliations:** † Department of Chemical, Pharmaceutical and Agricultural Sciences, University of Ferrara, Via Luigi Borsari, 46, I-44121 Ferrara, Italy; ‡ Department of Environmental and Prevention Sciences, University of Ferrara, Via Luigi Borsari, 46, I-44121 Ferrara, Italy

## Abstract

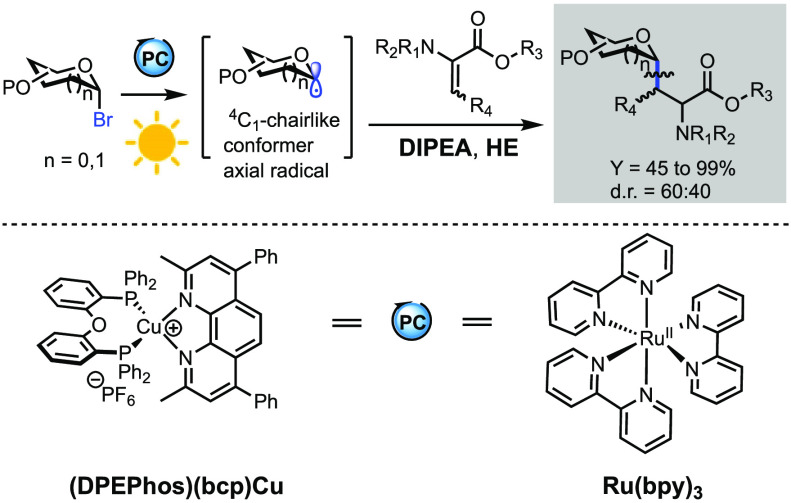

Herein, we present the α-selective Giese reaction between pyranosyl/furanosyl bromides and dehydroalanine analogues, which provides access to a library of highly valuable α-*C*-glycosyl alanines. The key *C*-glycosyl radical is generated through photocatalysis by either the new generation copper(I) complex [(DPEPhos)(bcp)Cu]PF_6_ or [Ru(bpy)_3_](BF_4_)_2_. The reactions proceed smoothly, affording the desired α-*C*-glycosyl alanines in up to 99% yield when diethyl 1,4-dihydro-2,6-dimethyl-3,5-pyridinedicarboxylate [Hantzsch ester (HE)] is used as an additive. *N*,*N*-Diisopropylethylamine (DIPEA) has been selected as a reductant in both protocols. A mechanistic study by means of transient absorption spectroscopy unveils a halogen-atom transfer (XAT) process in *C*-glycosyl radical formation.

The *C*-glycosyl peptides are mimics of native glycoproteins, in which glycans are linked to amino acid moieties by C–C bonds in the anomeric position.^1^ These glycoconjugates can be obtained mainly through two strategies, namely, by installing the glycosyl functionalization on the target peptide or using *C*-glycosyl amino acids during the peptide synthesis.^2^ For example, Wang and Koh reported an elegant nickel-catalyzed C–C coupling reaction between glycosyl halides and the amino or acid functional group, which must be activated by previous chemical modification into pyridinium salts and NHPI ester, respectively.^3^ Goddard-Borger and co-workers reported an interesting protocol to decorate small peptides with a glycosyl moiety by C–C bond formation between glycosyl bromide and modified tryptophan, exploiting photocatalysis.^4^ Even though notably examples have been disclosed in this regard, synthesis of tailored *C*-glycosyl amino acids, which can be further employed in conventional peptide synthesis, represents a more flexible strategy. For example, Kooyk and Codeé recently disclosed a protocol to prepare *C*-mannosyl lysine derivates as building blocks for solid-phase peptide synthesis (SPPS) (Scheme 1A).^5^ To date, post-modification of *C*-glycosyl alkyl carboxylic or carbonylic compounds remains the main strategy in any case, as proven by contributions of Dondoni and Massi, employing proline catalysis (Scheme 1B),^6^ and Gagné, who achieved *C*-glycosyl serine synthesis through Strecker cyanation (Scheme 1C).^7^ In light of that, operationally simple protocols to access these compounds are always welcome. We envisaged the possibility to exploit photocatalysis to promote the Giese reaction between pyranosyl bromide analogues with electron-poor dehydroalanine derivatives to access α-*C*-glycosyl alanine analogues.^8−15^ Photocatalysis that involves a single-electron transfer (SET) mechanism represents a powerful approach for synthesis,^16−18^ as reported in the last 2 decades.^19−26^

**Scheme 1:**
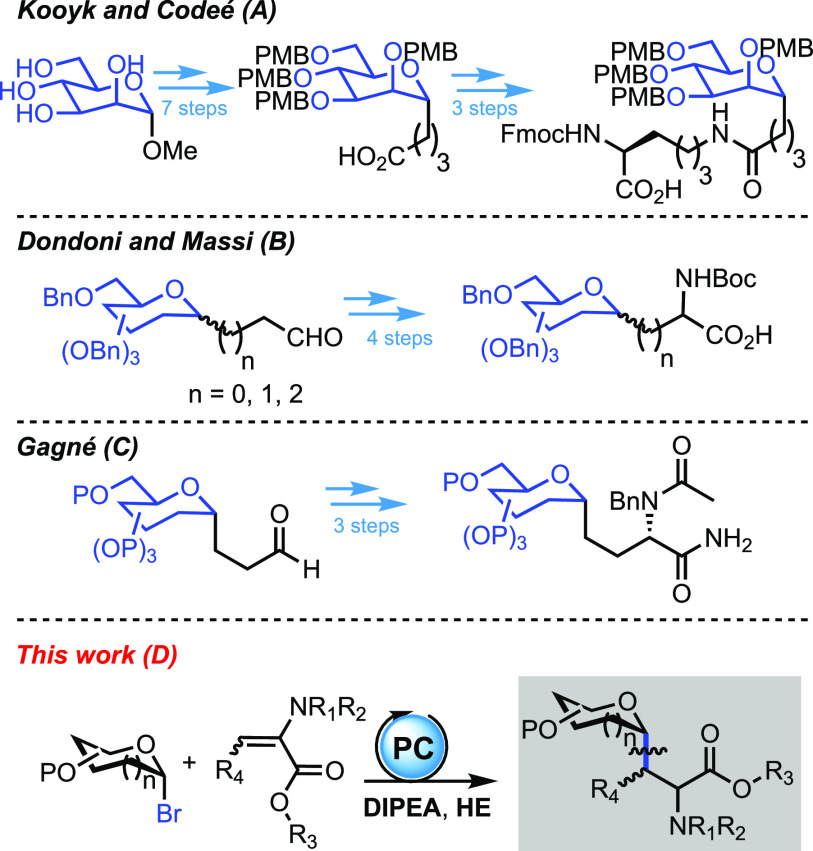
Selected Strategies for the Synthesis of *C*-Glycosyl Amino Acids

A preliminary test has been performed by mixing compound **1a** with compound **2a** in the presence of 5 mol % [Ru(bpy)_3_](BF_4_)_2_**A**, *N*,*N*-diisopropylethylamine (DIPEA), and Hantzsch ester HE(1) under blue light-emitting diode (LED) irradiation (entry 1 in Table 1, *Y* = 50%, dr = 60:40). 2 equiv of compound **2a** were employed to suppress reductive debromination. The diastereomeric ratio (dr) refers to configuration at the C-2 position, whereas the α selectivity of anomeric carbon (C-4) is always maintained thanks to the rigidity of the axial radical by the stereoelectronic effect.^27^ In the absence of HE, the desired product **3aa** is observed, albeit in a low yield (entry 2 in Table 1, *Y* = 25%, dr = 56:44) at the cost of oligomerization side products (see page S53 of the Supporting Information for details on the mechanism).^8^

**Table 1:**
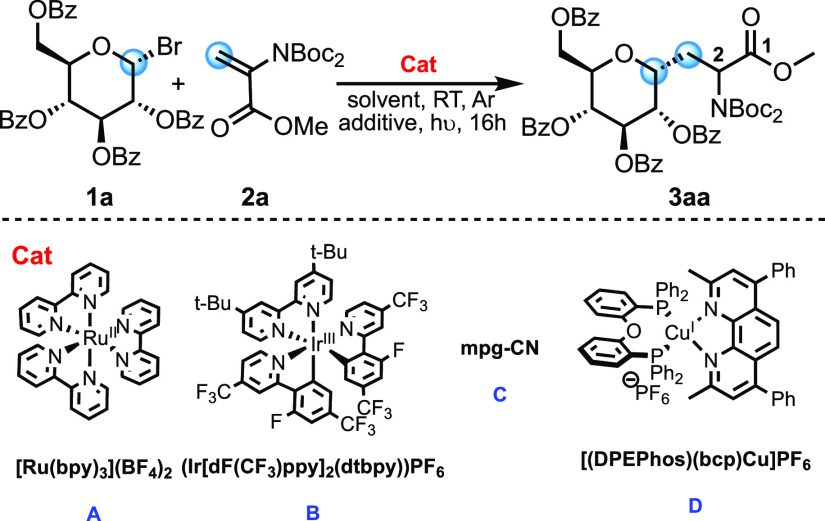
Optimization of the Photocatalysed Giese Reaction between Compound **1a** and **2a**^a^

entry	catalyst	solvent	additive	*Y* (%)^b^	dr^c^
1	**A**	DCM	HE(1)/DIPEA	50	60:40
2	**A**	DCM	DIPEA	25	56:44
3	**A**	DCM	HE(1)	0	
4	**B**	DCM	HE(1)/DIPEA	54	65:35
5	**C**^d^	DCM	HE(1)/TEOA^e^	10	62:38
6	**D**	DCM	HE(1)/DIPEA	86	55:45
7	**D**	H_2_O	HE(1)/DIPEA	20	50:50
8	**D**	EtOH	HE(1)/DIPEA	75	55:45
9	**D**	THF	HE(1)/DIPEA	0	
10	**D**	Tol	HE(1)/DIPEA	0	
11	**D**	EtOAc	HE(1)/DIPEA	trace	nd
12	**D**	MeCN	HE(1)/DIPEA	76	60:40
13^f^	**D**	MeCN/H_2_O^g^	HE(1)/DIPEA	97	60:40
14^f^	**D**	MeCN/H_2_O^g^	HE(2)/DIPEA	50	60:40
15^f^	**D**	MeCN/H_2_O^g^	HE(3)/DIPEA	48	60:40
16^f^	**D**^h^	MeCN/H_2_O^g^	HE(1)/DIPEA	53	60:40

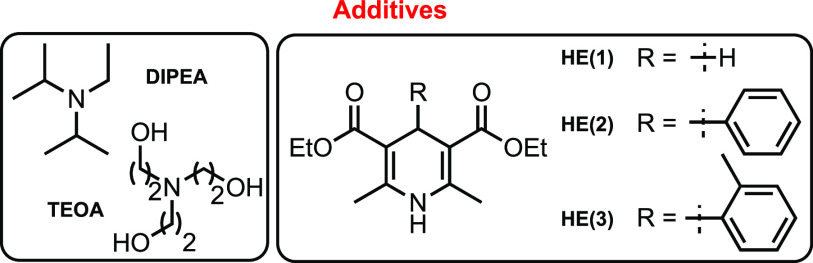

a Reaction conditions: compound **1a** (1 eq., 0.12 mmol), compound **2a** (2 eq., 0.24 mmol), DIPEA (3 equiv, 0.36 mmol), HE (2 equiv, 0.24 mmol), catalyst (5 mol %, 0.006 mmol), solvent (1 mL), and blue LED (10 W).

b Conversion and yield have been calculated by nuclear magnetic resonance (NMR) employing durene as the external standard.

c Calculated by NMR.

d mpg-CN = 10 mg.

e TEOA = 1.2 mmol.

f Reaction time = 2 h.

g MeCN/H_2_O = 2:1.

h Catalyst = 2.5 mol %.

No reactivity was observed without DIPEA with complex **A** (entry 3 in Table 1, *Y* = 0%), and iridium(III) complex **B** does not show significant improvements (entry 4 in Table 1, *Y* = 54%, dr = 65:35). Recently, heterogeneous organic photocatalysts have gained more and more interest thanks to their green features. We tested mesoporous graphitic carbon nitride (mpg-CN) **C** with triethanolamine (TEOA) with unsatisfactory results (entry 5 in Table 1, *Y* = 10%, dr = 62:38).^23^ Finally, compound **3aa** was obtained in good yield with copper(I) complex **D** in dichloromethane (DCM) encouraging us to further investigate different conditions (entry 6 in Table 1, *Y* = 86%, dr = 55:45).^28−31^

An unsatisfactory yield was obtained in water (entry 7 in Table 1, *Y* = 20%, dr = 50:50) and ethanol (entry 8 in Table 1, *Y* = 75%, dr = 55:45). The reaction does not take place in both tetrahydrofuran (THF) (entry 9 in Table 1) and toluene (entry 10 in Table 1), and only a trace amount of product was observed in EtOAc (entry 11 in Table 1). Finally, MeCN/H_2_O (2:1) (entry 13 in Table 1) provides compound **3aa** in 97% yield with dr = 60:40 in only 2 h. Because of the role of HE to quench the odd-electron species as a result of the addition of the glycosyl radical to acceptor **2a** (H transfer from the DHP-4 position), we tested more hindered HEs to increase the diastereoselectivity (see pages S51–S53 of the Supporting Information for details on the mechanism). Unfortunately, no improvement was observed in this regard, as reported in entries 14 and 15 in Table 1; furthermore, we observed the formation of side products as a result of the consecutive addition of compound **2a** (oligomerization). Moreover, lowering the catalytic loading to 2.5% slightly decreases the yield (entry 16 in Table 1, *Y* = 53%, dr = 60:40). With optimized reaction conditions in hand, we moved to explore the generality of the Giese reaction. Table 2 summarizes the results that we obtained by employing the optimized conditions with photocatalyst **D**. With the variation of the bromide derivates, similar results were obtained for mannosyl and galactosyl derivates (**3ba** and **3ca**), in terms of the yield and diastereoselectivity; furthermore, the lactosyl disaccharide derivative (**3da**) proved to be a suitable radical donor in this reaction. By variation of the acceptor, the reactivity remains the same for methyl acrylate (**3ad**), whereas more hindered electrophiles, such as 3-methyl substituted *N*,*N*-Boc2 dehydroalanine **2b** and 3-methyl-substituted *N*-phenyl dehydroalanine **2c**, show lower reactivity (**3ab** and **3ac**); moreover, the tosyl-protected analogue proved to be unreactive under these conditions (**3ae**).

**Table 2:**
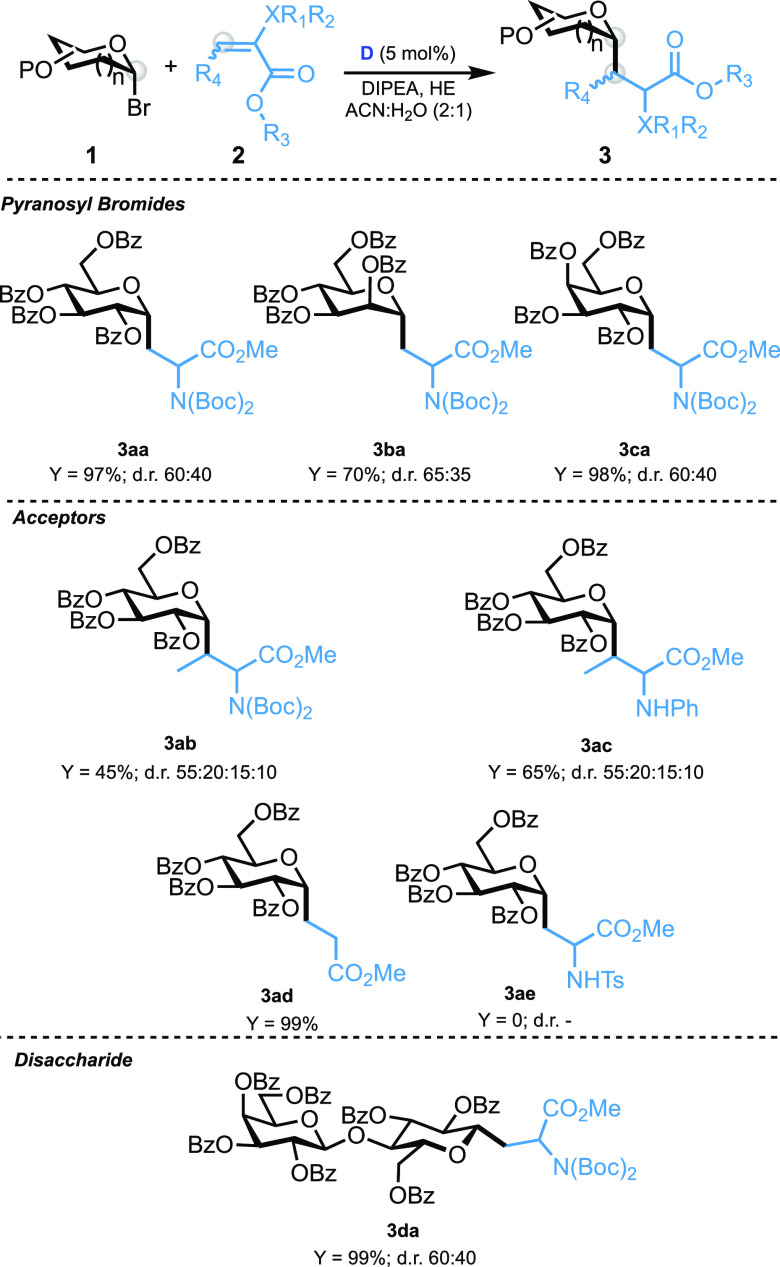
Giese Reaction between Compounds **1a**–**1e** and **2a**–**2e**^a^

a Reaction conditions: compounds **1a**–**1d** (1 equiv, 0.12 mmol), compounds **2a**–**2e** (2 equiv, 0.24 mmol), DIPEA (3 equiv, 0.36 mmol), HE (2 equiv, 0.24 mmol), photocatalyst **D** (5 mol %, 0.006 mmol), ACN/H_2_O (2:1, 1 mL), blue LED (10 W), and time of 2 h. dr was calculated by NMR on the crude, and yield was calculated after the chromatography column.

We tested some of the unreactive acceptors/donors with conditions reported in entry 1 in Table 1 (ruthenium complex **A** as a photocatalyst). The results are summarized in Table 3: reactions performed with peracetylated donor series (glucosyl, mannosyl, and galactosyl) afford desired products in a good yield (**3fa**, **3ga**, and **3ha**). The furanosyl analogue proves to be a suitable reagent by reacting smoothly with compound **2a** (**3ea**). Benchmark donor **1a** reacts smoothly with acetylated dehydroalanine **2f** (**3af**) as well as the cellobiosyl donor analogue **1i** (**3ia**). Scope extension employing catalyst **D** showed that the reaction does not proceed for all acceptors and donors; further attempts proved that substrate poisoning of photocatalyst **D** is the reason. Thus, we investigated photocatalyst **D** substrate-dependent inhibition by NMR experiments. Photocatalyst **D** was dissolved in a mixture of MeCN-*d*_3_/D_2_O (2:1), and the appearance of new signals (5.75 and 5.85 ppm) was observed when 1 equiv of acetylated glucosyl analogue **1f** is added. The magnitude of signals rose by increasing the amount of sugar, and instant precipitation of phosphine ligand (bis[(2-diphenylphosphino)phenyl ether) was observed in the presence of 2 equiv of compound **1f**. The characteristic septet of the PF_6_^–^ anion in the ^31^P NMR spectrum of the filtrate confirmed that copper remains in solution as a new complex, likely involving bathocuproine and sugar as ligands, as possibly inferred from ultraviolet–visible (UV–vis) absorption spectroscopy (see pages S28–S50 of the Supporting Information for details on NMR studies and the UV–vis spectrum).

**Table 3:**
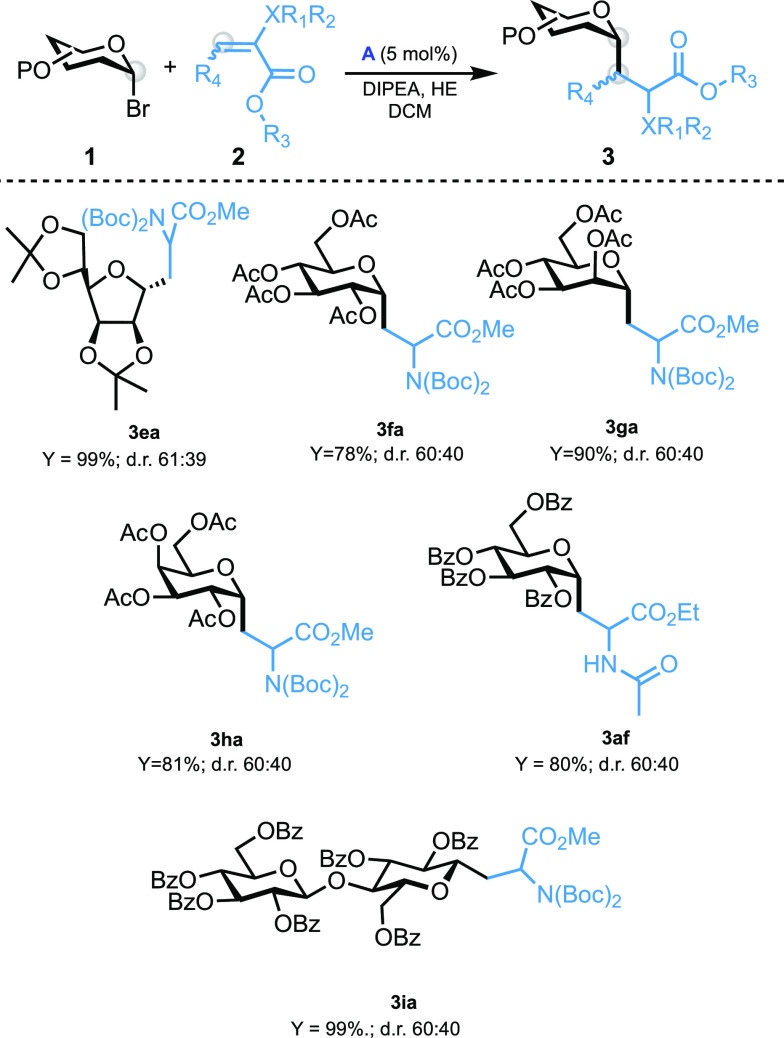
Giese Reaction between Compounds **1f**–**1i** and **2a** and **2f** (Entry 1 in Table 1 Conditions)^a^

a Reaction conditions: compounds **1f**–**1i** (1 equiv, 0.12 mmol), compound **2a** or **2f** (2 equiv, 0.24 mmol), DIPEA (3 equiv, 0.36 mmol), HE (2 equiv, 0.24 mmol), complex **A** (5 mol%, 0.006 mmol), DCM (1 mL), blue LED (10 W), and time of 16 h. dr was calculated by NMR on the crude, and yield was calculated after the chromatography column.

Probably, an intermediate featured by a coordination bond between sugar and metal is involved in the deactivation mechanism. Furthermore, the coordination between the metal center and the carboxylic group should be more favored when the substituent is −C=OCH_3_ (**1f**) than −C=OPh (**1a**) for steric reasons. Furthermore, it was demonstrated that the carbonyl group could insert into the α bond of a metal acyl complex.^32^ Finally, time-resolved absorption experiments were carried out to shine light on the mechanism involved in *C*-glycosyl radical formation. Laser excitation at 355 nm of a DCM solution containing complex **A** and DIPEA (0.37 M) yields a transient spectrum with a maximum at 500 nm, characteristic of the reduced complex **A**^–^ (Figure 1a).^33^ Kinetic analysis of the transient absorption signal at 500 nm shows the presence of two components in the formation of complex **A**^–^. The prompt component (τ < 10 ns) arises from reductive quenching of the triplet excited state of the dye by DIPEA and is consistent with the bimolecular rate constant of 4.0 × 10^7^ M^–1^ s^–1^ estimated by Stern–Volmer analysis (Figure S29 of the Supporting Information). The delayed component (τ = 5.1 μs) is instead ascribable to the reaction of the photogenerated DIPEA^•^ radical with complex **A**. In the absence of any electron acceptor, the amount of photogenerated complex **A**^–^ (proportional to the ΔOD signal at 500 nm according to the Lambert–Beer law) remains appreciably constant during the time window of the experiment as a result of the irreversible nature of the photoreaction involving the DIPEA electron donor.^34^

**Figure 1:**
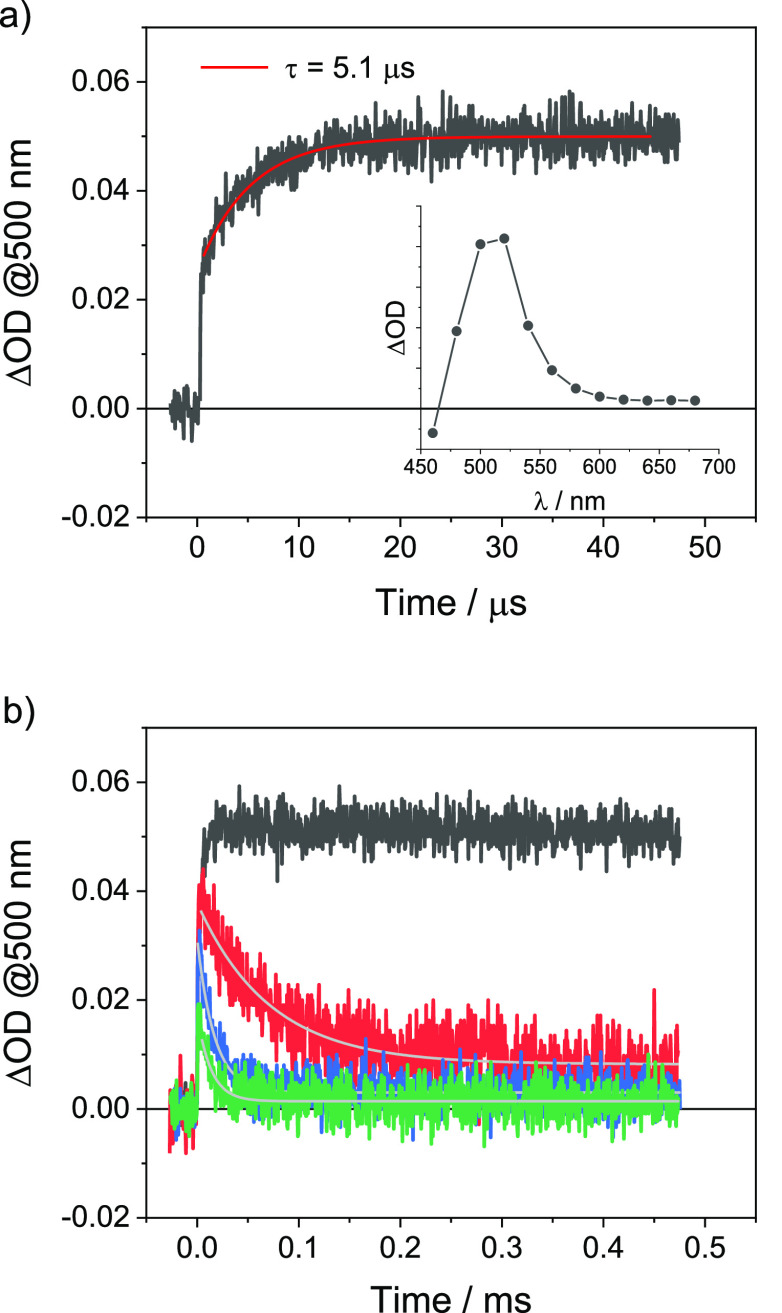
(a) Kinetic trace at 500 nm and transient absorption spectrum at 20 μs (inset) obtained by flash photolysis [excitation at 355 nm and full width at half maximum (fwhm) = 10 ns] of a DCM solution containing complex **A** and 0.37 M DIPEA and (b) kinetic traces at 500 nm under the same conditions in the presence of 0 M (black), 0.013 M (red), 0.036 M (blue), and 0.059 M (green) compound **1a**.

On the other hand, upon the addition of compound **1a**, the transient absorption at 500 nm undergoes a progressive decay with kinetics, which are dependent upon the concentration of sugar (Figure 1b). This can be taken as an indication of the occurrence of a bimolecular electron transfer process from photogenerated complex **A**^–^ to compound **1a**. The decays can be analyzed using pseudo-first-order kinetics, and a bimolecular rate constant of 1.7 × 10^6^ M^–1^ s^–1^ can be calculated (Figure S30 of the Supporting Information). This low value is consistent with a highly activated SET, in agreement with the expected endergonicity of the electron transfer process from complex **A**^–^ to compound **1a**. Interestingly, the maximum amount of photogenerated complex **A**^–^ species (prompt positive signal in Figure 1b; see also Figure S31 of the Supporting Information for further details) is always lower when sugar **1a** is present in the photolyzed solution. This experimental evidence can be explained by considering a fast reaction between the DIPEA^•^ radical and compound **1a** outcompeting the reaction with ground-state complex **A** molecule. According to Leonori and co-workers, α-aminoalkyl radicals from tertiary amines are able to efficiently generate alkyl radicals from alkyl halides through a halogen-atom transfer (XAT) mechanism. Similar results were observed in transient absorption experiments carried out with the iridium(III) complex **B** (see Figures S32–S36 of the Supporting Information for further details). The same conclusions can be transposed to copper(I) complex **D**. Unfortunately, this latter complex gives less efficient excited-state quenching by DIPEA (Figure S37 of the Supporting Information). Thus, we can propose a detailed photoreaction mechanism, which is depicted in Scheme 2.^35,36^

**Scheme 2:**
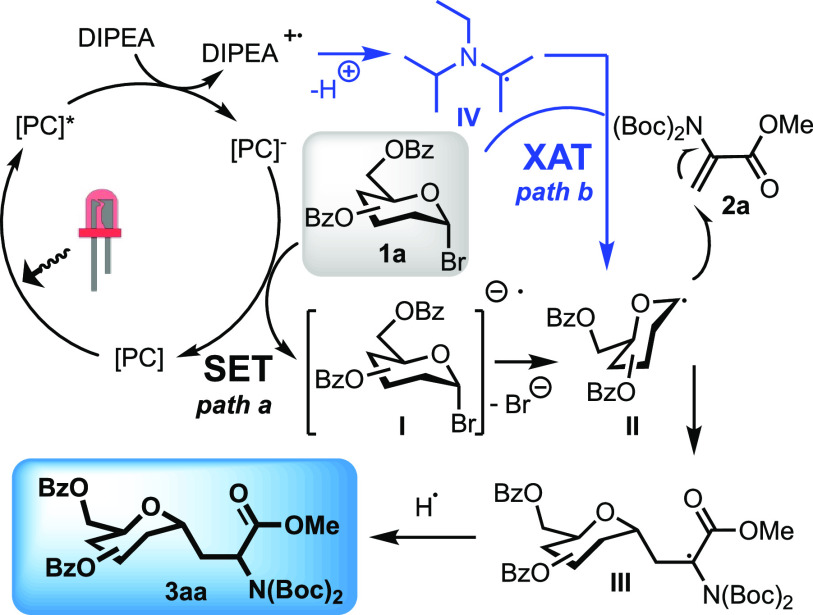
Proposed Mechanism of Photocatalyzed Giese Reaction Including XAT

Photoreaction is initiated with reductive quenching of the photocatalyst excited state (PC*) by DIPEA to form PC^–^, which is then involved in a SET process with compound **1a** affording intermediate **I**. This undergoes a mesolytic cleavage, providing the anomeric radical analogue **II**, which is then sequestrated by compound **2a** to produce intermediate **III** and subsequently compound **3aa** by hydrogen abstraction (path a). According to the photophysical results discussed above, an additional pathway could in parallel occur, in which the α-aminoalkyl radical **IV** abstracts the bromide atom from compound **1a** affording intermediate **II** (path b). Mechanistically, XAT is initiated by the α-amino radical, generated by DIPEA and PC*; thus, it cannot be excluded that the glycosyl radical is directly involved in the quenching of PC^–^. We should consider that, only in the case of photocatalysts **B** and **D**, photoreaction could in principle proceed even in the absence of DIPEA because parallel generation of PC^–^ can also be triggered by reductive quenching of excited PC* by HE (see Figures S33 and S38 of the Supporting Information). The low conversion experimentally observed (see entry 33 in Table S3 of the Supporting Information for details) thus suggests that path a is not very efficient, likely associated with the slow rates of the SET between PC^–^ and compound **1a**. This evidence supports the critical requirement of the DIPEA electron donor to achieve high product yields and the relevance of path b toward the profitable generation of intermediate **II** and the final product.

In conclusion, we have developed a straightforward route to access α-*C*-glycosyl alanine analogues by the photocatalyzed Giese reaction. Two protocols have been disclosed: one involves the copper(I) complex **D** in the MeCN/H_2_O mixture affording the desired product in a very fast reaction time (2 h), with the common ruthenium(II) photocatalyst **A** being employed when this procedure does not work as a result of substrate inhibition. A small library of α-*C*-glycosyl alanine analogues was reported, and photocatalyst substrate-dependent inhibition has been investigated by NMR experiments. Finally, photophysical measurements were performed to elucidate the mechanism involved, unveiling a profitable XAT mechanism occurring in parallel with the established SET process.

## Data Availability

The data underlying this study are available in the published article and its Supporting Information.

## Supporting Information Available

FAIR data, including the primary NMR FID files, for compounds **1**–**13** (ZIP)
Materials and methods, optimization of reaction conditions, characterization data, synthetic procedures, and additional mechanistic experiments (PDF)
