# Supplementary material for: Modulable Photocatalyzed Strategies for the Synthesis of α-C-Glycosyl Alanine Analogues via the Giese Reaction with Dehydroalanine Derivates
Source: Org Lett. 2023 Jun 22;25(26):4862–7. doi: 10.1021/acs.orglett.3c01660 (PMC10334469; doi:10.1021/acs.orglett.3c01660)
Supplement: Supplementary file 2 — ol3c01660_si_002.pdf [file ol3c01660_si_002.pdf]

Modulable photo-catalyzed strategies for the synthesis of α-C-glycosyl alanine analogues *via* Giese reaction with dehydroalanine derivates

Lorenzo Poletti[†], Alessandro Massi[†], Daniele Ragno[†], Federico Droghetti[†], Mirco Natali[†], Carmela De Risi[†], Olga Bortolini[‡] and Graziano Di Carmine[†]*

Corresponding authors
Graziano Di Carmine - †Department of Chemical, Pharmaceutical and Agricultural Sciences, University of Ferrara, Via L. Borsari, 46, 44121 Ferrara (Italy); Email: graziano.dicarmine@unife.it

Authors:

Lorenzo Poletti: [†]Department of Chemical, Pharmaceutical and Agricultural Sciences, University of Ferrara, Via L. Borsari, 46, 44121 Ferrara (Italy)

Alessandro Massi: [†]Department of Chemical, Pharmaceutical and Agricultural Sciences, University of Ferrara, Via L. Borsari, 46, 44121 Ferrara (Italy)

Daniele Ragno: [†]Department of Chemical, Pharmaceutical and Agricultural Sciences, University of Ferrara, Via L. Borsari, 46, 44121 Ferrara (Italy)

Federico Droghetti: [†]Department of Chemical, Pharmaceutical and Agricultural Sciences, University of Ferrara, Via L. Borsari, 46, 44121 Ferrara (Italy)

Mirco Natali: [†]Department of Chemical, Pharmaceutical and Agricultural Sciences, University of Ferrara, Via L. Borsari, 46, 44121 Ferrara (Italy)

Carmela De Risi: [†]Department of Chemical, Pharmaceutical and Agricultural Sciences, University of Ferrara, Via L. Borsari, 46, 44121 Ferrara (Italy)

Olga Bortolini: [‡]Department of Environmental and Prevention Sciences, University of Ferrara, Via L. Borsari, 46, 44121 Ferrara (Italy)

Supporting Information

Summary

Experimental Section 3

TABLES 4

Table S1. Preliminary tests 4

Table S2. Solvent Screening 5

Table S3. Tests with different Hantzsch Ester and with or without additive 6

Table S4. Light and Dark Experiments 7

Test ON/OFF. 8

SYNTHESIS OF PHOTOCATALYSTS 9

SYNTHESIS OF STARTING COMPOUNDS 11

SYNTHESIS OF PRODUCTS 22

NMR INVESTIGATION 28

TEST N°1. 28

TEST N°2. 33

Test N°3: 37

Test N°4: 48

REACTION CARRIED OUT IN DEUTERATED DICHLOROMETHANE 51

SPECTRA OF STARTING COMPOUNDS 54

NMR OF PRODUCTS 82

Mechanistic studies by optical spectroscopy 99

References: 109

Experimental Section

General Experimental Methods. Commercially available reagents were purchased from commercial sources and used without any subsequent purification. The solvents used for starting preparations were distilled from appropriate drying agents and stored over 3 Å molecular sieves. The solvents used for product synthesis were distilled and subsequently deaerated by freeze and pump process. ^{1}H, ^{13}C, and ^{19}F NMR spectra were recorded on Varian Mercury Plus 300 and Varian Mercury Plus 400 spectrometers in $CDCl_3$ at room temperature. ^{13}C{^{1}H} NMR spectra were recorded in ^{1}H broad-band decoupled mode, and chemical shifts (δ) are reported in parts per million relatives to the residual solvent peak. Reactions were monitored by TLC on silica gel 60 F254. Flash column chromatography was performed on silica gel 60 (230−400 mesh). High-resolution mass spectra (HRMS) were recorded in positive ion mode by an Agilent 6520 HPLC-Chip Q/TF-MS nanospray instrument using a time-of-flight, a quadrupole, or a hexapole unit to produce spectra. The blue LEDs (465 nm) used for the synthesis of the products were purchased from Aftertech s.a.s.
UV-Vis spectra were registered on an Agilent Technologies UV-Vis-NIR spectrophotometer. Photoluminescence spectra were taken on an Edinburgh Instrument spectrofluorometer. Time-resolved emission measurements and transient absorption spectroscopy data were taken on a laser flash photolysis apparatus comprised of a Continuum Surelite II Nd:YAG laser (excitation at 355 nm, FWHM = 6–8 ns, provided by THG from the 1064-nm fundamental). Light emitted or transmitted by the sample was focused onto the entrance slit of a 300 mm focal length Acton SpectraPro 2300i triple grating, flat field, and double exit monochromator equipped with a photomultiplier detector (Hamamatsu R3896) or a PIMAX CCD camera. Signals from the photomultiplier were processed by means of a TeledyneLeCroy 604Zi (400 MHz, 20 GS/s) digital oscilloscope. All CH_2Cl_2 solutions were purged using nitrogen gas for 20 minutes before the time-resolved spectroscopic measurements.

TABLES

Table S1. Preliminary tests

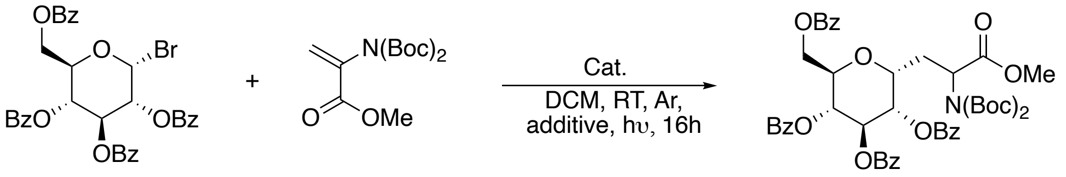

1a **2a** **3aa**

Entry	Cat.	hv	HE	Additive	Conversion	Yield	d.r.
1[a]	K-PHI	10 W	HE	DIPEA	/	/	/
2[a, b]	mpg-CN	10 W	HE	TEOA	15%	<10%	/
3	$[Ru(bpy)_3](BF_4)_2$	10 W	HE	DIPEA	60%	50%	60:40
4	$[Ru(bpy)_3](BF_4)_2$	10 W	/	DIPEA	67%	25%	56:44
5	$[Ru(bpy)_3](BF_4)_2$	10 W	HE	/	/	/	/
6	$[Ru(bpy)_3]Cl_2$	10 W	/	TEOA	44%	12%	54:46
7	$[Ru(bpy)_3](BF_4)_2$	10 W	/	TEOA	10%	≤5%	/
8	$[Ru(bpy)_3]Cl_2$	10 W	HE	TEOA	22%	10%	52:48
9[c]	$[Ru(bpy)_3](BF_4)_2$	10 W	HE	DIPEA	100%	48%	60:40
10[c]	$[Ru(bpy)_3]Cl_2$	10 W	/	TEOA	41%	12%	58:42
11[c, d]	$[Ru(bpy)_3](BF_4)_2$	10 W	HE	DIPEA	45%	42%	60:40
12	$(Ir[dF(CF_3)ppy]_2(dtbpy))PF_6$	20 W	HE	DIPEA	85%	54%	65:35
13	$[(DPEPhos)(bcp)Cu]PF_6$	20 W	HE	DIPEA	75%	60%	50:50
14	$[(DPEPhos)(bcp)Cu]PF_6$	10 W	HE	DIPEA	70%	64%	55:45
15	$[(DPEPhos)(bcp)Cu]PF_6$	10 W	HE	DIPEA	100%	86%	55:45

Reaction conditions: **1a** (1 eq., 0.12 mmol), **2a** (2 eq., 0.24 mmol), Additive (3 eq., 0.36 mmol), HE (2 eq., 0.24 mmol), Cat. (5 mol%, 0.006 mmol), solvent (1 mL), blue LED (10 W). [a]K-PHI (10 mg). [b]mpg-CN (10 mg). [c]hν (10 W, fluorescent bulb). [d]Cat. (2.5 mol%, 0.003 mol).

Table S2. Solvent Screening

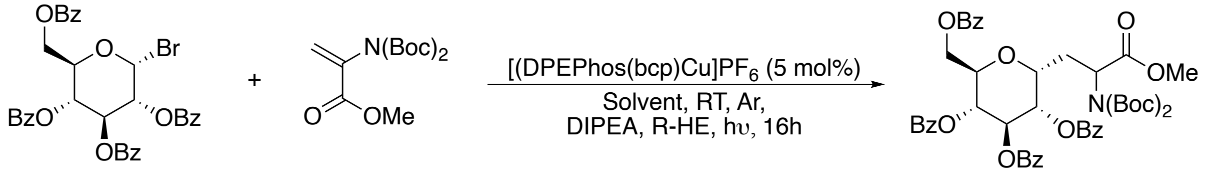

1a **2a** **3aa**

Entry	Solvent	Conversion	Yield	d.r.
16	DCM	100%	86%	55:45
17	H_2O	25%	20%	50:50
18	EtOH	100%	75%	55:45
19	THF	/	/	/
20	DMF	32%	32%	50:50
21	MeCN	100%	76%	60:40
22	Toluene	/	/	/
23	Reline	17%	13%	50:50
24	EtOAc	≤5%	≤5%	n.d.
25[a]	MeCN/H_2O (2:1)	100%	97%	60:40
26	EtOH/H_2O (2:1)	80%	45%	60:40
27	γ-Valerolactone	55%	45%	55:45
28	PEG 600/H_2O (1:1)	30%	24%	50:50
29	Ethyl Lactate	100%	60%	60:40

Reaction conditions: **1a** (1 eq., 0.12 mmol), **2a** (2 eq., 0.24 mmol), Additive (3 eq., 0.36 mmol), HE (2 eq., 0.24 mmol), Cat. (5 mol%, 0.006 mmol), solvent (1 mL), blue LED (10 W). [a]Reaction time 2h.

Table S3. Tests with different Hantzsch Ester and with or without additive

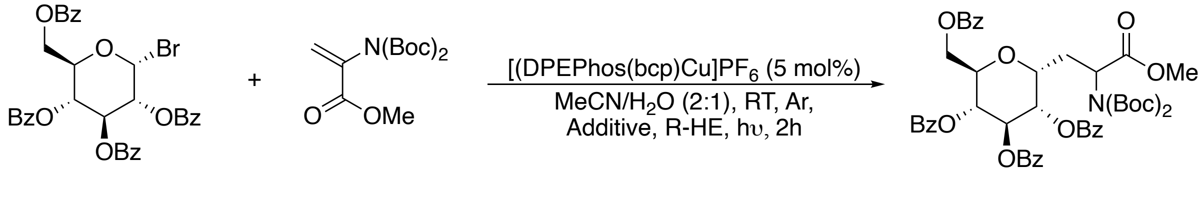

1a **2a** **3aa**

Entry	Cat.	Additive	(R) Additive	Conversion	Yield	d.r.
30	5 mol%	DIPEA	H	100%	97%	60:40
31	10 mol%	DIPEA	H	85%	75%	55:45
32	5 mol%	DIPEA	/	100%	48%	50:50
33	5 mol%	/	H	25%	0%	/
34	5 mol%	DIPEA	Ph	85%	50%	60:40
35	5 mol%	/	Ph	10%	0%	/
36	5 mol %	DIPEA	Ph-ortoMe	100%	48%	60:40

Reaction conditions: **1a** (1 eq., 0.12 mmol), **2a** (2 eq., 0.24 mmol), Additive (3 eq., 0.36 mmol), R-HE (2 eq., 0.24 mmol), Cat. (5 mol%, 0.006 mmol), solvent (1 mL), blue LED (10 W).

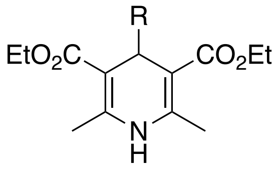

Hantzsch Ester

R = H, Ph, Ph-ortoMe

Ph-ortoMe = 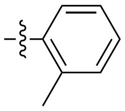

Table S4. Light and Dark Experiments

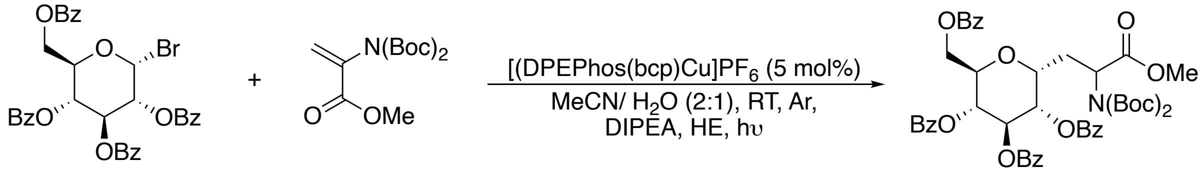

1a **2a** **3aa**

Entry	hv	Cat.	Conversion	Yield	Diast.
36[a]	10 W	/	0%	0%	/
37[b]	/	5 mol%	0%	0%	/

Reaction conditions: [a]**1a** (1 eq., 0.12 mmol), **2a** (2 eq., 0.24 mmol), DIPEA (3 eq., 0.36 mmol), HE (2 eq., 0.24 mmol), MeCN/H_2O (2:1) (1 mL), blue LED (10 W). [b]**1a** (1 eq., 0.12 mmol), **2a** (2 eq., 0.24 mmol), DIPEA (3 eq., 0.36 mmol), HE (2 eq., 0.24 mmol), MeCN/H_2O (2:1) (1 mL), Cat. (5 mol%, 0.006 mmol), blue LED (10 W).

Test ON/OFF.

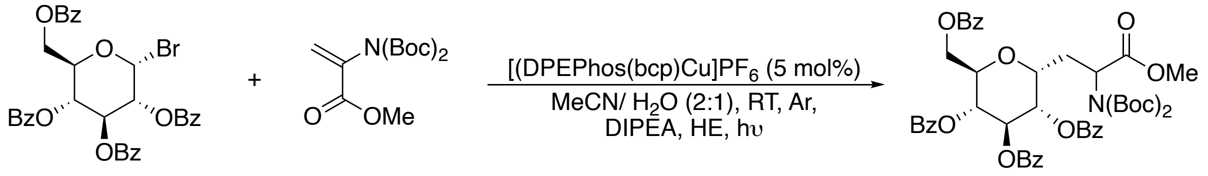

1a **2a** **3aa**

Reaction conditions: **1a** (1 eq., 0.12 mmol), **2a** (2 eq., 0.24 mmol), DIPEA (3 eq., 0.36 mmol), HE (2 eq., 0.24 mmol), Cat. (5 mol%, 0.006 mmol), de-aerated MeCN/H_2O (2:1) (1 mL), blue LED (10 W).

Reaction was left under irradiation 1 hour, after this time the led was switched OFF and reaction kept in dark for 1h. After this time led was switched ON and reaction was maintained under irradiation for 1h. After every hour an NMR sampling was performed in order to measure the yield value.

The results of this experiment are shown in the graphic and table below.

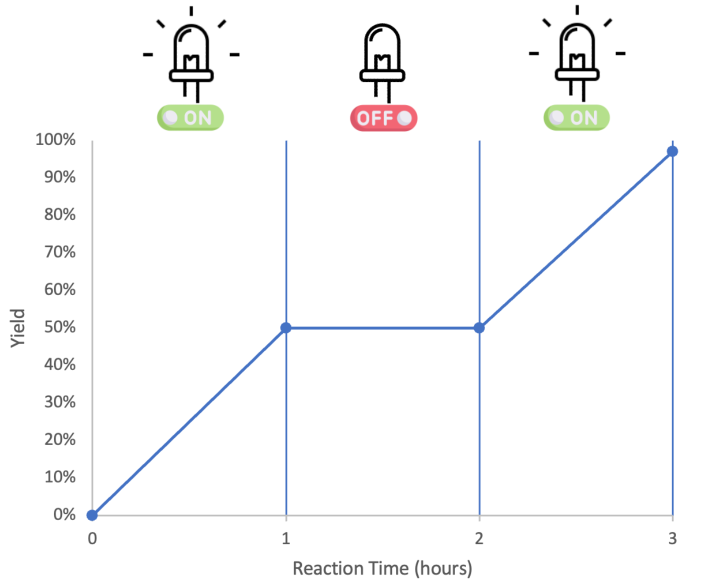

Figure S1: Graphic of Yield vs Raeaction Time for the ON/OFF Test.

Table S5: ON/OFF Test.

Entry	irradiation	Yield	d.r.
38	ON	50%	60:40
39	OFF	50%	60:40
40	ON	97%	60:40

SYNTHESIS OF PHOTOCATALYSTS

Procedure for preparation of [Ru(bpy)$_3$](BF$_4$)$_2$.[1]

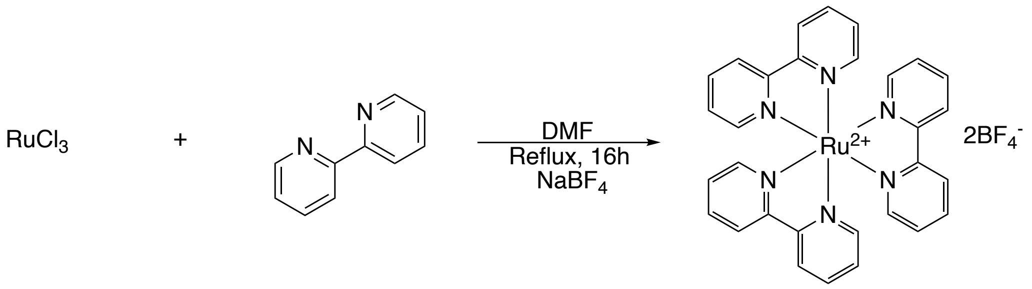

In a bottom round flask a mixture of ruthenium(III) chloride (10 mmol, 2.1g) and 2,2-Bipyridyl (30 mmol, 4.7 g) in DMF was refluxed (oil bath) overnight. Reaction was monitored by TLC (cyclohexane: EtOAc 1:1). Ru(bpy)$_2$Cl$_2$ runs on TLC and doesn't emit under UV light, on the contrary Ru(bpy)$_3$Cl$_2$ doesn't run on TLC and strong emits under UV light.

After 16 hours the solution was diluted with water and extracted with DCM (3x25 mL). The aqueous layer was concentrated at the rotary evaporator and a saturated solution of NaBF$_4$ was added dropwise. The formed precipitate was filtered and washed with cold acetone, collected, and dried under high vacuum.

Procedure for preparation of Ru(bpy)$_3$(PF$_6$)$_2$.[1]

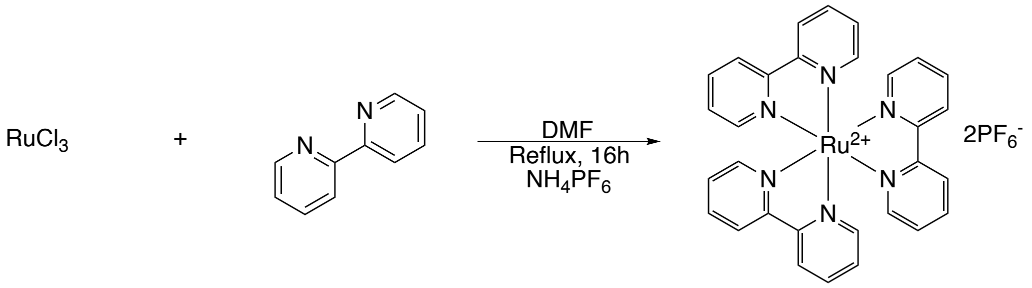

In a bottom round flask a mixture of ruthenium(III) chloride (10 mmol, 2.1g) and 2,2-Bipyridyl (30 mmol, 4.7 g) in DMF was refluxed (oil bath) overnight. Reaction was monitored by TLC (cyclohexane: EtOAc 1:1). Ru(bpy)$_2$Cl$_2$ runs on TLC and doesn't emit under UV light, on the contrary Ru(bpy)$_3$Cl$_2$ doesn't run on TLC and strong emits under UV light.

After 16 hours the solution was diluted with water and extracted with DCM (3x25 mL). The aqueous layer was concentrated at the rotary evaporator and a saturated solution of NH$_4$PF$_6$ was added dropwise. The formed precipitate was filtered and washed with cold acetone, collected and dried under high vacuum.

Procedure for preparation of [DPEPhos(bcp)Cu]PF₆[2]

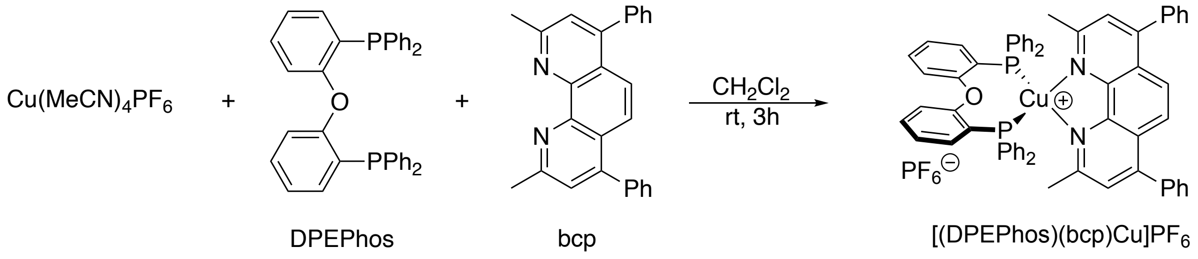

A mixture of tetrakisacetonitrile copper (I) hexafluorophospate (5 mmol, 1.86 g) and bis[(2-diphenylphosphino)phenyl] ether (5 mmol, 2.70 g) was dissolved in dry dichloromethane (400 mL) and stirred two hours at room temperature under argon atmosphere. A solution of bathocuproine (5 mmol, 1.80 g) in dichloromethane (100 mL) was added and the mixture was stirred for an additional hour at room temperature. After this time the mixture was filtered through a pad of Celite, concentrated to half by using rotary evaporator and 500 mL of ether was then added dropwise at the solution strongly stirred. The precipitate was then collected by vacuum to afford [DPEPhos(bcp)Cu]PF_6 (5 g, 4.50 mmol, 90%) as a yellow solid.

Procedure for preparation of [bcp)₂Cu]PF₆[2]

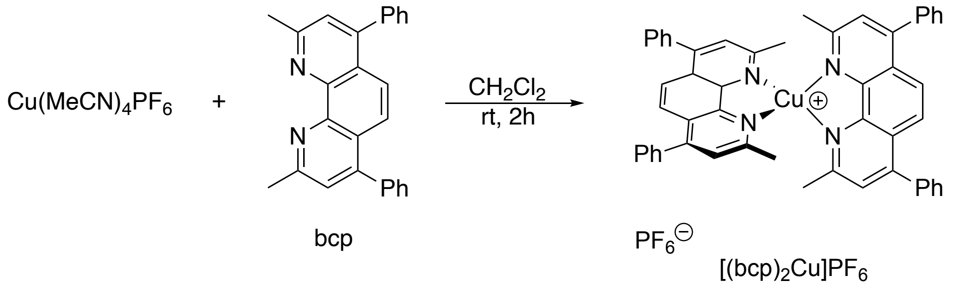

A mixture of tetrakisacetonitrile copper (I) hexafluorophospate (5 mmol, 1.86 g) and bathocuproine (10 mmol, 3.60 g) was dissolved in dry dichloromethane (400 mL) and stirred two hours at room temperature under argon atmosphere. After this time the mixture was filtered through a pad of Celite, concentrated to half volume by using rotary evaporator and 500 mL of ether was then added dropwise at the solution strong stirred. The precipitate was then collected by vacuum to afford [$(bcp)_2$Cu]PF_6 (4.28 g, 4.6 mmol, 92%) as a dark red solid.

SYNTHESIS OF STARTING COMPOUNDS

Procedure for preparation of hexose benzoyl-protected sugars.[3]

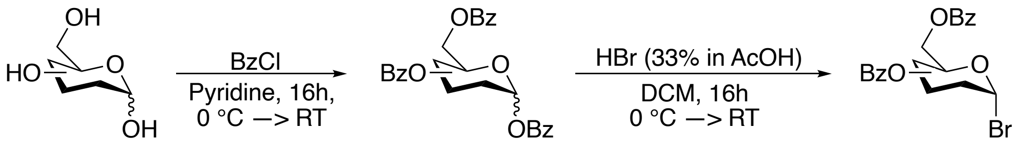

Part A. Sugar (5.0 g, 27.75 mmol, 1 eq.) was dissolved in pyridine (130 mL) and cooled to 0 °C. Benzoyl chloride (21 mL, 181 mmol, 6.5 eq.) was then added dropwise, and the solution was warmed to room temperature and stirred overnight. After this time, ice cold water was added, and the mixture stirred 15 minutes. The mixture was then diluted with dichloromethane (25 mL) and the organic layer was extracted with 1M HCl, water and brine, dried with Na_2SO_4 and the solvent removed by rotary evaporator and by high vacuum pump. Part B. Benzoyl-protected sugar (19.5 g, 27.75 mmol, 1 eq.) was dissolved in dichloromethane (130 mL) and cooled to 0 °C. HBr (33% in Acetic Acid, 40 mL) was added dropwise, and the solution warmed to room temperature and stirred overnight. After this time ice-cold water was added and the reaction was stirred for additional 15 minutes. The aqueous layer was extracted with dichloromethane, dried with Na_2SO_4 and the solvent removed by rotary evaporator and high vacuum pump to give the final compound in a quantitative yield. No further purification steps are needed.

1,2,3,4,6-Penta-O-benzoyl-α-D-glucopyranose (4). By following procedure (Part. A) reported above **4** (19.4 g, 27.75 mmol, quant.) was obtained as a white amorphous solid. ^{1}H NMR (300 MHz, $CDCl_3$) δ 8.17 (d, *J* = 7.1 Hz, 2H), 8.03 (d, *J* = 7.1 Hz, 2H), 7.96 (s, *J* = 7.1 Hz, 2H), 7.88 (s, *J* = 7.1 Hz, 4H), 7.71 – 7.63 (m, 1H), 7.56 – 7.30 (m, 14H), 6.86 (d, *J* = 3.7 Hz, 1H), 6.33 (t, *J* = 10.0 Hz, 1H), 5.87 (t, *J* = 10.0 Hz, 1H), 5.69 (dd, *J* = 10.0, 3.7 Hz, 1H), 4.68 – 4.58 (m, 2H), 4.54 – 4.45 (m, 1H). ^{13}C{^{1}H} NMR (101 MHz, $CDCl_3$) δ 166.1, 166.0, 165.3, 165.1, 164.4, 133.9, 133.5, 133.5, 133.4, 133.1, 130.0, 129.9, 129.8, 129.8, 129.7, 129.5, 129.0, 128.8, 128.7, 128.5, 128.43, 128.41, 90.0, 70.5, 70.5, 70.4, 68.8, 62.4. HRMS (ESI) m/z: $[M + H]^+$ calcd for $C_{41}H_{33}O_{11}$ 701.2017, found701.1984.

2,3,4,6-Tetra-O-benzoyl-α-D-glucopyranosyl bromide (1a). By following procedure (Part. B) reported above, **1a** (18.3 g, 27.75 mmol, quant.) was obtained as a white amorphous solid. ^{1}H NMR (400 MHz, $CDCl_3$) δ 8.07 (d, *J* = 7.1 Hz, 2H), 8.00 (d, *J* = 7.1 Hz, 2H), 7.95 (d, *J* = 7.1 Hz, 2H), 7.87 (d, *J* = 7.1 Hz, 2H), 7.61 – 7.50 (m, 3H), 7.48 – 7.35 (m, 7H), 7.31 (t, *J* = 7.9 Hz, 2H), 6.86 (d, *J* = 4.0 Hz, 1H), 6.26 (t, *J* = 9.8 Hz, 1H), 5.82 (t, *J* = 9.8 Hz, 1H), 5.33 (dd, *J* = 9.8, 4.0 Hz, 1H), 4.77 – 4.70 (m, 1H), 4.67 (dd, *J* = 12.5, 2.7 Hz, 1H), 4.51 (dd, *J* = 12.5, 4.0 Hz, 1H). ^{13}C{^{1}H} NMR (101 MHz, $CDCl_3$) δ 166.0, 165.6, 165.3, 165.1, 133.8, 133.6, 133.3, 133.2, 130.1, 129.9, 129.8, 129.7, 129.4, 128.8, 128.54, 128.48, 128.44, 128.36, 86.9, 72.7, 71.5, 70.6, 68.0, 61.9. HRMS (ESI) m/z: $[M + H]^+$ calcd for $C_{34}H_{28}BrO_9$ 659.0911, found 659.0879.

1,2,3,4,6-Penta-O-benzoyl-α/β-D-mannopyranose (5). By following procedure (Part. A) reported above **5** (19.4 g, 27.75 mmol, quant.) was obtained as a viscous transparent oil in a mixture of two anomers in ratio 1:0.2. ^{1}H NMR (400 MHz, $CDCl_3$) δ 8.21 (d, J = 7.1 Hz, 2H), 8.18 – 8.15 (m, 0.8H), 8.09 (d, J = 7.8 Hz, 4.8H), 7.96 (d, J = 7.2 Hz, 2H), 7.92 (d, J = 8.0 Hz, 0.4H), 7.86 (d, J = 7.2 Hz, 2H), 7.68 – 7.30 (m, 18H), 6.63 (d, J = 2.1 Hz, 1H), 6.43 (d, J = 1.2 Hz, 0.2H), 6.28 (t, J = 10.2 Hz, 1H), 6.17 (t, J = 9.8 Hz, 0.2H), 6.11 (dd, J = 3.1, 1.0 Hz, 0.2H), 6.07 (dd, J = 10.2, 3.3 Hz, 1H), 5.93 – 5.90 (m, 1H), 5.80 (dd, J = 9.9, 3.2 Hz, 0.2H), 4.76 (dd, J = 12.2, 2.7 Hz, 0.2H), 4.70 (dd, J = 12.3, 2.5 Hz, 1H), 4.60 – 4.54 (m, 1H), 4.50 (dd, J = 12.2, 3.7 Hz, 1H), 4.40 – 4.34 (m, 0.2H). 13C{^{1}H} NMR (101 MHz, $CDCl_3$) δ 166.0, 165.7, 165.3, 165.1, 163.8, 134.5, 134.1, 133.7, 133.5, 133.4, 133.0, 130.6, 130.1, 130.0, 129.8, 129.8, 129.0, 128.86, 128.81, 128.7, 128.46, 128.41, 128.39, 91.4, 71.2, 70.0, 69.4, 66.2, 62.3. HRMS (ESI) m/z: $[M + H]^+$ calcd for $C_{41}H_{33}O_{11}$ 701.2017, found 701.2038.

2,3,4,6-Tetra-O-benzoyl-α-mannopyranosyl bromide (1b). By following procedure (Part. B) reported above, **1c** (18.3 g, 27.75 mmol, quant.) was obtained as a white amorphous solid. ^{1}H NMR (400 MHz, $CDCl_3$) δ 8.10 (dd, *J* = 8.4, 1.3 Hz, 2H), 8.03 (dd, *J* = 8.4, 1.3 Hz, 2H), 7.98 (dd, *J* = 8.4, 1.3 Hz, 2H), 7.84 (dd, *J* = 8.4, 1.3 Hz, 2H), 7.64 – 7.50 (m, 3H), 7.48 – 7.36 (m, 7H), 7.29 (d, *J* = 8.0 Hz, 1H), 6.59 (d, *J* = 1.1 Hz, 1H), 6.35 – 6.18 (m, 2H), 5.91 (dd, *J* = 2.9, 1.7 Hz, 1H), 4.74 (dd, *J* = 12.5, 2.4 Hz, 1H), 4.71 – 4.62 (m, 1H), 4.51 (dd, *J* = 12.5, 3.8 Hz, 1H). $^{13}C\{^1H\}$ NMR (101 MHz, $CDCl_3$) δ 165.9, 165.34, 165.27, 164.9, 133.7, 133.6, 133.4, 133.2, 129.86, 129.78, 129.76, 128.7, 128.52, 128.48, 128.37, 83.2, 73.1, 72.9, 69.0, 65.9, 61.7. HRMS (ESI) m/z: $[M + H]^+$ calcd for $C_{34}H_{28}BrO_9$ 659.0911, found 659.0941.

1,2,3,4,6-Penta-O-benzoyl-α-D-galactopyranose (6) By following procedure (Part. A) reported above **6** (19.4 g, 27.75 mmol, quant.) was obtained as a white amorphous solid. ^{1}H NMR (400 MHz, $CDCl_3$) δ 8.11 (dd, *J* = 7.1, 4.8 Hz, 4H), 7.96 (d, *J* = 7.1 Hz, 2H), 7.86 (d, *J* = 7.1 Hz, 2H), 7.82 (d, *J* = 7.1 Hz, 2H), 7.64 (t, *J* = 7.1 Hz, 2H), 7.57 – 7.49 (m, 5H), 7.46 (t, *J* = 7.5 Hz, 2H), 7.39 (t, *J* = 7.5 Hz, 2H), 7.33 – 7.26 (m, 4H), 6.95 (d, *J* = 3.5 Hz, 1H), 6.19 (d, *J* = 3.5 Hz, 1H), 6.12 (dd, *J* = 10.7, 3.5 Hz, 1H), 6.03 (dd, *J* = 10.7, 3.5 Hz, 1H), 4.83 (t, *J* = 6.6 Hz, 1H), 4.63 (dd, *J* = 11.3, 6.6 Hz, 1H), 4.42 (dd, *J* = 11.3, 6.6 Hz, 1H). $^{13}C\{^1H\}$ NMR (101 MHz, $CDCl_3$) δ 165.9, 165.7, 165.5, 165.4, 164.5, 133.9, 133.7, 133.46, 133.38, 133.2, 129.9, 129.7, 129.3, 129.0, 128.9, 128.8, 128.7, 128.4, 128.3, 90.6, 69.4, 68.5, 68.4, 67.7, 61.8, 26.9. HRMS (ESI) m/z: $[M + H]^+$ calcd for $C_{41}H_{33}O_{11}$ 701.2017, found 701.1994.

2,3,4,6-Tetra-O-benzoyl-α-galactopyranosyl bromide (1c). By following procedure (Part. B) reported above, **1c** (18.3 g, 27.75 mmol, quant.) was obtained as a white amorphous solid. ^{1}H NMR (300 MHz, $CDCl_3$) δ 8.07 (d, *J* = 7.1 Hz, 2H), 8.03 – 7.97 (m, 4H), 7.80 (d, *J* = 7.1 Hz, 2H), 7.64 (t, *J* = 7.1 Hz, 1H), 7.59 – 7.36 (m, 10H), 6.97 (d, *J* = 3.9 Hz, 1H), 6.12 (d, *J* = 2.5 Hz, 1H), 6.05 (dd, *J* = 10.4, 3.9 Hz, 1H), 5.67 (dd, *J* = 10.4, 3.9 Hz, 1H), 4.92 (t, *J* = 6.4 Hz, 1H), 4.64 (dd, *J* = 11.5, 6.4 Hz, 1H), 4.46 (dd, *J* = 11.5, 6.4 Hz, 1H). $^{13}C\{^1H\}$ NMR (101 MHz, $CDCl_3$) δ 165.9, 165.5, 165.33, 165.29, 133.8, 133.4, 133.3, 130.0, 129.9, 129.8, 129.7, 128.8, 128.7, 128.5, 128.4, 128.3, 88.2, 71.8, 68.8, 68.6, 68.0, 61.6. HRMS (ESI) m/z: $[M + H]^+$ calcd for $C_{34}H_{28}BrO_9$ 659.0911, found 659.0931.

Procedure for preparation of hexose acetyl-protected sugars.[3]

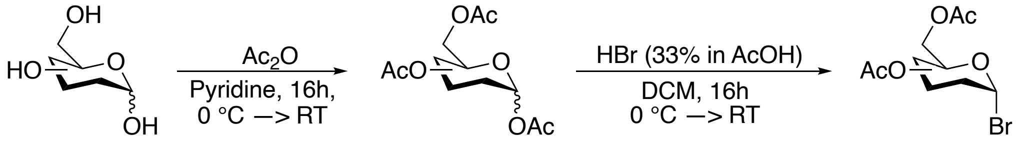

Part A. Sugar (5.0 g, 27.75 mmol, 1 eq.) was dissolved in pyridine (130 mL) and cooled to 0 °C. Acetic anhydride (18.3 mL, 194 mmol, 7 eq.) was then added dropwise, and the solution was warmed to room temperature and stirred overnight. After this time, ice cold water was added, and the mixture stirred 15 minutes. The mixture was then diluted with dichloromethane (25 mL) and the organic layer was extracted with 1M HCl, satd $NaHCO_3$ and water and dried with Na_2SO_4. The solvent was removed by rotary evaporator and high vacuum pump to afford the desired compound (11.4 g, 27.75 mmol) who was used in the next step without further purification.

Part B. Acetyl-protected sugar (11.4 g, 27.75 mmol, 1 eq.) was dissolved in dichloromethane (130 mL) and cooled to 0 °C. HBr (33% in Acetic Acid, 40 mL) was added dropwise, and the solution warmed to room temperature and stirred overnight. After this time ice-cold water was added and the reaction was stirred for additional 15 minutes. The aqueous layer was extracted with dichloromethane, dried with Na_2SO_4 and the solvent removed by rotary evaporator and high vacuum pump to give the final compound in a quantitative yield. No further purification steps are needed.

2,3,4,6-Tetra-O-acetyl-α-glucopyranosyl bromide (1f). By following procedure (Part. B) reported above, **1f** (11.4 g, 27.75 mmol, quant.) was obtained as a colorless viscous oil. ^{1}H NMR (400 MHz, $CDCl_3$) δ 6.61 (d, *J* = 4.0 Hz, 1H), 5.55 (t, *J* = 9.7 Hz, 1H), 5.16 (t, *J* = 9.7 Hz, 1H), 4.83 (dd, *J* = 9.7, 4.0 Hz, 1H), 4.35 – 4.26 (m, 2H), 4.13 (d, *J* = 10.7 Hz, 1H), 2.10 (s, 3H), 2.09 (s, 3H), 2.05 (s, 3H), 2.03 (s, 3H). $^{13}C\{^1H\}$ NMR (101 MHz, $CDCl_3$) δ 170.5, 169.85, 169.79, 169.5, 86.5, 72.1, 70.6, 70.1, 67.1, 60.9, 20.62, 20.59, 20.52. HRMS (ESI) m/z: $[M + H]^+$ calcd for $C_{14}H_{20}BrO_9$ 412.0285, found 412.0267.

1,2,3,4,6-Penta-O-acetyl-α/β-mannopyranose (7). By following procedure (Part. A) reported above **7** (10.8 g, 27.75 mmol, quant.) was obtained as a colorless viscous oil in a mixture of two anomers in ratio 1:0.3. ^{1}H NMR (400 MHz, $CDCl_3$) δ 6.08 (d, *J* = 1.7 Hz, 1H), 5.85 (s, 0.3H), 5.48 (d, *J* = 3.0 Hz, 0.3H), 5.38 – 5.32 (m, 2H), 5.32 – 5.28 (m, 0.3H), 5.25 (d, *J* = 2.2 Hz, 1H), 5.12 (dd, *J* = 10.0, 3.3 Hz, 0.3H), 4.33 – 4.24 (m, 1.3H), 4.15 – 4.02 (m, 2.3H), 3.80 (ddd, *J* = 9.8, 5.3, 2.3 Hz, 0.3H), 2.21 (s, 0.9H), 2.17 (s, 3H), 2.16 (s, 3H), 2.094 (s, 0.9H), 2.085 (s, 3.9H), 2.04 (s, 3.9H), 2.00 (s, 3.9H). ^{13}C NMR (101 MHz, $CDCl_3$) δ 170.6, 170.2, 170.0, 169.8, 169.7, 169.55, 169.51, 168.0, 90.6, 90.4, 73.3, 70.6, 70.6, 68.7, 68.3, 68.2, 65.5, 65.4, 62.1, 62.0, 20.8, 20.75, 20.70, 20.6, 20.5.HRMS (ESI) m/z: $[M + H]^+$ calcd for $C_{16}H_{23}O_{11}$ 391.1235, found 391.1216.

2,3,4,6-Tetra-O-acetyl-α-mannopyranosyl bromide (1g). By following procedure (Part. B) reported above, **1g** (11.4 g, 27.75 mmol, quant.) was obtained as a colorless viscous oil. ^{1}H NMR (400 MHz, $CDCl_3$) δ 6.29 (s, 1H), 5.72 (dd, *J* = 10.2, 3.4 Hz, 1H), 5.45 (dd, *J* = 3.3, 1.5 Hz, 1H), 5.37 (t, *J* = 10.2 Hz, 1H), 4.33 (dd, *J* = 12.5, 4.9 Hz, 1H), 4.22 (ddd, *J* = 10.2, 4.9, 2.1 Hz, 1H), 4.14 (dd, *J* = 12.4, 2.1 Hz, 1H), 2.17 (s, 3H), 2.10 (s, 3H), 2.07 (s, 3H), 2.01 (s, 3H). $^{13}C\{^1H\}$ NMR (101 MHz, $CDCl_3$) δ 170.5, 169.6, 169.5, 83.0, 72.8, 72.1, 67.9, 65.3, 61.4, 20.74, 20.66, 20.63, 20.56. HRMS (ESI) m/z: $[M + H]^+$ calcd for $C_{14}H_{20}BrO_9$ 412.0285, found 412.0302.

2,3,4,6-Tetra-O-acetyl-α-galactopyranosyl bromide (1h). By following procedure (Part. B) reported above, **1h** (11.4 g, 27.75 mmol, quant.) was obtained as a colorless viscous oil. ^{1}H NMR (400 MHz, $CDCl_3$) δ 6.69 (d, *J* = 3.9 Hz, 1H), 5.52 (dd, *J* = 3.3, 1.1 Hz, 1H), 5.40 (dd, *J* = 10.6, 3.3 Hz, 1H), 5.05 (dd, *J* = 10.6, 4.0 Hz, 1H), 4.48 (t, *J* = 6.6 Hz, 1H), 4.15 (ddd, *J* = 31.3, 11.4, 6.6 Hz, 2H), 2.15 (s, 3H), 2.11 (s, 3H), 2.06 (s, 3H), 2.01 (s, 3H). $^{13}C\{^1H\}$ NMR (101 MHz, $CDCl_3$) δ 170.3, 170.0, 169.9, 169.7, 88.1, 71.0, 68.0, 67.7, 66.9, 60.8, 20.7, 20.62, 20.56, 20.53. HRMS (ESI) m/z: $[M + H]^+$ calcd for $C_{14}H_{20}BrO_9$ 412.0285, found 412.0277.

Procedure for preparation of 2,3:5,6-Di-*O*-isopropylidene-α-D-mannofuranosyl Bromide (1e)[4,5]

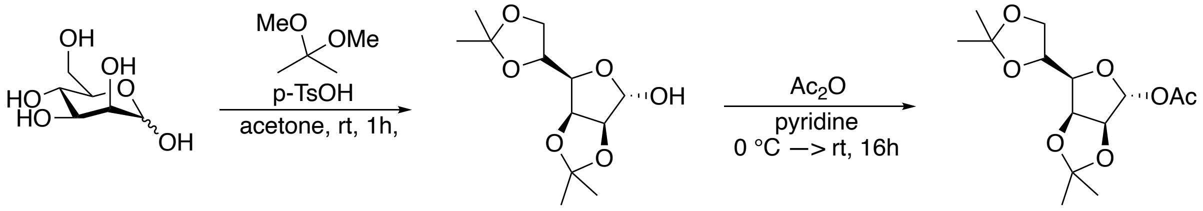

8 **9**

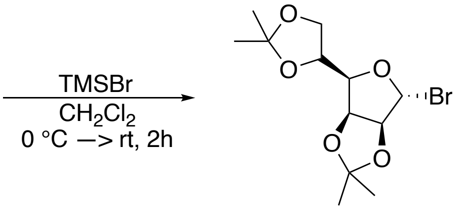

1e

Part A. A suspension of D-mannose (1.69 g, 9.375 mmol) in 2,3-dimethoxypropane (5 mL, 27.71 mmol) and acetone (5 mL) with a catalytic amount of p-TsOH (178 mg, 0.937 mmol) was stirred at room temperature for 1 hour. The resulting solution was treated with saturated solution of $NaHCO_3$ and concentrated to half volume using rotary evaporator. The solution was extracted with EtOAc (15 mL) and the organic layer was washed with brine, dried with anhydrous Na_2SO_4, filtered, and the solvent removed by rotary evaporator and high vacuum pump affording **8** that was used in next step without further purification.

Part B. 2,3:5,6-Di-O-isopropylidene-α-D-mannofuranose (**8**) (2.44 g, 9.375 mmol) was dissolved in pyridine (5 mL) and acetic anhydride (1.77 mL, 18.75 mmol) was added dropwise at 0 °C. The resulting solution was stirred at room temperature overnight. After this time the mixture was cooled to 0 °C and EtOH (5 mL) was added. Using rotary evaporator, the solvent was removed, and the resulting viscous oil was taken up in EtOAc (15 mL) and washed with 1M HCl and saturated $NaHCO_3$ solution. The organic phase was dried with anhydrous Na_2SO_4, filtered, and the solvent removed by rotary evaporator and high vacuum pump affording **9** that was used in next step without further purification.

Part C. 1-*O*-Acetyl-2,3:5,6-Di-O-isopropylidene-α-D-mannofuranose (**9**) (2.8 g, 9.375 mmol) was dissolved in dichloromethane and TMSBr (1.85 mL, 14 mmol) was added dropwise at 0 °C. Reaction was then allowed to achieve room temperature while being stirred. The progress of the reaction was monitored by TLC (cyclohexane:EtOAc 1:1) by phosphomolybdate coloration. After the reaction was complete, the solvent and any excess of TMSBr was remove by rotary evaporator and high vacuum pump to give the final compound **1e** in a quantitative yield. No further purification steps are needed.

2,3:5,6-Di-O-isopropylidene-α/β-D-mannofuranose (8). By following procedure (Part. A) reported above **8** (2.44 g, 9.375 mmol, quant.) was obtained as an amorphous white solid in a mixture of two anomers in ratio 1:0.3. ^1H NMR (400 MHz, $CDCl_3$) δ 5.37 (s, 1H), 5.00 (s, 0.3H) 4.81 (dd, *J* = 5.6, 4.1 Hz, 1H), 4.76 (dd, *J* = 6.0, 3.3 Hz, 0.3H), 4.62 (d, *J* = 5.9 Hz, 1H), 4.53 (dd, *J* = 6.0, 3.6 Hz, 0.3H), 4.43 – 4.36 (m, 1.3H), 4.18 (dd, *J* = 7.2, 3.6 Hz, 1H), 4.11 – 4.02 (m, 2.6H), 3.47 (dd, *J* = 8.2, 3.2 Hz, 0.3H), 1.53 (s, 0.9H), 1.46 (s, 3H), 1.45 (s, 3H), 1.43 (s, 0.9H), 1.39 (s, 0.9H), 1.37 (s, 3.9H), 1.32 (s, 3H). $^{13}C\{^1H\}$ NMR (101 MHz, $CDCl_3$) δ 113.4, 112.6, 109.3, 109.1, 101.2, 97.1, 85.4, 80.1, 79.6, 79.3, 78.5, 76.1, 73.2, 72.9, 67.1, 66.5, 27.0, 26.8, 25.8, 25.7, 25.3, 25.1, 24.8, 24.4. HRMS (ESI) m/z: $[M + H]^+$ calcd for $C_{12}H_{21}O_6$ 261.1333, found 261.1339.

1-*O*-Acetyl-2,3:5,6-Di-O-isopropylidene-α-D-mannofuranose (9). By following procedure (Part. B) reported above **9** (2.83 g, 9.375 mmol, quant.) was obtained as a yellow oil. ^{1}H NMR (300 MHz, $CDCl_3$) δ 6.12 (s, 1H), 4.85 (dd, *J* = 5.9, 3.6 Hz, 1H), 4.70 (d, *J* = 5.9 Hz, 1H), 4.40 (ddd, *J* = 8.0, 6.1, 4.2 Hz, 1H), 4.11 – 4.02 (m, 3H), 2.07 (s, 3H), 1.48 (s, 3H), 1.46 (s, 3H), 1.37 (s, 3H), 1.34 (s, 3H). ^{13}C{^{1}H} NMR (101 MHz, $CDCl_3$) δ 169.4, 113.3, 109.3, 100.8, 85.1, 82.2, 79.3, 72.9, 66.8, 27.0, 25.9, 25.1, 24.7, 21.1. HRMS (ESI) m/z: [M + H]$^+$ calcd for $C_{14}H_{23}O_7$ 303.1438, found 303.1427.

2,3:5,6-Di-O-isopropylidene-α-D-mannofuranosyl bromide (1e). By following procedure (Part. B) reported above **1e** (2.83 g, 9.375 mmol, quant.) was obtained as an amorphous pale red solid. ^{1}H NMR (300 MHz, $CDCl_3$) δ 6.38 (d, *J* = 1.0 Hz, 1H), 5.16 (d, *J* = 5.8 Hz, 1H), 4.89 (dd, *J* = 5.8, 3.6 Hz, 1H), 4.47 (ddd, *J* = 7.8, 5.8, 3.6 Hz, 1H), 4.19 (dd, *J* = 7.5, 3.6 Hz, 1H), 4.10 (dd, *J* = 8.8, 5.8 Hz, 1H), 4.00 (dd, *J* = 8.8, 4.3 Hz, 1H), 1.48 (s, 3H), 1.46 (s, 3H), 1.39 (s, 3H), 1.33 (s, 3H). ^{13}C{^{1}H} NMR (101 MHz, $CDCl_3$) δ 113.3, 109.6, 92.8, 90.0, 83.2, 78.3, 72.0, 66.7, 30.9, 25.8, 25.1, 24.6. HRMS (ESI) m/z: [M + H]$^+$ calcd for $C_{12}H_{20}BrO_5$ 323.0489, found 323.0474.

Procedure for preparation of 2,3,4,6-Tetra-O-benzoyl-β-D-galactopyranosyl-(1→4)-2,3,6-tri-O-benzoyl-α-D- glucopyranosyl bromide (1d).[3]

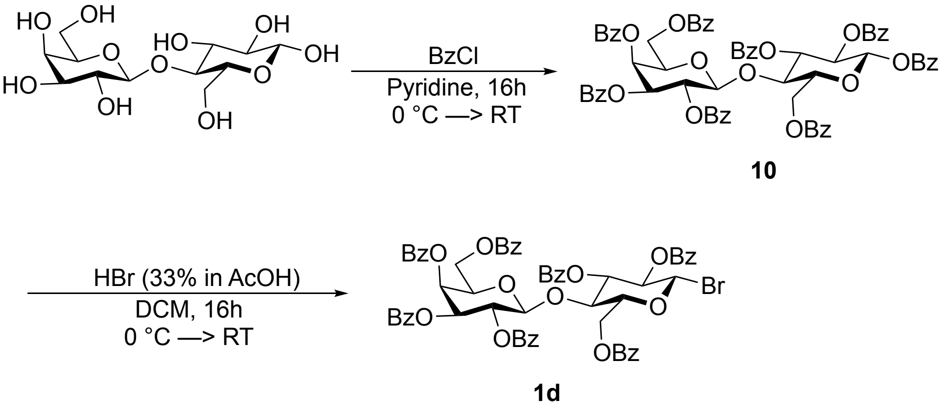

<u>Part A</u>. Lactose (2.0 g, 5.8 mmol) and DMAP (70 mg, 0.58 mmol) was dissolved in pyridine (40 mL) and cooled to 0 °C. Benzoyl chloride (5.40 mL, 46.4 mmol) was then added dropwise, and the solution was warmed to room temperature and stirred overnight. After this time, ice cold water was added, and the mixture stirred 15 minutes. The mixture was then diluted with dichloromethane (15 mL) and the organic layer was extracted with 1M HCl, water and then brine and dried with Na_2SO_4. The solvent was removed by rotary evaporator and high vacuum pump to afford the desired product **10** (6.8 g, 5.8 mmol) who was used in the next step without further purification.

<u>Part B</u>. 2,3,4,6-Tetra-O-benzoyl-β-D-galactopyranosyl-(1→4)-2,3,6-tri-O-benzoyl-α-D-glucopyranosyl bromide (6.80 g, 5.8 mmol) (**10**) was dissolved in dichloromethane (50 mL) and cooled to 0 °C. HBr (33% in Acetic Acid, 15 mL) was added dropwise, and the solution warmed to room temperature and stirred overnight. After this time ice-cold water was added and the reaction was stirred for additional 15 minutes. The aqueous layer was extracted with dichloromethane, dried with Na_2SO_4 and the solvent removed by rotary evaporator and high vacuum pump to give the final compound **1d** in a quantitative yield. No further purification steps are needed.

2,3,4,6-Tetra-O-benzoyl-β-D-galactopyranosyl-(1→4)-2,3,6-tri-O-benzoyl-α-D-glucopyranosyl bromide (10).

By following procedure (Part. A) reported above **10** (6.80 g, 5.8 mmol, quant.) was obtained as an amorphous white solid. ^{1}H NMR (300 MHz, $CDCl_3$) δ 8.12 (d, *J* = 7.3 Hz, 2H), 8.07 – 7.93 (m, 8H), 7.88 (t, *J* = 5.8 Hz, 4H), 7.73 (d, *J* = 7.0 Hz, 2H), 7.67 – 7.29 (m, 20H), 7.23 – 7.15 (m, 4H), 6.74 (t, *J* = 3.7 Hz, 1H), 6.20 (t, *J* = 10.3 Hz, 1H), 5.81 – 5.71 (m, 2H), 5.61 (dd, *J* = 10.3, 3.8 Hz, 1H), 5.36 (dd, *J* = 10.3, 3.2 Hz, 1H), 4.93 (d, *J* = 7.9 Hz, 1H), 4.55 (s, 2H), 4.44 – 4.26 (m, 2H), 3.89 (t, *J* = 6.7 Hz, 1H), 3.83 – 3.64 (m, 2H). $^{13}C\{^{1}H\}$ NMR (101 MHz, Chloroform-d) δ 166.3, 165.8, 165.5, 165.4, 165.2, 164.8, 164.5, 163.6, 133.8, 133.5, 133.4, 133.3, 133.2, 130.1, 130.0, 129.83, 129.78, 129.75, 129.6, 129.5, 129.4, 129.0, 128.9, 128.8, 128.6, 128.4, 128.3, 128.2, 101.2, 89.9, 75.6, 72.0, 71.4, 71.2, 70.4, 70.2, 69.9, 67.5, 61.8, 61.0. HRMS (ESI) m/z: $[M + H]^+$ calcd for $C_{68}H_{55}O_{19}$ 1175.3332, found 1175.3387.

2,3,4,6-Tetra-O-benzoyl-β-D-galactopyranosyl-(1→4)-2,3,6-tri-O-benzoyl-α-D-glucopyranosyl bromide (1d). By following procedure (Part. B) reported above **1d** (6.57 g, 5.8 mmol, quant.) was obtained as an amorphous white solid. ^{1}H NMR (300 MHz, $CDCl_3$) δ 8.06 – 7.94 (m, 9H), 7.92 – 7.86 (m, 2H), 7.76 – 7.70 (m, 2H), 7.67 – 7.27 (m, 20H), 7.21 (q, *J* = 7.7 Hz, 5H), 6.74 (d, *J* = 4.0 Hz, 1H), 6.14 (t, *J* = 9.5 Hz, 1H), 5.75 (t, *J* = 9.5 Hz, 2H), 5.39 (dd, *J* = 10.0, 3.8 Hz, 1H), 5.25 (dd, *J* = 10.0, 3.8 Hz, 1H), 4.94 (d, *J* = 8.0 Hz, 1H), 4.67 – 4.52 (m, 2H), 4.46 – 4.25 (m, 2H), 3.96 – 3.80 (m, 2H), 3.73 (dd, J = 11.0, 6.9 Hz, 1H). $^{13}C\{^{1}H\}$ NMR (101 MHz, $CDCl_3$) δ 165.7, 165.5, 165.4, 165.4, 165.2, 165.1, 164.7, 133.7, 133.5, 133.5, 133.42, 133.38, 133.30, 133.27, 130.1, 130.0, 129.8, 129.6, 129.5, 129.34, 129.31, 128.8, 128.5, 128.4, 128.32, 128.26, 101.1, 86.7, 74.8, 73.4, 71.9, 71.4, 71.3, 70.5, 69.8, 67.4, 61.5, 61.0. HRMS (ESI) m/z: $[M + H]^+$ calcd for $C_{61}H_{50}BrO_{17}$ 1133.2226, found 1133.2274.

Procedure for preparation of 2,3,4,6-Tetra-O-benzoyl-β-D-glucopyranosyl-(1→4)-2,3,6-tri-O-benzoyl-α-D-glucopyranosyl bromide (1i).[3]

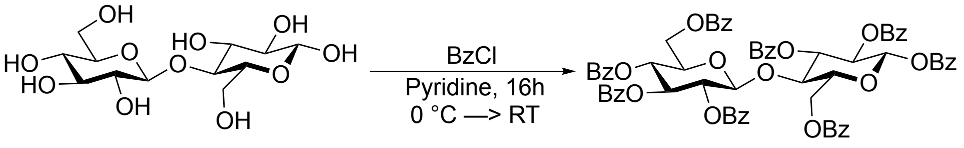

11

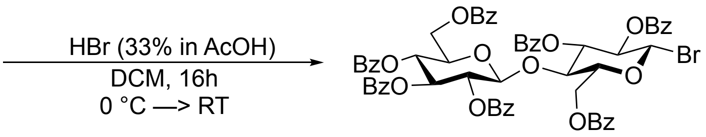

1i

<u>Part A</u>. Cellobiose (2.0 g, 5.8 mmol) and DMAP (70 mg, 0.58 mmol) were dissolved in pyridine (40 mL) and cooled to 0 °C. Benzoyl chloride (5.40 mL, 46.4 mmol) was then added dropwise, and the solution was warmed to room temperature and stirred overnight. After this time, ice cold water was added, and the mixture was stirred 15 minutes. The mixture was then diluted with dichloromethane (15 mL) and the organic layer was extracted with 1M HCl, water and brine and dried with Na_2SO_4. The solvent was removed by rotary evaporator and high vacuum pump to afford the desired product (6.8 g, 5.8 mmol) which was used in the next step without further purification.

<u>Part B</u>. 2,3,4,6-Tetra-O-benzoyl-β-D-glucopyranosyl-(1→4)-(1,2,3,6-tetra-O-benzoyl)-β-D-glucopyranose (6.80 g, 5.8 mmol) (**11**) was dissolved in dichloromethane (50 mL) and cooled to 0 °C. HBr (33% in Acetic Acid, 15 mL) was added dropwise, and the solution was warmed to room temperature and stirred overnight. After this time ice-cold water was added and the reaction was stirred for additional 15 minutes. The aqueous layer was extracted with dichloromethane, dried with Na_2SO_4 and the solvent was removed by rotary evaporator and high vacuum pump to give the final compound in a quantitative yield. No further purification steps are needed.

2,3,4,6-Tetra-O-benzoyl-β-D-glucopyranosyl-(1→4)-(1,2,3,6-tetra-O-benzoyl)-β-D-glucopyranose (11). By following procedure (Part. A) reported above **11** (6.80 g, 5.8 mmol, quant.) was obtained as an amorphous white solid.
^{1}H NMR (400 MHz, $CDCl_3$) δ 8.17 (d, *J* = 7.2 Hz, 4H), 8.00 – 7.93 (m, 8H), 7.88 (d, *J* = 7.2 Hz, 2H), 7.75 (ddd, *J* = 8.5, 5.4, 1.4 Hz, 4H), 7.71 – 7.65 (m, 2H), 7.53 (t, *J* = 7.7 Hz, 6H), 7.44 – 7.37 (m, 8H), 7.31 – 7.27 (m, 2H), 7.23 (d, J = 4.3 Hz, 2H), 6.11 (d, *J* = 8.0 Hz, 1H), 5.92 (t, *J* = 9.2 Hz, 1H), 5.77 – 5.69 (m, 2H), 5.53 (dd, *J* = 9.6, 7.9 Hz, 1H), 5.40 (t, *J* = 9.6 Hz, 1H), 4.95 (d, *J* = 7.9 Hz, 1H), 4.60 (dd, *J* = 12.3, 1.7 Hz, 1H), 4.49 (dd, *J* = 12.3, 4.0 Hz, 1H), 4.37 (t, *J* = 9.6 Hz, 1H), 4.09 – 4.00 (m, 2H), 3.85 – 3.76 (m, 2H). ^{13}C{^{1}H} NMR (101 MHz, $CDCl_3$) δ 165.74, 165.71, 165.6, 165.2, 165.0, 164.7, 164.5, 130.6, 130.1, 129.8, 129.7, 129.6, 129.6, 129.5, 129.4, 129.3, 128.9, 128.7, 128.6, 128.53, 128.52, 128.4, 128.3, 128.2, 101.0, 92.4, 76.0, 73.8, 72.7, 72.4, 71.84, 70.82, 69.3, 62.6, 62.1. HRMS (ESI) m/z: $[M + H]^+$ calcd for $C_{68}H_{55}O_{19}$ 1175.3332, found 1175.3371.

2,3,4,6-Tetra-O-benzoyl-β-D-glucopyranosyl-(1→4)-2,3,6-tri-O-benzoyl-α-D-glucopyranosyl bromide (1i). By following procedure (Part. B) reported above **1i** (6.57 g, 5.8 mmol, quant.) was obtained as an amorphous white solid. ^{1}H NMR (400 MHz, $CDCl_3$) δ 8.01 – 7.92 (m, 10H), 7.80 – 7.72 (m, 4H), 7.60 – 7.48 (m, 4H), 7.45 – 7.35 (m, 11H), 7.31 – 7.27 (m, 4H), 7.25 – 7.21 (m, 3H), 6.72 (d, *J* = 4.1 Hz, 1H), 6.13 (t, *J* = 9.5 Hz, 1H), 5.76 (t, *J* = 9.6 Hz, 1H), 5.54 (dd, *J* = 9.8, 7.9 Hz, 1H), 5.43 (t, *J* = 9.6 Hz, 1H), 5.19 (dd, *J* = 9.9, 4.1 Hz, 1H), 5.03 (d, *J* = 7.9 Hz, 1H), 4.64 (dd, *J* = 12.5, 1.9 Hz, 1H), 4.54 (dd, *J* = 12.5, 3.6 Hz, 1H), 4.43 (ddd, *J* = 10.2, 3.3, 1.8 Hz, 1H), 4.33 (dd, *J* = 9.9, 9.0 Hz, 1H), 4.09 (dd, *J* = 11.8, 3.0 Hz, 1H), 3.94 – 3.79 (m, 2H). ^{13}C{^{1}H} NMR (101 MHz, $CDCl_3$) δ 165.6, 165.4, 165.2, 165.1, 165.0, 164.8, 164.7, 133.7, 133.4, 133.2, 130.0, 129.8, 129.73, 129.70, 129.64, 129.59, 129.4, 129.3, 128.6, 128.51, 128.47, 128.39, 128.36, 128.2, 100.9, 86.5, 75.2, 73.3, 72.9, 72.5, 71.9, 71.5, 70.3, 69.3, 62.6, 61.5. HRMS (ESI) m/z: $[M + H]^+$ calcd for $C_{61}H_{50}BrO_{17}$ 1133.2226, found 1133.2258.

Procedure for preparation of methyl-2-(di(tert-butoxycarbonyl)amino)but-2-enoate (2a).[6]

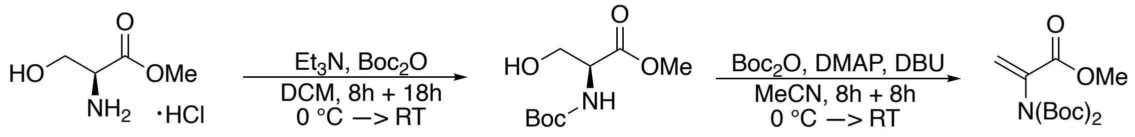

12 **2a**

Part A. To a solution of L-serine methyl ester hydrochloride (5 g, 32 mmol) in dichloromethane (40 mL) was added di-*tert*-butyl decarbonate (9.25 mL, 33.75 mmol) and triethylamine (10 mL, 70.5 mmol) at 0 °C. After 30 minutes of stirring, the solution was warmed to room temperature and stirred additional 18 hours. The solution was diluted with ice-cold water and the organic phase was washed with 1M HCl, satd. $NaHCO_3$ solution and brine. The organic layer wad dried with anhydrous Na_2SO_4, filtered and the solvent removed by rotary evaporator and high vacuum pump. The crude product was passed through a silica plug (cyclohexane:EtOAc 1:1) to give methyl (tert-butoxycarbonyl)-L-serinate (**12**) as a colorless oil (6.31 g, 28.8 mmol, 90% Yield).
Part B. methyl (tert-butoxycarbonyl)-L-serinate (**12**) (1.84 g, 13.2 mmol) was dissolved in acetonitrile (15 mL) at 0 °C and to the solution was added di-*tert*-butyl dicarbonate (3 mL, 13.2 mmol), 4-dimethylaminopyridine (146 mg, 1.2 mmol). The solution was warmed to room temperature and stirred for 8 hours. After this time DBU (90 µL, 0.6 mmol) was added and the solution was stirred for additional 8 hours. The solution was concentrated with rotary evaporator, diluted with EtOAc and washed with 1M HCl and satd. $NaHCO_3$. The organic layer was dried with anhydrous Na_2SO_4, filtered and the solvent was removed by rotary evaporator and high vacuum pump. The residue was purified by flash column chromatography column (cyclohexane:ethyl acetate 8:2) to give the final product **2a** as an amorphous white solid (3.38 g, 11.22 mmol, 85% Yield).

Methyl (tert-butoxycarbonyl)-L-serinate (12).

By following procedure (Part. A) reported above **12** (1.84 g, 13.2 mmol) was obtained as a colorless oil. ^{1}H NMR (300 MHz, $CDCl_3$) δ 5.44 (s, 1H), 4.39 (s, 1H), 4.00 – 3.89 (m, 2H), 3.79 (s, 3H), 1.45 (s, 9H). ^{13}C{^{1}H} NMR (101 MHz, $CDCl_3$) δ 171.3, 63.6, 55.7, 52.7, 28.3. HRMS (ESI) m/z: $[M + H]^+$ calcd for $C_9H_{18}NO_5$ 220.1179, found 220.1188.

Methyl-2-(di(tert-butoxycarbonyl)amino)but-2-enoate (2a).

By following procedure (Part. B) reported above **2a** (3.38 g, 11.22 mmol, 85% Yield) was obtained as an amorphous white solid. ^{1}H NMR (300 MHz, $CDCl_3$) δ 6.34 (s, 1H), 5.65 (s, 1H), 3.80 (s, 3H), 1.46 (s, 18H). ^{13}C{^{1}H} NMR (101 MHz, $CDCl_3$) δ 164.0, 150.6, 136.1, 124.6, 83.1, 52.3, 27.8. HRMS (ESI) m/z: $[M + H]^+$ calcd for $C_{14}H_{24}NO_6$ 302.1598, found 302.1587.

Procedure for preparation of methyl (2S)-2-((tert-butoxycarbonyl)amino)-3-hydroxybutanoate (2b).[6]

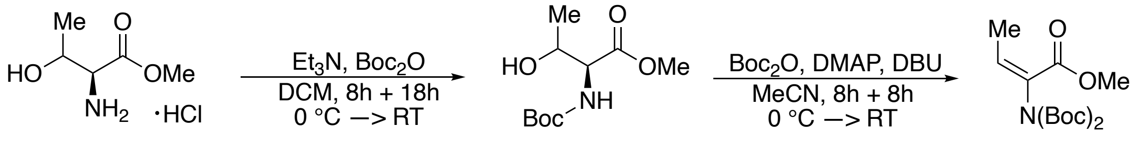

13 **2b**

Part A. To a solution of L-threonine methyl ester hydrochloride (1 g, 6 mmol) in dichloromethane (10 mL) was added di-*tert*-butyl decarbonate (1.5 mL, 6.6 mmol) and triethylamine (2.5 mL, 18 mmol) at 0 °C. After 30 minutes of stirring, the solution was warmed to room temperature and stirred additional 18 hours. The solution was diluted with ice-cold water and the organic phase was washed with 1M HCl, satd. $NaHCO_3$ solution and brine. The organic layer wad dried with anhydrous Na_2SO_4, filtered and the solvent removed by rotary evaporator and high vacuum pump. The crude product was passed through a silica plug (cyclohexane:EtOAc 1:1) to give methyl (2*S*)-2-((tert-butoxycarbonyl)amino)-3-hydroxybutanoate (**13**) as a pale red oil (1.32 g, 5.67 mmol, 86% Yield).

Part B. methyl (2*S*)-2-((tert-butoxycarbonyl)amino)-3-hydroxybutanoate (**13**) (1.32 g, 5.67 mmol) was dissolved in acetonitrile (10 mL) at 0 °C and to the solution was added di-*tert*-butyl dicarbonate (2.63 mL, 11.76 mmol), 4-dimethylaminopyridine (68 mg, 0.56 mmol). The solution was warmed to room temperature and stirred for 8 hours. After this time DBU (179 μL, 1.12 mmol) was added and the solution was stirred for additional 8 hours. The solution was concentrated with rotary evaporator, diluted with EtOAc and washed with 1M HCl and satd. $NaHCO_3$. The organic layer was dried with anhydrous Na_2SO_4, filtered and the solvent was removed by rotary evaporator and high vacuum pump. The residue was purified by flash column chromatography column (cyclohexane:ethyl acetate 8:2) to give the final product as a colorless oil (1.43 g, 4.54 mmol, 80% Yield).

Methyl (2S)-2-((tert-butoxycarbonyl)amino)-3-hydroxybutanoate (13).

By following procedure (Part. A) reported above **13** (1.32 g, 5.67 mmol, 86% Yield) was obtained as a pale red oil. ^{1}H NMR (400 MHz, $CDCl_3$) δ 5.30 (s, 1H), 4.27 (s, 2H), 3.77 (s, 4H), 1.93 (br s, 1H), 1.45 (s, 9H), 1.25 (d, J = 6.4 Hz, 3H). ^{13}C{^{1}H} NMR (101 MHz, $CDCl_3$) δ 172.0, 156.1, 146.7, 85.2, 80.1, 68.1, 58.6, 52.5, 28.3, 27.4, 19.9. HRMS (ESI) m/z: $[M + H]^+$ calcd for $C_{10}H_{20}NO_5$ 234.1336, found 234.1341.

Methyl-2-(di(tert-butoxycarbonyl)amino)but-2-enoate (2b).

By following procedure (Part. B) reported above **2b** (1.43 g, 4.54 mmol, 80% Yield) was obtained as a colorless oil. ^{1}H NMR (300 MHz, $CDCl_3$) δ 6.88 (q, *J* = 7.2 Hz, 1H), 3.76 (s, 3H), 1.75 (d, *J* = 7.1 Hz, 3H), 1.44 (s, 18H). ^{13}C{^{1}H} NMR (101 MHz, $CDCl_3$) δ 164.4, 150.5, 136.6, 130.3, 82.8, 52.1, 27.9, 13.3. HRMS (ESI) m/z: $[M + H]^+$ calcd for $C_{10}H_{18}NO_4$ 216.1230, found 216.1221.

Procedure for preparation of methyl 2-(phenylamino)but-2-enoate (2c).[6]

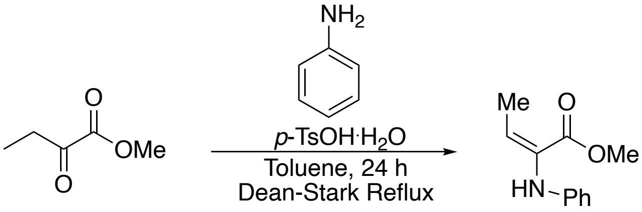

2c

In a round bottom flask, to a solution of methyl-2-oxobutanoate (1 g, 8.6 mmol) in Toluene (25 mL) was added aniline (780 μL, 8.6 mmol) and *p*-toluensulfonic acid monohydrate (82 mg, 0.43 mmol). A Dean-Stark apparatus and a condenser were attached, and the solution was warmed to 95 °C and stirred 24 hours. The mixture was then concentrated at the rotary evaporator and purified by flash column chromatography (cyclohexane:ethyl acetate 9:1) to give the product (1.05 g ,5.5 mmol ,64% Yield) as a pale orange oil.

Methyl 2-(phenylamino)but-2-enoate (2c).
By following procedure reported above **2c** (1.05 g, 5.5 mmol, 64% Yield) was obtained as a pale orange oil. ^{1}H NMR (300 MHz, $CDCl_3$) δ 7.21 (t, *J* = 8.4 Hz, 2H), 6.82 (t, *J* = 7.3 Hz, 1H), 6.69 – 6.53 (m, 3H), 5.49 (s, 1H), 3.77 (s, 3H), 1.70 (d, *J* = 7.3 Hz, 3H). ^{13}C{^{1}H} NMR (101 MHz,$CDCl_3$) δ 166.3, 144.1, 129.0, 127.0, 119.5, 115.4, 52.3, 14.5. HRMS (ESI) m/z: $[M + H]^+$ calcd for $C_{11}H_{14}NO_2$ 192.1019, found 192.1014.

Procedure for preparation of 2-aminoacrylate (2e).[7]

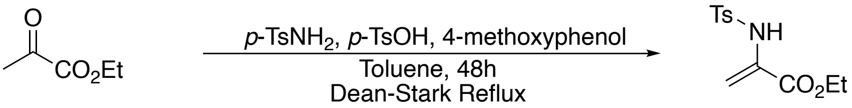

2e

In a round bottom flask, to a solution of ethyl pyruvate (1.27 mL, 11.45 mmol) in Toluene (25 mL) was added *p*-$TsNH_2$ (2.17 g, 12.7 mmol), *p*-TsOH (240 mg, 1.3 mmol) and 4-methoxyphenol (2 mg, 0.013 mmol). A Dean-Stark apparatus and a condenser were attached, and the solution was warmed to 95 °C and stirred 48 hours. The mixture was then concentrated at the rotary evaporator, taken up in dichloromethane and washed with satd. $NaHCO_3$ solution and water. The organic phase was dried with anhydrous Na_2SO_4, filtered, and concentrated by rotary evaporator and high vacuum pump. The residue was purified by flash column chromatography (cyclohexane:ethyl acetate 8:2) to give the final product (935 mg ,5.9 mmol ,52% Yield) as a yellow oil.

2-aminoacrylate (2e).
By following procedure reported above **2e** (935 mg, 5.9 mmol, 52% Yield) was obtained as a yellow oil. ^{1}H NMR (300 MHz, $CDCDl_3$) δ 7.75 (d, *J* = 8.3 Hz, 2H), 7.30 (d, *J* = 8.0 Hz, 2H), 7.12 (s, 1H), 5.64 (s, 2H), 4.19 (q, *J* = 7.1 Hz, 2H), 2.42 (s, 4H), 1.25 (t, *J* = 7.1 Hz, 4H). ^{13}C{^{1}H} NMR (101 MHz, $CDCl_3$) δ 163.1, 144.3, 135.5, 131.0, 129.7, 127.6, 106.7, 62.5, 21.6, 14.0. HRMS (ESI) m/z: $[M + H]^+$ calcd for $C_{12}H_{16}NO_4S$ 270.0795, found 270.0801.

Procedure for preparation of ethyl 2-(Acetylamino)acrylate (2f).[8]

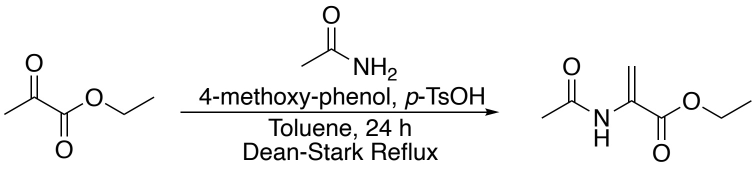

2f

In a round bottom flask, to a solution of ethyl pyruvate (1.27 mL, 11.45 mmol) in Toluene (25 mL) was added acetamide (750 mg, 12.7 mmol), *p*-toluensulfonic acid monohydrate (240 mg, 1.3 mmol) and 4-methoxyphenol (4 mg, 0.026 mmol). A Dean-Stark apparatus and a condenser were attached, and the solution was warmed to 95 °C and stirred 24 hours. The mixture was then concentrated at the rotary evaporator, taken up in dichloromethane and washed with satd. $NaHCO_3$ solution and water. The organic phase was dried with anhydrous Na_2SO_4, filtered, and concentrated by rotary evaporator and high vacuum pump. The residue was purified by flash column chromatography (cyclohexane:ethyl acetate 8:2) to give the final product **2f** (1.06 g ,7.44 mmol ,65% Yield) as a yellow oil.

Ethyl 2-(Acetylamino)acrylate (2f).
By following procedure reported above **2f** (1.17 g, 7.44 mmol, 65% Yield) was obtained as a yellow oil. ^{1}H NMR (400 MHz, $CDCl_3$) δ 7.75 (s, 1H), 6.58 (s, 1H), 5.88 (s, 1H), 4.29 (q, *J* = 7.2 Hz, 2H), 2.13 (s, 3H), 1.34 (t, *J* = 7.1 Hz, 3H). ^{13}C{^{1}H} NMR (101 MHz, $CDCl_3$) δ 168.8, 164.2, 131.0, 108.4, 62.2, 24.7, 14.1. HRMS (ESI) m/z: $[M + H]^+$ calcd for $C_7H_{12}NO_3$ 158.0812, found 158.0816.

Procedure for preparation of diethyl 2,6-dimethyl-1,4-dihydropyridine-3,5-dicarboxylate (HE-1).[9]

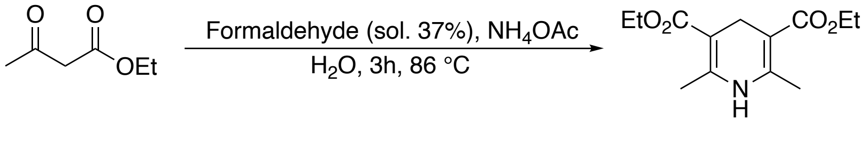

To a round bottom flask equipped with a condenser were added formaldehyde (37% aqueous solution, 1.9 mL, 25 mmol), ethyl acetoacetate (12.5 mL, 100 mmol), ammonium acetate (3.9 g, 50 mmol) and distilled water (50 mL). The solution was warmed to 86 °C in an oil bath and stirred for 3h. The mixture was then filtered, and the yellow balls formed were pulverized and washed with ice-cold water and cold acetone. The solid was dried in vacuum to afford the product **HE-1** as a yellow amorphous solid (6.2g, 24.5mmol,98% Yield).

Diethyl 2,6-dimethyl-1,4-dihydropyridine-3,5-dicarboxylate (HE-1).
By following procedure reported above **HE-1** (6.2 g, 24.5 mmol, 98% Yield) was obtained as a yellow amorphous solid. ^{1}H NMR (300 MHz, $CDCl_3$) δ 5.12 (s, 1H), 4.17 (q, *J* = 7.1 Hz, 4H), 3.26 (s, 2H), 2.19 (s, 6H), 1.28 (t, *J* = 7.1 Hz, 6H). ^{13}C{^{1}H} NMR (101 MHz, $CDCl_3$) δ 168.0, 144.8, 99.5, 59.6, 24.8, 19.1, 14.4. HRMS (ESI) m/z: $[M + H]^+$ calcd for $C_{13}H_{20}NO_4$ 254.1387, found 254.1391.

Procedure for preparation of diethyl 2,6-dimethyl-4-phenyl-1,4-dihydropyridine-3,5-dicarboxylate (HE-2).[9]

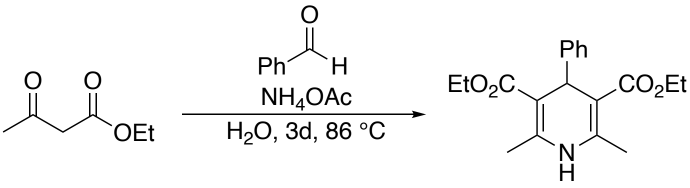

To a round bottom flask equipped with a condenser were added Ethyl acetoacetate (11.5 mL, 60 mmol), benzaldehyde (5 mL, 30 mmol) and ethanol (anhydrous, 50 mL). The mixture was warmed to reflux for 60 minutes, and ammonium acetate (1.92 g, 30 mmol) was added. The reaction was stirred under reflux for 3 days. Then the solution was cooled down and diluted with brine (100 mL) and extracted with ethyl acetate (3x50 mL). The cabined organic layers were dried with with anhydrous Na_2SO_4, filtered, and concentrated by rotary evaporator and high vacuum pump. The mixture was triturated with pentane to give the final product **HE-2** (6.9 g, 21 mmol, 70%) as a yellow amorphous solid.

Diethyl 2,6-dimethyl-4-phenyl-1,4-dihydropyridine-3,5-dicarboxylate (HE-2).
By following procedure reported above **HE-2** (6.9 g, 21 mmol, 70% Yield) was obtained as a yellow amorphous solid. ^{1}H NMR (300 MHz, $CDCl_3$) δ 7.30 – 7.10 (m, 5H), 5.61 (s, 1H), 4.98 (s, 1H), 4.12 – 4.01 (m, 4H), 2.32 (s, 6H), 1.21 (t, *J* = 7.1 Hz, 6H). ^{13}C{^{1}H} NMR (101 MHz, $CDCl_3$) δ 167.6, 147.7, 143.8, 128.0, 127.8, 126.1, 104.1, 59.7, 39.6, 19.6, 14.2. HRMS (ESI) m/z: $[M + H]^+$ calcd for $C_{19}H_{24}NO_4$ 330.1700, found 330.1691.

Procedure for preparation of diethyl 2,6-dimethyl-4-(o-tolyl)-1,4-dihydropyridine-3,5-dicarboxylate (HE-3).[9]

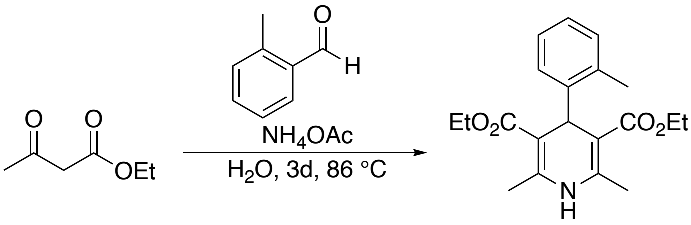

To a round bottom flask equipped with a condenser were added Ethyl acetoacetate (11.5 mL, 60 mmol), 2-methylbenzaldehyde (4 mL, 30 mmol) and ethanol (anhydrous, 50 mL). The mixture was warmed to reflux for 60 minutes, and ammonium acetate (1.92 g, 30 mmol) was added. The reaction was stirred under reflux for 3 days. Then the solution was cooled down and diluted with brine (100 mL) and extracted with ethyl acetate (3x50 mL). The cabined organic layers were dried with anhydrous Na_2SO_4, filtered, and concentrated by rotary evaporator and high vacuum pump. The mixture was purified with flash column chromatography (cyclohexane:ethyl acetate 8:2) to give the final product **HE-3** (6.7 g, 19.5 mmol, 65%) as a yellow amorphous solid.

Diethyl 2,6-dimethyl-4-(o-tolyl)-1,4-dihydropyridine-3,5-dicarboxylate (HE-3).
By following procedure reported above **HE-3** (6.7 g, 19.5 mmol, 65% Yield) was obtained as a yellow amorphous solid. ^{1}H NMR (300 MHz, $CDCl_3$) δ 7.30 (d, *J* = 7.1 Hz, 1H), 7.08 – 6.96 (m, 3H), 5.55 (s, 1H), 5.16 (s, 1H), 4.15 – 4.01 (m, 4H), 2.56 (s, 3H), 2.31 (s, 6H), 1.20 (t, *J* = 7.1 Hz, 6H). ^{13}C{^{1}H} NMR (101 MHz, $CDCl_3$) δ 167.8, 147.4, 143.3, 135.1, 129.8, 129.4, 126.02, 126.01, 105.1, 59.7, 35.7, 19.8, 19.5, 14.4. HRMS (ESI) m/z: $[M + H]^+$ calcd for $C_{20}H_{26}NO_4$ 344.1856, found 344.1839.

SYNTHESIS OF PRODUCTS

General procedure A for the synthesis of C-glycosyl amino acids.

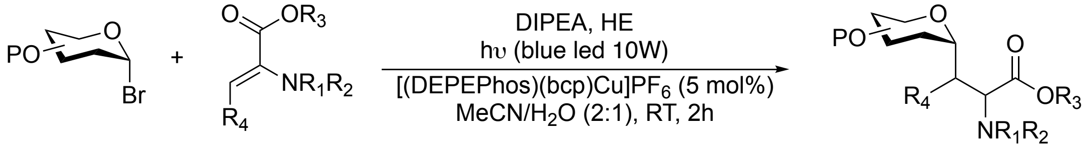

Protected bromide sugar (0.12 mmol, 1 eq.), acceptor (0.24 mmol, 2 eq.), Hantzsch ester (60 mg, 0.24 mmol, 2 eq.), DIPEA (62 µL, 0.36 mmol, 3 eq.) and [(DPEPhos)(bcp)Cu]PF_6 (5 mol%, 6.6 mg, 0.006 mmol, 0.05 eq.) were inserted in a sealed microwave vial. The internal atmosphere was saturated with argon after 3 cycles of vacuum/argon. Then 1 mL of MeCN/H_2O (2:1) (deaerated with freeze and pump) was added and the reaction was placed at 5 cm from the light source (blue led 10 W) and stirred and irradiated for 2 hours at room temperature. After this time the mixture was extracted with DCM (3x5 mL), dried with anhydrous Na_2SO_4, filtered, concentrated by rotary evaporator and purified with flash column chromatography to give the final product.

General procedure B for the synthesis of C-glycosyl amino acids.

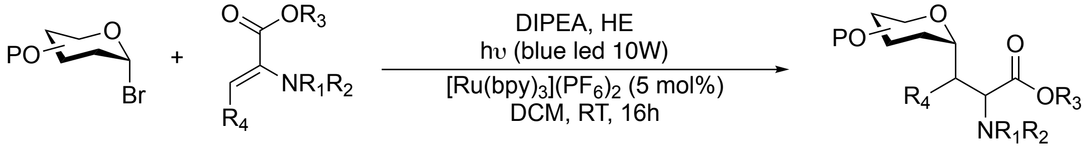

Protected bromide sugar (0.12 mmol, 1 eq.), acceptor (0.24 mmol, 2 equiv), Hantzsch ester (60 mg, 0.24 mmol, 2 eq.), DIPEA (62 µL, 0.36 mmol, 3 eq.) and $[Ru(bpy)_3](PF_6)_2$ (5 mol%, 4 mg, 0.006 mmol, 0.05 eq.) were inserted in a sealed microwave vial. The internal atmosphere was saturated with argon after 3 cycles of vacuum/argon. Then 1 mL of DCM (deaerated with freeze and pump) was added, and the reaction was placed at 5 cm from the light source (blue led 10 W) and stirred and irradiated for 16 hours at room temperature. After this time the mixture was concentrated by rotary evaporator and purified with flash column chromatography to give the final product.

Methyl[3-α-(2,3,4,6-tetra-*O*-Benzoyl-α-D-glucopyranosyl)]-(2S/2R)-N,N-di-*tert*-buthoxycarbonyl-alanine (3aa).

By following general procedure A (**3aa**) was obtained as a white amorphous solid (102 mg, 0.116 mmol, 97%) in a mixture of two diastereomers in ratio (1:0.8) after flash chromatography (cyclohexane:ethyl acetate 8:2). ^{1}H NMR (400 MHz, $CDCl_3$) δ 8.06 – 7.87 (m, 14.4H), 7.52 – 7.30 (m, 21.6H), 6.04 (t, *J* = 9.3 Hz, 0.8H), 5.97 (t, *J* = 8.9 Hz, 1H), 5.69 – 5.61 (m, 1.8H), 5.61 – 5.54 (m, 1.8H), 5.28 – 5.19 (m, 1.8H), 4.87 (ddd, *J* = 11.7, 5.4, 2.7 Hz, 0.8H), 4.62 – 4.29 (m, 7.4H), 3.68 (s, 3H), 3.65 (s, 2.4H), 3.00 (td, *J* = 14.6, 13.2, 3.3 Hz, 1H), 2.66 (ddd, *J* = 14.9, 7.9, 2.9 Hz, 0.8H), 2.45 (ddd, *J* = 15.0, 12.0, 5.3 Hz, 0.8H), 2.29 (ddd, *J* = 15.5, 10.4, 2.7 Hz, 1H), 1.45 (s, 14.4H), 1.36 (s, 18H). $^{13}C\{^1H\}$ NMR (101 MHz, $CDCl_3$) δ 171.3, 170.6, 166.2, 166.1, 165.7, 165.23, 165.19, 165.10, 152.0, 151.8, 133.5, 133.4, 133.31, 133.28, 133.27, 133.0, 129.9, 129.8, 129.73, 129.70, 129.0, 128.95, 128.89, 128.79, 128.75, 128.67, 128.5, 128.4, 128.3, 83.6, 83.4, 71.0, 70.8, 70.7, 70.3, 69.9, 69.7, 69.6, 69.4, 69.2, 63.3, 62.6, 54.8, 54.2, 52.5, 52.3, 27.9, 27.8. HRMS (ESI) m/z: $[M + H]^+$ calcd for $C_{48}H_{52}NO_{15}$ 882.3331, found 882.3371.

Methyl[3-α-(2,3,4,6-tetra-*O*-Benzoyl-α-D-mannopyranosyl)]-(2S/2R)-N,N-di-*tert*-buthoxycarbonyl-alanine (3ba).

By following general procedure A (**3ba**) was obtained as a white amorphous solid (74 mg, 0.08 mmol, 70%) in a mixture of two diastereomers in ratio (1:0.5) after flash chromatography (cyclohexane:ethyl acetate 8:2). ^{1}H NMR (400 MHz, $CDCl_3$) δ 8.10 (d, J = 7.1 Hz, 1H), 8.08 – 8.02 (m, 5H), 7.97 (d, *J* = 7.1 Hz, 2H), 7.93 (d, *J* = 7.1 Hz, 1H), 7.87 – 7.81 (m, 3H), 7.62 – 7.33 (m, 15H), 7.32 – 7.26 (m, 3H), 6.10 – 6.00 (m, 1.5H), 5.86 – 5.77 (m, 1.5H), 5.71 (t, *J* = 3.0 Hz, 0.5H), 5.66 (t, *J* = 2.9 Hz, 1H), 5.27 (ddd, *J* = 16.8, 8.6, 4.2 Hz, 1.5H), 4.66 (dd, *J* = 12.0, 3.3 Hz, 2H), 4.57 (dd, *J* = 12.1, 4.1 Hz, 0.5H), 4.50 (dd, *J* = 11.9, 4.9 Hz, 1H), 4.45 – 4.37 (m, 1H), 4.37 – 4.25 (m, 1.5H), 3.72 (s, 1.5H), 3.70 (s, 3H), 3.07 (ddd, *J* = 15.6, 12.4, 3.4 Hz, 1H), 2.72 (ddd, *J* = 14.5, 7.9, 3.4 Hz, 0.5H), 2.50 – 2.40 (m, 0.5H), 2.28 (ddd, *J* = 15.2, 9.2, 2.9 Hz, 1H), 1.49 (s, 9H), 1.47 (s, 18H). $^{13}C\{^1H\}$ NMR (101 MHz, $CDCl_3$) δ 171.1, 170.5, 166.2, 166.0, 165.7, 165.54, 165.48, 165.40, 165.2, 152.1, 151.9, 133.4, 133.35, 133.27, 132.9, 130.0, 129.9, 129.8, 129.75, 129.72, 129.6, 129.5, 129.1, 129.0, 128.52, 128.50, 128.4, 128.3, 83.6, 83.6, 73.7, 73.0, 71.6, 70.5, 70.4, 70.2, 70.1, 67.5, 67.4, 63.2, 62.7, 54.73, 54.70, 52.5, 52.4, 30.2, 29.7, 28.0, 27.9. HRMS (ESI) m/z: $[M + H]^+$ calcd for $C_{48}H_{52}NO_{15}$ 882.3331, found 882.3288.

Methyl[3-α-(2,3,4,6-tetra-*O*-Benzoyl-α-D-galactopyranosyl)]-(2S/2R)-N,N-di-*tert*-buthoxycarbonyl-alanine (3ca).

By following general procedure A after flash chromatography (cyclohexane:ethyl acetate 9:1), (**3ca**) was obtained as a white amorphous solid (118 mg, 0.118 mmol, 98%) in a mixture of two diastereomers (d.r. 60:40). In this case flash chromatography allows separation of the two diastereomers. Diast. A: ^{1}H NMR (400 MHz, $CDCl_3$) δ 8.03 (d, *J* = 7.1 Hz, 2H), 7.99 (d, *J* = 8.4 Hz, 4H), 7.86 (d, *J* = 7.1 Hz, 2H), 7.60 – 7.36 (m, 11H), 7.33 – 7.27 (m, 2H), 6.02 (t, *J* = 2.9 Hz, 1H), 5.88 – 5.83 (m, 1H), 5.24 (dd, *J* = 8.1, 5.1 Hz, 1H), 5.02 – 4.95 (m, 1H), 4.76 (dd, *J* = 11.2, 6.7 Hz, 1H), 4.55 (td, *J* = 6.4, 2.6 Hz, 1H), 4.40 (dd, *J* = 11.2, 6.5 Hz, 1H), 3.65 (s, 3H), 2.63 (ddd, *J* = 14.8, 8.2, 3.1 Hz, 1H), 2.32 (ddt, *J* = 12.7, 10.0, 5.0 Hz, 1H), 1.44 (s, 18H). $^{13}C\{^1H\}$ NMR (101 MHz, $CDCl_3$) δ 170.6, 166.0, 165.6, 165.5, 165.3, 152.0, 133.5, 133.4, 133.3, 133.0, 129.9, 129.84, 129.82, 129.7, 129.6, 129.2, 129.1, 129.0, 128.6, 128.5, 128.4, 128.3, 83.5, 69.0, 68.8, 68.7, 68.4, 61.7, 54.9, 52.2, 27.9. HRMS (ESI) m/z: $[M + H]^+$ calcd for $C_{48}H_{52}NO_{15}$ 882.3331, found 882.3292.

Diast. B: ^{1}H NMR (400 MHz, $CDCl_3$) δ 8.07 (d, J = 7.1 Hz, 2H), 8.00 (d, J = 7.1 Hz, 3H), 7.94 (d, J = 7.1 Hz, 2H), 7.81 (d, J = 7.1 Hz, 2H), 7.60 (t, J = 7.4 Hz, 1H), 7.51 – 7.36 (m, 11H), 6.02 (dd, J = 3.3, 1.4 Hz, 1H), 5.93 (dd, J = 10.2, 5.8 Hz, 1H), 5.85 (dd, J = 10.3, 3.3 Hz, 1H), 5.25 (dd, J = 10.1, 3.1 Hz, 1H), 4.64 – 4.50 (m, 4H), 4.44 (dd, J = 9.5, 4.5 Hz, 1H), 3.67 (s, 3H), 2.95 (ddd, J = 15.8, 12.8, 3.1 Hz, 1H), 2.27 (ddd, J = 15.7, 10.1, 2.9 Hz, 1H), 1.39 (s, 18H). $^{13}C\{^1H\}$ NMR (101 MHz, $CDCl_3$) δ 171.2, 166.2, 166.0, 165.6, 165.4, 151.9, 133.5, 133.2, 133.0, 129.9, 129.8, 129.7, 128.6, 128.5, 128.33, 128.31, 83.3, 70.3, 68.9, 68.8, 68.6, 68.3, 62.4, 54.4, 52.5, 27.8. HRMS (ESI) m/z: $[M + H]^+$ calcd for $C_{48}H_{52}NO_{15}$ 882.3331, found 882.3364.

Methyl[3-α(2,3,4,6-tetra-*O*-Benzoyl-α-D-galactopyranosyl)]-(2*S*/2*R*)-N,N-di-*tert*-buthoxycarbonyl-(3S/3R)-methyl-alanine (3ab).

By following general procedure A (**3ab**) was obtained as a white amorphous solid (97 mg, 0.108 mmol, 45%) in a mixture of four diastereomers in ratio (1:0.9:0.6:0.4) after flash chromatography (cyclohexane:ethyl acetate 8:2)^{1}H NMR (400 MHz, $CDCl_3$) δ 8.22 (d, *J* = 8.0 Hz, 1.8H), 8.15 (t, *J* = 7.7 Hz, 4H), 8.10 – 7.97 (m, 11.6H), 7.93 (d, *J* = 8.1 Hz, 2H), 7.88 – 7.77 (m, 2.6H), 7.72 (d, *J* = 8.3 Hz, 1.2H), 7.70 – 7.30 (m, 31.9H), 7.24 – 7.01 (m, 2.9H), 5.91 (t, *J* = 9.4 Hz, 0.4H), 5.80 – 5.70 (m, 2.5H), 5.69 – 5.57 (m, 0.9H), 5.46-5.54 (m, 1H), 5.43 (d, *J* = 3.4 Hz, 2.5H), 5.38 (d, *J* = 4.3 Hz, 1.9H), 5.30 (d, *J* = 5.3 Hz, 0.9H), 4.97 (d, *J* = 6.5 Hz, 1H), 4.92 (d, *J* = 9.3 Hz,0.4H), 4.82 – 4.55 (m, 5.3H), 4.46 – 4.37 (m, 1.9H), 4.27 (dd, *J* = 9.5, 2.8 Hz, 0.6H), 3.92 (dd, *J* = 23.3, 10.1 Hz, 1H), 3.67 (s, 1.4H), 3.58 (s, 1.8H), 3.57 (s, 2.7H), 3.43 (s, 3H), 2.93 (s, 1.6H), 2.72 – 2.49 (m, 1.5H), 2.37 – 2.32 (m, 0.4H), 2.29 – 2.21 (m, 0.4H), 2.01 – 1.94 (m, 0.9H), 1.93 – 1.84 (m, 1H), 1.47 (s, 18H), 1.45 (s, 7.2H), 1.44 (s, 10.8H), 1.39 (s, 16.2H), 1.35 (d, J = 6.8 Hz, 2.7H), 1.30 (d, J = 6.7 Hz, 1.8H), 1.12 (d, J = 7.1 Hz, 1.2H), 0.89 (d, J = 7.2 Hz, 3H diast. D). ^{13}C NMR (101 MHz, $CDCl_3$) δ 171.6, 171.4, 170.8, 170.0, 169.93, 169.87, 167.83, 167.1, 167.0, 166.02, 165.94, 165.5, 165.2, 164.6, 152.5, 133.7, 133.6, 133.5, 133.42, 133.38, 133.30, 133.2, 133.1, 133.0, 132.9, 130.2, 130.14, 130.11, 129.91, 129.87, 129.86, 129.82, 129.75, 129.70, 129.6, 129.1, 129.0, 128.6, 128.52, 128.49, 128.43, 128.33, 128.29, 128.1, 128.1, 83.22, 83.16, 73.3, 73.2, 73.1, 69.2, 69.13, 69.06, 69.02, 68.63, 68.58, 67.61, 67.56, 62.1, 59.6, 59.5, 51.9, 51.8, 36.5, 36.4, 27.81, 27.76, 26.9, 14.2, 13.35, 13.30. HRMS (ESI) m/z: $[M + H]^+$ calcd for $C_{49}H_{54}NO_{15}$ 896.3488, found 896.3529.

Methyl[3-α(2,3,4,6-tetra-*O*-Benzoyl-α-D-galactopyranosyl]-(2S/2R)-N-phenyl-(3S/3R)-methyl-alanine (3ac).

By following general procedure A (**3ac**) was obtained as a white amorphous solid (120 mg, 0.16 mmol, 65%) in a mixture of four diastereomers in ratio (1:0.9:0.8:0.4) after flash chromatography (cyclohexane:ethyl acetate 95:15). ^{1}H NMR (400 MHz, $CDCl_3$) δ 8.21 – 8.03 (m, 12.4H), 7.98 – 7.80 (m, 12.4H), 7.65 – 7.28 (m, 37.2H), 7.20 – 7.09 (m, 5.4H), 7.09 – 6.98 (m, 0.8H), 6.82 (dd, *J* = 8.5, 7.4 Hz, 1.9H), 6.72 (t, *J* = 7.3 Hz, 0.8H), 6.66 (d, *J* = 7.6 Hz, 2H), 6.63-6.55 (m, 3.8H), 6.51 (d, *J* = 7.6 Hz, 0.8H), 5.95 – 5.83 (m, 2.3H), 5.77-5.69 (m, 1.7H), 5.66 (t, *J* = 9.7 Hz, 1H), 5.62 – 5.48 (m, 1.2H), 5.47 – 5.41 (m, 1.3H), 5.38 – 5.33 (m, 1.8H), 5.33 – 5.30 (m, 0.9H), 5.21 – 5.19 (m, 1.3H), 5.19 – 5.15 (m, 1H), 5.14 (d, J = 3.5 Hz, 0.8H), 5.11 (d, J = 3.0 Hz, 0.9H), 5.08 (d, *J* = 2.4 Hz, 0.9H), 5. 05 (d, *J* = 7.4 Hz, 0.4H), 4.79 – 4.74 (m, 0.8H), 4.71 (dd, J = 7.3, 2.0 Hz, 0.4H), 4.67 – 4.55 (m, 1.9H), 4.53 – 4.38 (m, 2.3H), 4.32 (dd, *J* = 11.7, 3.6 Hz, 0.8H), 4.20 – 4.09 (m, 1.2H), 4.09 – 4.01 (m, 1.9H), 3.67 (s, 3H), 3.65 (s, 1.2H), 3.58 (s, 2.7H), 3.55 (s, 2.4H), 2.79 – 2.58 (m, 2.7H), 2.58 – 2.44 (m, 0.4H), 2.42 – 2.02 (m, 3.1H), 1.14 (d, J = 7.0 Hz, 1.2H), 1.08 (d, J = 7.0 Hz, 2.4H), 1.00 (d, J = 6.8 Hz, 2.7H), 0.92 (d, J = 7.0 Hz, 3H). ^{13}C NMR (101 MHz, $CDCl_3$) δ 174.2, 174.0, 173.6, 173.2, 173.1, 172.8, 172.0, 166.4, 166.2, 166.1, 165.93, 165.89, 165.7, 165.5, 165.40, 165.36, 165.32, 165.2, 164.6, 164.5, 164.2, 146.9, 146.5, 145.7, 133.9, 133.8, 133.7, 133.6, 133.5, 133.4, 133.30, 133.28, 133.21, 133.1, 130.2, 130.2, 130.1, 130.0, 129.9, 129.8, 129.72, 129.67, 129.3, 129.0, 128.8, 128.74, 128.66, 128.56, 128.52, 128.44, 128.36, 128.32, 128.2, 118.9, 118.2, 118.1, 114.0, 113.6, 113.5, 113.4, 73.9, 73.8, 73.6, 73.2, 70.1, 69.7, 69.4, 69.0, 68.6, 68.1, 67.9, 67.8, 67.6, 67.5, 67.2, 67.0, 66.9, 63.2, 61.2, 61.1, 61.0, 57.2, 56.9, 56.8, 52.1, 52.0, 51.8, 36.7, 35.8, 35.6, 28.3. HRMS (ESI) m/z: $[M + H]^+$ calcd for $C_{42}H_{42}NO_{11}$ 772.2752, found 772.2733.

Methyl 3-(2,3,4,6-tetra-O-benzoyl-α-D-glucopyranosyl)propanoate (3ad).

By following general procedure A after flash chromatography (cyclohexane:ethyl acetate 8:2), **(3ad)** was obtained as a white amorphous solid (160 mg, 0.24 mmol, quant.). ^{1}H NMR (400 MHz, $CDCl_3$) δ 8.05 (d, *J* = 7.1 Hz, 2H), 7.99 (d, *J* = 7.1 Hz, 2H), 7.94 – 7.88 (m, 4H), 7.59 – 7.36 (m, 8H), 7.33 (t, *J* = 7.8 Hz, 4H), 5.99 (t, *J* = 8.8 Hz, 1H), 5.58 (t, *J* = 8.6 Hz, 1H), 5.52 (dd, *J* = 9.1, 5.5 Hz, 1H), 4.60 – 4.46 (m, 3H), 4.36 – 4.27 (m, 1H), 3.65 (s, 3H), 2.62 – 2.30 (m, 3H), 2.10 – 1.97 (m, 1H). ^{13}C NMR (101 MHz, Chloroform-d) δ 173.3, 166.2, 165.6, 165.35, 165.26, 133.5, 133.4, 133.3, 133.1, 129.92, 129.89, 129.7, 129.6, 129.05, 129.02, 128.8, 128.5, 128.4, 72.0, 71.0, 70.2, 69.8, 69.4, 63.0, 51.7, 29.7, 21.3. HRMS (ESI) m/z: $[M + H]^+$ calcd for $C_{38}H_{35}NO_{11}$ 667.2124, found 667.2149.

Methyl[3-α(2,3,4,6-Tetra-O-benzoyl-β-D-galactopyranosyl-(1→4)-2,3,6-tri-O-benzoyl-α-D-glucopyranosyl]-(2S/2R)-N,N-di-*tert*-buthoxycarbonyl-alanine (3da).

By following general procedure A (**3da**) was obtained as a white amorphous solid (325 mg, 0.24 mmol, 99%) in a mixture of two diastereomers in ratio (1:0.85) after flash chromatography (cyclohexane:ethyl acetate 8:2). ^{1}H NMR (400 MHz, $CDCl_3$) δ 8.06 – 7.93 (m, 18.5H), 7.88 – 7.82 (m, 3.7H), 7.76 – 7.68 (m, 3.7H), 7.66 – 7.28 (m, 38.85H), 7.25 – 7.10 (m, 5.55H), 5.96 (t, *J* = 9.0 Hz, 1H), 5.89 (t, *J* = 9.3 Hz, 0.85H), 5.76 – 5.69 (m, 3.7H), 5.59 – 5.50 (m, 1.85H), 5.37 (dd, *J* = 10.3, 3.4 Hz, 1H), 5.32 (dd, *J* = 10.3, 3.4 Hz, 0.85H), 5.15 (dd, *J* = 7.8, 5.2 Hz, 0.85H), 5.07 (dd, *J* = 10.8, 3.6 Hz, 1H), 4.89 (dd, *J* = 12.8, 8.0 Hz, 1.85H), 4.55 (d, *J* = 2.3 Hz, 1.7H), 4.53 – 4.37 (m, 2H), 4.29 – 4.17 (m, 1.85H), 3.98 (t, *J* = 10.0 Hz, 1.85H), 3.93 – 3.72 (m, 7.4H), 3.67 (s, 3H), 3.62 (s, 2.55H), 2.87 – 2.75 (m, 1H), 2.61 (ddd, *J* = 14.9, 7.9, 2.7 Hz, 0.85H), 2.39 – 2.22 (m, 1.85H), 1.32 (s, 15.3H), 1.31 (s, 18H). ^{13}C NMR (101 MHz, $CDCl_3$) δ 171.0, 170.5, 165.9, 165.7, 165.6, 165.5, 165.4, 165.33, 165.30, 165.26, 165.22, 164.8, 164.7, 151.81, 151.77, 133.44, 133.41, 133.3, 133.2, 130.0, 129.9, 129.8, 129.7, 129.6, 129.5, 129.4, 128.9, 128.7, 128.6, 128.5, 128.4, 128.3, 128.2, 101.2, 101.1, 83.3, 83.2, 83.1, 76.0, 72.1, 71.9, 71.6, 71.3, 70.8, 70.7, 69.9, 69.83, 69.79, 67.5, 62.8, 62.4, 61.1, 54.6, 54.2, 52.4, 52.2, 27.8, 27.7. HRMS (ESI) m/z: $[M + H]^+$ calcd for $C_{75}H_{74}NO_{23}$ 1356.4646, found 1356.4582.

Methyl[3-α-(2,3:5,6-Di-*O*-isopropylidene-α-D-mannofuranosyl)]-(2S/2R)-N,N-di-*tert*-buthoxycarbonyl-alanine (3ea). By following general procedure B (**3ea)** was obtained as a white amorphous solid (131 mg, 0.24 mmol, 99%) in a mixture of two diastereomers in ratio (1:0.3) after flash chromatography (cyclohexane:ethyl acetate 9:1). ^{1}H NMR (400 MHz, $CDCl_3$) δ 5.13 (t, *J* = 6.6 Hz, 1H major diast.), 4.97 (dd, *J* = 10.0, 4.1 Hz, 0.3H), 4.79 (dd, *J* = 6.0, 3.6 Hz, 0.3H), 4.76 (dd, *J* = 6.0, 3.7 Hz, 1H), 4.56 – 4.51 (m, 1.3H), 4.46 – 4.28 (m, 3.6H), 4.14 – 4.02 (m, 1.9H), 3.72 (s, 0.9H), 3.71 (s, 3H), 3.65 (dd, *J* = 8.5, 3.7 Hz, 1H), 2.33 – 2.21 (m, 1.3H), 2.04 – 1.95 (m, 0.3H), 1.87 (ddd, *J* = 14.5, 10.8, 6.7 Hz, 1H), 1.52 (s, 0.9H), 1.494 (s, 18H), 1.485 (s, 5.4H), 1.481 (s, 3H), 1.46 (s, 0.9H), 1.44 (s, 0.9H), 1.42 (s, 0.9H), 1.41 (s, 3H), 1.37 (s, 3H), 1.33 (s, 3H). ^{13}C NMR (101 MHz, $CDCl_3$) δ 170.9, 151.8, 109.2, 85.5, 83.4, 83.2, 82.6, 82.4, 80.8, 80.84, 80.82, 80.7, 80.4, 73.24, 73.18, 67.2, 55.4, 55.3, 52.3, 31.4, 28.0, 27.0, 26.9, 26.1, 25.23, 25.16, 24.8. HRMS (ESI) m/z: $[M + H]^+$ calcd for $C_{26}H_{44}NO_{11}$ 546.2909, found 546.2887.

Methyl[3-α(2,3,4,6-Tetra-O-acetyl-α-glucopyranosyl)]-(2S/2R)-N,N-di-*tert*-buthoxycarbonyl-alanine (3fa).

By following general procedure B (**3fa)** was obtained as a white amorphous solid (114 mg, 0.18 mmol, 78%) in a mixture of two diastereomers in ratio (1:0.3) after flash chromatography (cyclohexane:ethyl acetate 8:2) (d.r. 60:40). ^{1}H NMR (400 MHz, $CDCl_3$) δ 5.38 – 5.26 (m, 1.3H), 5.19 – 4.96 (m, 3.9H), 4.53 – 4.43 (m, 1H), 4.30 – 4.16 (m, 2.3H), 4.16 – 4.09 (m, 1.3H), 3.89 – 3.80 (m, 0.3H), 4.03 (dd, J = 12.3, 2.6 Hz, 0.3H), 3.75 (s, 0.9H), 3.71 (s, 3H), 2.67 – 2.46 (m, 1H), 2.25 – 2.11 (m, 1.6H), 2.10 (s, 0.9H), 2.09 (s, 3H), 2.07 (s, 0.9H), 2.06 (s, 3H), 2.04 (s, 0.9H), 2.02 (s, 3H), 2.01 (s, 3H.), 1.99 (s, 0.9H), 1.49 (s, 18H), 1.47 (s, 5.4H). ^{13}C NMR (101 MHz, $CDCl_3$) δ 170.9, 170.8, 170.7, 170.6, 170.3, 170.1, 169.50, 169.47, 152.2, 152.1, 83.5, 83.3, 71.2, 70.5, 69.8, 69.7, 69.5, 68.9, 68.8, 68.7, 68.5, 62.1, 61.7, 54.7, 54.1, 52.5, 52.3, 28.0, 27.9, 27.0, 25.6, 20.7, 20.6. HRMS (ESI) m/z: $[M + H]^+$ calcd for $C_{28}H_{44}NO_{15}$ 634.2705, found 634.2731.

Methyl[3-α(2,3,4,6-Tetra-O-acetyl-α-mannopyranosyl)]-(2S/2R)-N,N-di-*tert*-buthoxycarbonyl-alanine (3ga).

By following general procedure B (**3ga**) was obtained as a white amorphous solid (136 mg, 0.22 mmol, 90%) in a mixture of two diastereomers in ratio (1:0.7) after flash chromatography (cyclohexane:ethyl acetate 9:1). ^{1}H NMR (400 MHz, $CDCl_3$) δ 5.33 – 5.16 (m, 3.4H), 5.16 – 5.03 (m, 2.7H), 4.37 – 4.17 (m, 2.7H), 4.17 – 4.10 (m, 1.7H), 3.98 – 3.89 (m, 1.7H), 3.89 – 3.82 (m, 0.7H), 3.73 (s, 3H), 3.71 (s, 2.1H), 3.54 – 3.39 (m, 1H), 3.18 – 3.06 (m, 0.7H), 2.73 – 2.61 (m, 1H), 2.52 (ddd, *J* = 14.7, 8.4, 3.5 Hz, 0.7H), 2.12 (s, 2.1H), 2.10 (s, 3H), 2.09 (s, 2.1H), 2.07 (s, 3H), 2.06 (s, 3H), 2.04 (s, 2,1H), 2.02 (s, 3H), 2.01 (s, 2.1), 1.49 (s, 12.6H), 1.48 (s, 18H). ^{13}C NMR (101 MHz, $CDCl_3$) δ 171.0, 170.8, 170.7, 170.6, 170.11, 170.06, 169.8, 169.6, 152.1, 152.0, 83.6, 83.5, 73.2, 71.3, 70.5, 70.3, 69.0, 68.8, 67.0, 66.6, 66.1, 62.4, 62.1, 54.4, 54.2, 54.1, 52.5, 52.3, 47.9, 42.3, 30.4, 29.5, 27.9, 20.9, 20.8, 20.71, 20.68, 19.2, 18.6, 17.4. HRMS (ESI) m/z: $[M + H]^+$ calcd for $C_{28}H_{44}NO_{15}$ 634.2705, found 634.2677.

Methyl[3-α(2,3,4,6-Tetra-O-acetyl-α-galactopyranosyl)]-(2S/2R)-N,N-di-*tert*-buthoxycarbonyl-alanine (3ha).

By following general procedure B (**3ha**) was obtained as a white amorphous solid (120 mg, 0.19 mmol, 81%) in a mixture of two diastereomers in ratio (1:0.7) after flash chromatography (cyclohexane:ethyl acetate 9:1). ^{1}H NMR (400 MHz, Chloroform-*d*) δ 5.44 – 5.38 (m, 1.7H), 5.38 – 5.29 (m, 1.7H), 5.19 (ddd, *J* = 16.6, 10.0, 3.3 Hz, 1.7H), 5.09 (t, *J* = 6.6 Hz, 0.7H), 5.02 (dd, *J* = 10.6, 3.8 Hz, 1H), 4.55 – 4.45 (m, 1H), 4.28 – 4.19 (m, 0.7H), 4.16 – 3.99 (m, 5.1H), 3.74 (s, 2.1H), 3.71 (s, 3H), 2.57 (ddd, *J* = 15.9, 12.5, 3.9 Hz, 0.7H), 2.46 (ddd, *J* = 15.0, 7.5, 3.1 Hz, 1H), 2.20 – 2.12 (m, 1.7H), 2.12 (s, 2.1H), 2.11 (s, 3H), 2.08 (s, 2.1H), 2.03 (s, 5.1H), 2.012 (s, 2.1H), 2.006 (s, 3H), 2.001 (s, 3H), 1.49 (s, 12.6H), 1.48 (s, 18H). ^{13}C NMR (101 MHz, $CDCl_3$) δ 171.2, 170.7, 170.5, 170.4, 170.2, 170.1, 170.0, 169.7, 152.2, 152.0, 83.5, 83.3, 71.0, 69.5, 68.1, 68.0, 67.9, 67.8, 67.6, 67.5, 61.3, 60.9, 55.0, 54.4, 52.5, 52.3, 28.0, 27.9, 27.0, 25.7, 20.8, 20.69, 20.67, 20.64. HRMS (ESI) m/z: $[M + H]^+$ calcd for $C_{28}H_{44}NO_{15}$ 634.2705, found 634.2679.

Ethyl[3-α(2,3,4,6-Tetra-O-benzoyl-α-glucopyranosyl)]-(2S/2R)-N-acetyl-alanine (3af)

By following general procedure B (**3af**) was obtained as a white amorphous solid (140 mg, 0.19 mmol, 80%) in a mixture of two diastereomers in ratio (1:0.5) after flash chromatography (cyclohexane:ethyl acetate 8:2). ^{1}H NMR (300 MHz, $CDCl_3$) δ 8.09 (d, *J* = 7.1 Hz, 3H), 7.99 (d, *J* = 8.4 Hz, 3H), 7.96 – 7.87 (m, 6H), 7.62 – 7.27 (m, 18H), 6.38 (dd, *J* = 19.9, 7.5 Hz, 1.5H), 5.88 (t, *J* = 7.9 Hz, 1.5H), 5.64 – 5.55 (m, 1.5H), 5.48 – 5.38 (m, 1.5H), 4.87-4.67 (m, 2.5H), 4.64 – 4.46 (m, 2.5H), 4.46 – 4.29 (m, 0.5H), 4.23 – 4.00 (m, 2H), 2.66 – 2.43 (m, 1.5H), 2.33 – 2.07 (m, 1.5H), 1.95 (s, 3H), 1.94 (s, 1.5H), 1.22 (t, J = 7.1 Hz, 3H), 1.13 (t, J = 7.1 Hz, 1.5H). ^{13}C NMR (101 MHz, $CDCl_3$) δ 171.7, 171.6, 170.1, 169.6, 166.4, 166.3, 165.4, 165.2, 133.7, 133.55, 133.46, 133.4, 133.2, 133.1, 129.9, 129.8, 129.7, 128.9, 128.8, 128.7, 128.6, 128.5, 128.44, 128.40, 128.3, 70.8, 70.6, 70.5, 70.3, 69.71, 69.67, 68.9, 68.7, 68.6, 62.6, 62.3, 61.9, 61.8, 49.9, 49.3, 29.5, 28.3, 23.1, 23.0, 14.03, 13.96. HRMS (ESI) m/z: $[M + H]^+$ calcd for $C_{41}H_{40}NO_{12}$ 738.7655, found 738.7619.

Methyl[3-α(2,3,4,6-Tetra-O-benzoyl-β-D-glucopyranosyl-(1→4)-2,3,6-tri-O-benzoyl-α-D-glucopyranosyl]-(2S/2R)-N,N-di-*tert*-buthoxycarbonyl-alanine (3ia).

By following general procedure B (**3ia**) was obtained as a white amorphous solid (325 mg, 0.24 mmol, quant.) in a mixture of two diastereomers in ratio (1:0.7) after flash chromatography (cyclohexane:ethyl acetate 8:2). ^{1}H NMR (300 MHz, $CDCl_3$) δ 8.07 – 7.86 (m, 17H), 7.81 – 7.69 (m, 6.8H), 7.60 – 7.26 (m, 28.9H), 7.26 – 7.18 (m, 6.8H), 6.01 – 5.82 (m, 1.7H), 5.79 – 5.64 (m, 1.7H), 5.55 – 5.42 (m, 3.4H), 5.42 – 5.25 (m, 1.7H), 5.18 – 5.03 (m, 1.7H), 4.97 (dd, *J* = 12.7, 8.0 Hz, 1.7H), 4.79 – 4.69 (m, 0.7H), 4.61 – 4.30 (m, 4.4H), 4.29 – 3.90 (m, 5.1H), 3.91 – 3.69 (m, 3.4H), 3.67 (s, 2.1H), 3.60 (s, 3H), 2.87 – 2.73 (m, 0.7H), 2.66 – 2.50 (m, 1H), 2.38 – 2.20 (m, 1.7H), 1.32 (s, 12.6H), 1.30 (s, 18H). ^{13}C NMR (101 MHz, $CDCl_3$) δ 171.0, 170.5, 165.8, 165.71, 165.68, 165.66, 165.62, 165.59, 165.46, 165.38, 165.30, 165.0, 164.8, 164.7, 151.8, 151.7, 133.4, 133.3, 133.25, 133.21, 133.14, 133.09, 129.9, 129.8, 129.73, 129.69, 129.61, 129.60, 129.1, 128.9, 128.7, 128.6, 128.4, 128.34, 128.29, 128.2, 101.2, 101.1, 83.3, 83.2, 73.1, 73.0, 72.33, 72.29, 71.9, 71.2, 70.8, 70.5, 70.4, 70.1, 69.9, 69.7, 69.45, 69.38, 62.9, 62.7, 62.6, 62.4, 54.5, 54.2, 52.4, 52.2, 27.8, 27.7. HRMS (ESI) m/z: $[M + H]^+$ calcd for $C_{75}H_{74}NO_{23}$ 1356.4646, found 1356.4710.

Scale up procedure for the synthesis of compound 3aa (2 mmol scale).

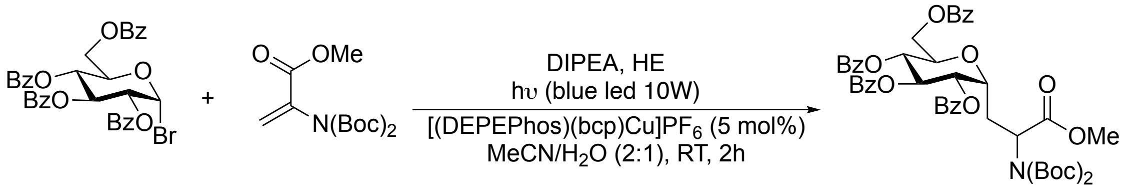

2,3,4,6-Tetra-O-benzoyl-α-D-glucopyranosyl bromide (**1a**) (1.30 g, 2 mmol), Methyl-2-(di(tert-butoxycarbonyl)amino)but-2-enoate (**2a**) (1.20 g, 4 mmol), Hantzsch ester (**HE-1**) (1.01 g, 4 mmol), DIPEA (1.56 mL, 9 mmol) and [(DPEPhos)(bcp)Cu]PF_6 (5 mol%, 0.1 mmol, 110 mg) were inserted in a sealed Schlenk flask. The internal atmosphere was saturated with argon after 3 cycles of vacuum/argon. Then 15 mL of MeCN/H_2O (2:1) (deaerated with freeze and pump) were added and the reaction was placed at 5 cm from the light source (blue led 10 W) and stirred and irradiated for 2 hours at room temperature. After this time the mixture was extracted with DCM (3x40 mL), dried with anhydrous Na_2SO_4, filtered, concentrated by rotary evaporator and purified with flash column chromatography (cyclohexane:EtOAc 8:2) to give Methyl[3-α-(2,3,4,6-tetra-*O*-Benzoyl-α-D-glucopyranosyl)]-(2S/2R)-N,N-di-tert-buthoxycarbonyl-alanine (**3aa**) in a 90% yield (1.58 g, 1.8 mmol).

NMR INVESTIGATION

Interaction between sugar and photocatalyst

In order to investigate the deactivation of Cu complex by interaction with protected sugar we performed different NMR tests in different deuterated solvents.

The three following tests were performed as described: The ^{1}H-NMR spectrum of the sugar was recorded (Step 1) and ^{1}H-NMR spectrum of the Cu-photocatalyst as well (Step 2); subsequently, the content of both NMR tubes was mixed together, and the resulting ^{1}H-NMR sample was recorded (Step 3). After this we moved to irradiate the sample under blue led light (10 W) for 1 hour and then ^{1}H-NMR was recorded again (Step 4).

TEST N°1.

Interaction between 2,3,4,6-Tetra-O-benzoyl-α-D-glucopyranosyl bromide (1a) and [DPEPhos(bcp)Cu]PF_6 in DCM-d_2.

Step 1:

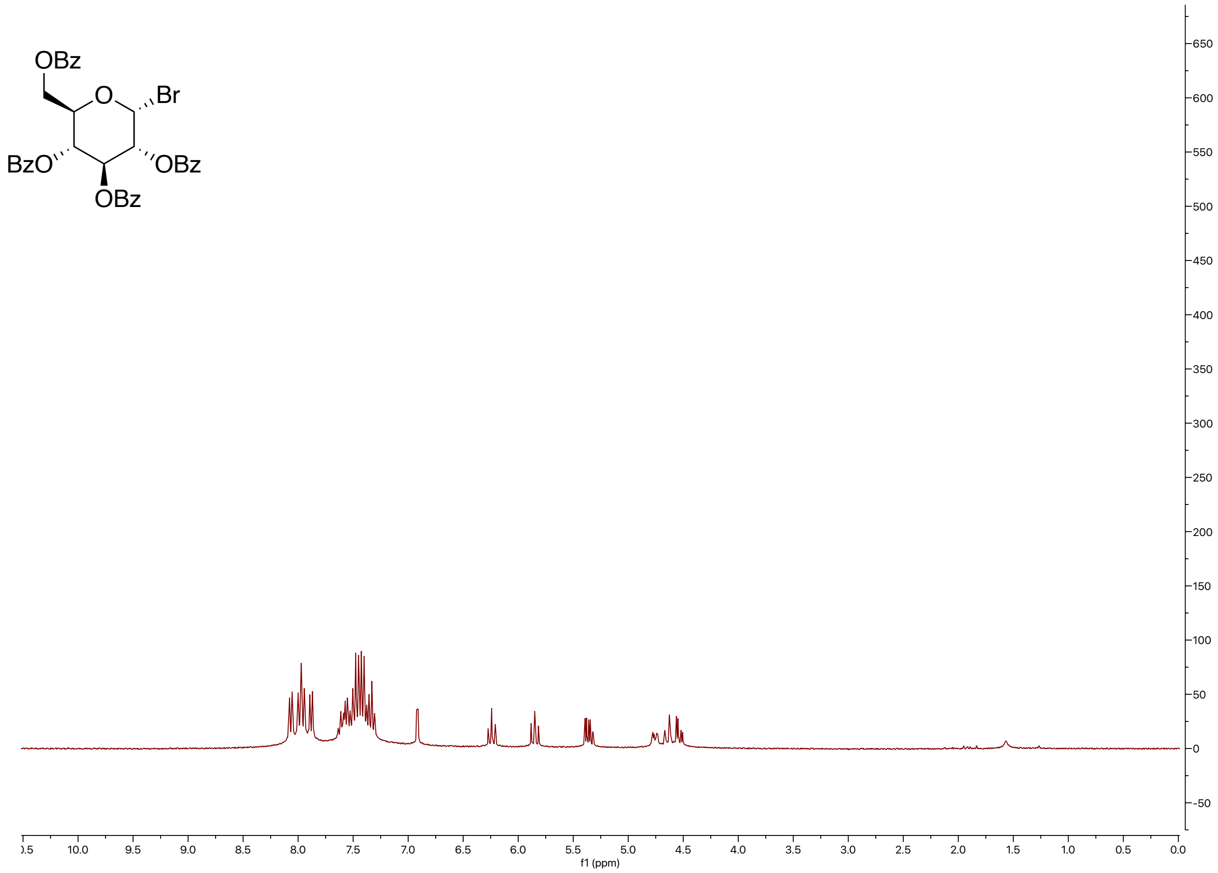

*Figure S2: ^{1}H-NMR of 2,3,4,6-Tetra-O-benzoyl-α-D-glucopyranosyl bromide (**1a**) in DCM-d_2*

Step 2:

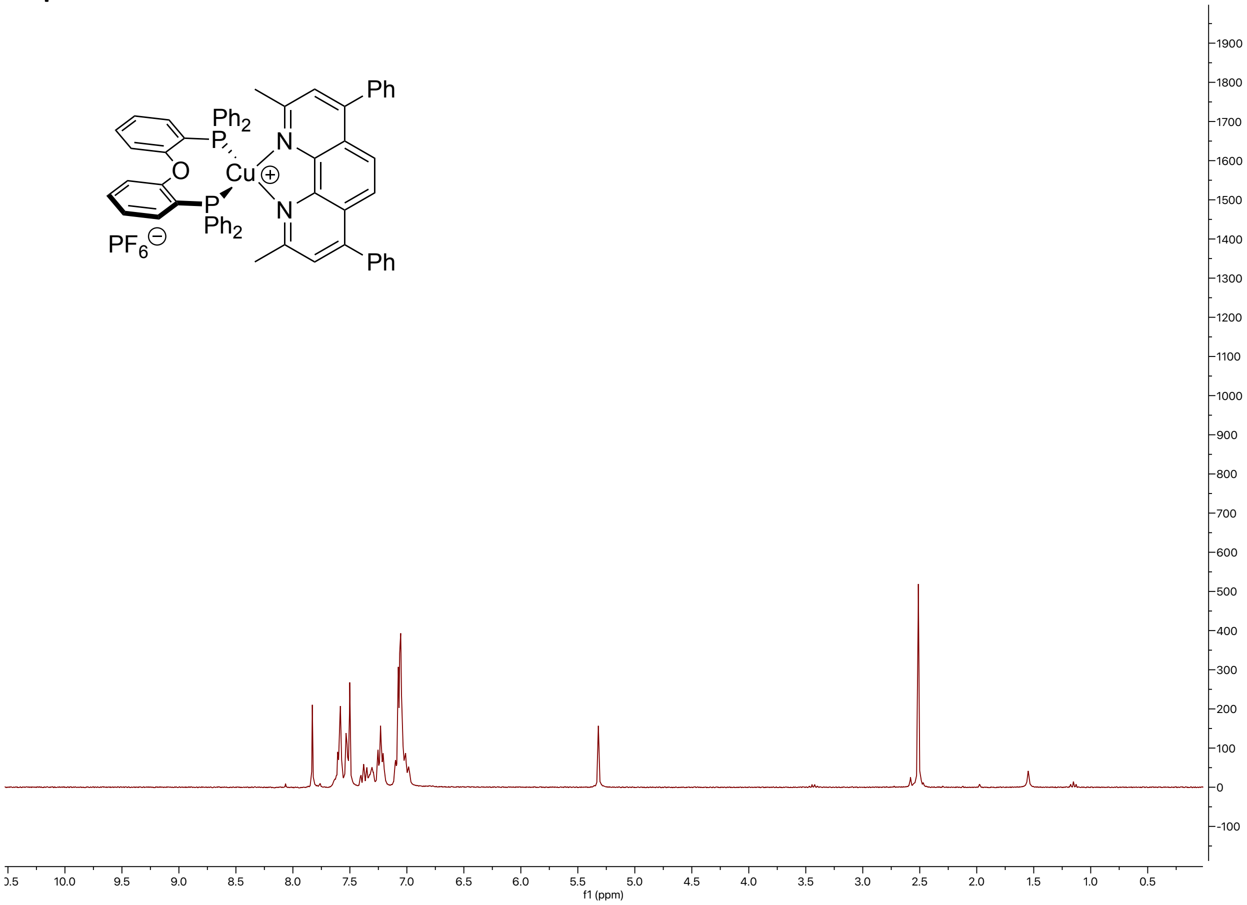

Figure S3: ^{1}H-NMR of [DPEPhos(bcp)Cu]PF_6 in DCM-d_2

Step 3:

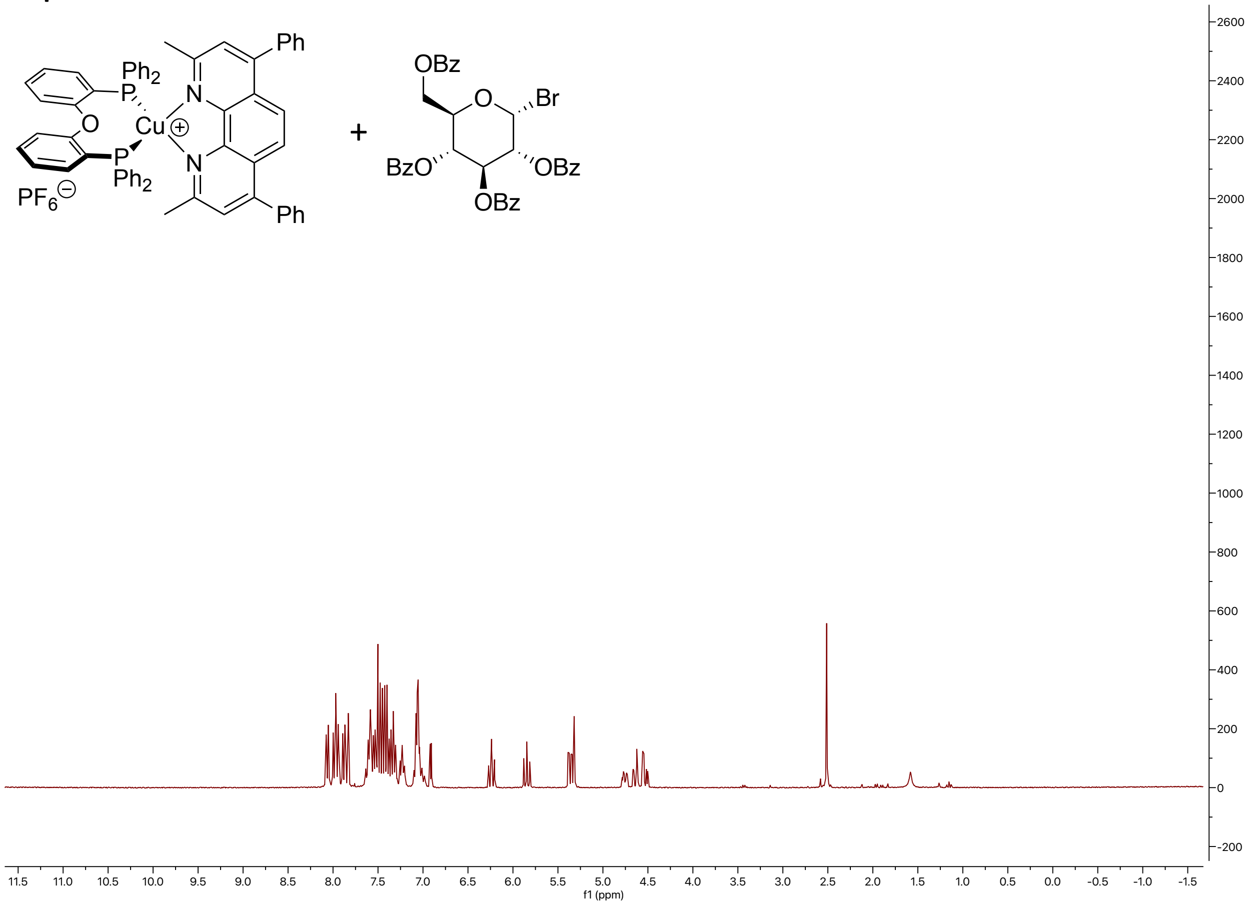

*Figure S4: ^{1}H-NMR of 2,3,4,6-Tetra-O-benzoyl-α-D-glucopyranosyl bromide (**1a**) mixed with [DPEPhos(bcp)Cu]PF_6 in DCM-d_2 before irradiation.*

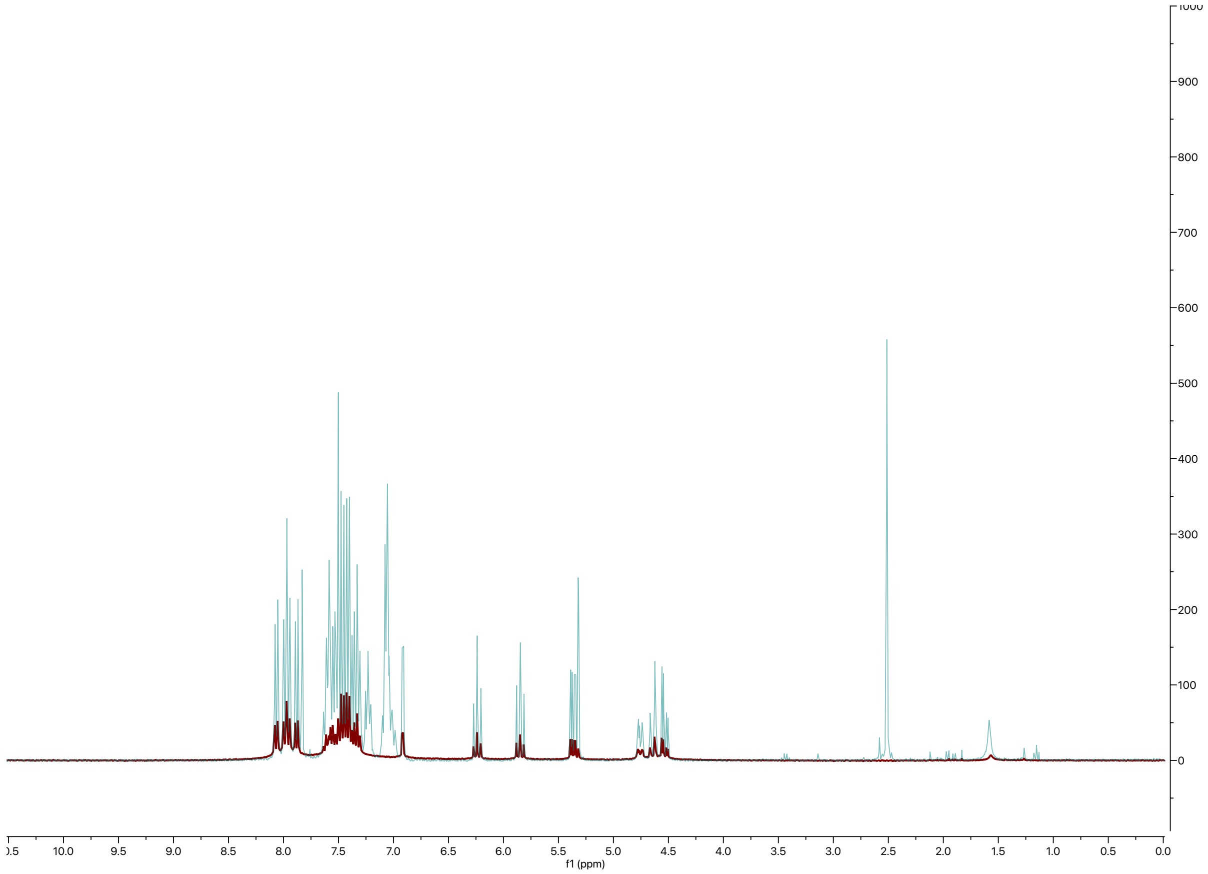

Figure S5: Overlapped ^{1}H-NMR spectra of 2,3,4,6-Tetra-O-benzoyl-α-D-glucopyranosyl bromide (1a) (red line) mixed with [DPEPhos(bcp)Cu]PF_6 in DCM-d_2 before irradiation (blue line).

As can be seen from the spectra reported above there is no interaction in DCM-d_2 between the sugar and the catalyst. In fact, the spectrum remains the same to the previous recorded of the two separated species even after 1h of irradiation.

Step 4:

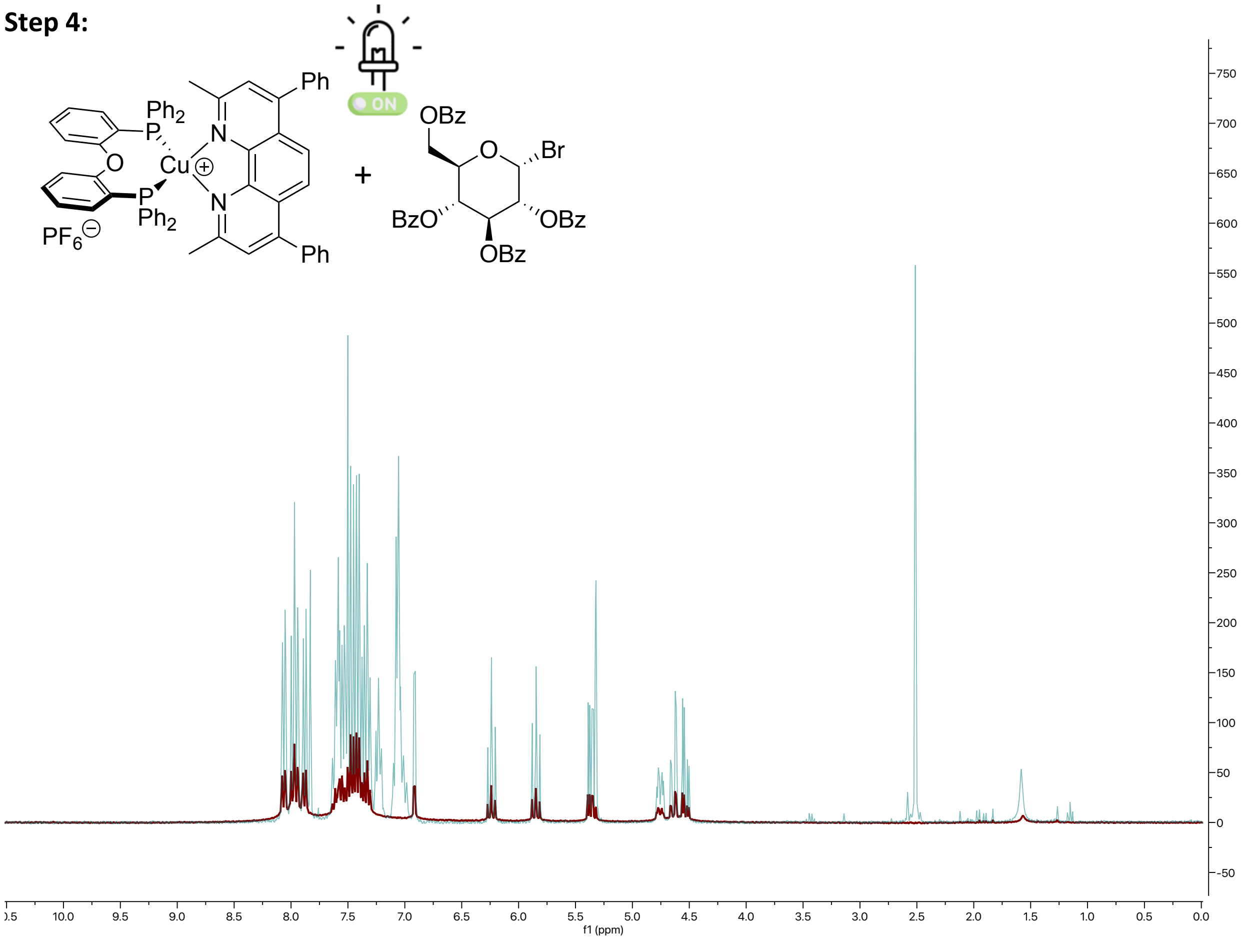

*Figure S6: Overlapped ^{1}H-NMR spectra of 2,3,4,6-Tetra-O-benzoyl-α-D-glucopyranosyl bromide (**1a**) (red line) mixed with [DPEPhos(bcp)Cu]PF_6 in DCM-d_2 after 1h of irradiation under blue led light (10 W) (blue line).*

TEST N°2.

Interaction between 2,3,4,6-Tetra-O-benzoyl-α-D-glucopyranosyl bromide (1a) and [DPEPhos(bcp)Cu]PF_6 in MeCN-d_3/D_2O.

Step 1:

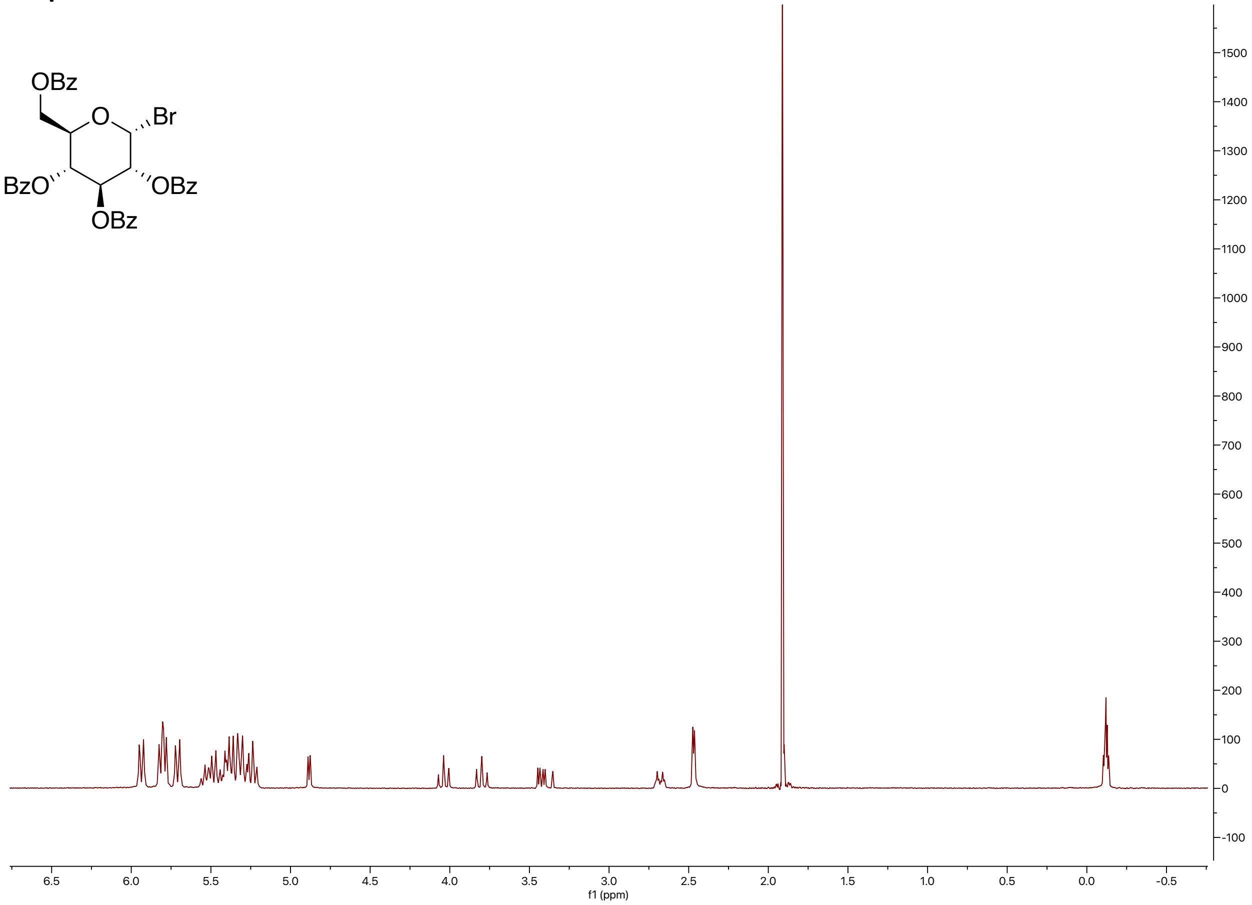

Figure S7: ^{1}H-NMR of 2,3,4,6-Tetra-O-benzoyl-α-D-glucopyranosyl bromide (1a) in MeCN-D_3/D_2O.

Step 2:

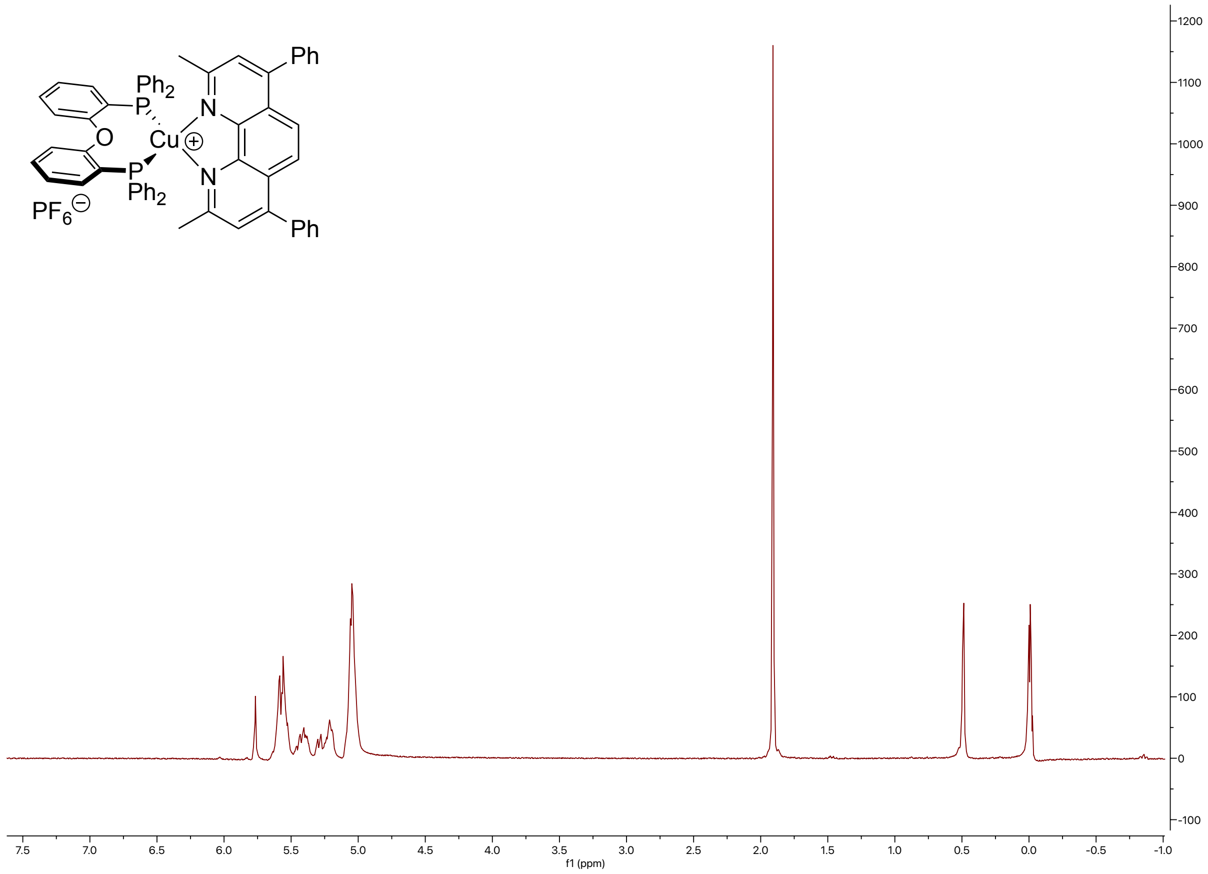

Figure S8: ^{1}H-NMR of [DPEPhos(bcp)Cu]PF$_6$ in MeCN-D$_3$/D$_2$O.

Step 3:

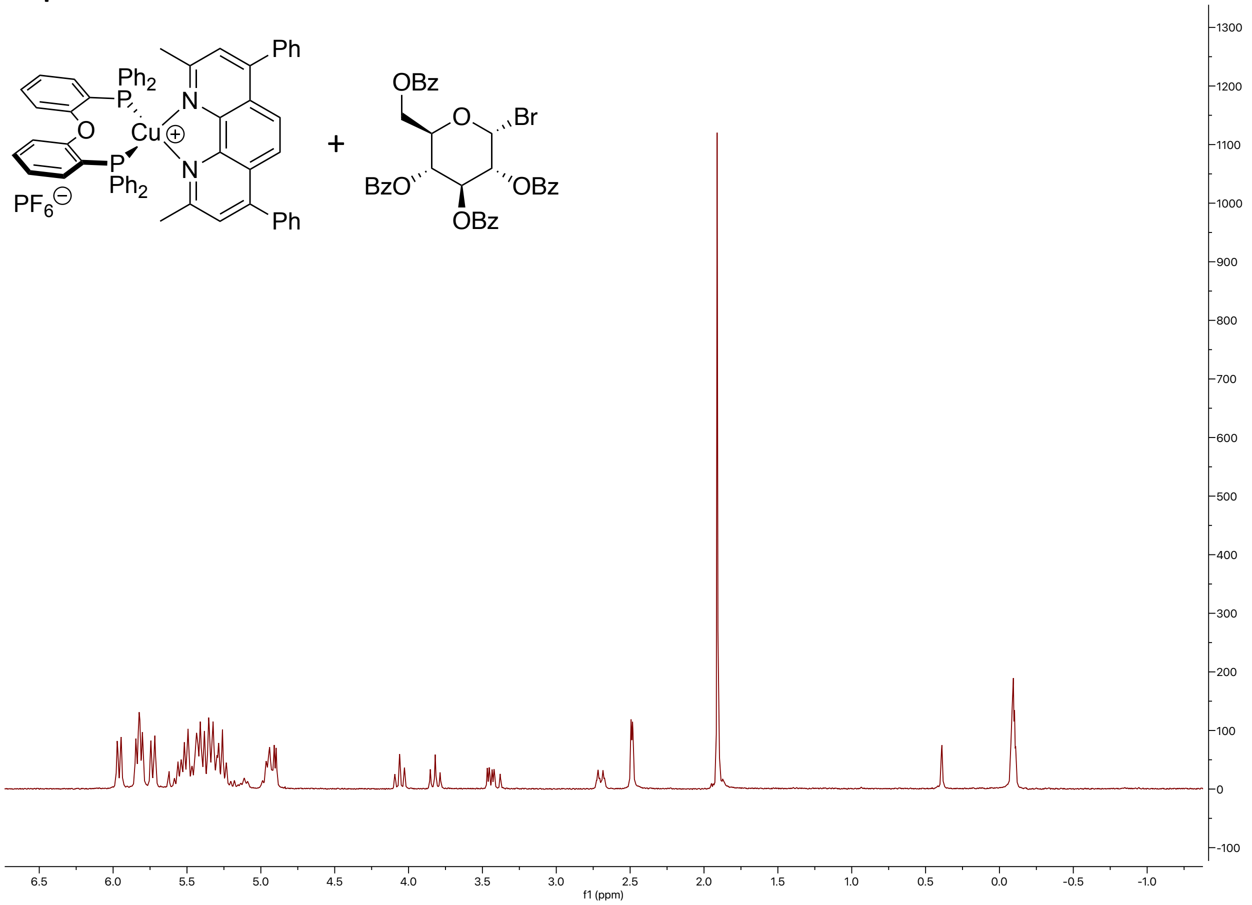

*Figure S9: ^{1}H-NMR of 2,3,4,6-Tetra-O-benzoyl-α-D-glucopyranosyl bromide (**1a**) mixed with [DPEPhos(bcp)Cu]PF_6 before irradiation in MeCN-d_3/D_2O.*

Step 4:

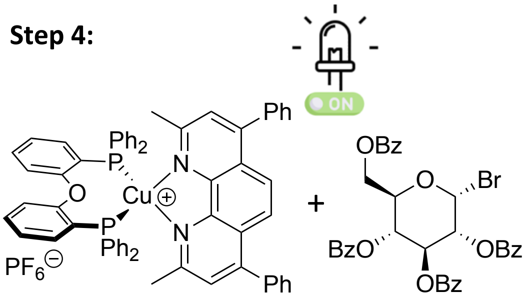

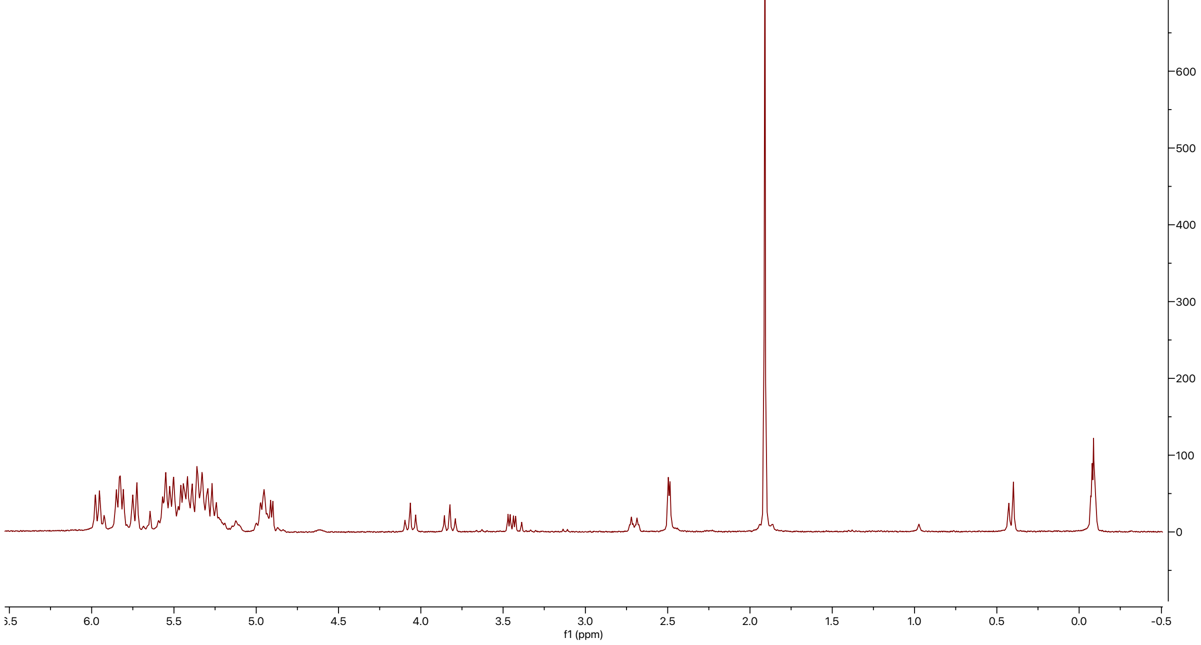

*Figure S10: ^{1}H-NMR spectra of 2,3,4,6-Tetra-O-benzoyl-α-D-glucopyranosyl bromide (**1a**) mixed with [DPEPhos(bcp)Cu]PF_6 in MeCN-d_3/D_2O after 1h of irradiation under blue led light (10 W).*

Even in this case (*MeCN-d_3/D_2O*) there is not interaction between 2,3,4,6-Tetra-O-benzoyl-α-D-glucopyranosyl bromide (**1a**) and the [DPEPhos(bcp)Cu]PF_6 in MeCN-d_3/D_2O. In fact, the spectrum remains the same before and after irradiation.

Test N°3:

<u>Interaction between</u> 2,3,4,6-Tetra-O-acetyl-α-glucopyranosyl bromide (1f) <u>and [DPEPhos(bcp)Cu]PF6 in MeCN-d3/D2O.</u>

Step 1:

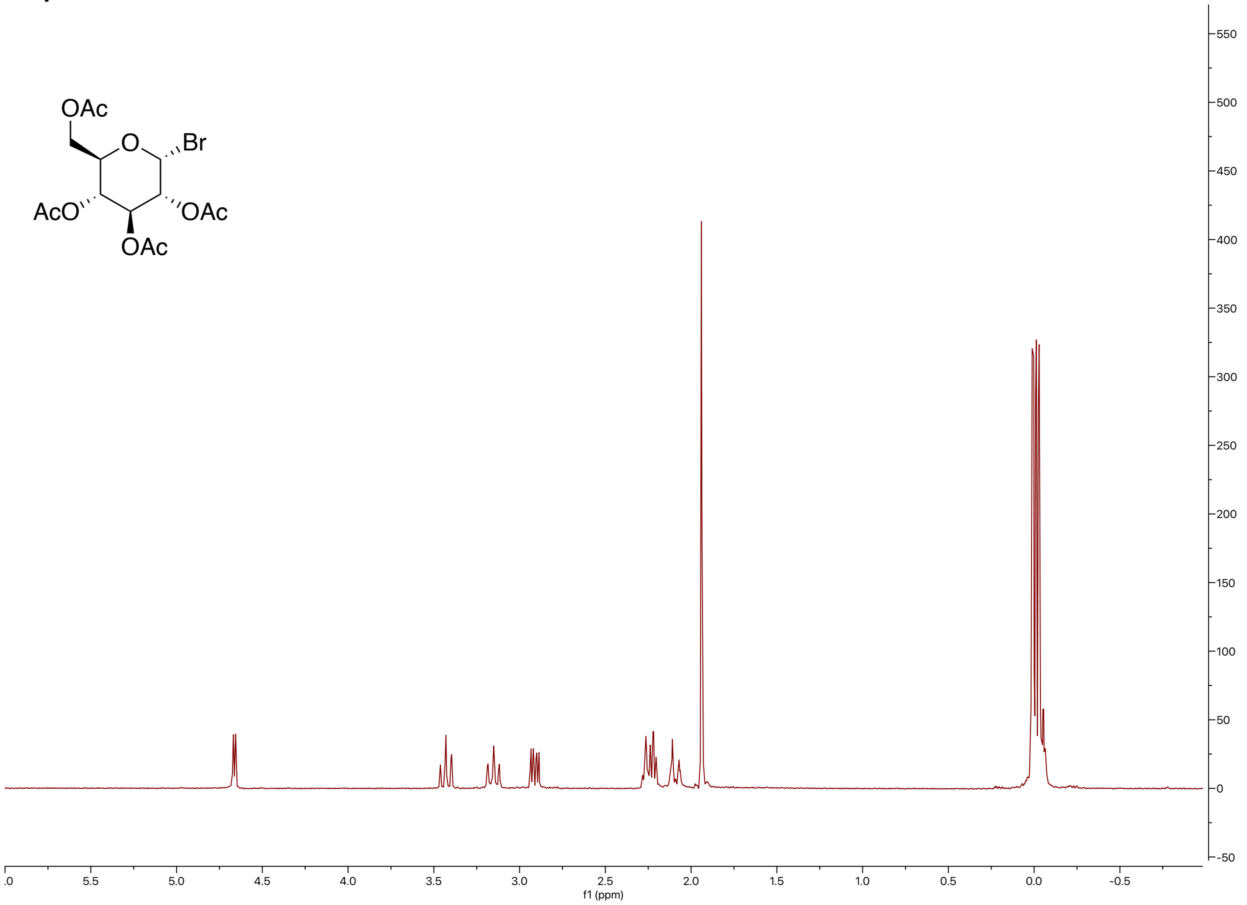

*Figure S11: ^{1}H-NMR of 2,3,4,6-Tetra-O-acetyl-α-glucopyranosyl bromide (**1f**) in MeCN-D_3/D_2O*

Step 2:

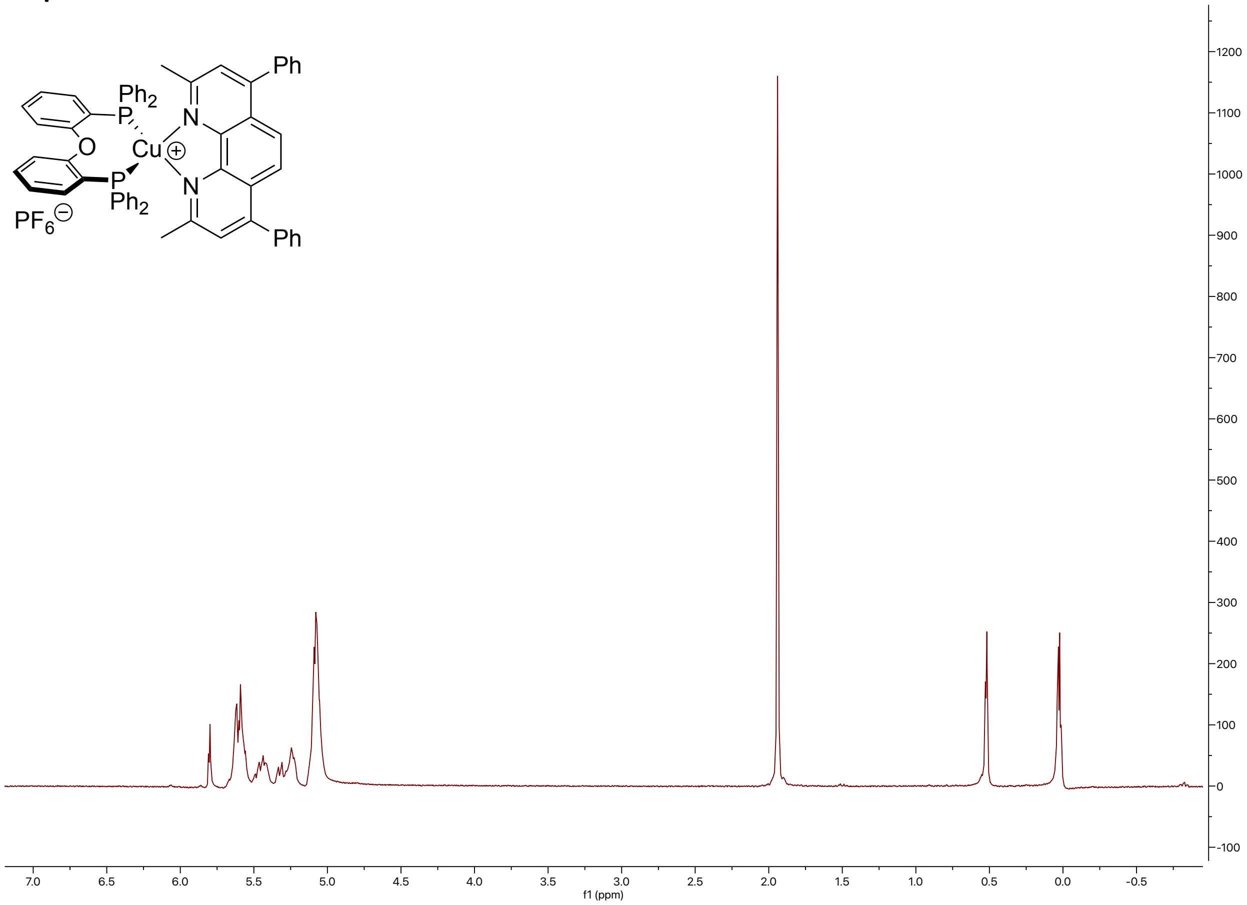

Figure S12: ^{1}H-NMR of [DPEPhos(bcp)Cu]PF_6 in MeCN-d_3/D_2O.

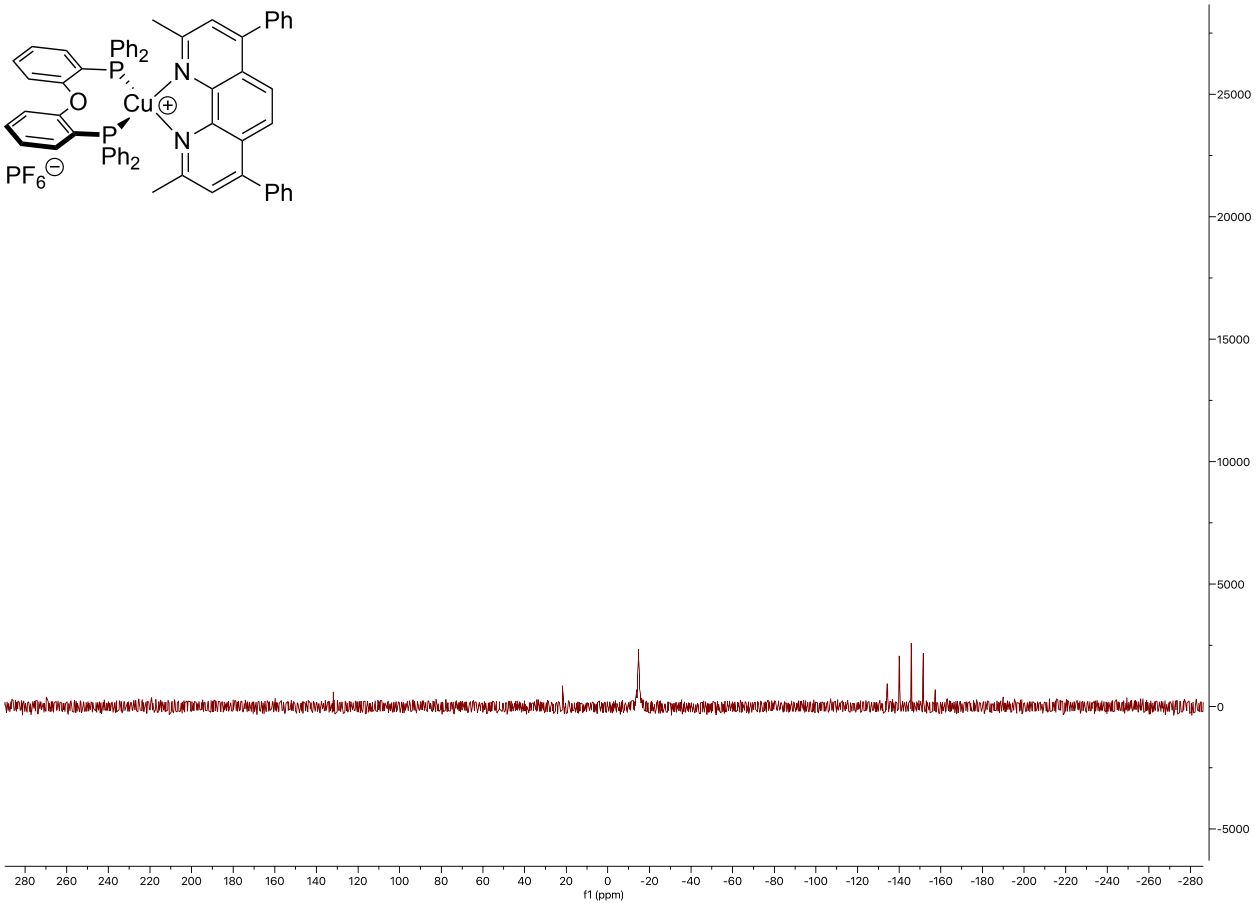

Figure S13: ^{31}P spectra of [DPEPhos(bcp)Cu]PF_6 in MeCN-d_3/D_2O.

Step 3:

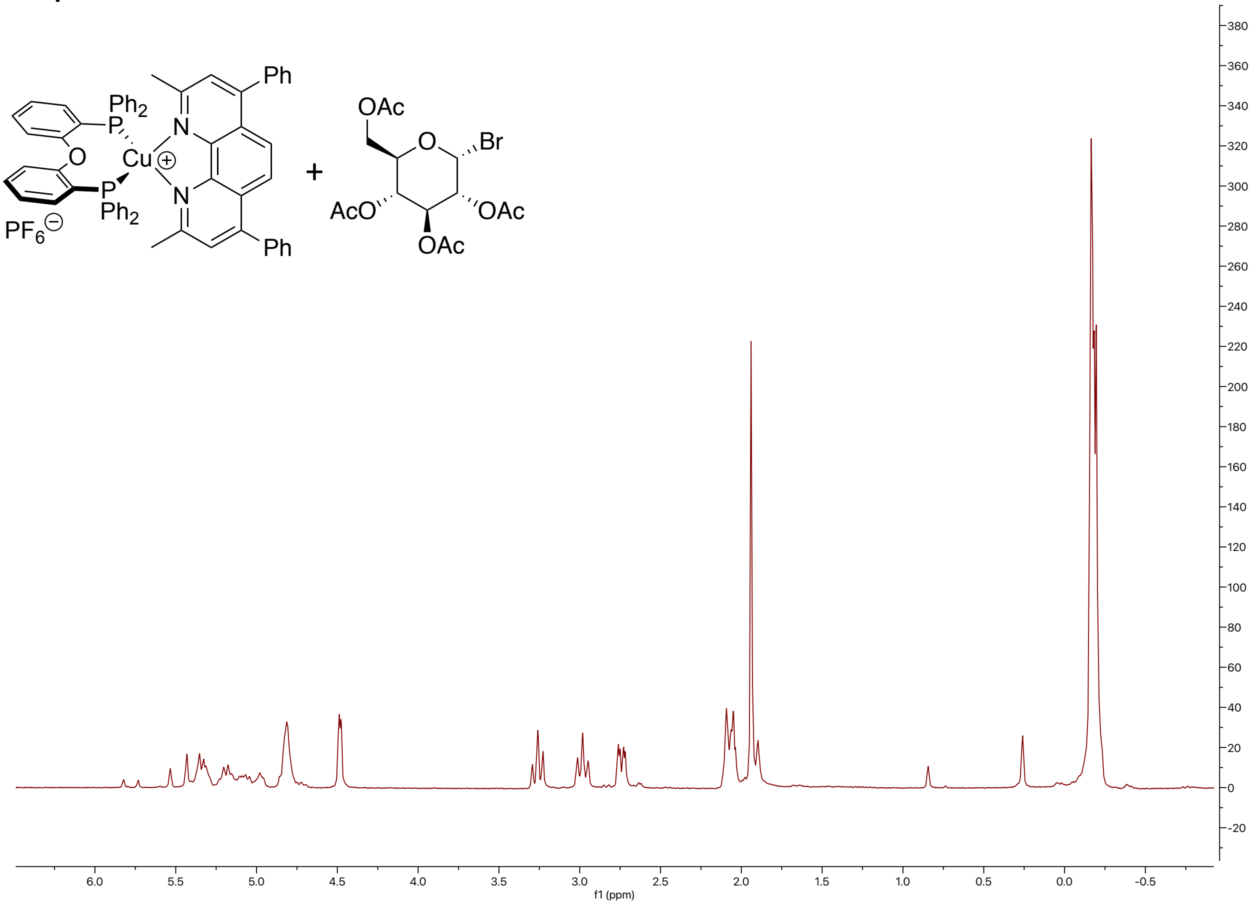

*Figure S14: ^{1}H-NMR of 2,3,4,6-Tetra-O-acetyl-α-glucopyranosyl bromide (**1f**) mixed with [DPEPhos(bcp)Cu]PF_6 before irradiation.*

Step 4:

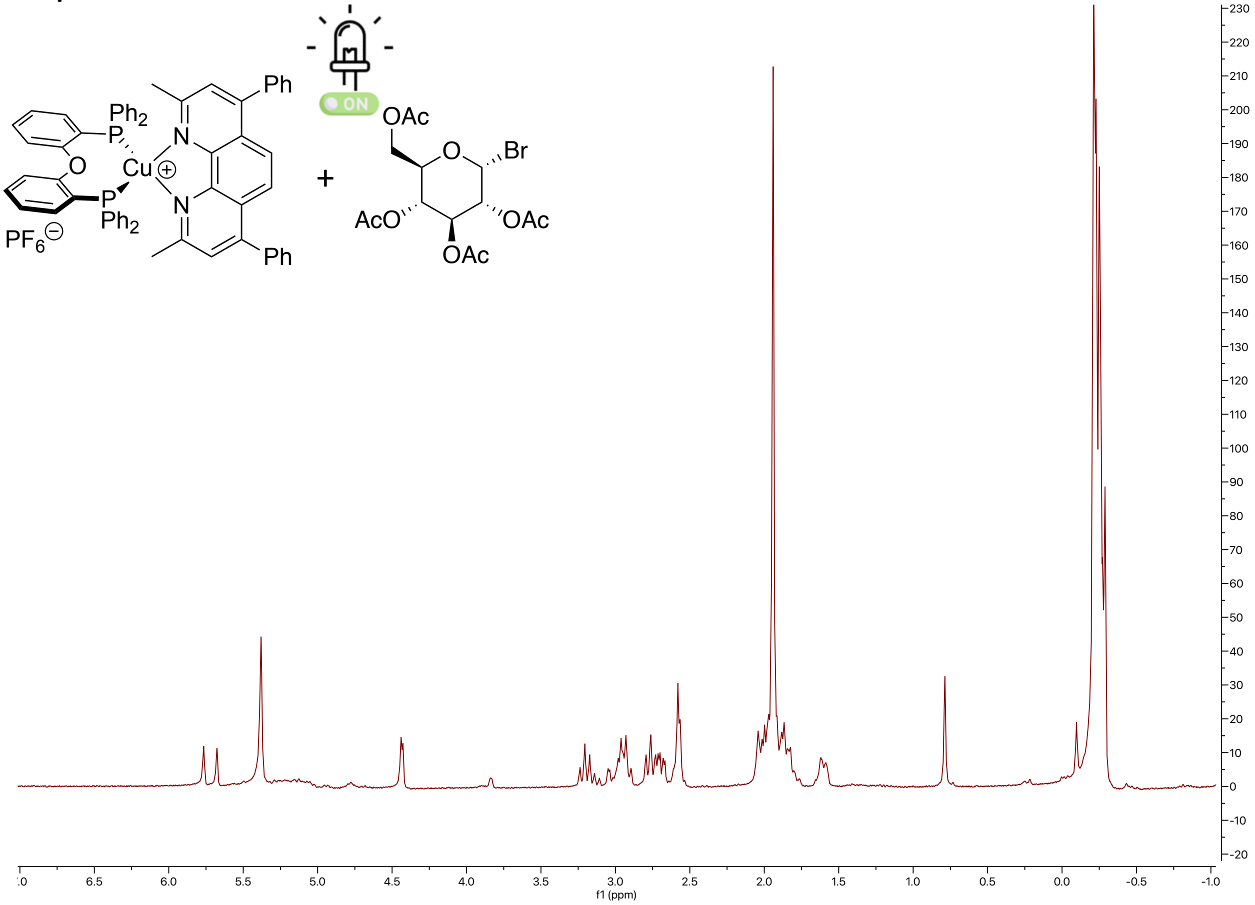

*Figure S14: ^{1}H-NMR of 2,3,4,6-Tetra-O-acetyl-α-glucopyranosyl bromide (**1b**) mixed with [DPEPhos(bcp)Cu]PF$_6$ after 1h irradiation under blue led light (10 W).*

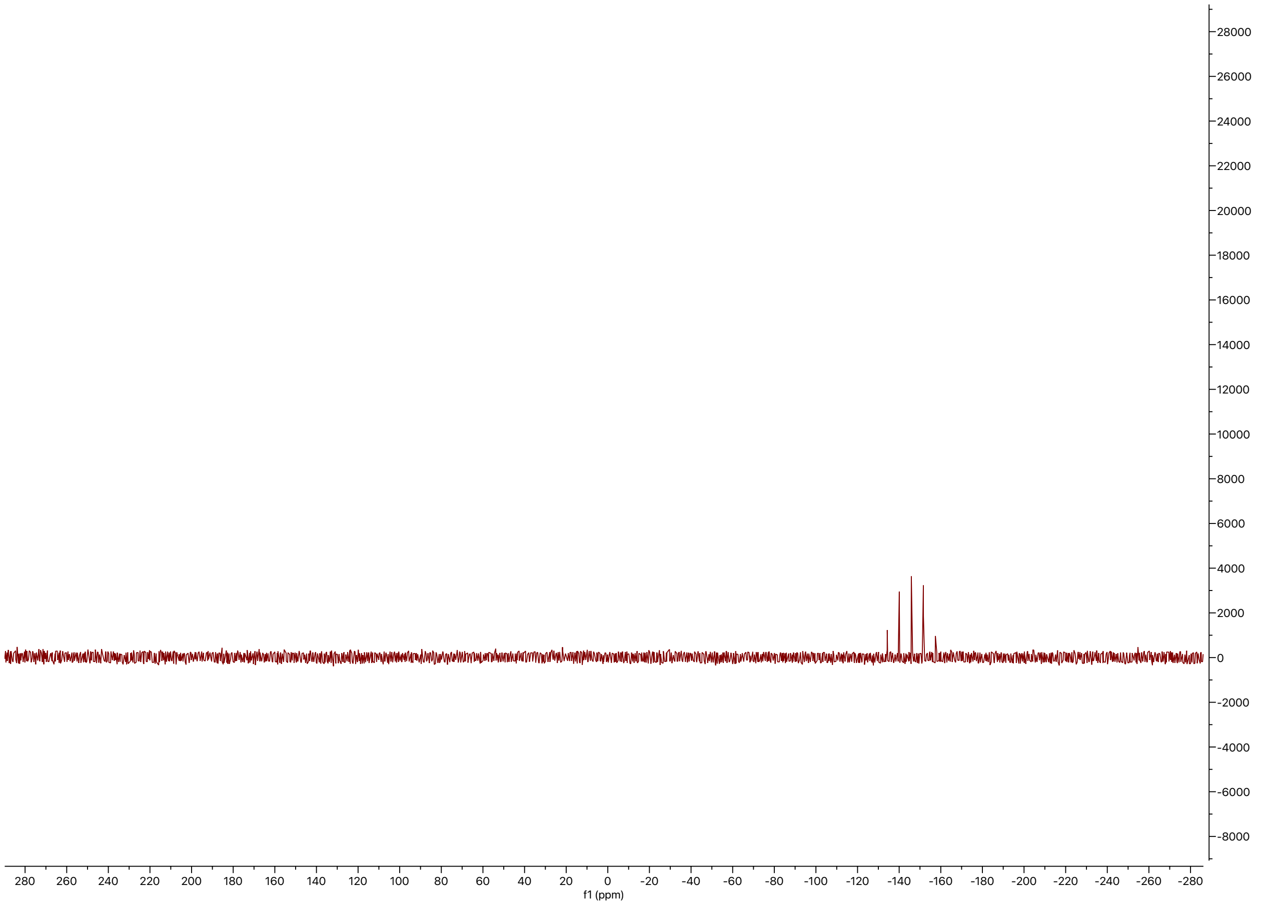

*Figure S15: ³¹P spectrum of 2,3,4,6-Tetra-O-acetyl-α-glucopyranosyl bromide (**1b**) mixed with [DPEPhos(bcp)Cu]PF_6 after 1h irradiation under blue led light (10 W).*

In this case we observed a precipitate in the NMR tube after the irradiation (Figure S16) which is the bis[(2-diphenylphosphino)phenyl] ether as it can be seen from the NMR spectra below. In ^{31}P-NMR spectra is also disappeared the signal of the phosphine ligand of the photocatalyst which probably is the precipitate; however, the ^{31}P NMR spectrum shows that the Cu complex remains in solution due to the signal of the counteranion (PF_6^-). in the tube. There are also important variations in the ^{1}H spectra, predominantly in the aromatic zone.

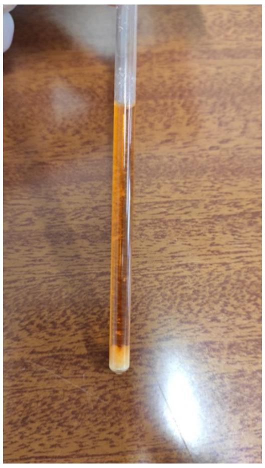

Figure S16: Formation of a precipitate after 1h of irradiation

^{1}H-NMR and ^{13}P-NMR spectra of the PRECIPITATE compared with the ligand used for the preparation of [DPEPhos(bcp)Cu]PF_6

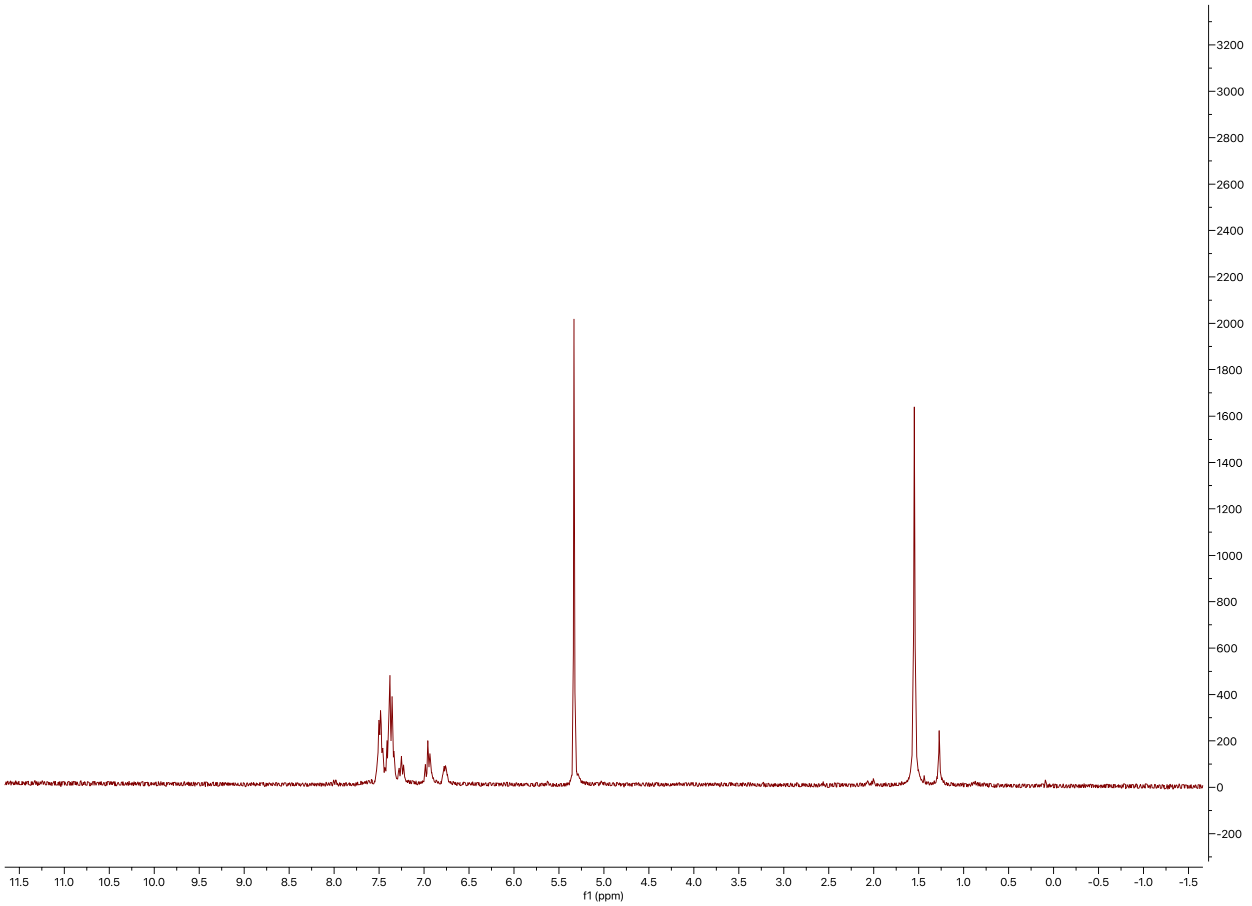

Figure S17: ^{1}H spectrum of the precipitate in DCM-D_2.

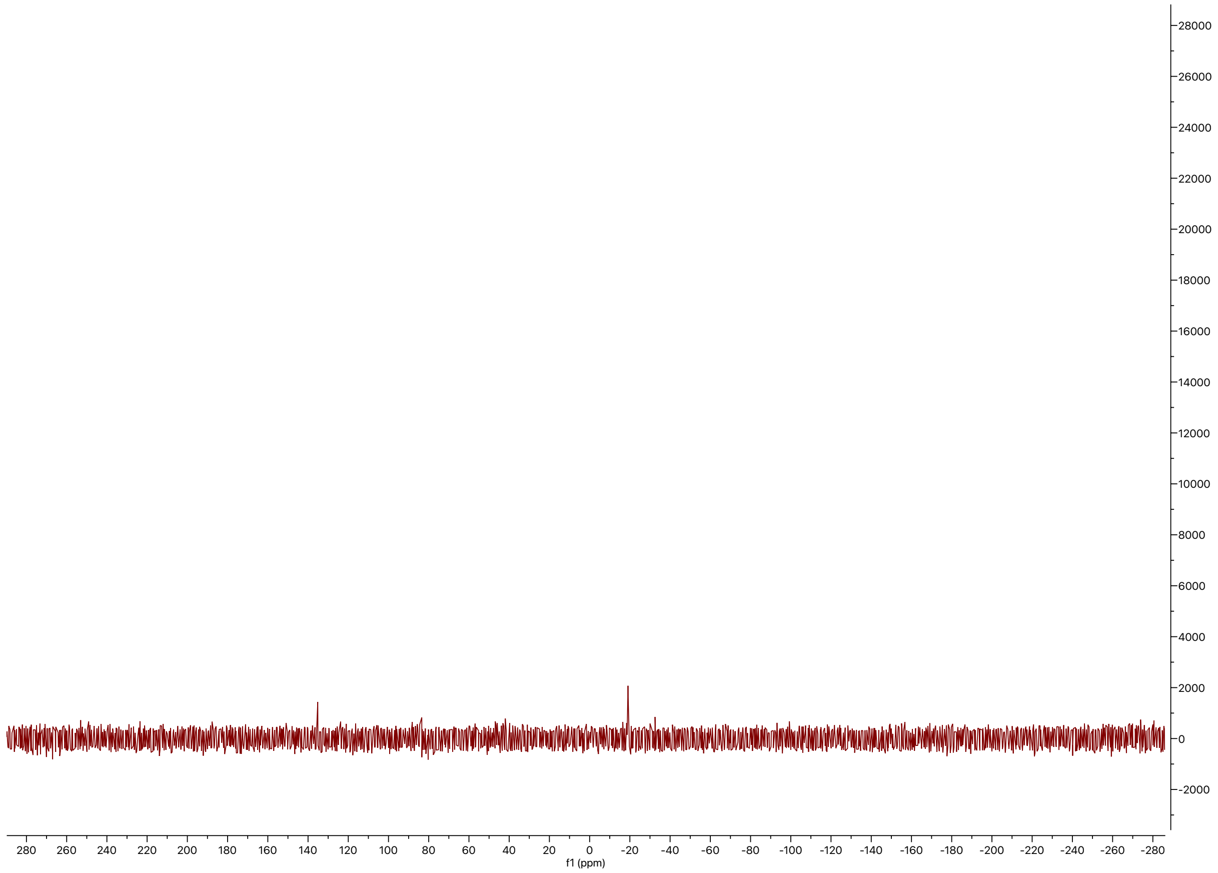

Figure S18: ^{31}P spectrum of the precipitate in DCM-d_2.

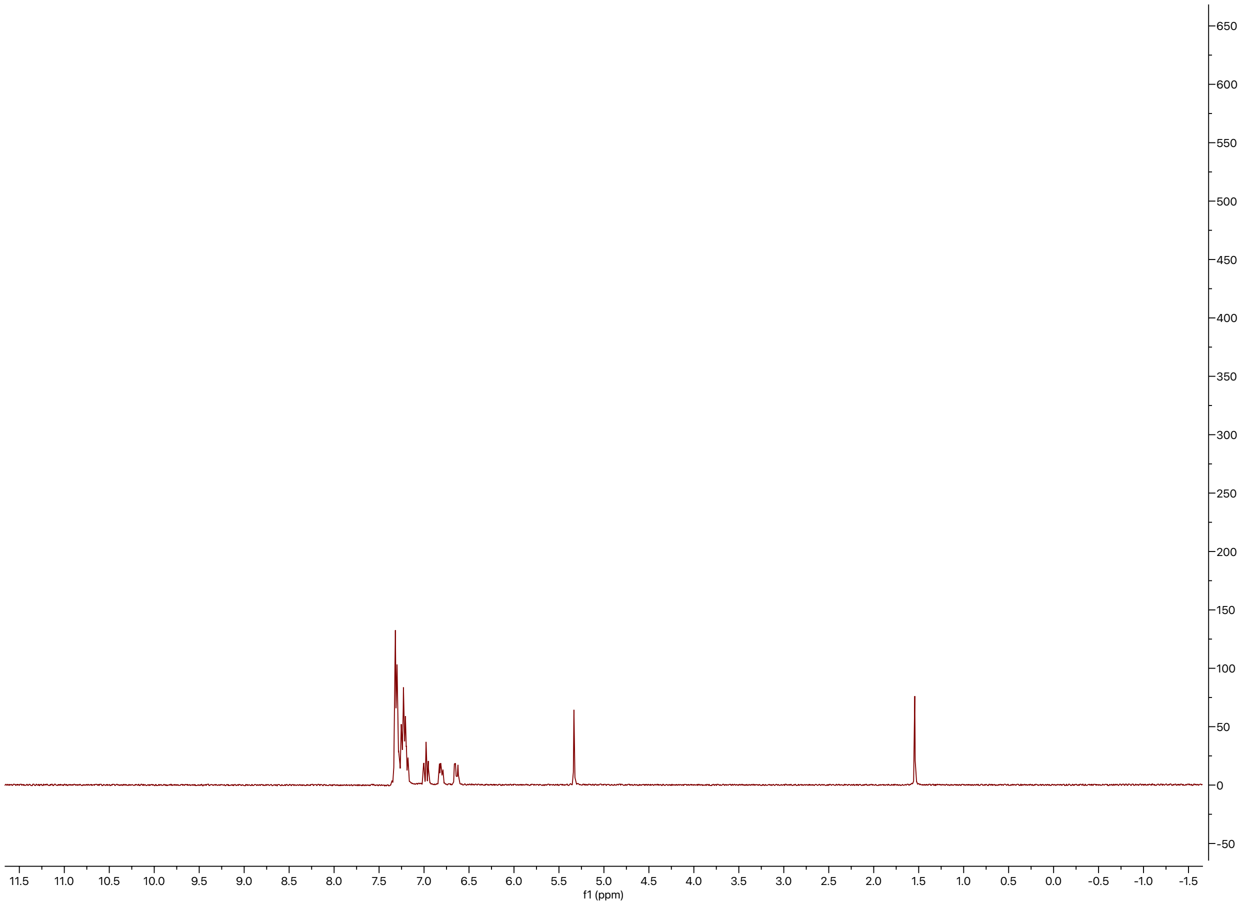

Figure S19: ^{1}H spectrum of bis[(2-diphenylphosphino)phenyl] ether in DCM-d_2.

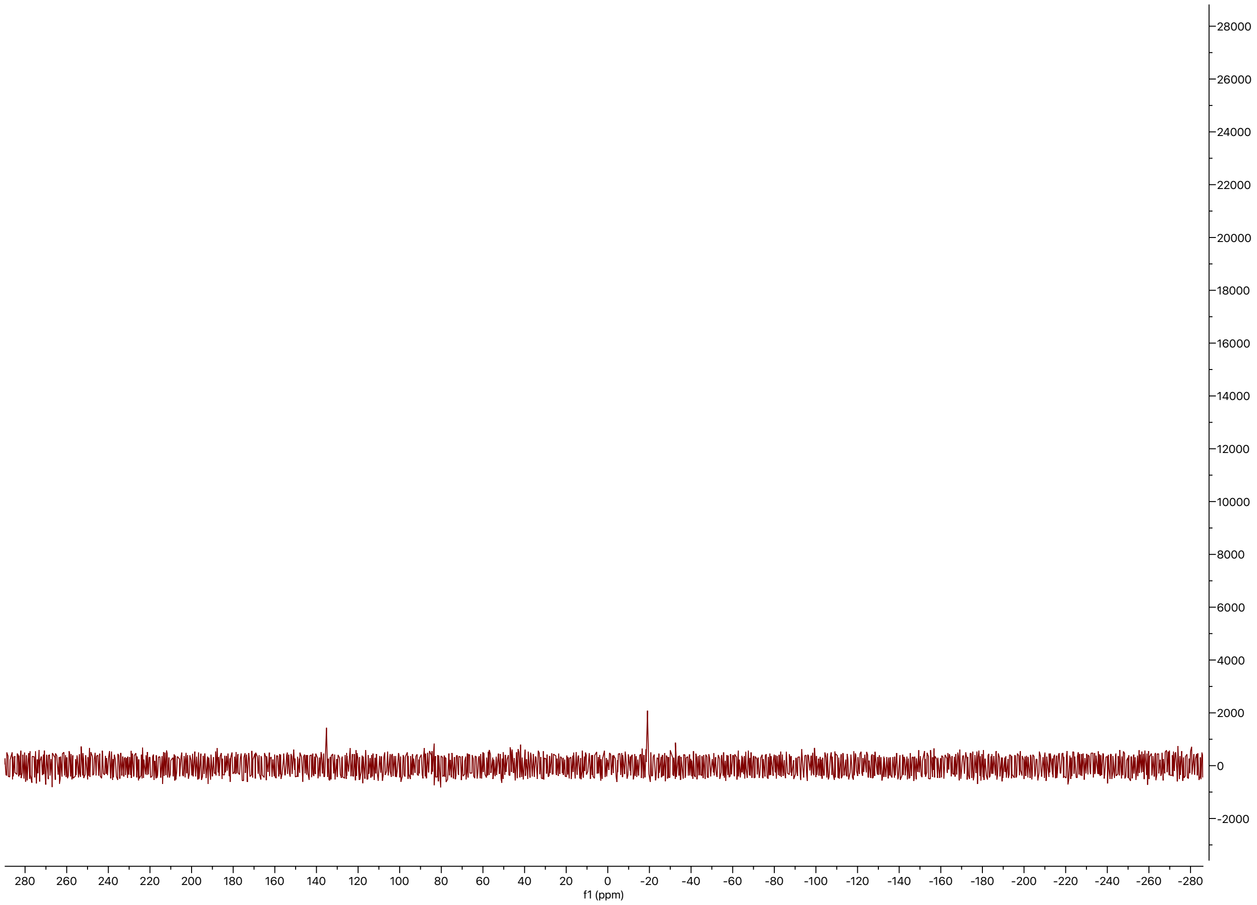

Figure S20: ^{31}P spectrum of bis[(2-diphenylphosphino)phenyl] ether in DCM-d_2.

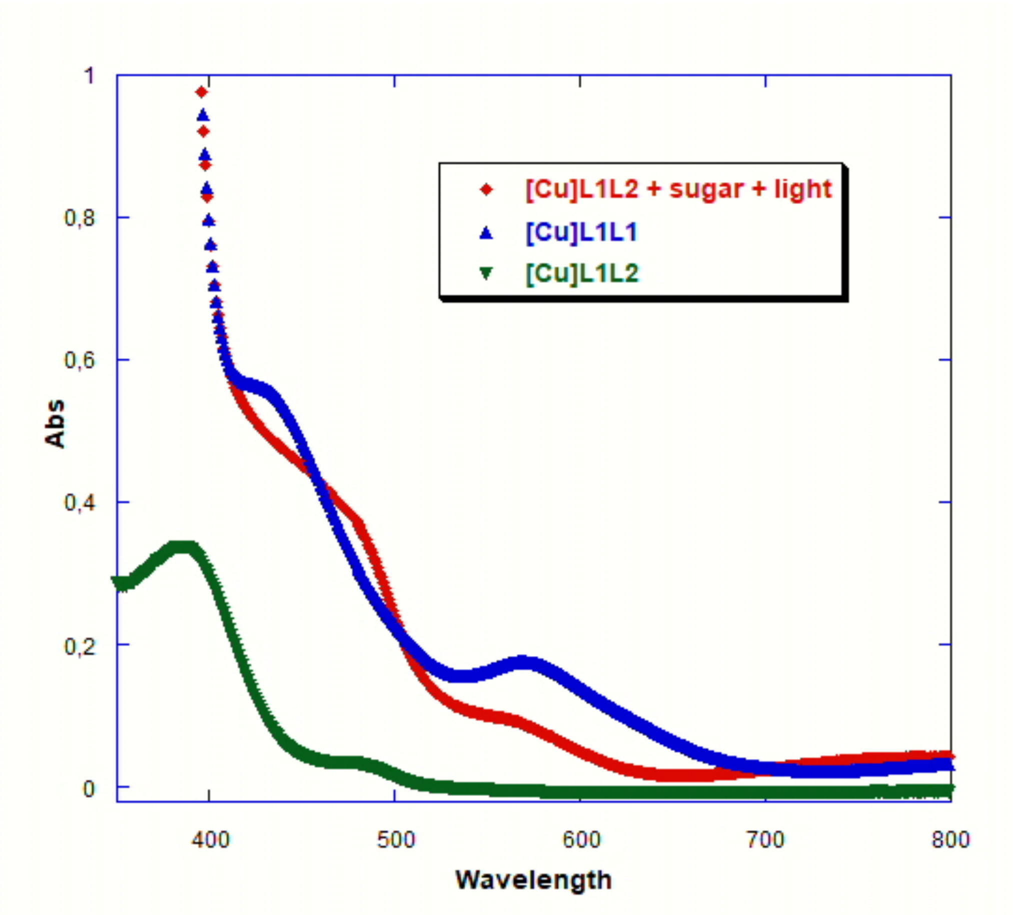

Figure S21: RED LINE UV-Vis spectra of: supernatant of test 3 (step 4), BLUE LINE homeleptic Cu complex, GREEN LINE heteroleptic Cu complex.

Test N°4:

Interaction between 2,3,4,6-Tetra-O-acetyl-α-glucopyranosyl bromide (1f) and [DPEPhos(bcp)Cu]PF_6 in MeCN-d_3/D_2O.

In this case we operate the same test described above (Test N°3) but with different steps.

1. Addition of 1eq of 2,3,4,6-Tetra-O-acetyl-α-glucopyranosyl bromide (**1f**) (0.015 mmol) and 1eq of (0.015 mmol) inside the NMR tube. The tube was left in dark for 45 minutes and then ^{1}H NMR was recorded;
2. 1 hour of irradiation with blue led light (10 W) and then ^{1}H NMR spectrum was recorded;
3. Addition of 1 eq of 2,3,4,6-Tetra-O-acetyl-α-glucopyranosyl bromide (**1f**) and another hour of irradiation and then ^{1}H NMR spectrum was recorded;
4. Addition of another eq of 2,3,4,6-Tetra-O-acetyl-α-glucopyranosyl bromide (**1f**) and another hour of irradiation and then ^{1}H NMR was recorded.

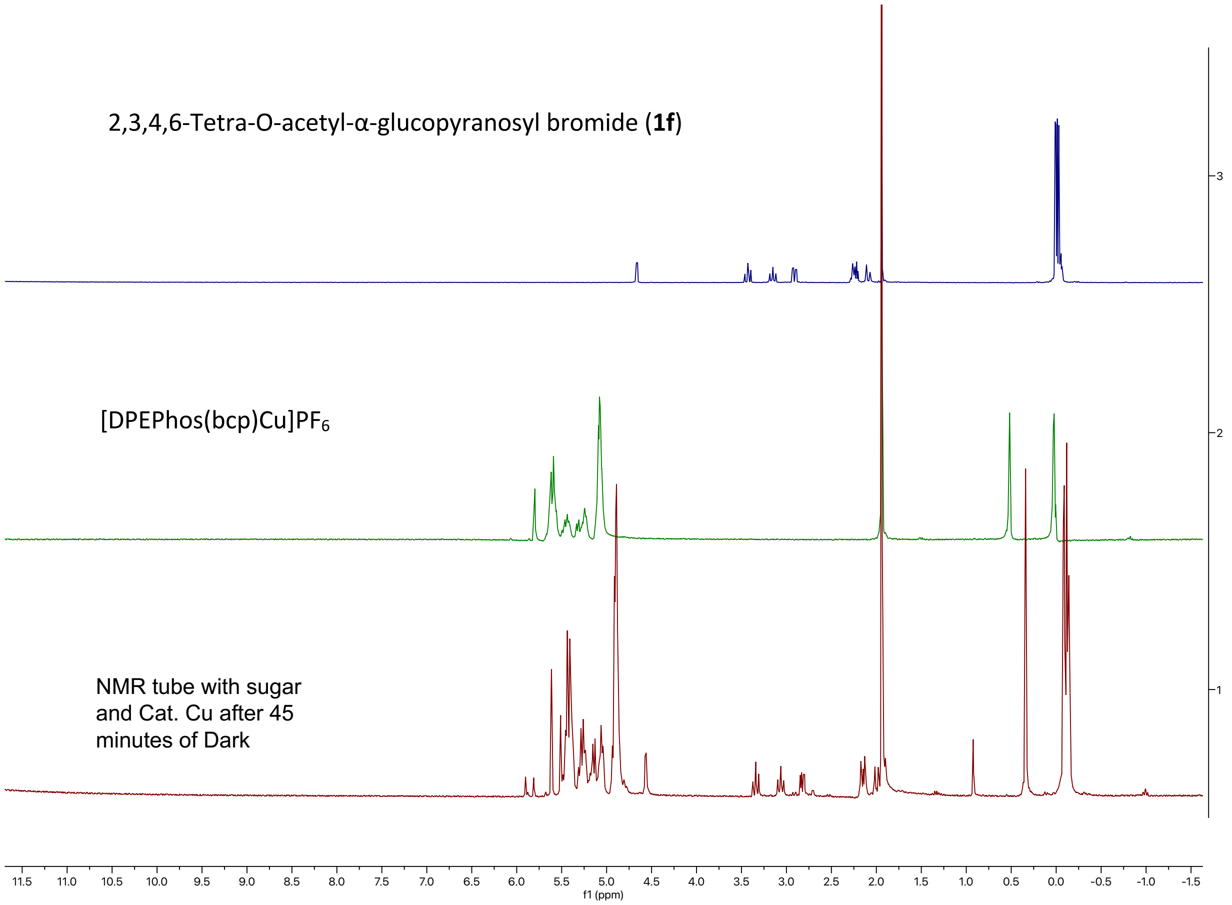

Figure S22: Comparison between 2,3,4,6-Tetra-O-acetyl-α-glucopyranosyl bromide (**1f**)*,* [DPEPhos(bcp)Cu]PF_6 *and NMR tube with sugar and catalyst recorded after 45 minutes of dark.*

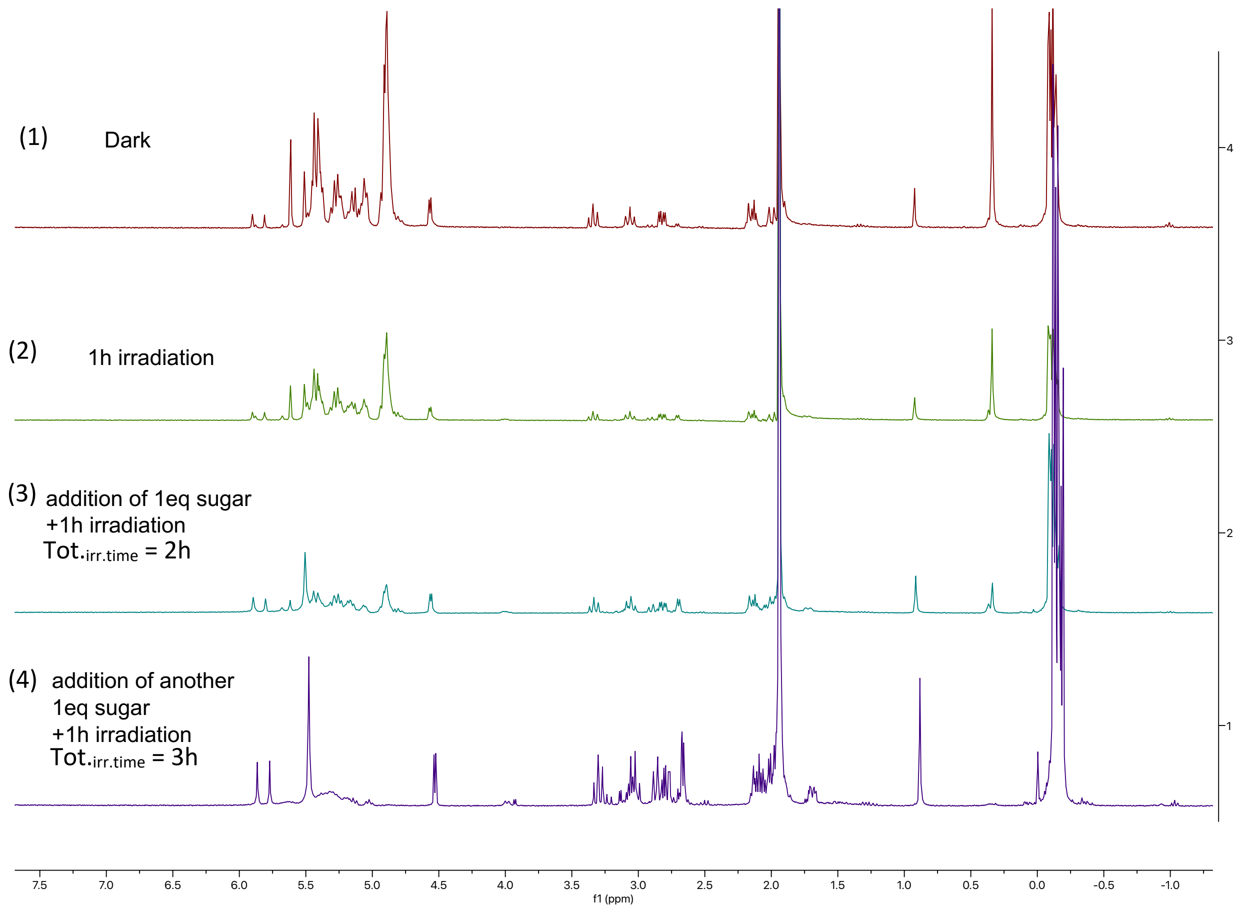

Figure S23: Comparison of NMR spectra between NMR tube left 45 minutes in dark (1), after 1h of irradiation (2), after addition of sugar and another hour of irradiation (3) and another addition of sugar and another hour of irradiation (4).

As can be seen from figure S21, after 45 minutes of dark, there is already the formation of new peaks (area from 5.8 to 6.0 ppm) and a shift between the ^{1}H-NMR spectra of the photocatalyst and the spectra of the mixture (sugar/photocatalyst) after 45 minutes of dark. After 1h of irradiation the spectra didn't change and is similar to the one recorded after dark, in this case there isn't formation of precipitate. At this point, has been added 1eq of the sugar, and the tube was left another hour under irradiation. After this time is clear the formation of a bit of precipitate. As can be seen from the spectra (Figure S23, (3)), there is an important variation of signals in the aromatic zone which becomes more chaotic. Has been added another equivalent of sugar and the tube was left under irradiation another hour. After this hour there is more precipitate, and the spectra clearly shows the disappearance of the main signals of the [DPEPhos(bcp)Cu]PF_6 in the aromatic zone and an increase of signals (5.5 ppm, 5.7 ppm, 5.9 ppm) probably related to homoleptic photocatalyst ([$(bcp)_2$Cu]PF_6) as can be seen from Figure S23, Furthermore, there are new signals, probably related to the sugar at 1.7ppm and 2.88ppm.

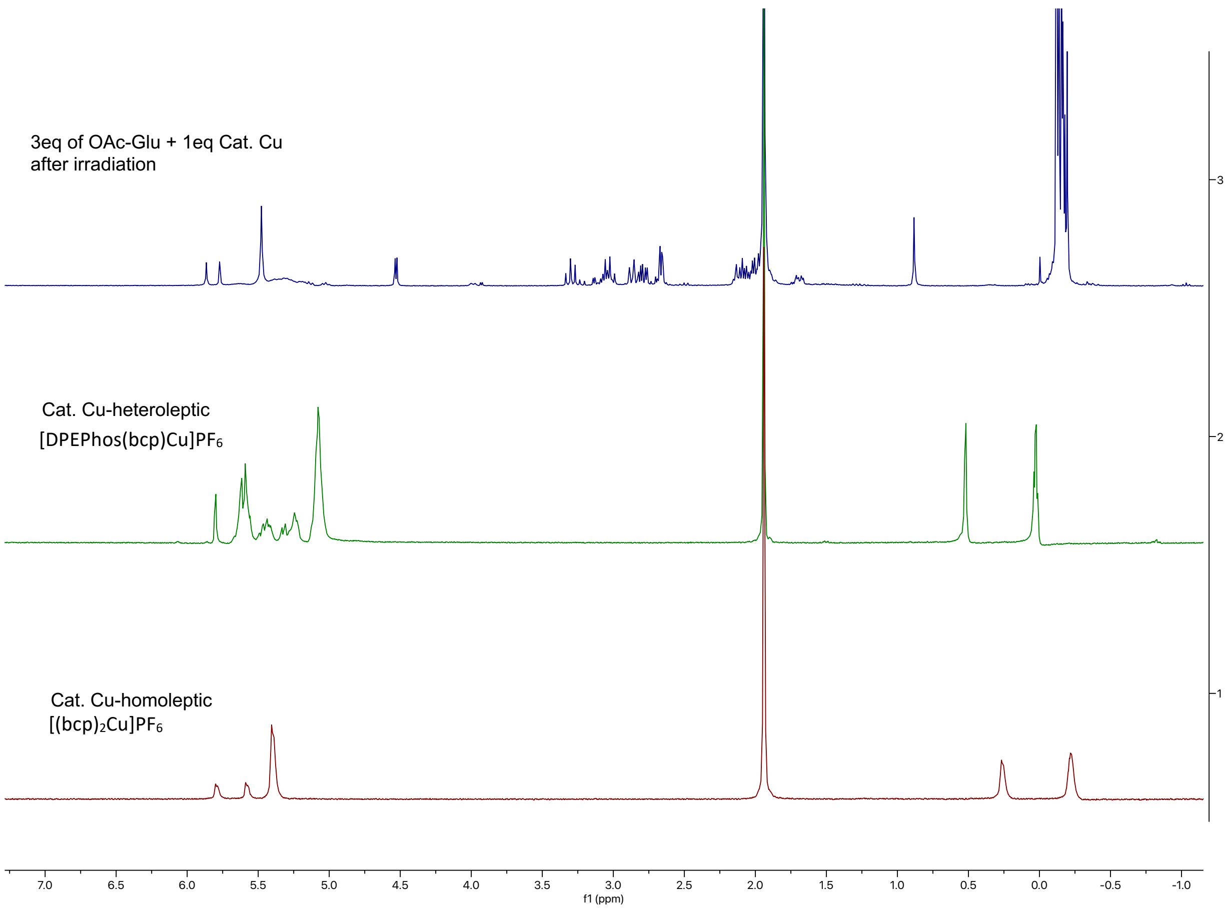

*Figure S24: Comparison between Cat. homoleptic ([(bcp)$_2$Cu]PF$_6$), Cat. Cu heteroleptic ([DPEPhos(bcp)Cu]PF$_6$) and the mixture of 3eq of 2,3,4,6-Tetra-O-acetyl-α-glucopyranosyl bromide (**1f**) and 1eq of Cu heteroleptic after irradiation (point (3) of Figure S23).*

REACTION CARRIED OUT IN DEUTERATED DICHLOROMETHANE

In order to understand if the radical quenching is due to the Hantzsch ester or came from the solvent, we performed the reaction using deuterated dichloromethane. If the quenching is due to the solvent, in the final NMR spectra, the H2 signal (red one in Figure S25) should not appear.

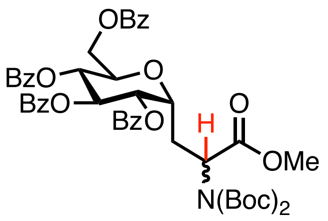

Figure S25: Main product of the photocatalyzed reaction with H2 in red

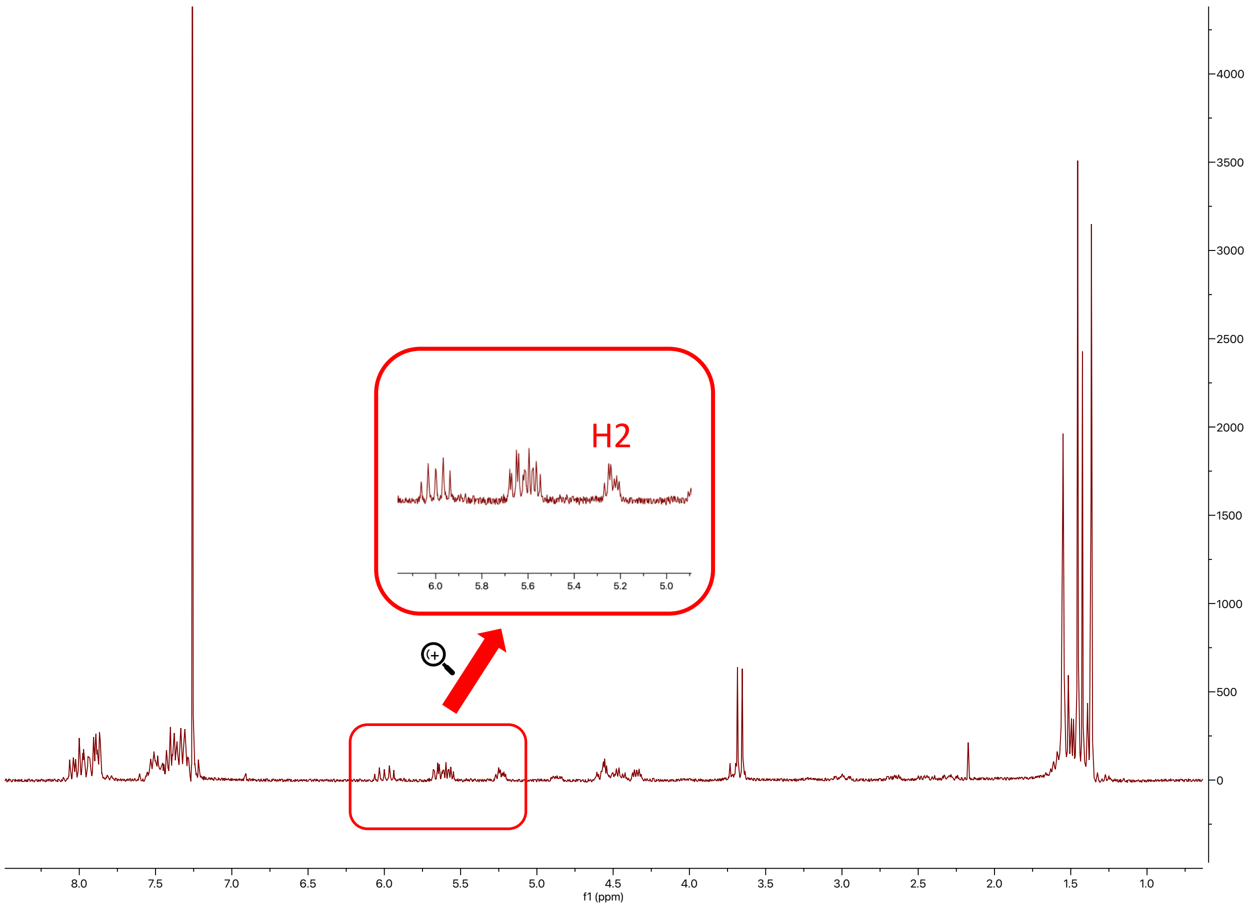

Figure S26: ^{1}H NMR of the product of the reaction carried out in DCM and NMR performed in DCM-d_2.

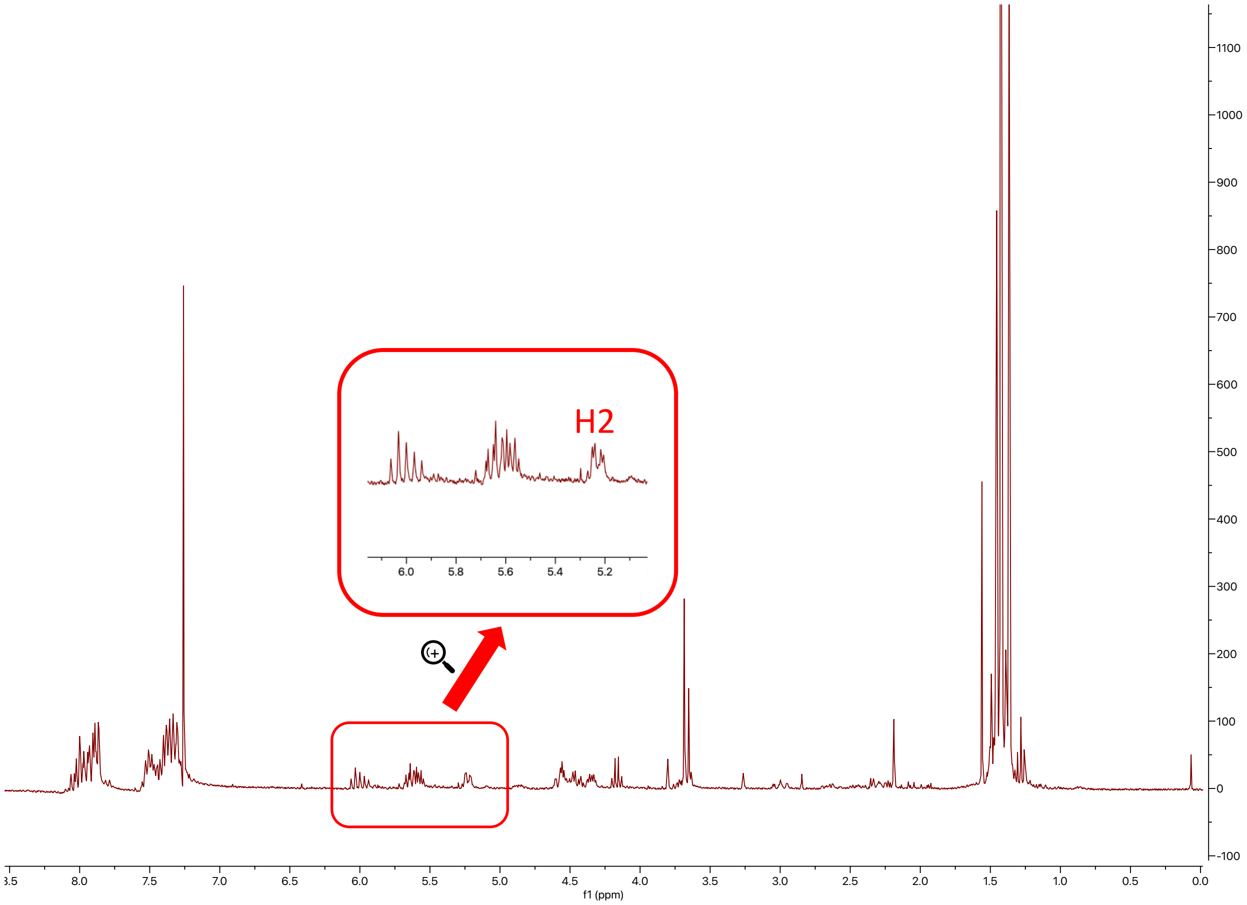

Figure S27: *^{1}H NMR of the product of the reaction carried out in DCM-d_2 and NMR performed in DCM-d_2.*

Comparing the two spectra reported above, is clear that the radical quenching is not due to the solvent, in fact the signal of the H2 is still visible in the spectra of the product isolated from the reaction carried out in DCM-d_2.

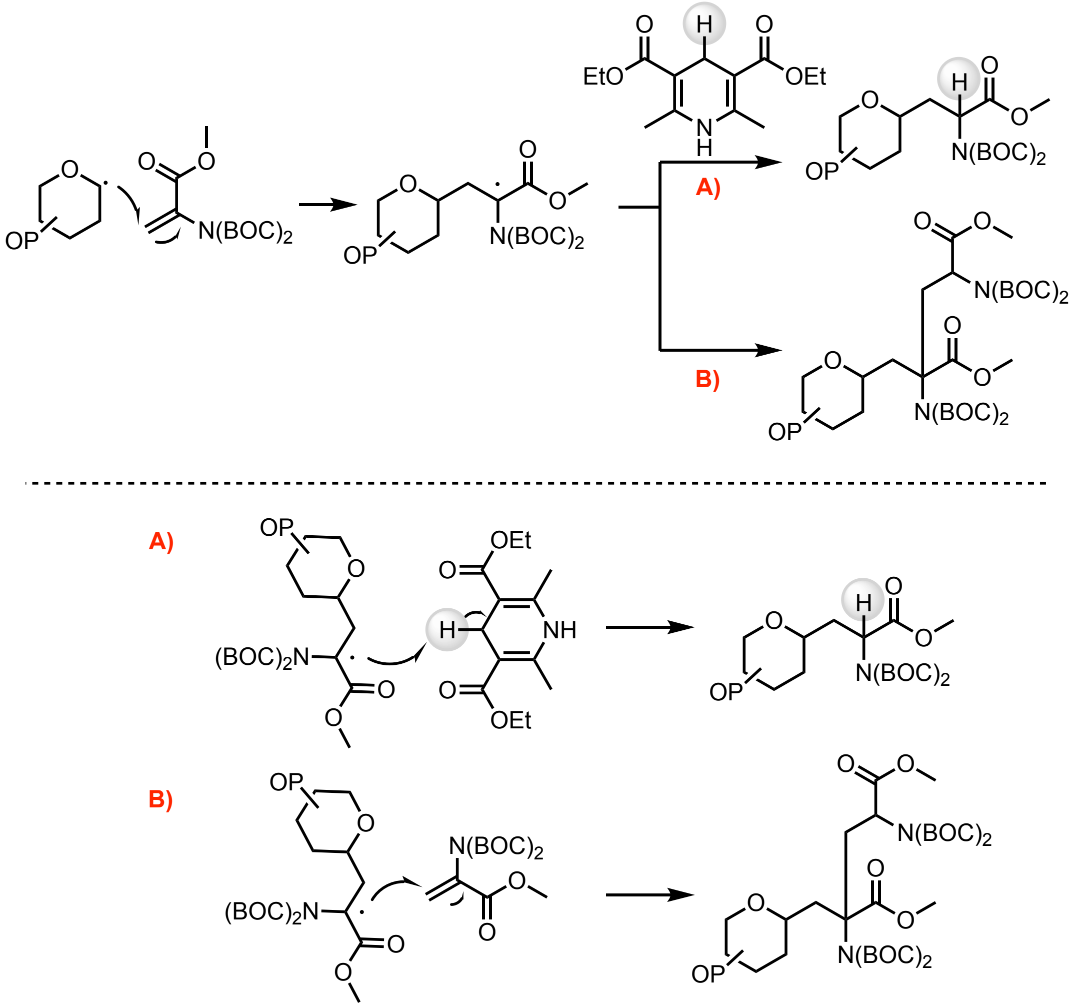

Figure S28: Complete mechanism which includes consecutive addition to olefin (path B)

SPECTRA OF STARTING COMPOUNDS

^{1}H-NMR (300 MHz),^{13}C{^{1}H}-NMR (101 MHz) of *1,2,3,4,6-Penta-O-benzoyl-α-D-glucopyranose (4) - (CDCl$_3$).*

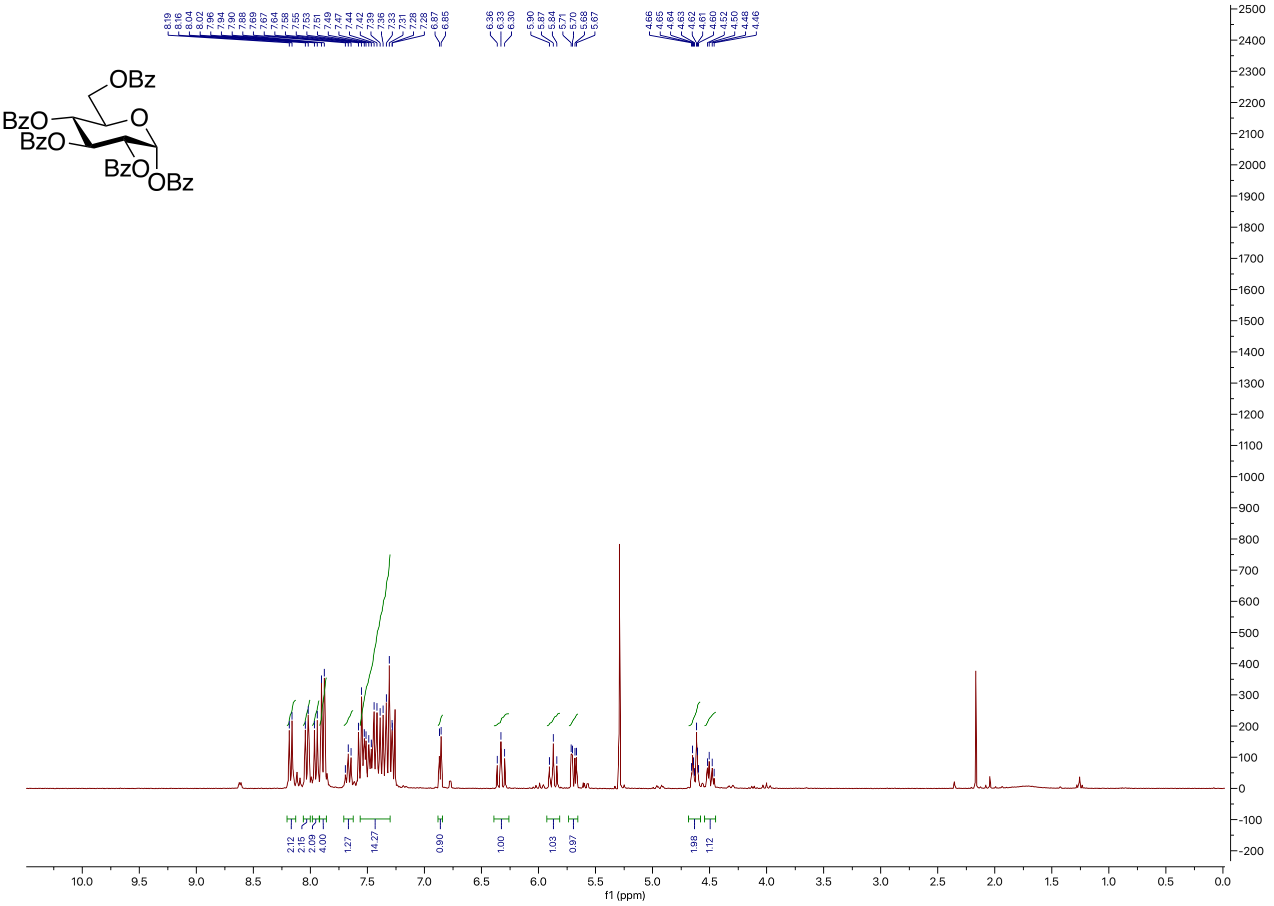

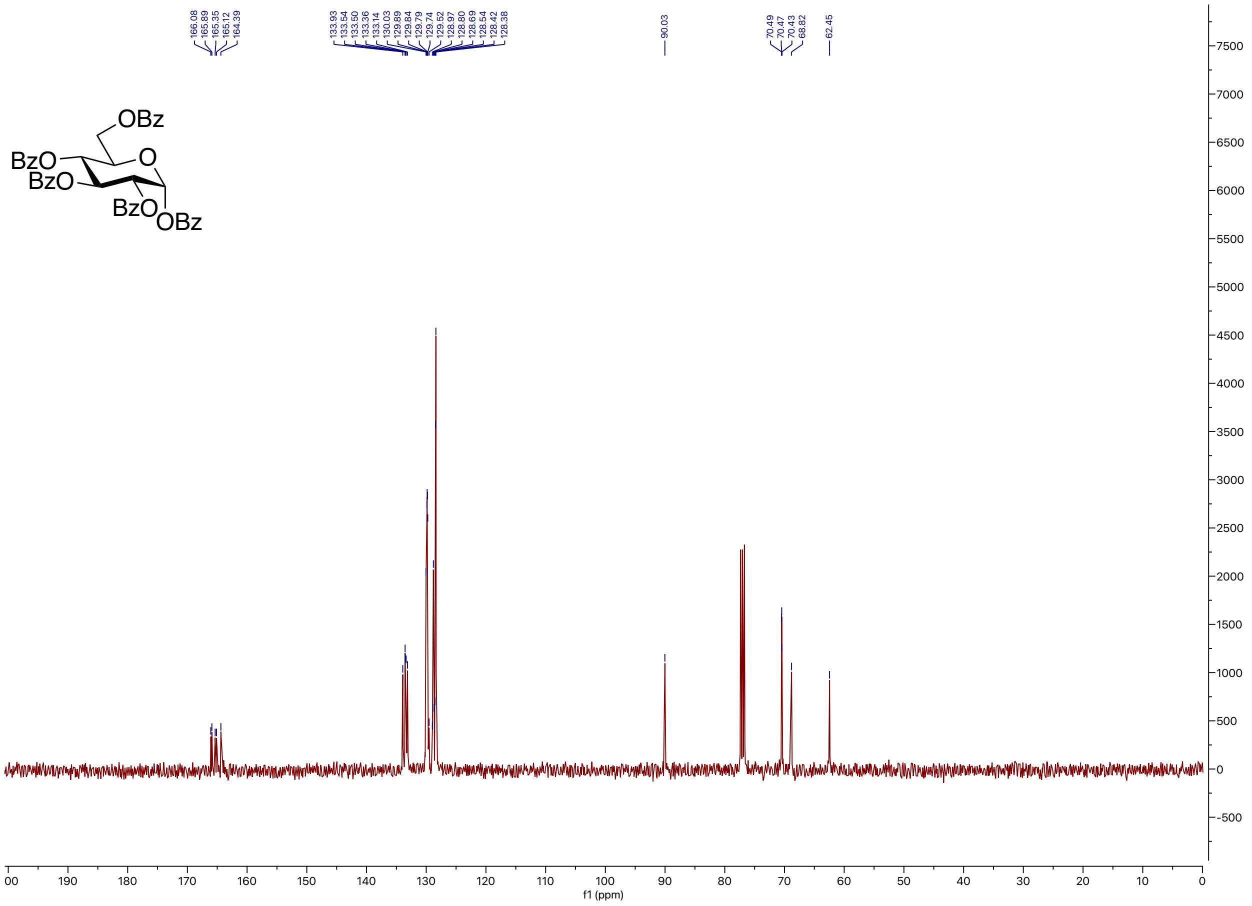

^{1}H-NMR (300 MHz),^{13}C{^{1}H}-NMR (101 MHz) of *2,3,4,6-Tetra-O-benzoyl-α-D-glucopyranosyl bromide (1a)* - *(CDCl$_3$).*

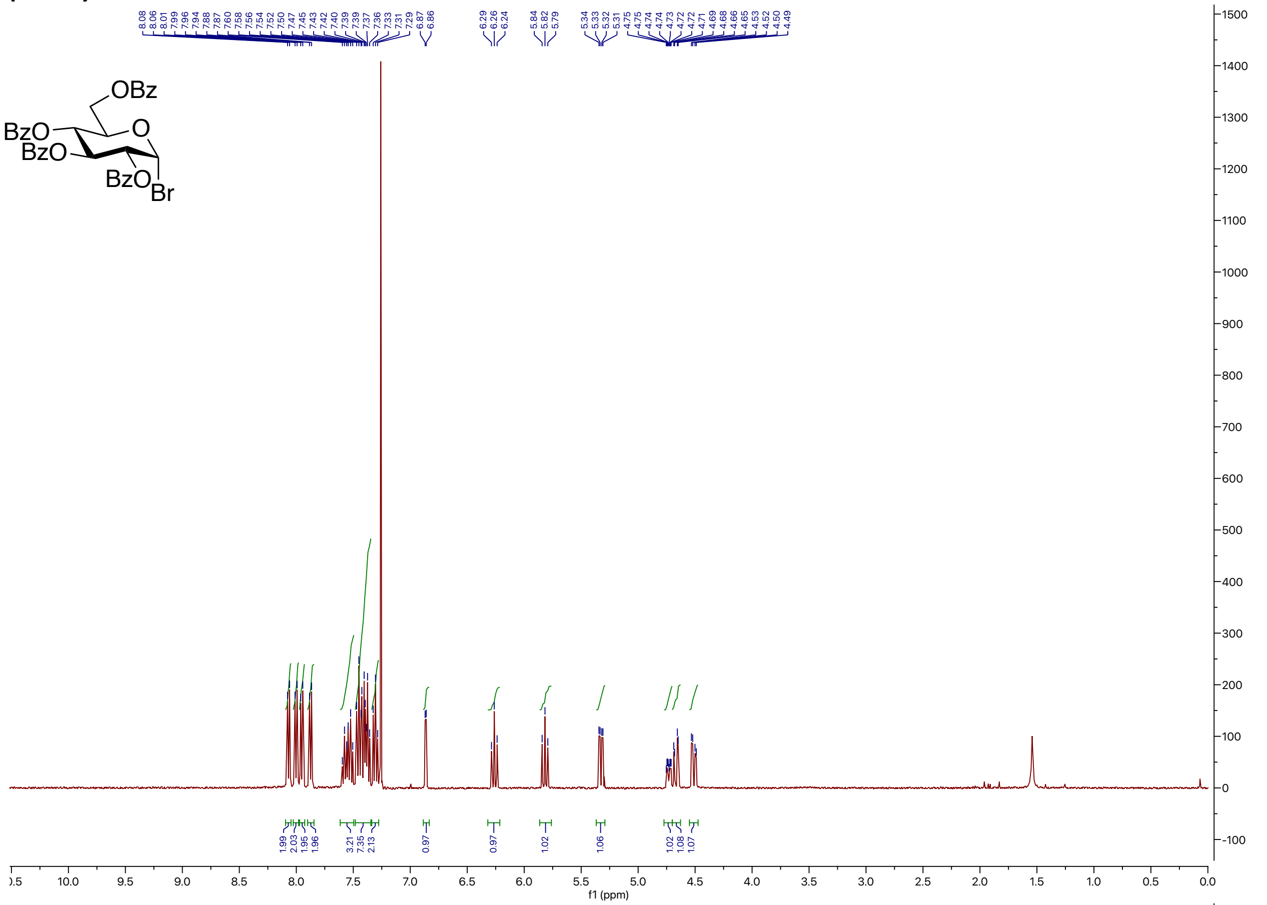

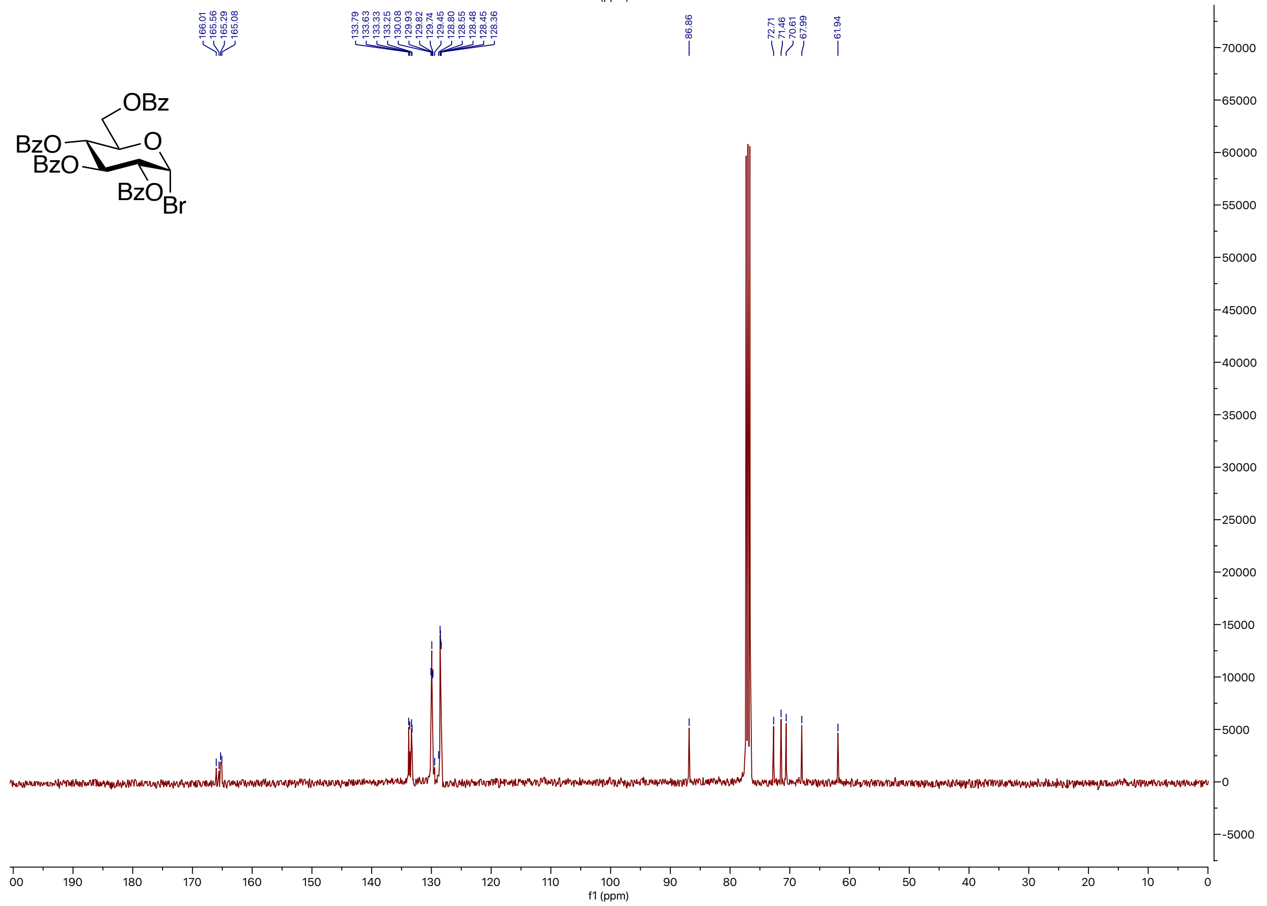

^{1}H-NMR (400 MHz),^{13}C{^{1}H}-NMR (101 MHz) of *1,2,3,4,6-Tetra-O-benzoyl-α/β-D-mannopyranose (5) - ($CDCl_3$).*

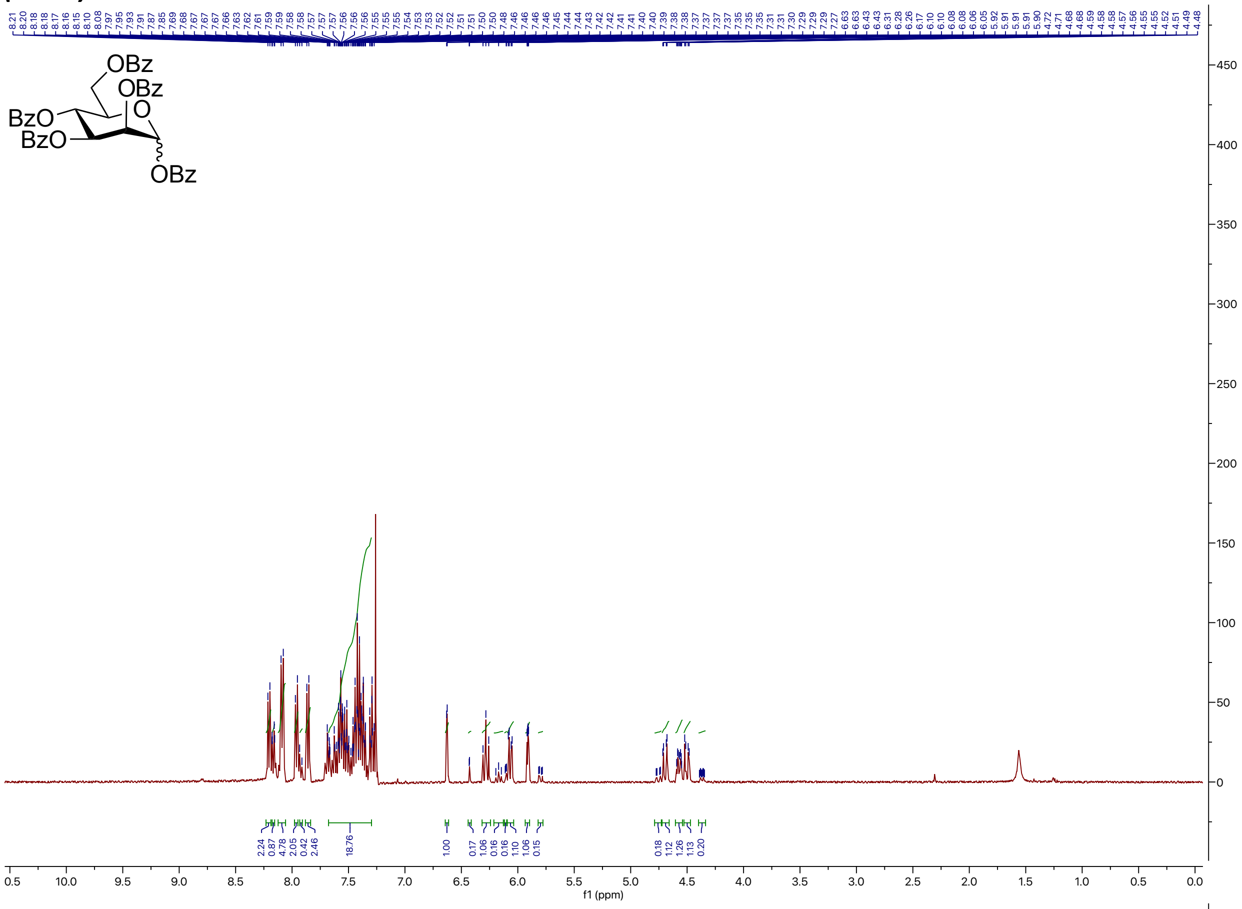

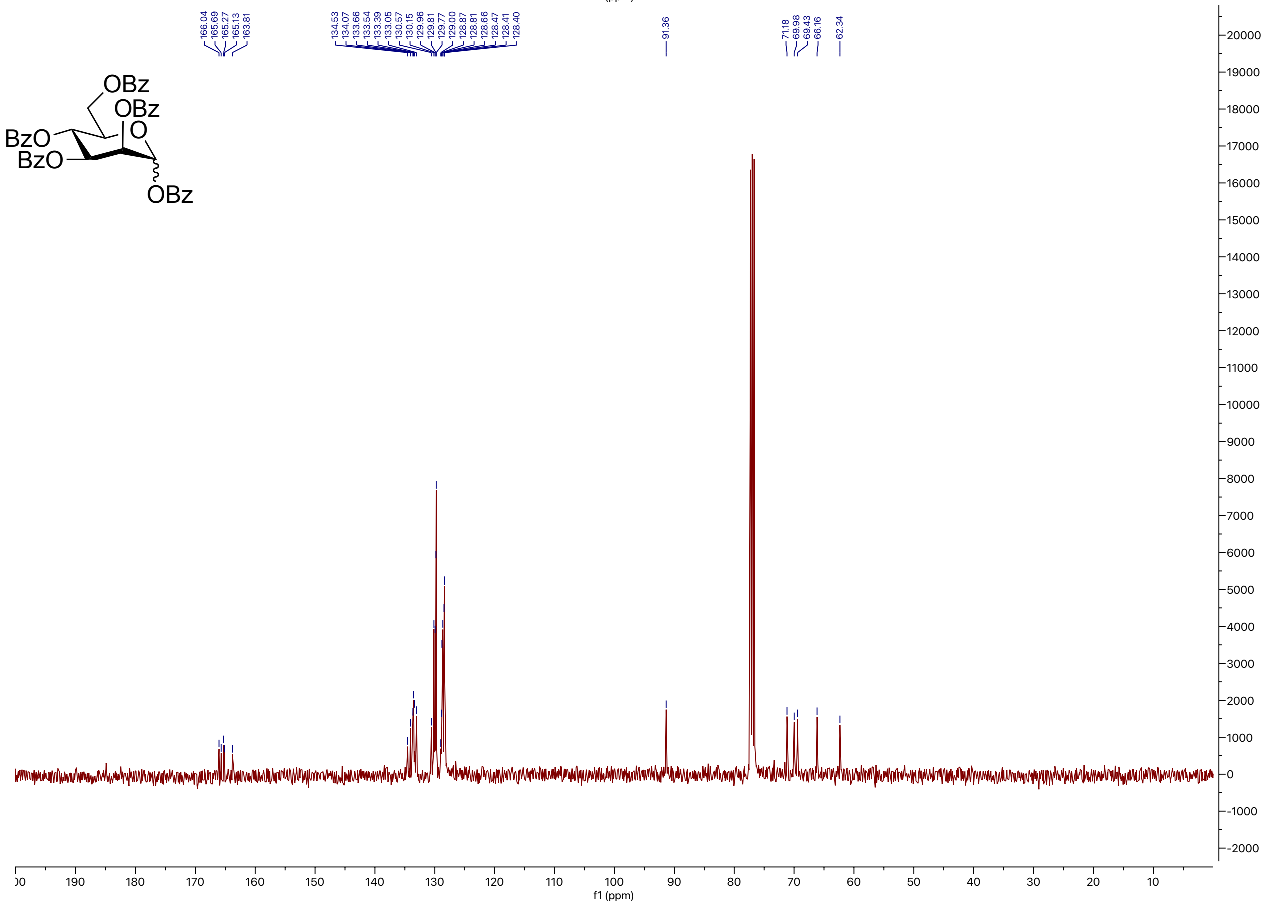

^{1}H-NMR (400 MHz),^{13}C{^{1}H}-NMR (101 MHz) of *2,3,4,6-Tetra-O-benzoyl-α-mannopyranosyl bromide (1b) - (CDCl$_3$).*

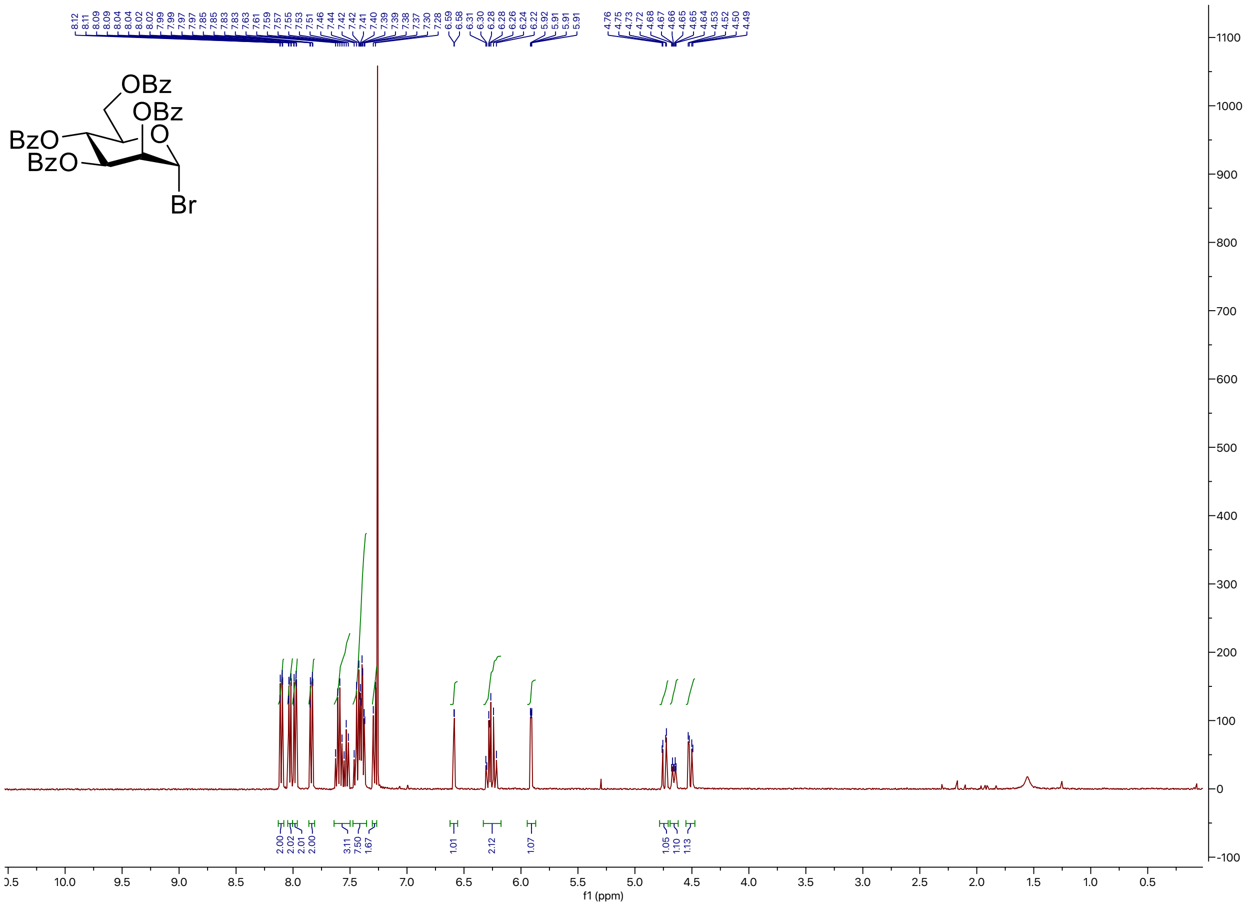

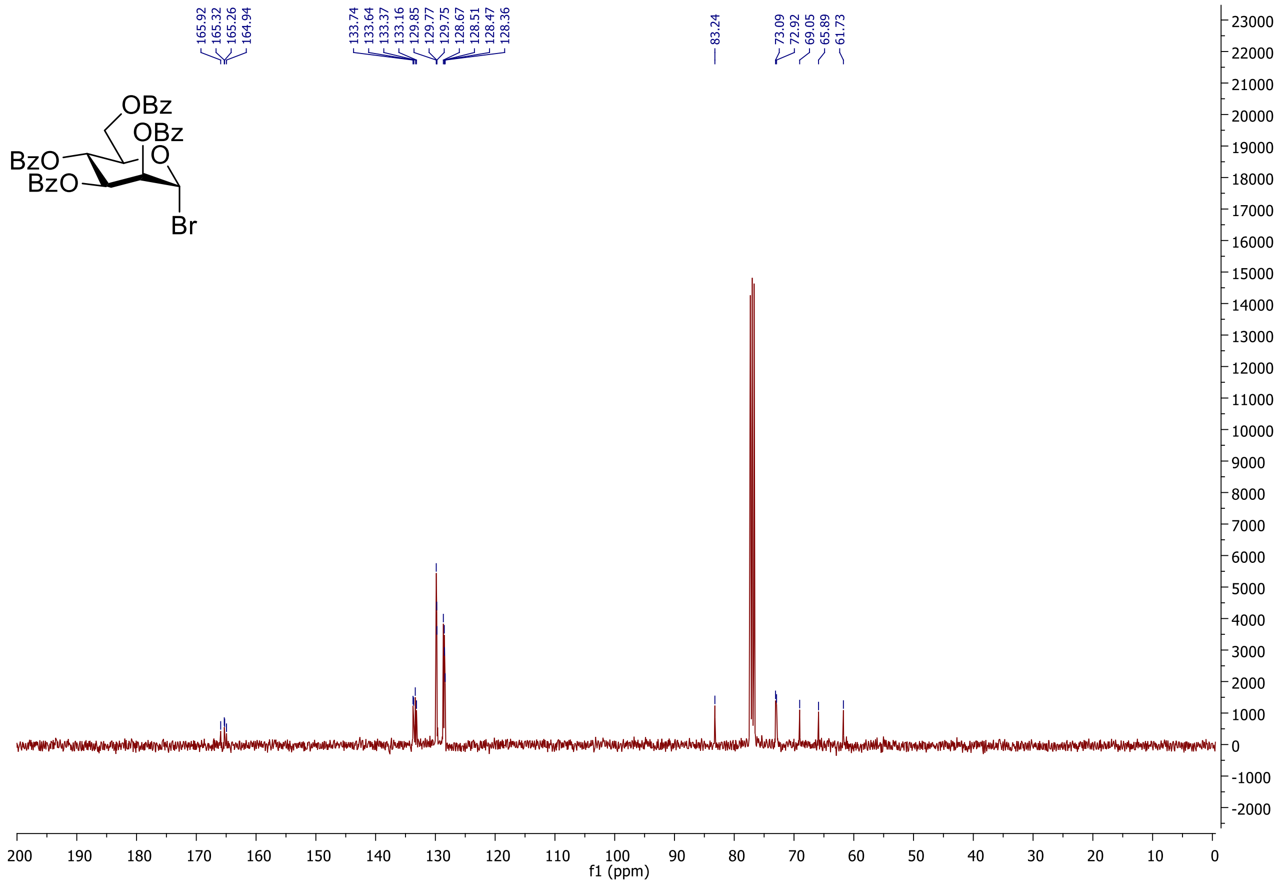

^{1}H-NMR (400 MHz),^{13}C{^{1}H}-NMR (101 MHz) of *1,2,3,4,6-Penta-O-benzoyl-α-D-galactopyranose (6) - (CDCl$_3$).*

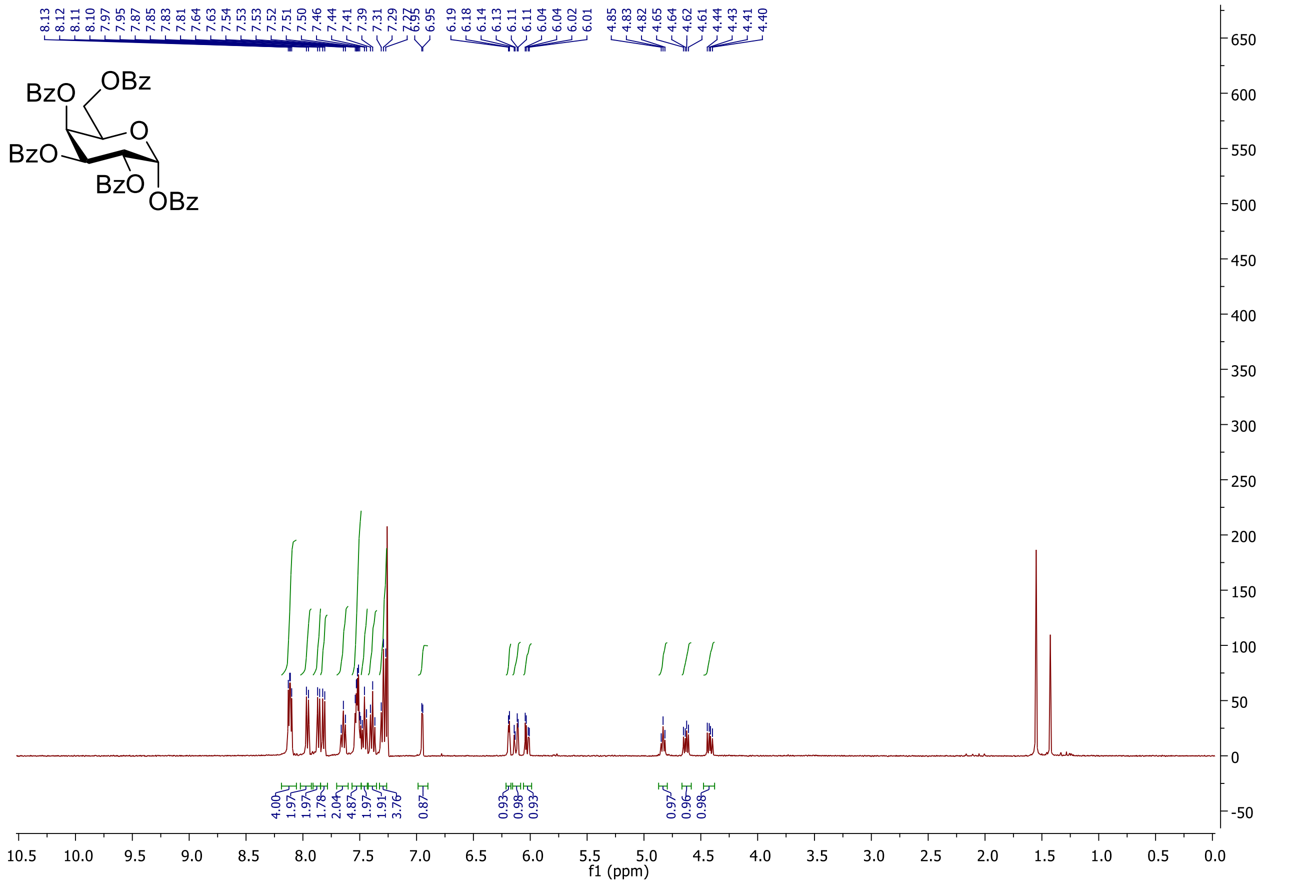

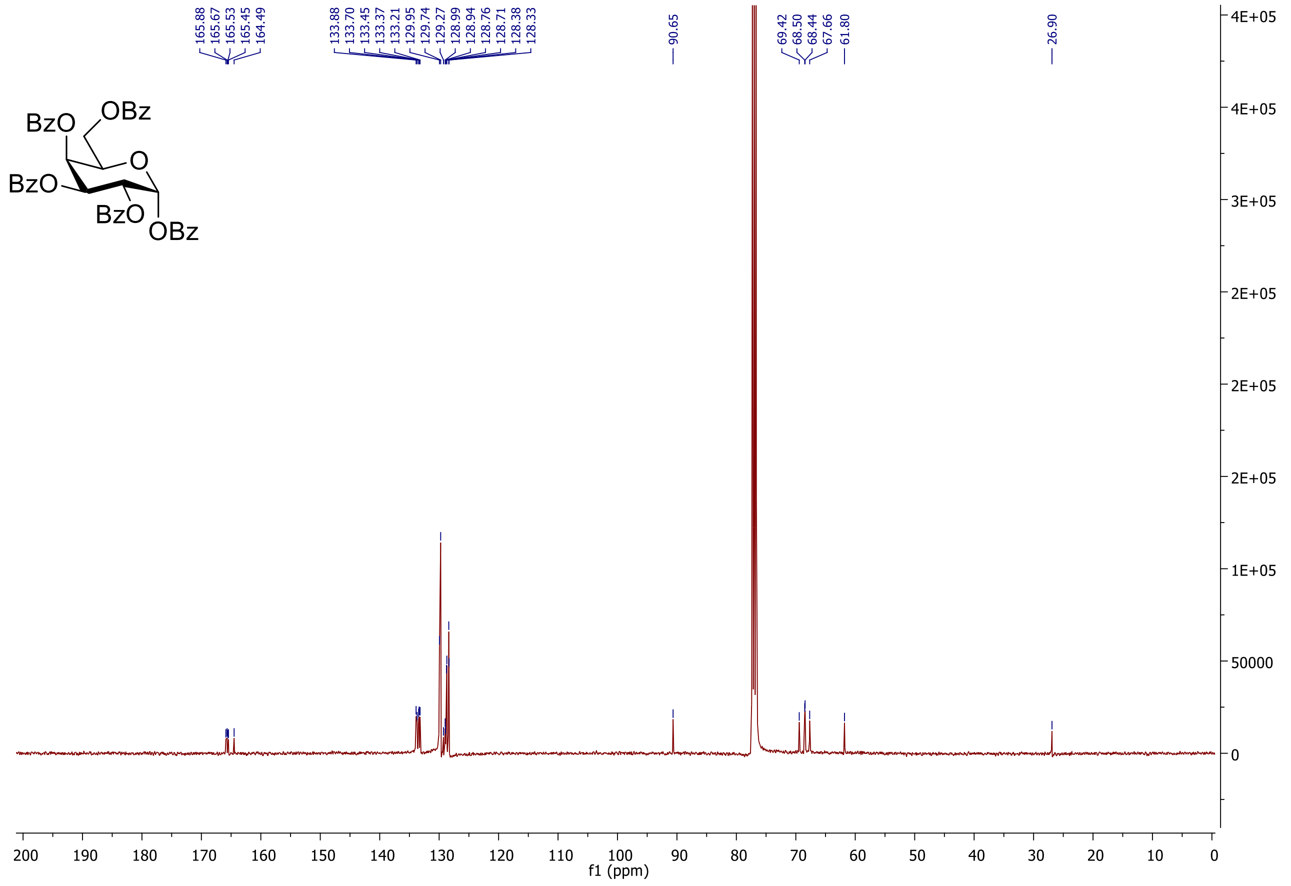

^{1}H-NMR (300 MHz),^{13}C{^{1}H}-NMR (101 MHz) of *2,3,4,6-Tetra-O-benzoyl-galactopyranosyl bromide (1c) - (CDCl$_3$).*

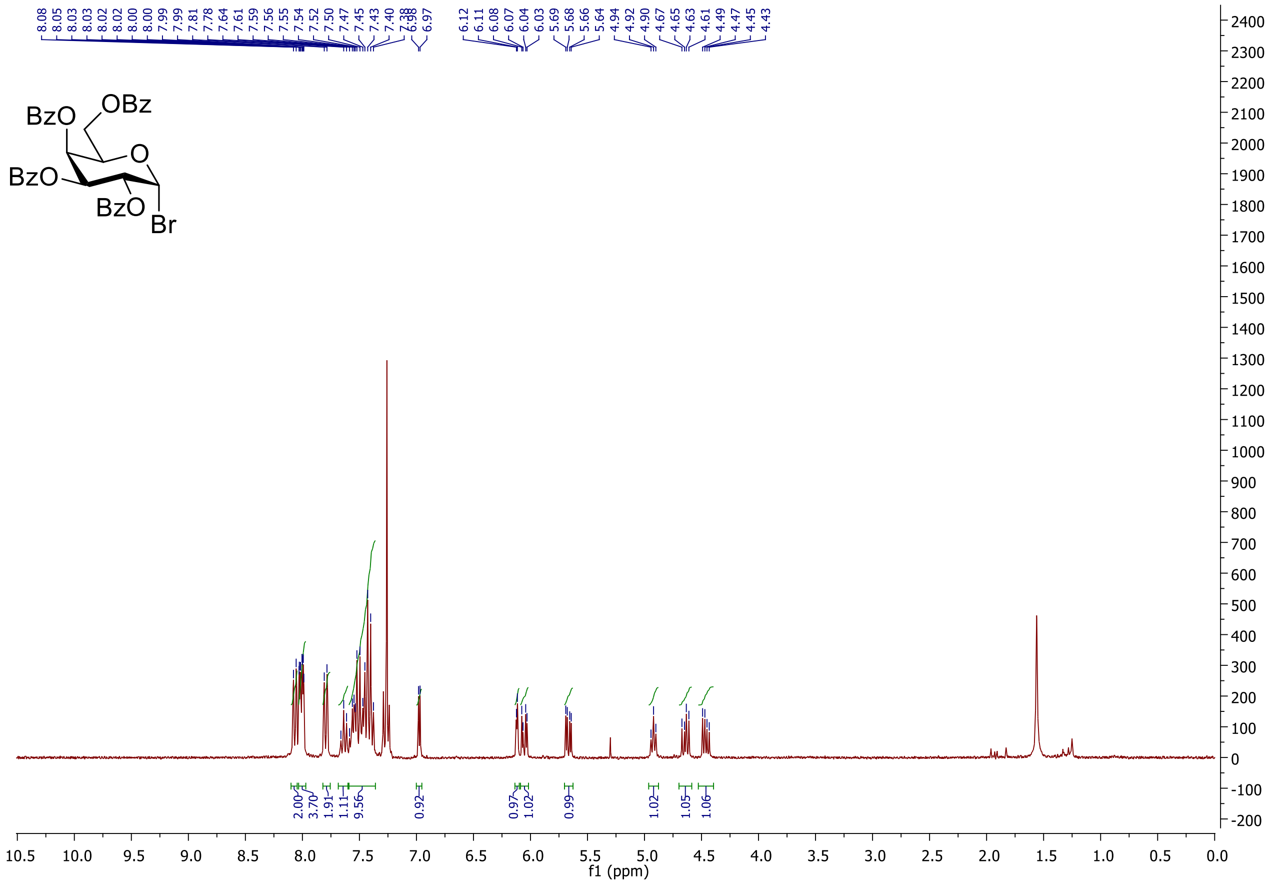

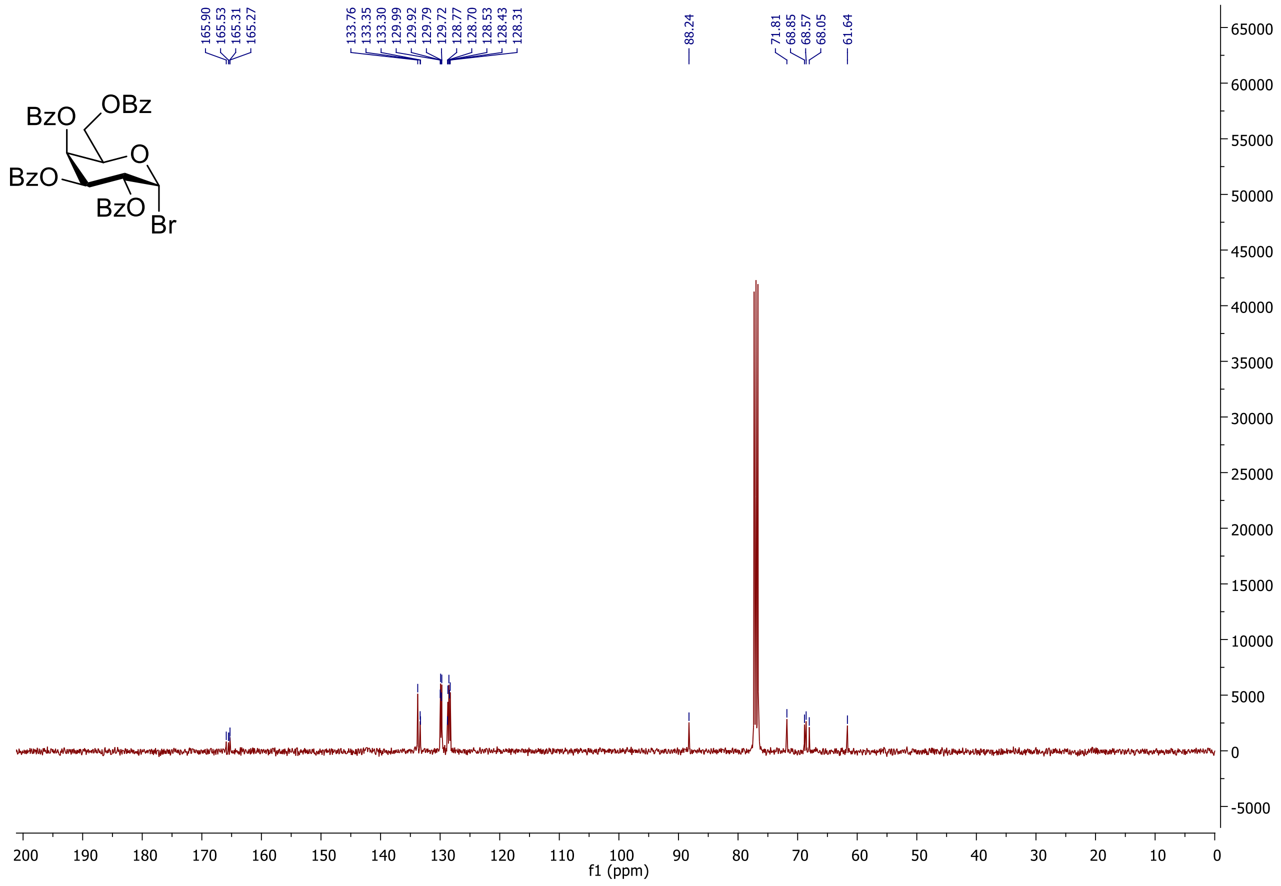

^{1}H-NMR (400 MHz),^{13}C{^{1}H}-NMR (101 MHz) of *2,3,4,6-Tetra-O-acetyl-α-glucopyranosyl bromide (1f) - (CDCl$_3$).*

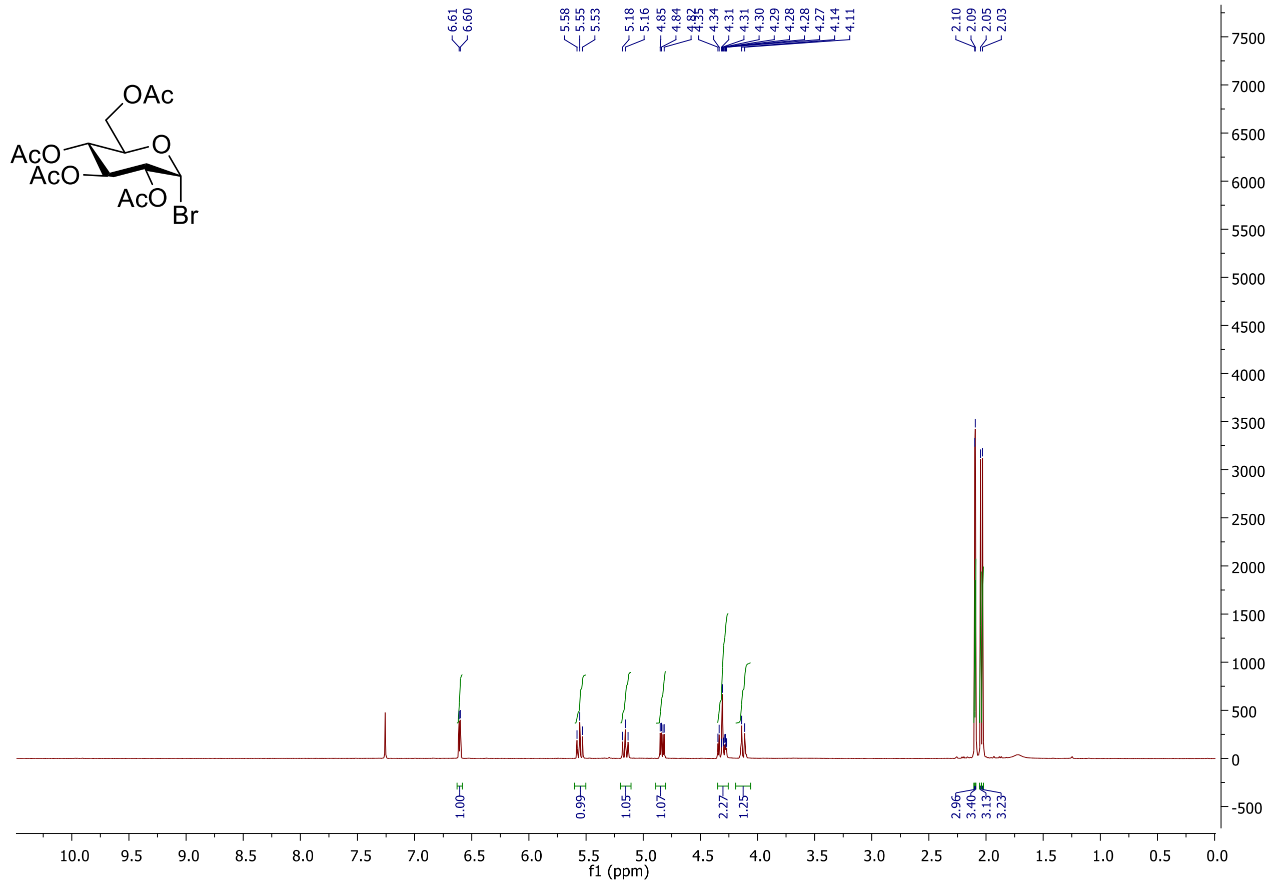

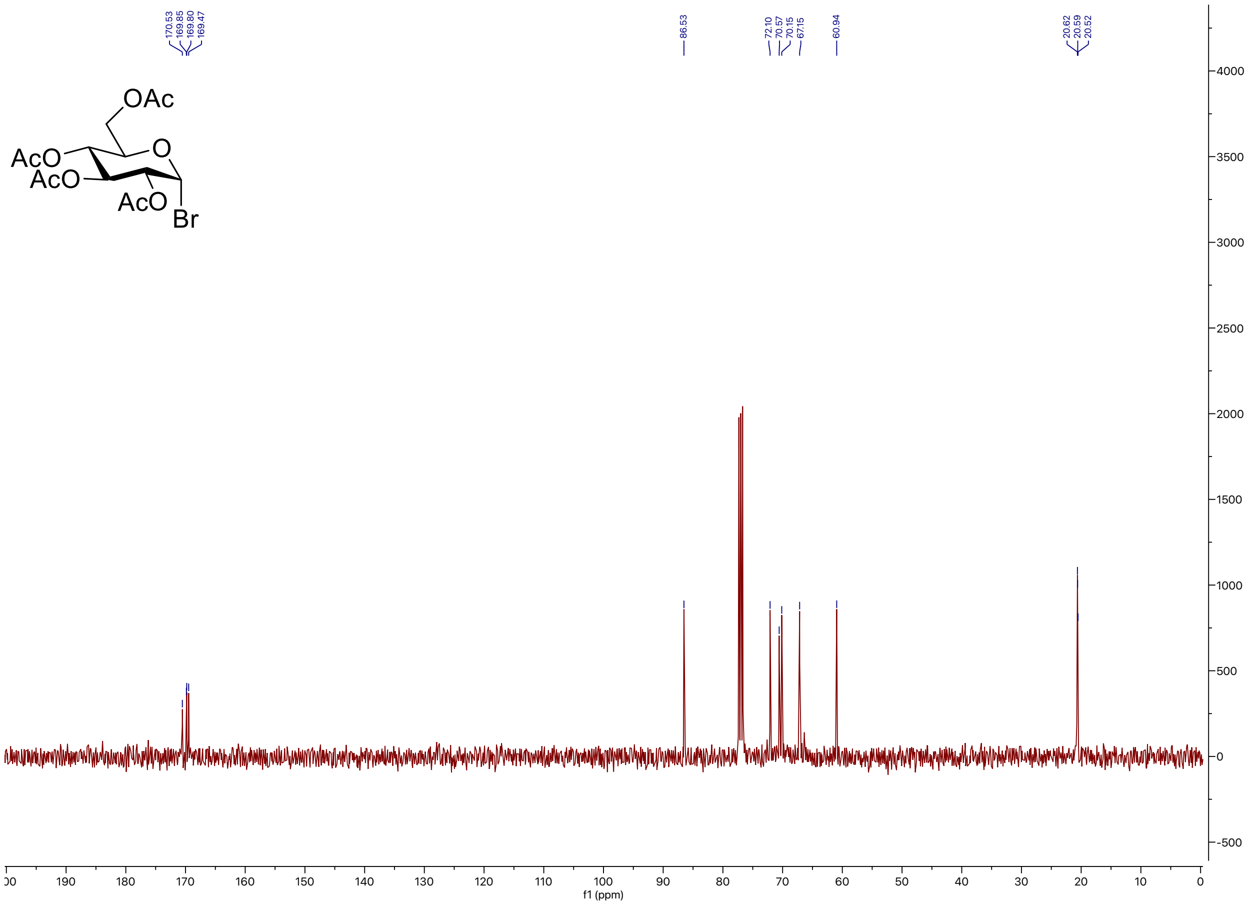

^{1}H-NMR (400 MHz),^{13}C{^{1}H}-NMR (101 MHz) of *1,2,3,4,6-Penta-O-acetyl-α/β-mannopyranose (7)* - ($CDCl_3$).

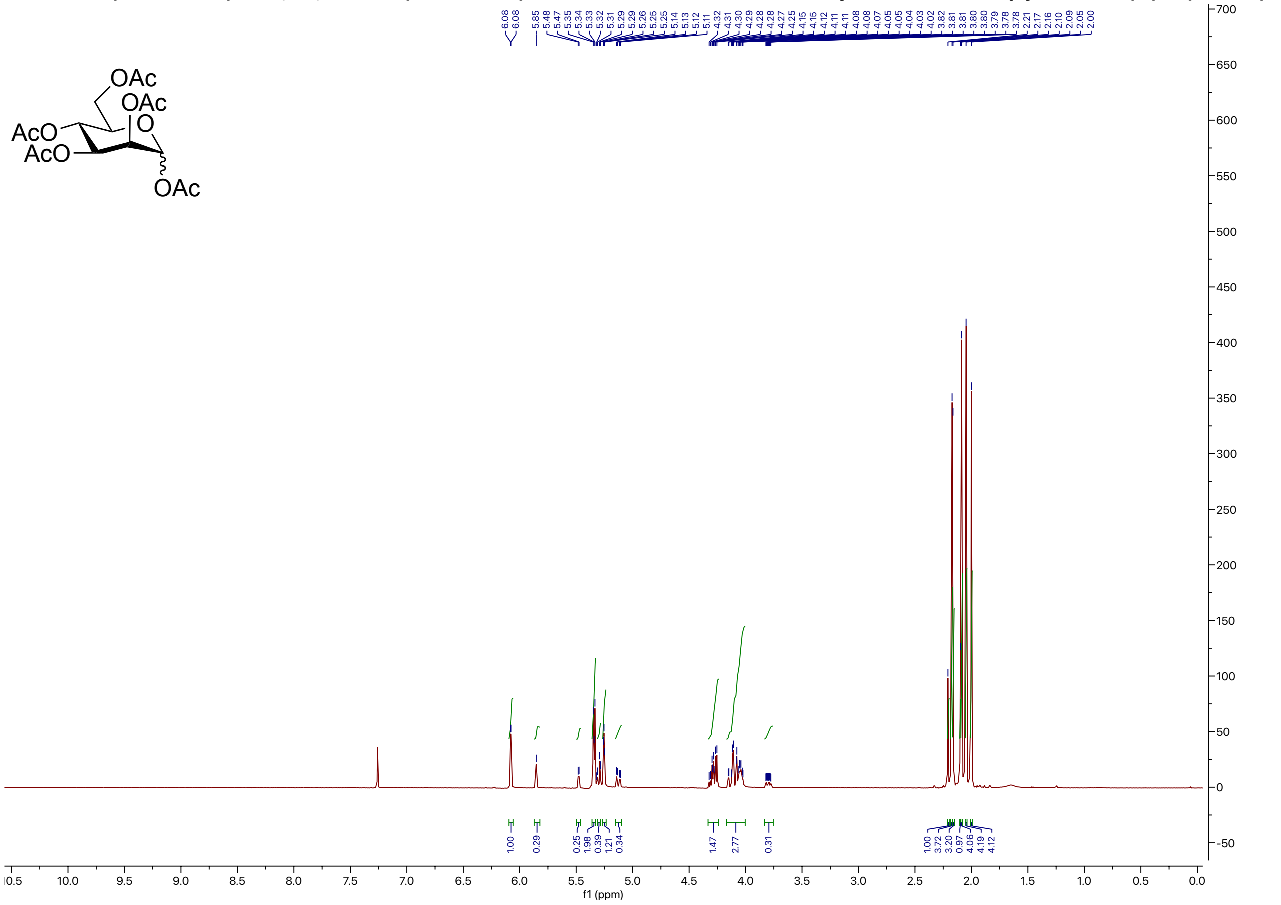

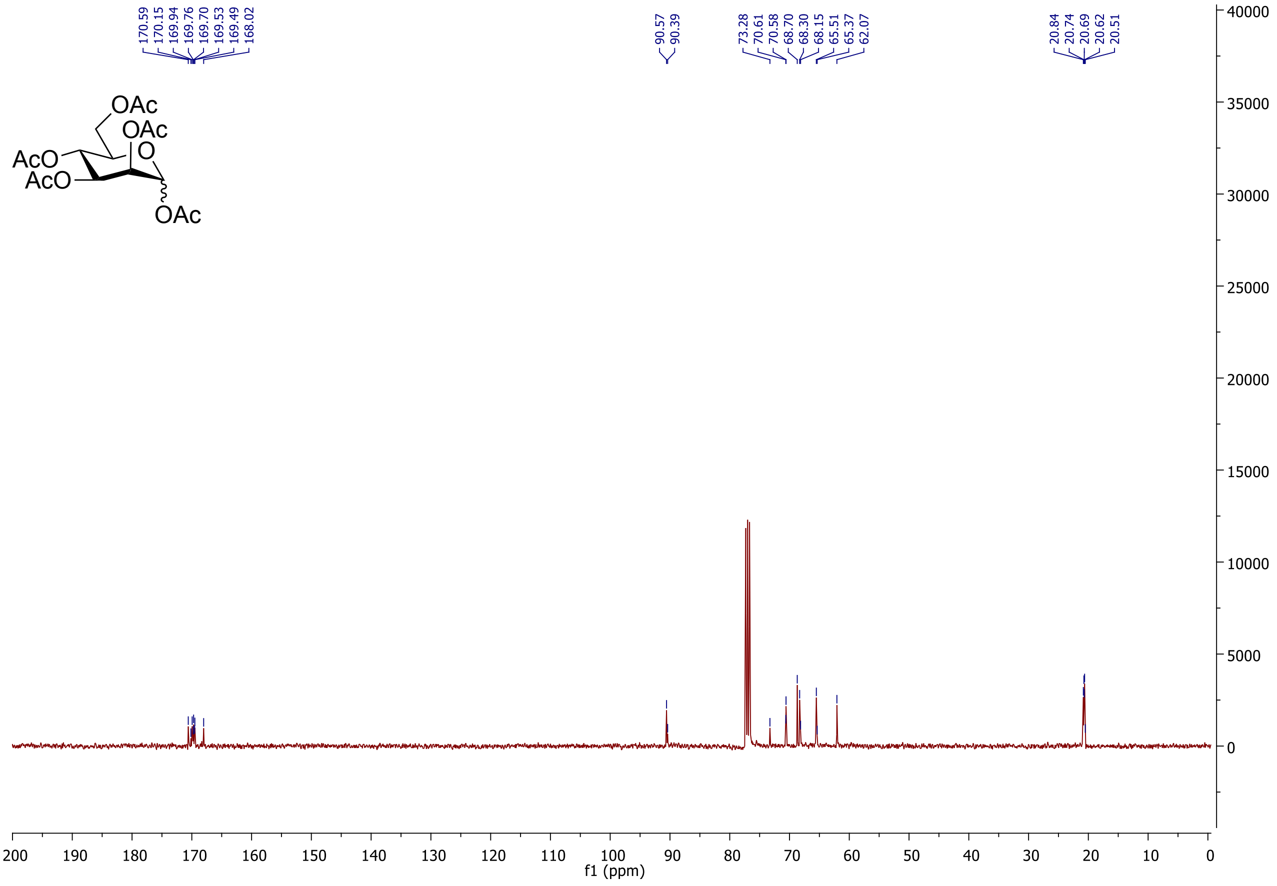

^{1}H-NMR (400 MHz),^{13}C{^{1}H}-NMR (101 MHz) of *2,3,4,6-Tetra-O-acetyl-α-mannopyranosyl bromide (1g) - (CDCl₃).*

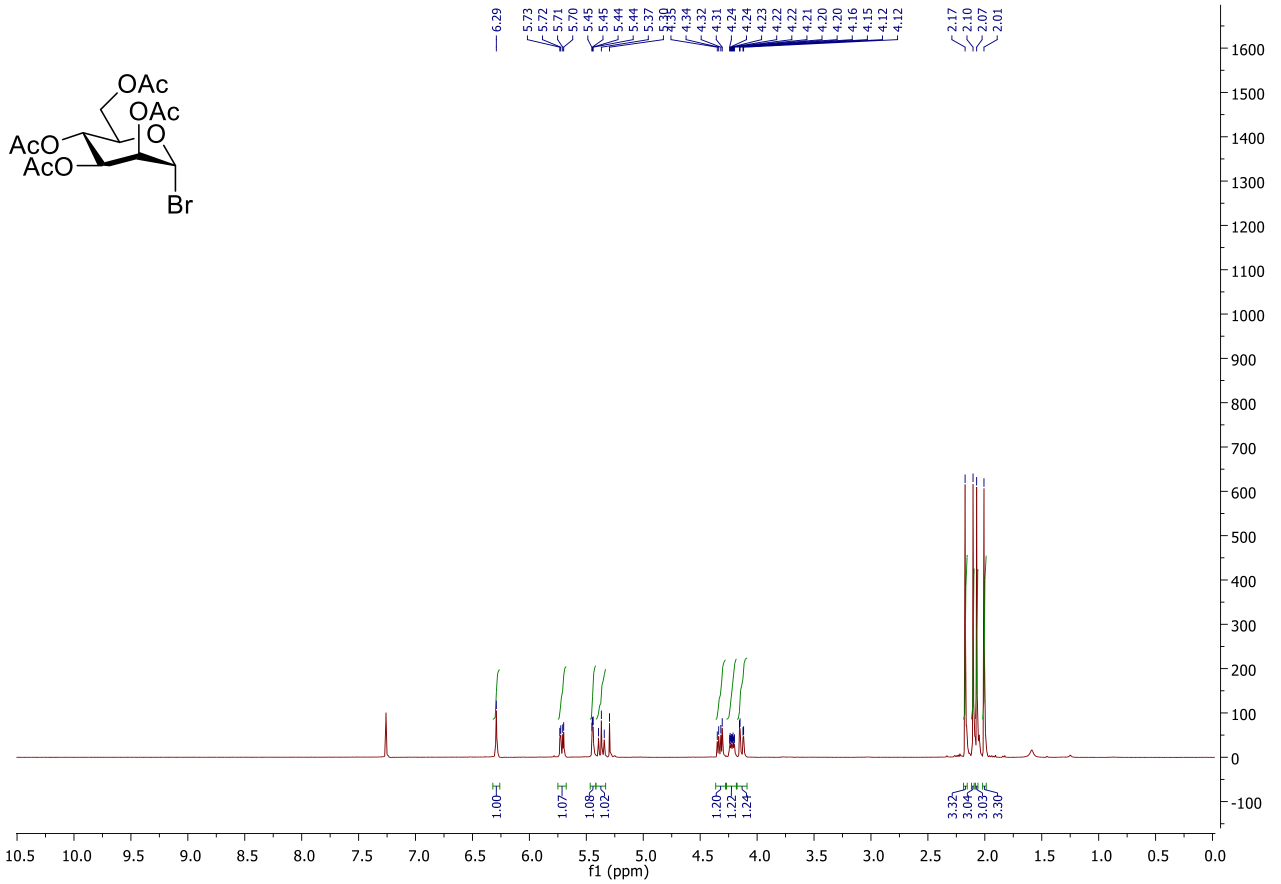

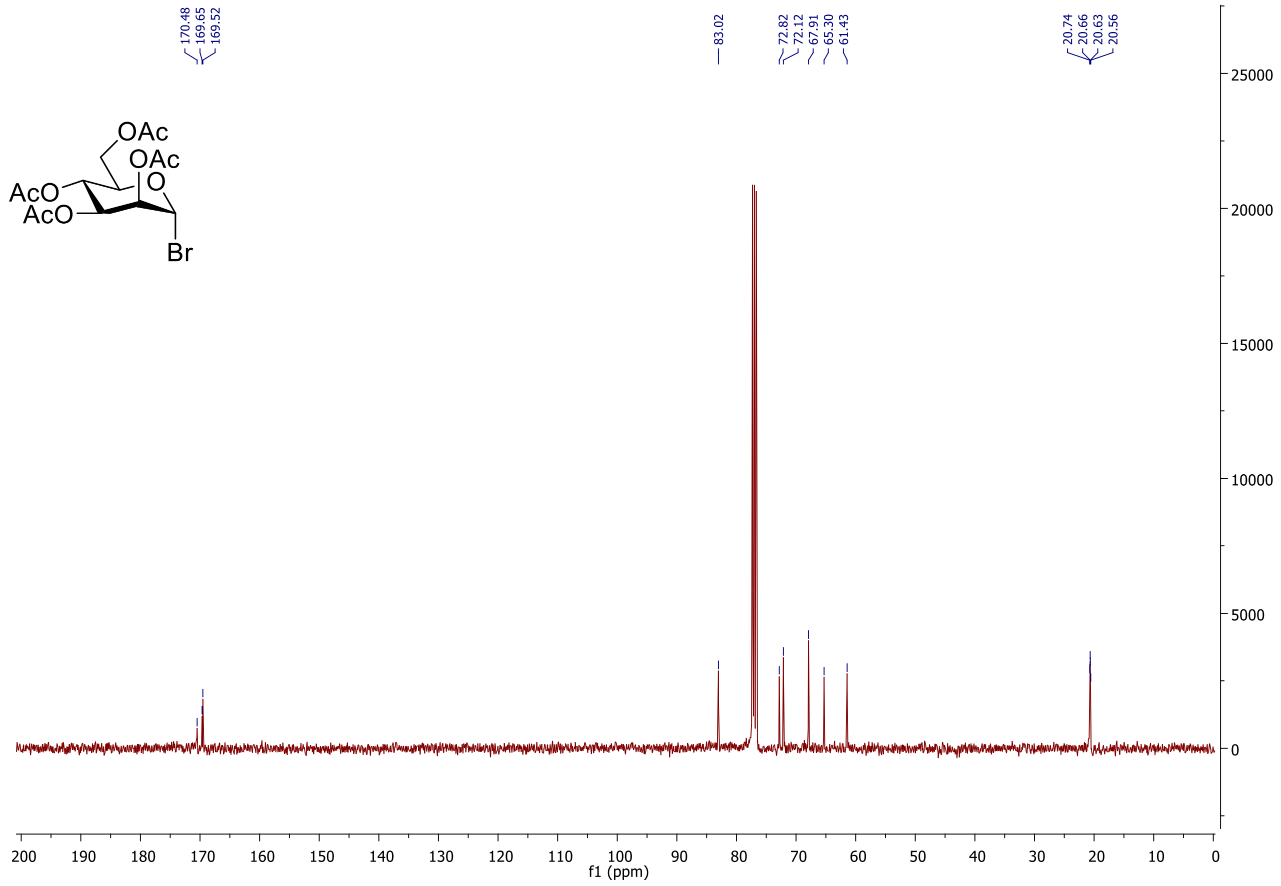

^{1}H-NMR (400 MHz),^{13}C{^{1}H}-NMR (101 MHz) of *2,3,4,6-Tetra-O-acetyl-α-galactopyranosyl bromide (1h) - (CDCl$_3$).*

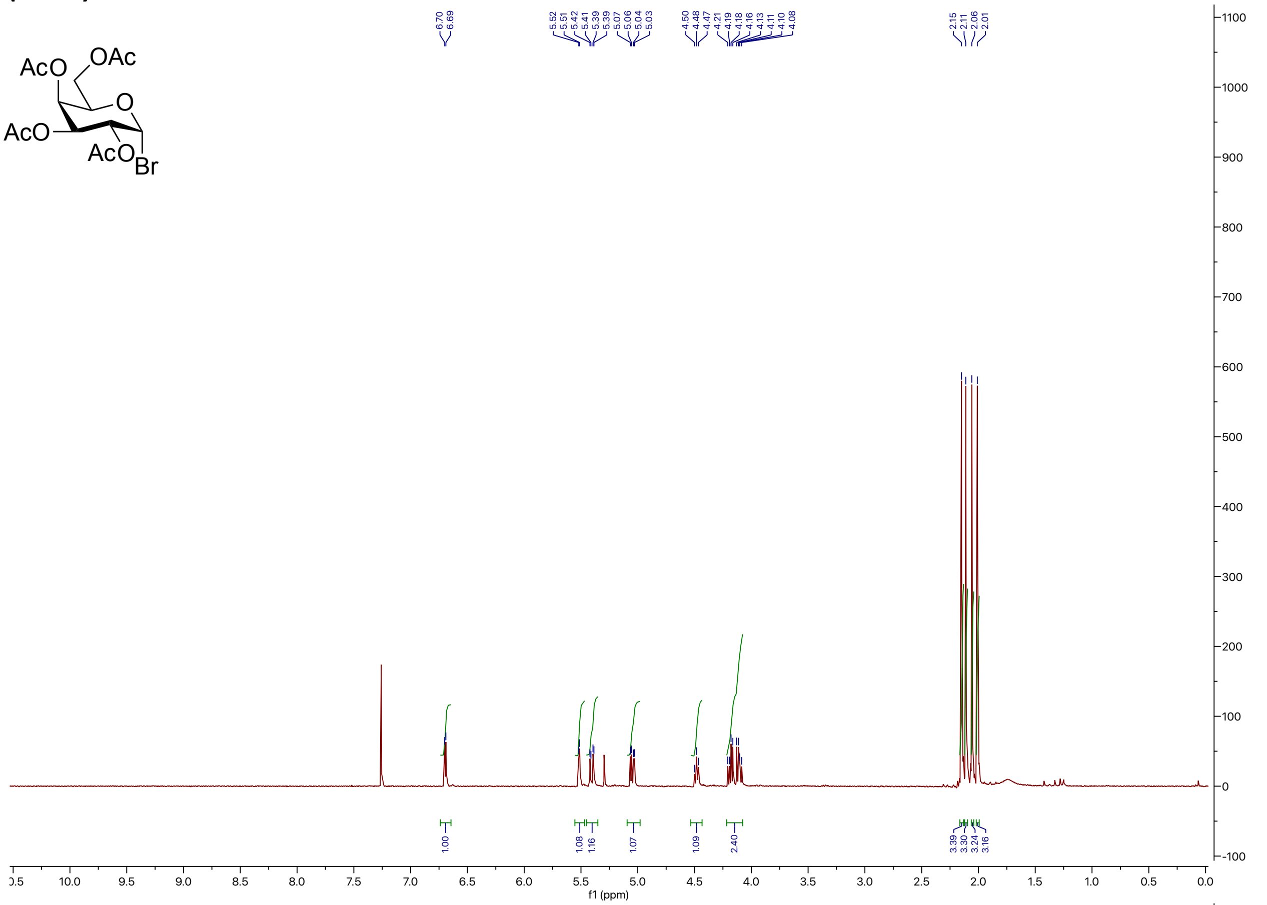

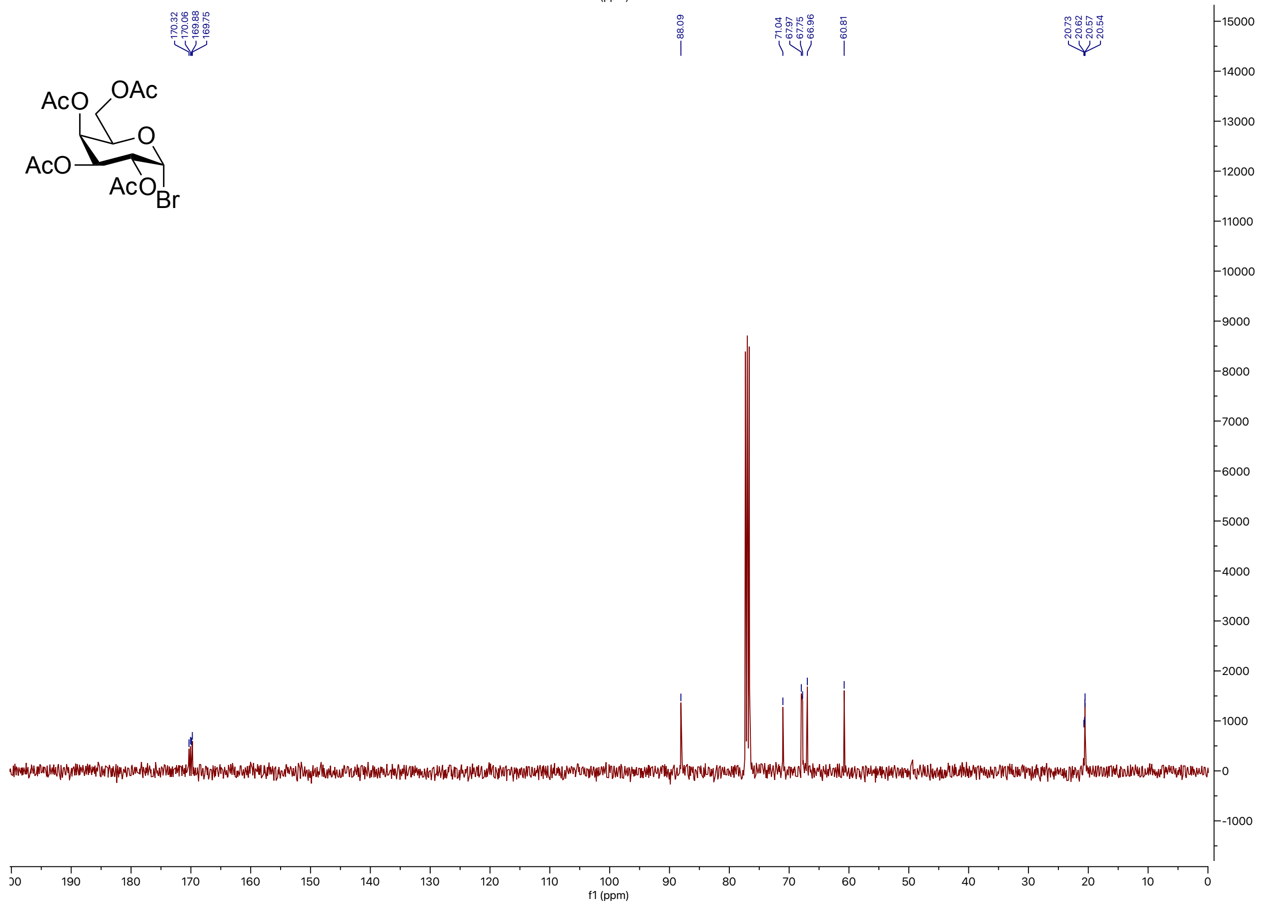

^{1}H-NMR (400 MHz),^{13}C{^{1}H}-NMR (101 MHz) of ***2,3:5,6-Di-O-isopropylidene-α/β-D-mannofuranose (8) - (CDCl$_3$).***

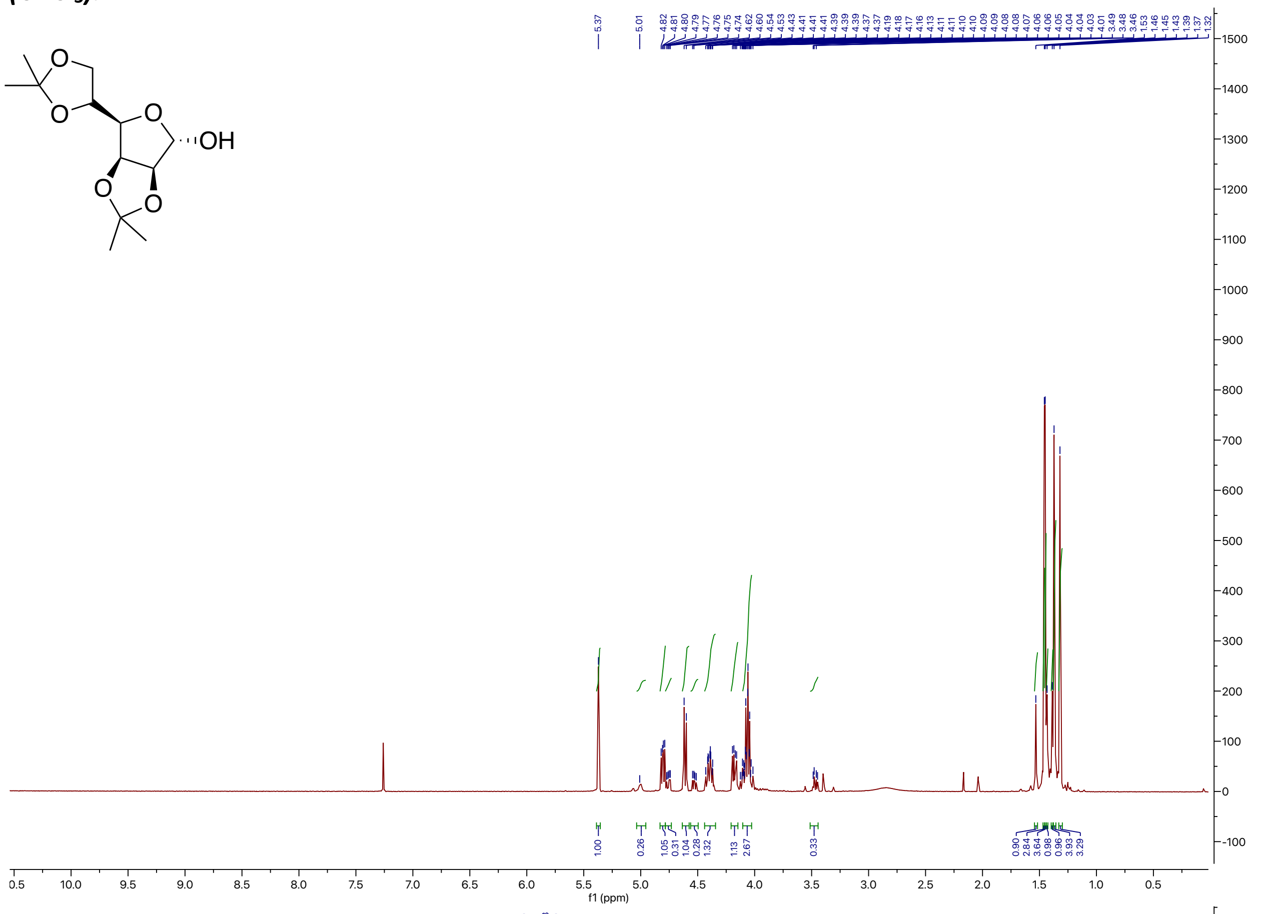

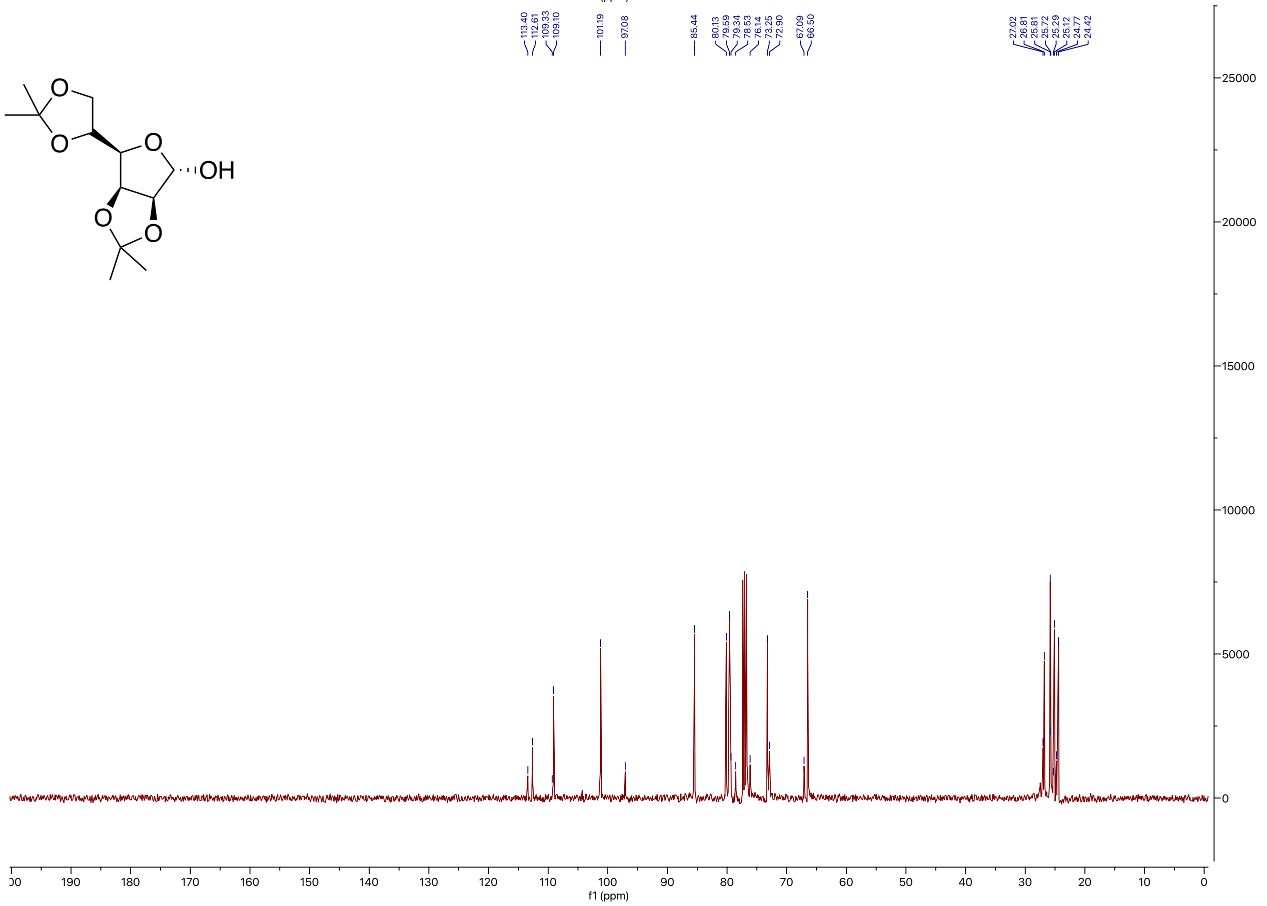

^{1}H-NMR (300 MHz),^{13}C{^{1}H}-NMR (101 MHz) of *1-O-Acetyl-2,3:5,6-Di-O-isopropylidene-α-D-mannofuranose (9) - (CDCl$_3$).*

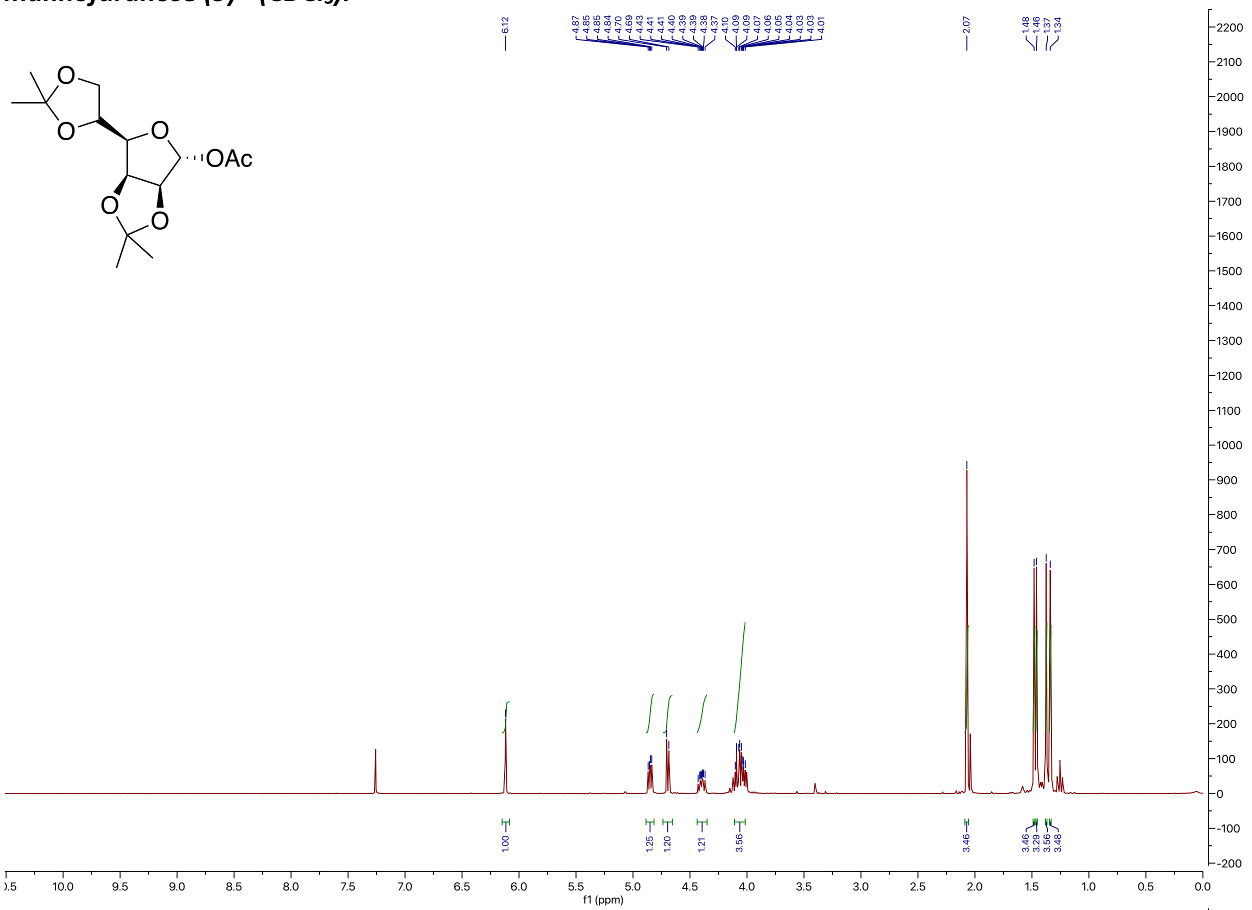

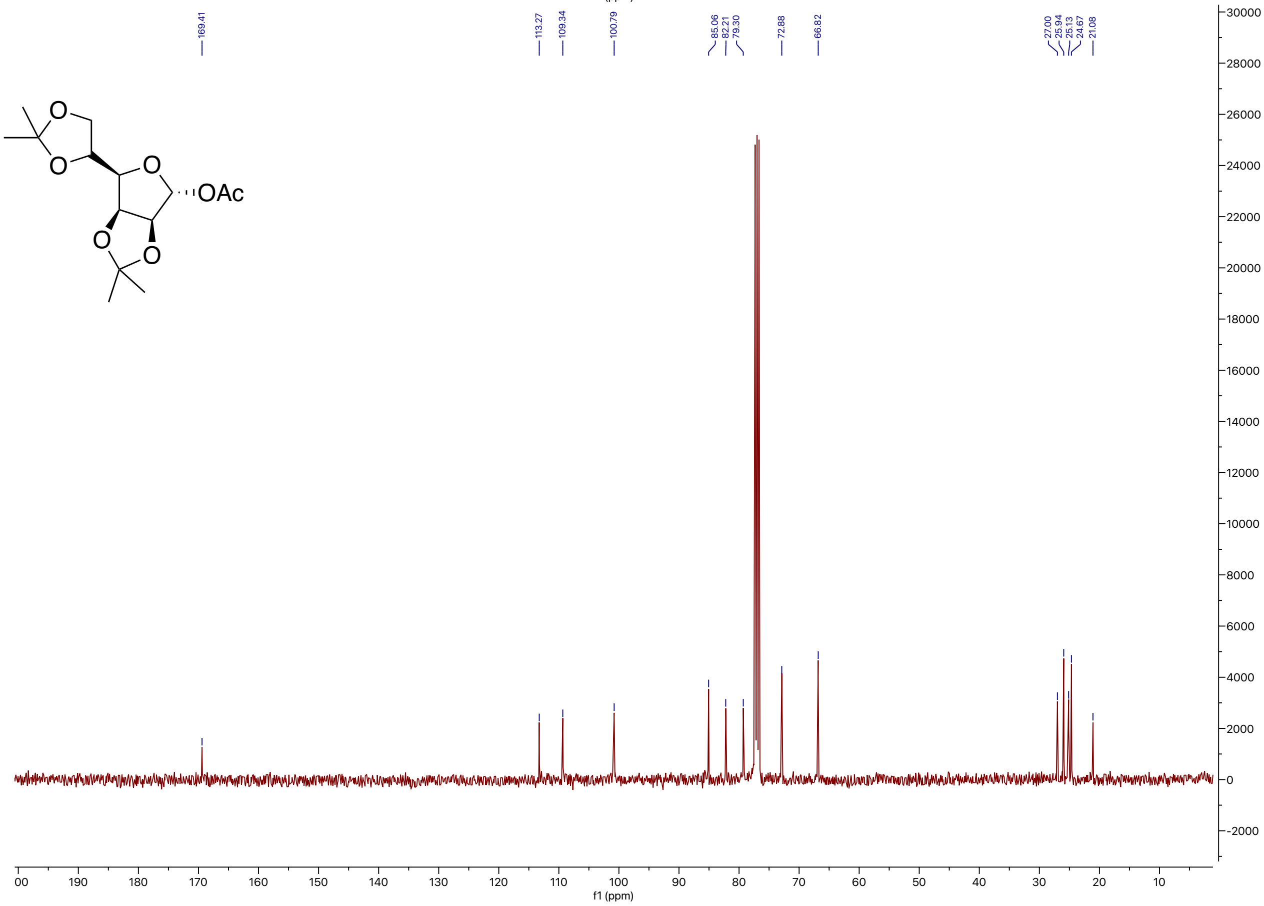

^{1}H-NMR (300 MHz),^{13}C{^{1}H}-NMR (101 MHz) of 2,*3:5,6-Di-O-isopropylidene-α-D-mannofuranosyl bromide (1e) - (CDCl$_3$).*

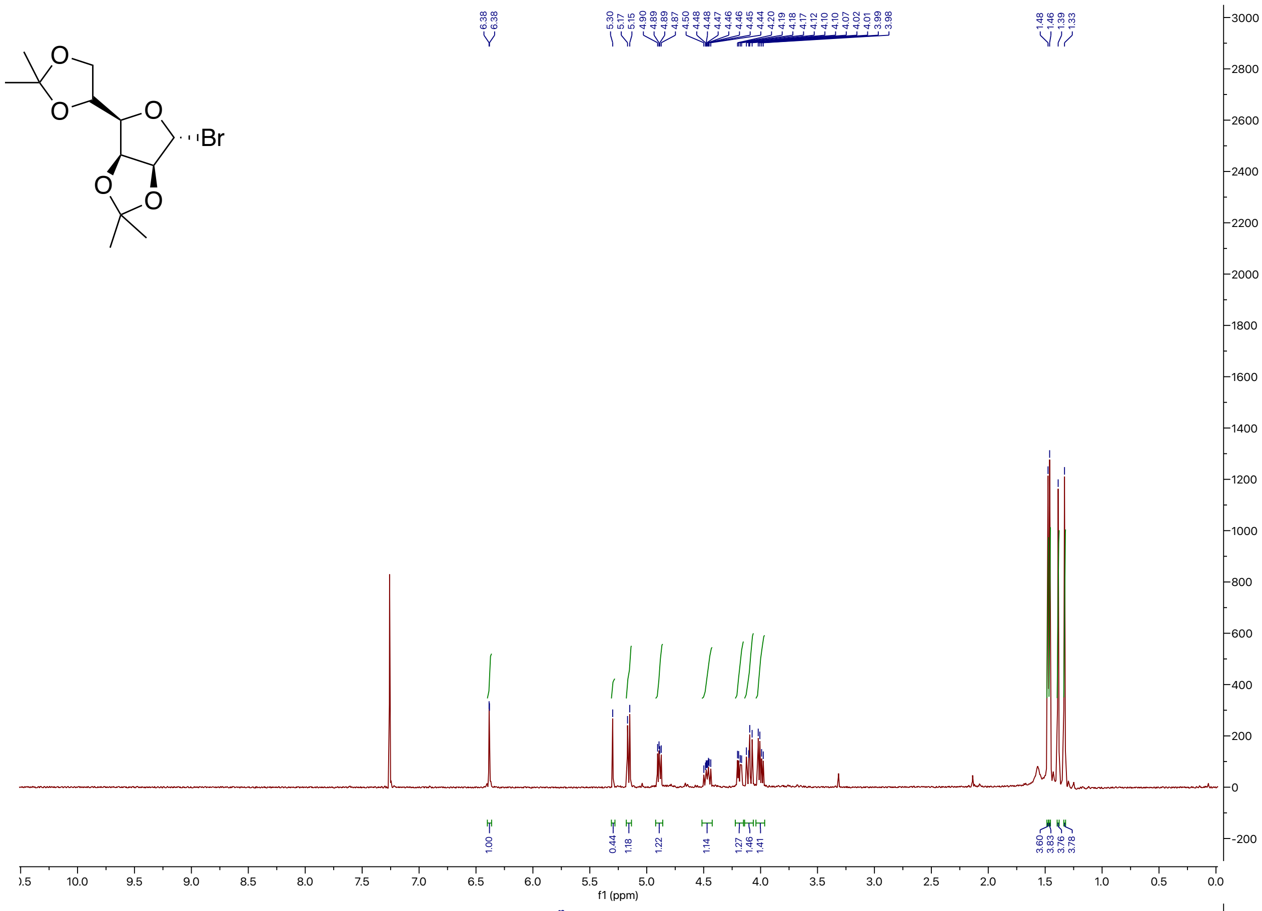

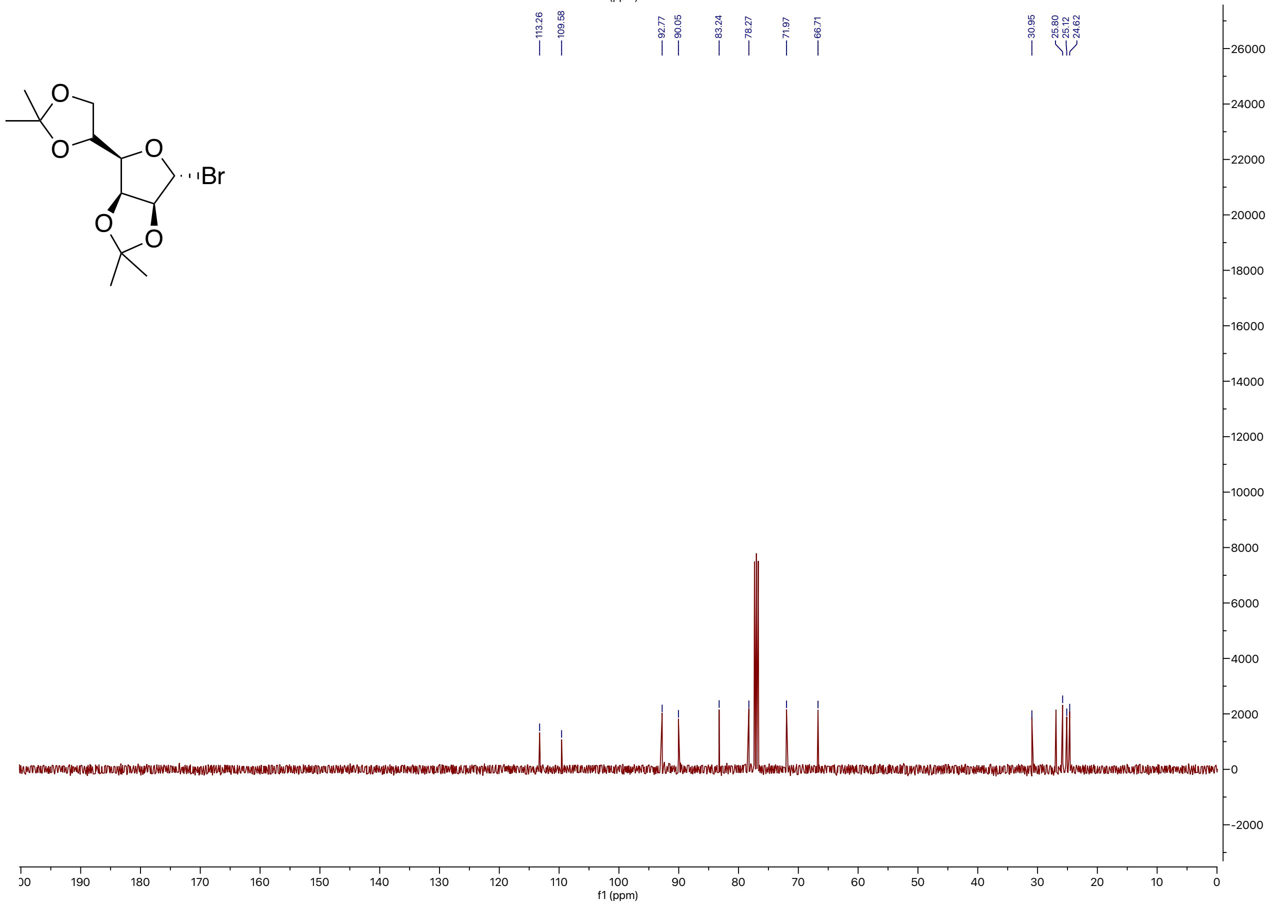

^{1}H-NMR (300 MHz),^{13}C{^{1}H}-NMR (101 MHz) of *2,3,4,6-Tetra-O-benzoyl-β-D-galactopyranosyl)-(1→4)-1,2,3,6-Tetra-O-benzoyl-α-D-glucopyranose (10) - (CDCl$_3$).*

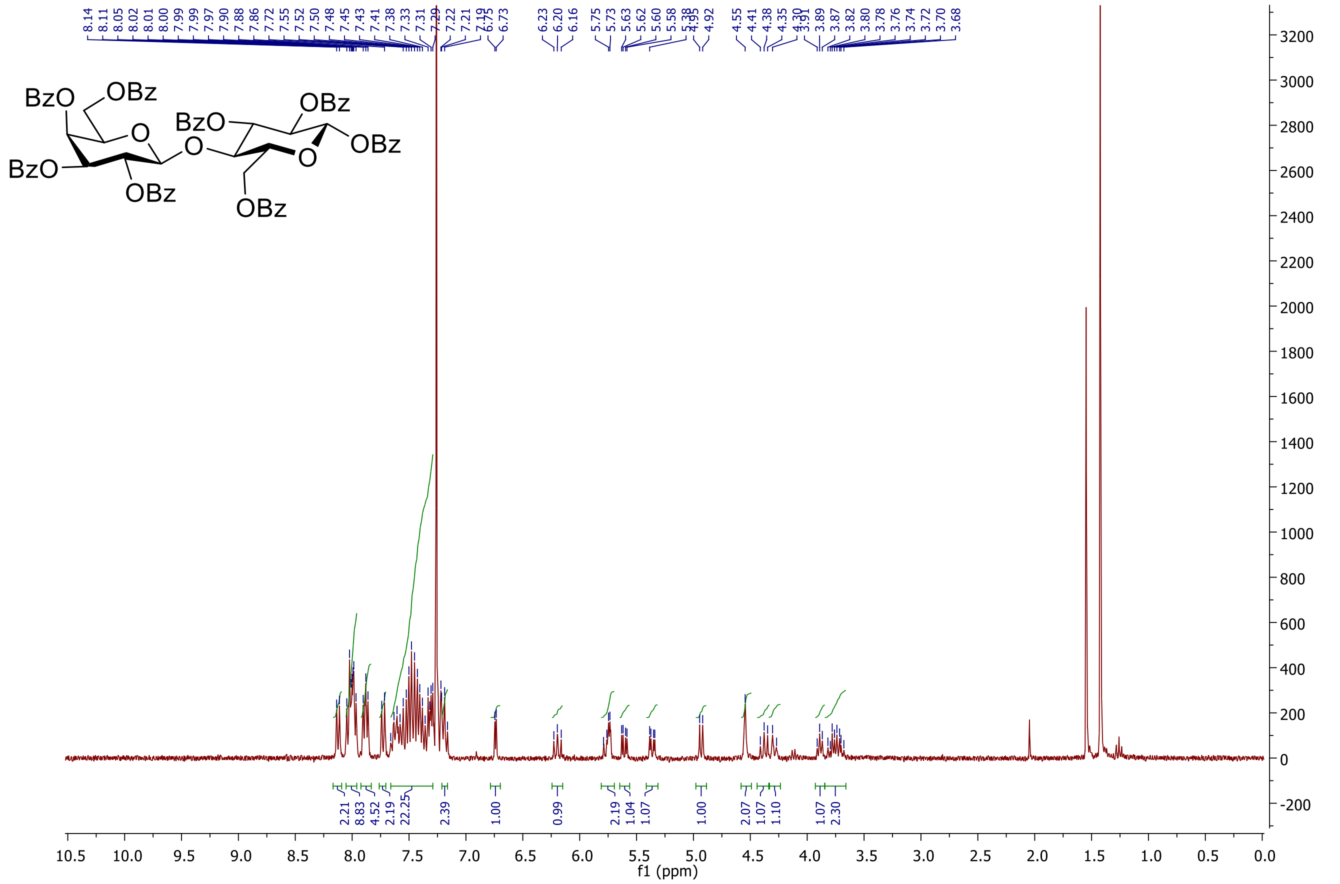

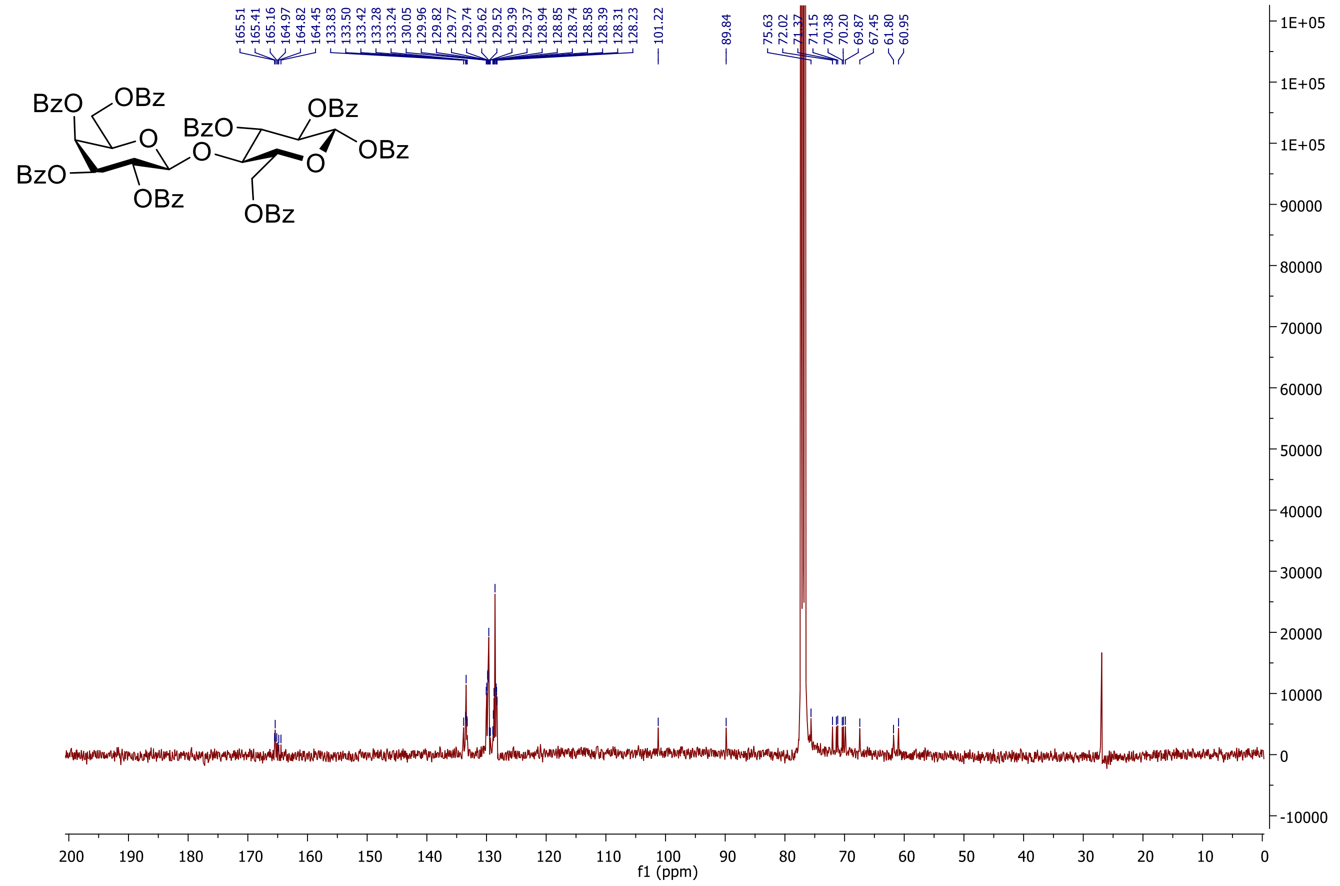

^{1}H-NMR (300 MHz),^{13}C{^{1}H}-NMR (101 MHz) of *2,3,4,6-Tetra-O-benzoyl-β-D-galactopyranosyl)-(1→4)-2,3,6-tri-O-benzoyl-α-D-glucopyranosyl bromide (1d) - (CDCl$_3$).*

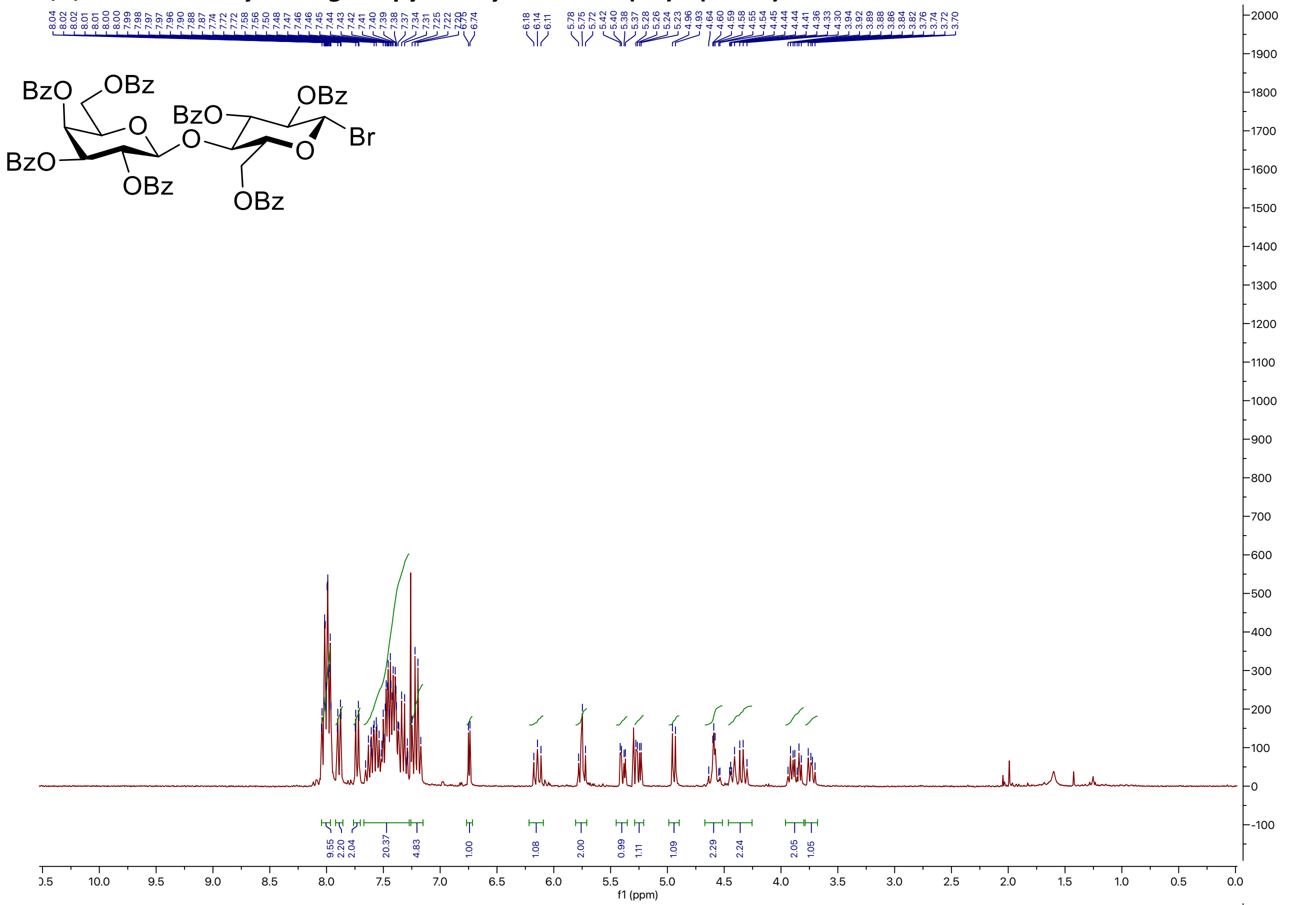

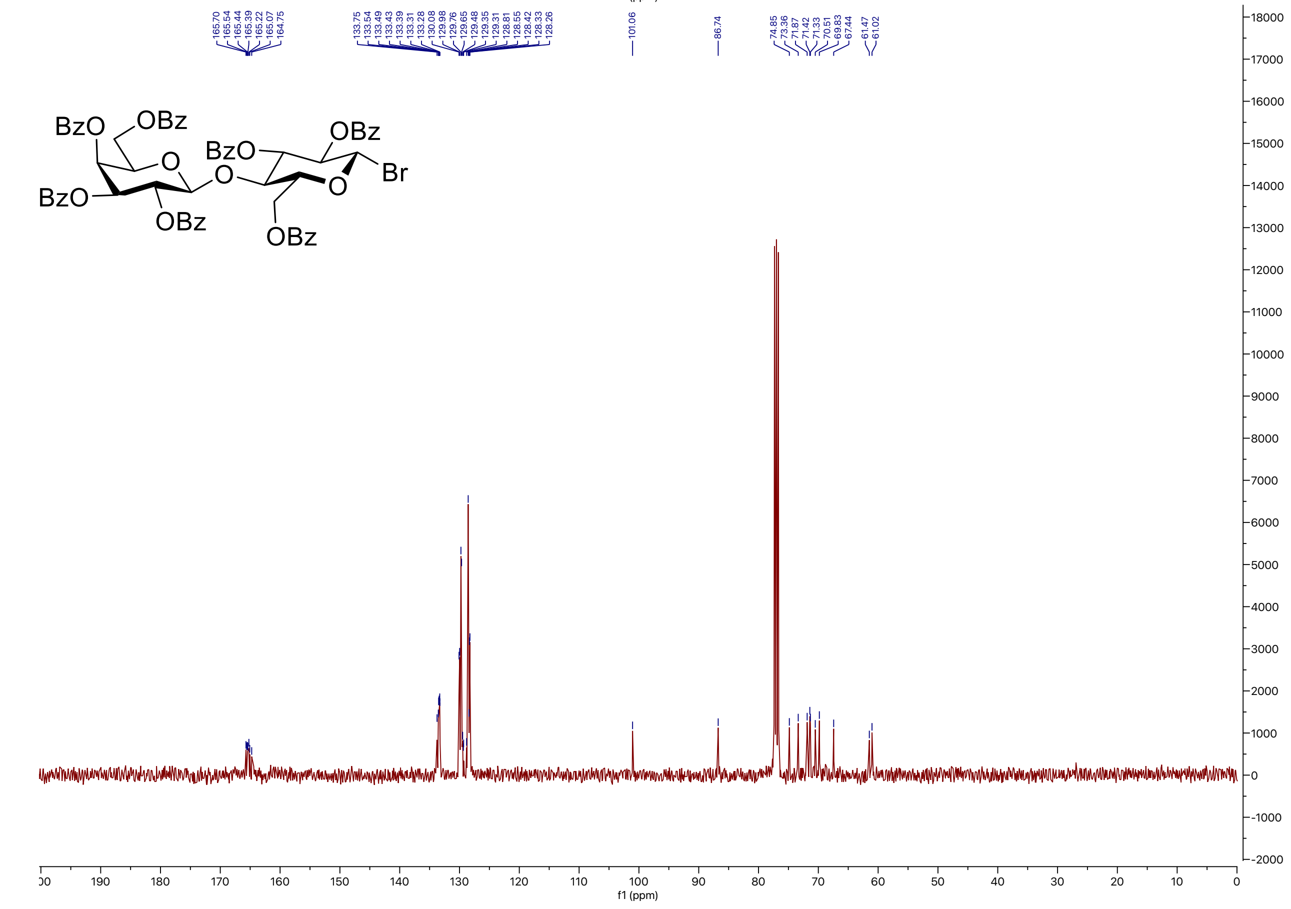

^{1}H-NMR (400 MHZ),^{13}C{^{1}H}-NMR (101 MHz) of *2,3,4,6-Tetra-O-benzoyl-β-D-glucopyranosyl-(1→4)-(1,2,3,6-tetra-O-benzoyl)-β-D-glucopyranose (11) - (CDCl$_3$).*

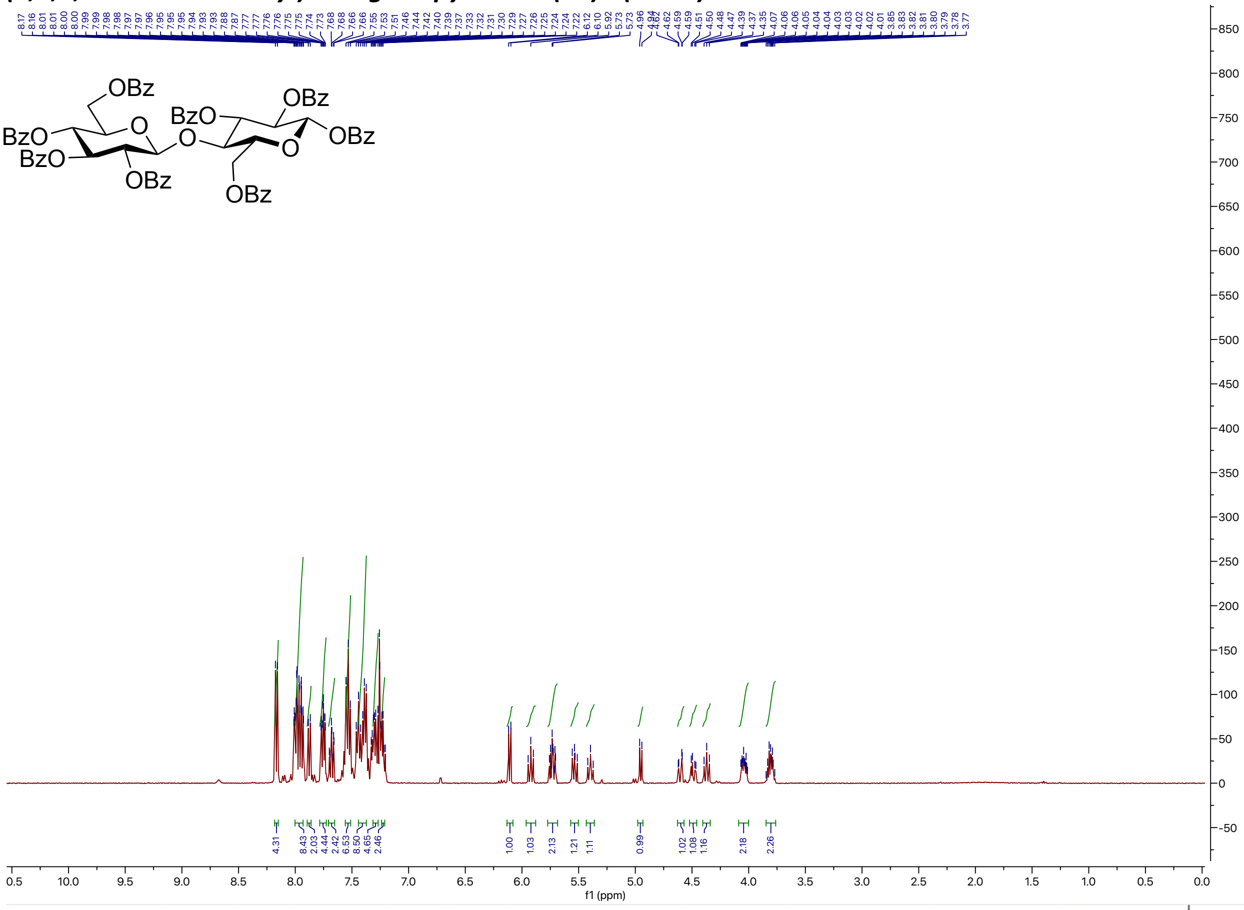

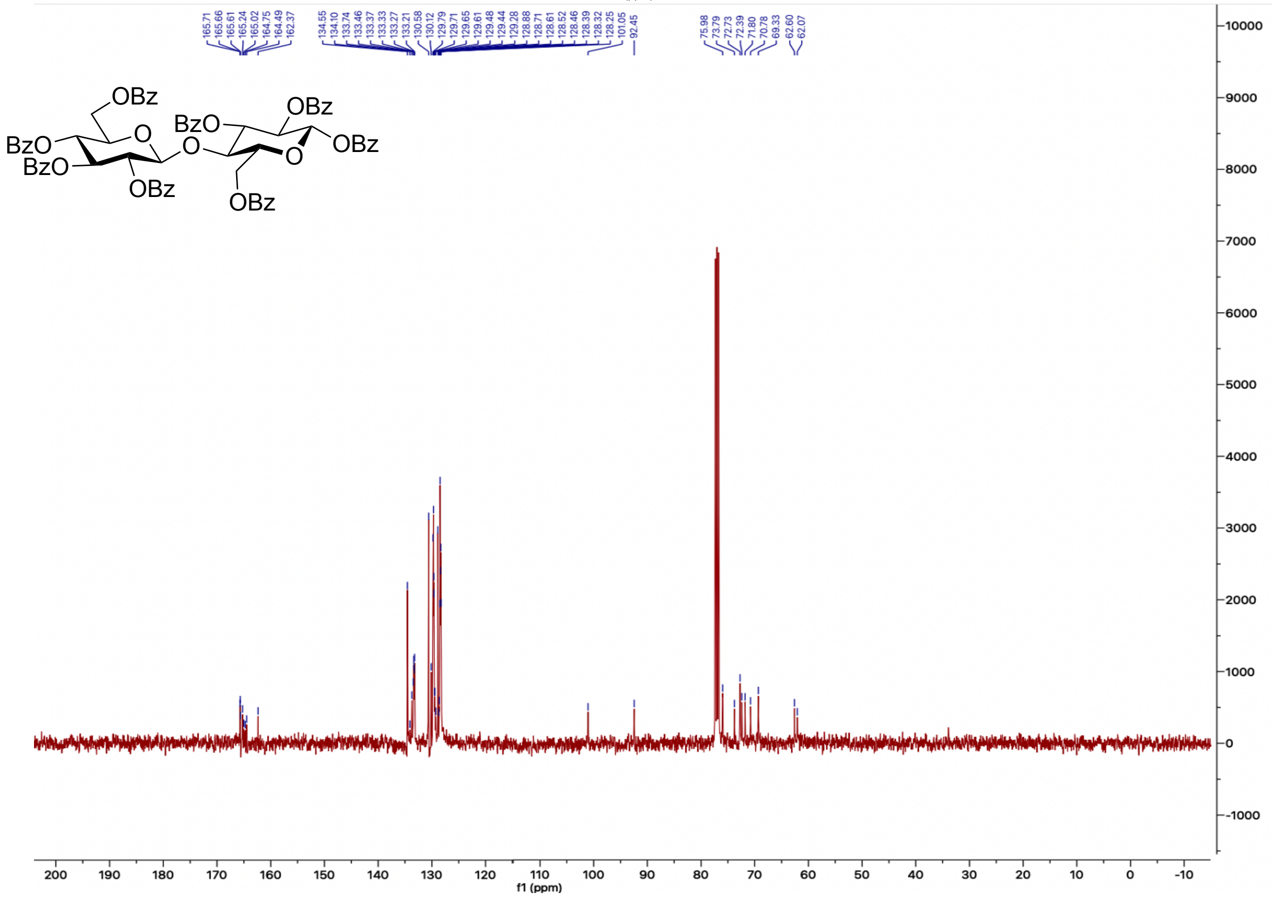

^{1}H-NMR (400 MHZ),^{13}C{^{1}H}-NMR (101 MHz) of *2,3,4,6-Tetra-O-benzoyl-β-D-glucopyranosyl-(1→4)-2,3,6-tri-O-benzoyl-α-D-glucopyranosyl bromide (1i) - (CDCl$_3$).*

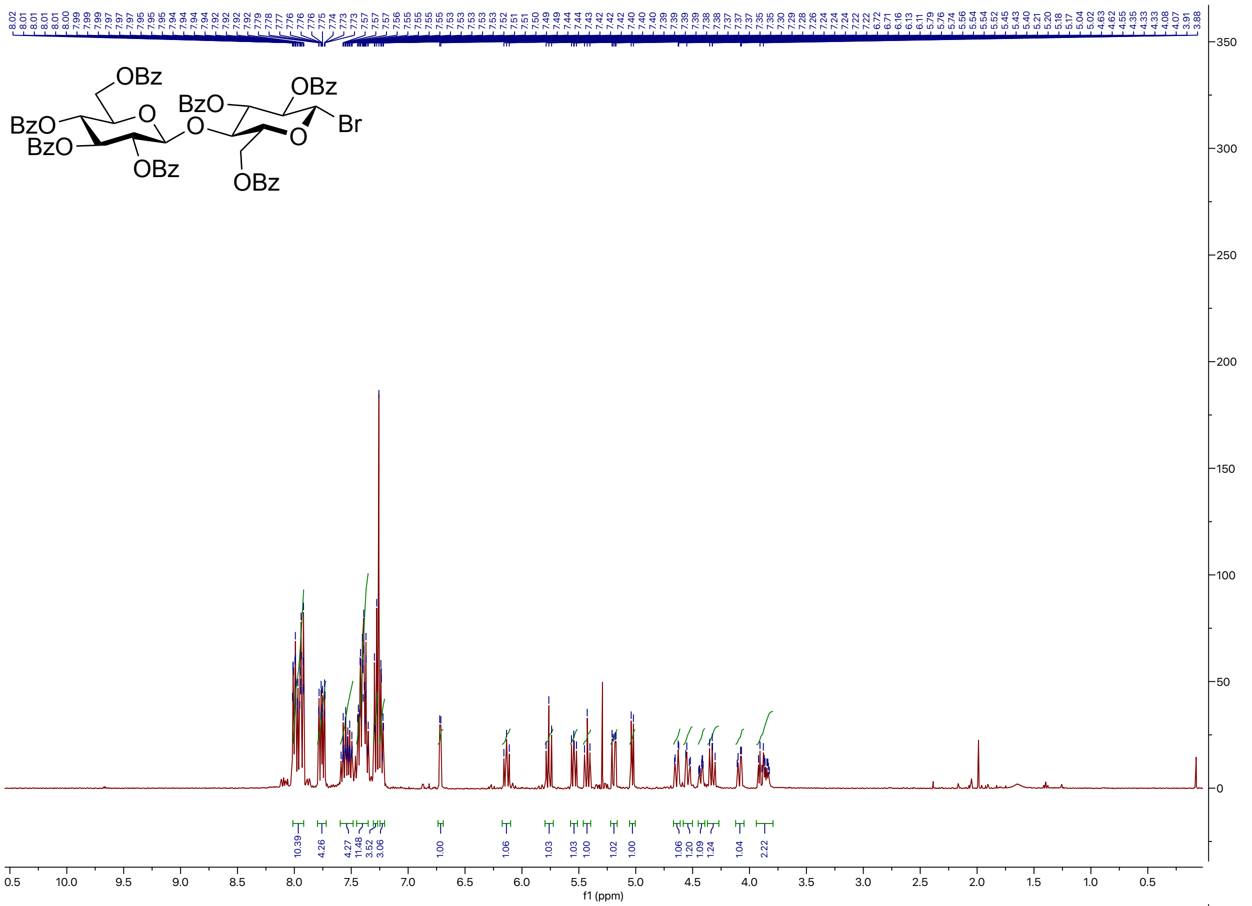

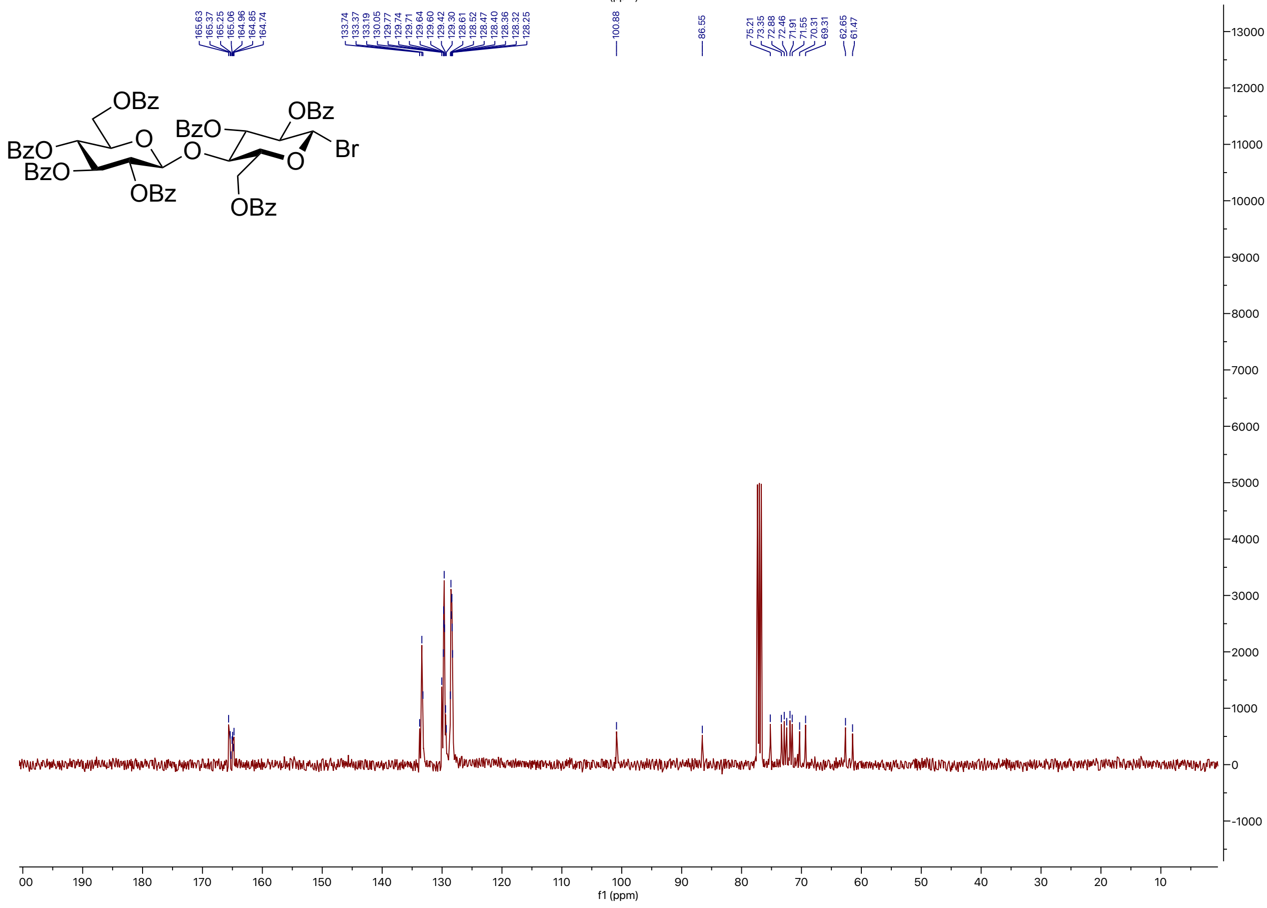

^{1}H-NMR (300 MHz),^{13}C{^{1}H}-NMR (101 MHz) of *methyl (tert-butoxycarbonyl)-L-serinate (12) - (CDCl$_3$).*

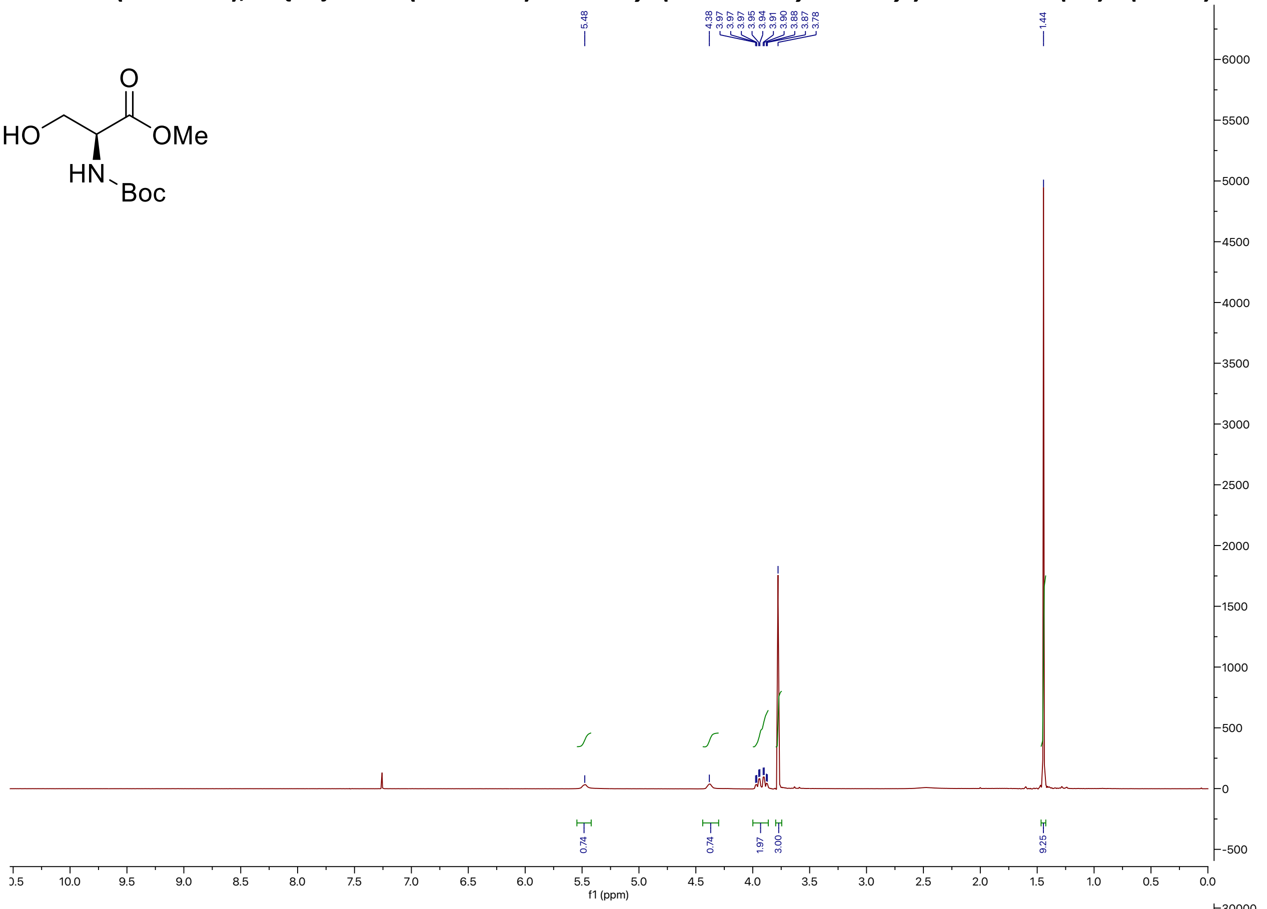

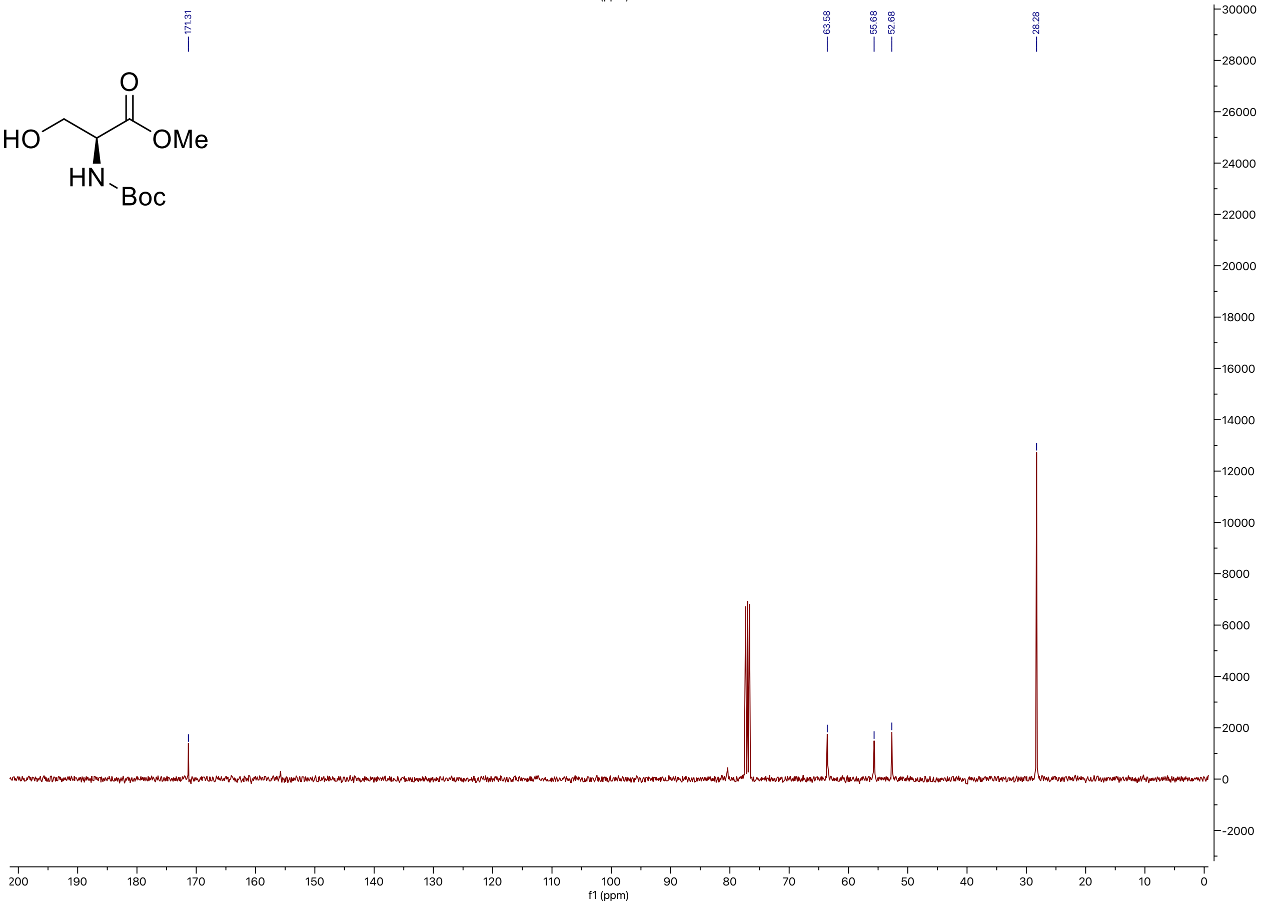

^{1}H-NMR (300 MHz),^{13}C{^{1}H}-NMR (101 MHz) of *Methyl-2-(di(tert-butoxycarbonyl)amino)but-2-enoate (2a) - (CDCl$_3$).*

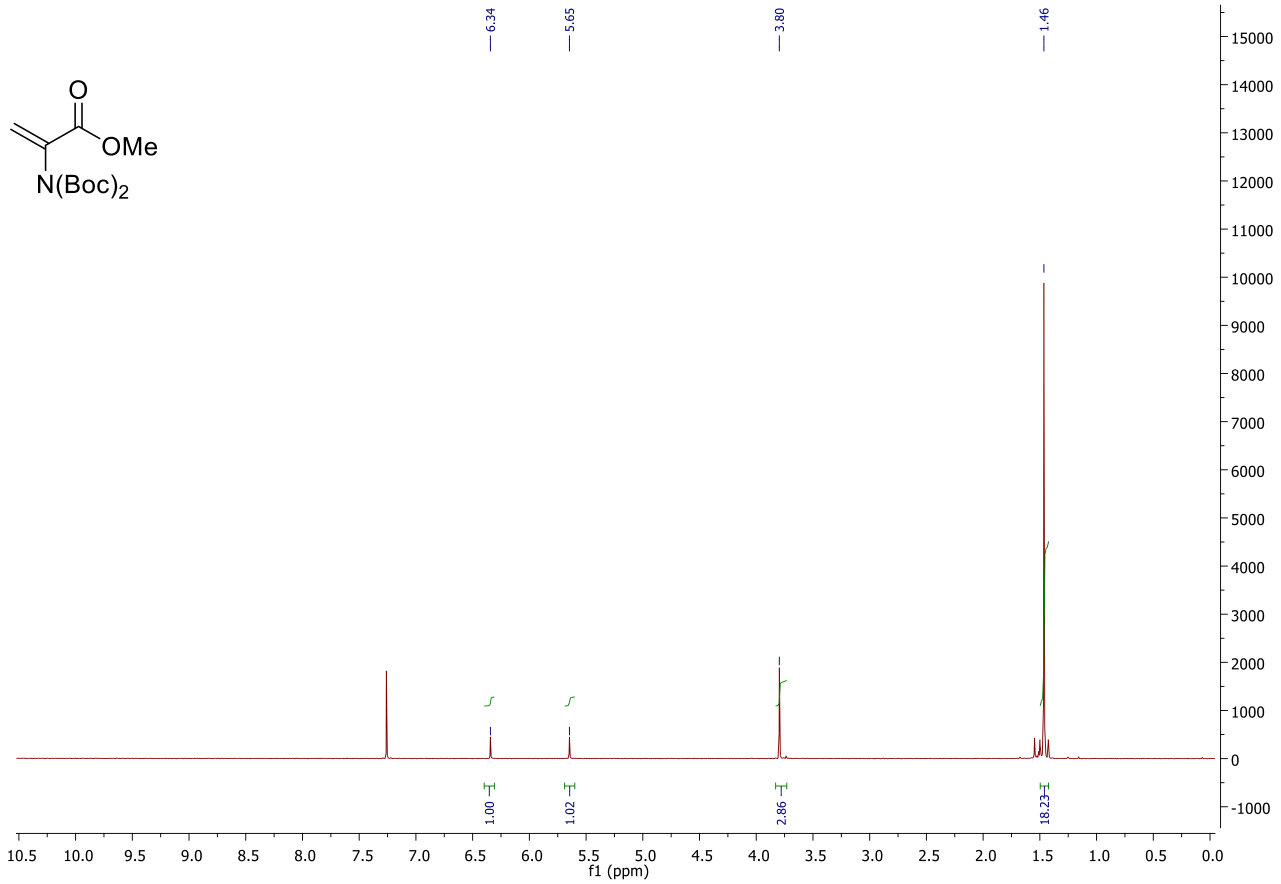

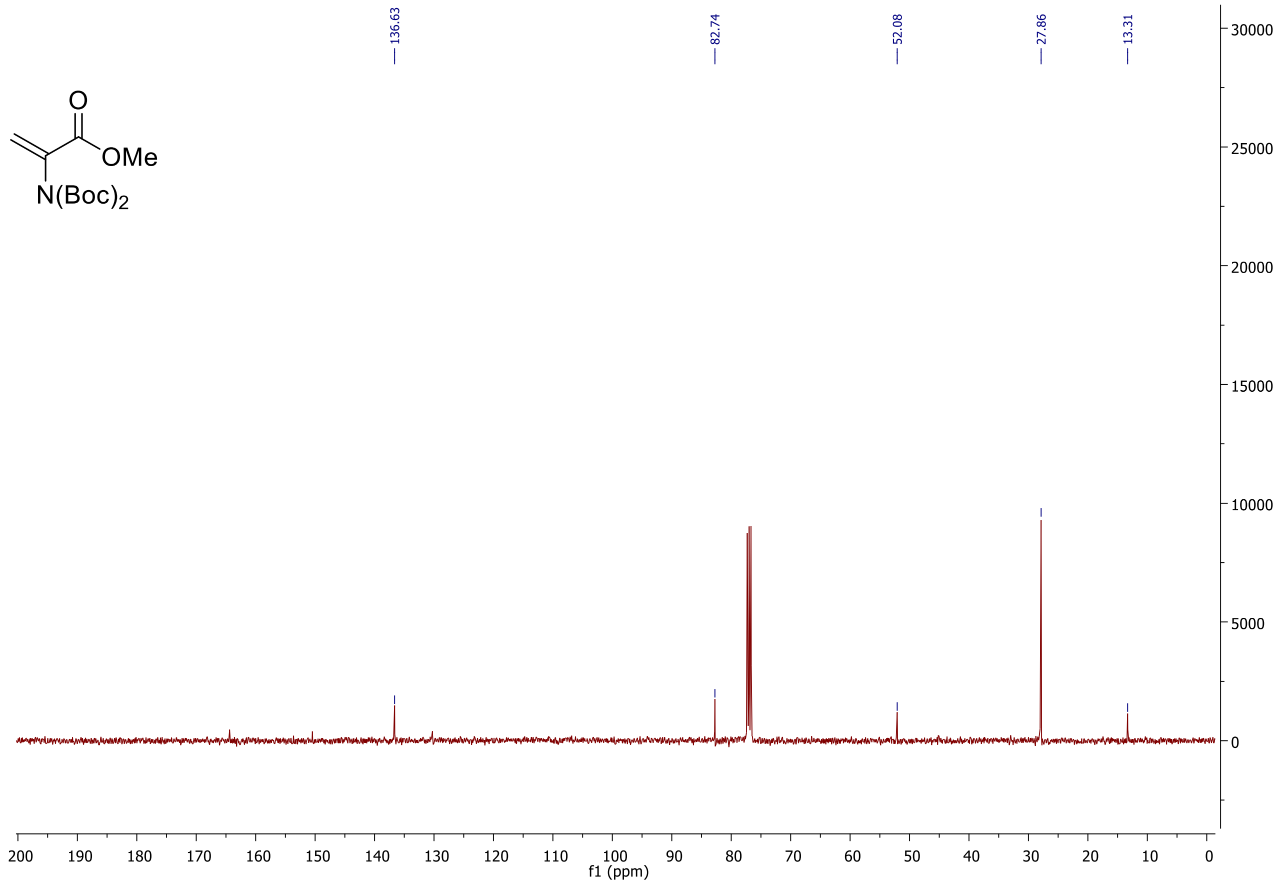

^{1}H-NMR (400 MHz),^{13}C{^{1}H}-NMR (101 MHz) of *Methyl (2S)-2-((tert-butoxycarbonyl)amino)-3-hydroxybutanoate (13) - (CDCl$_3$).*

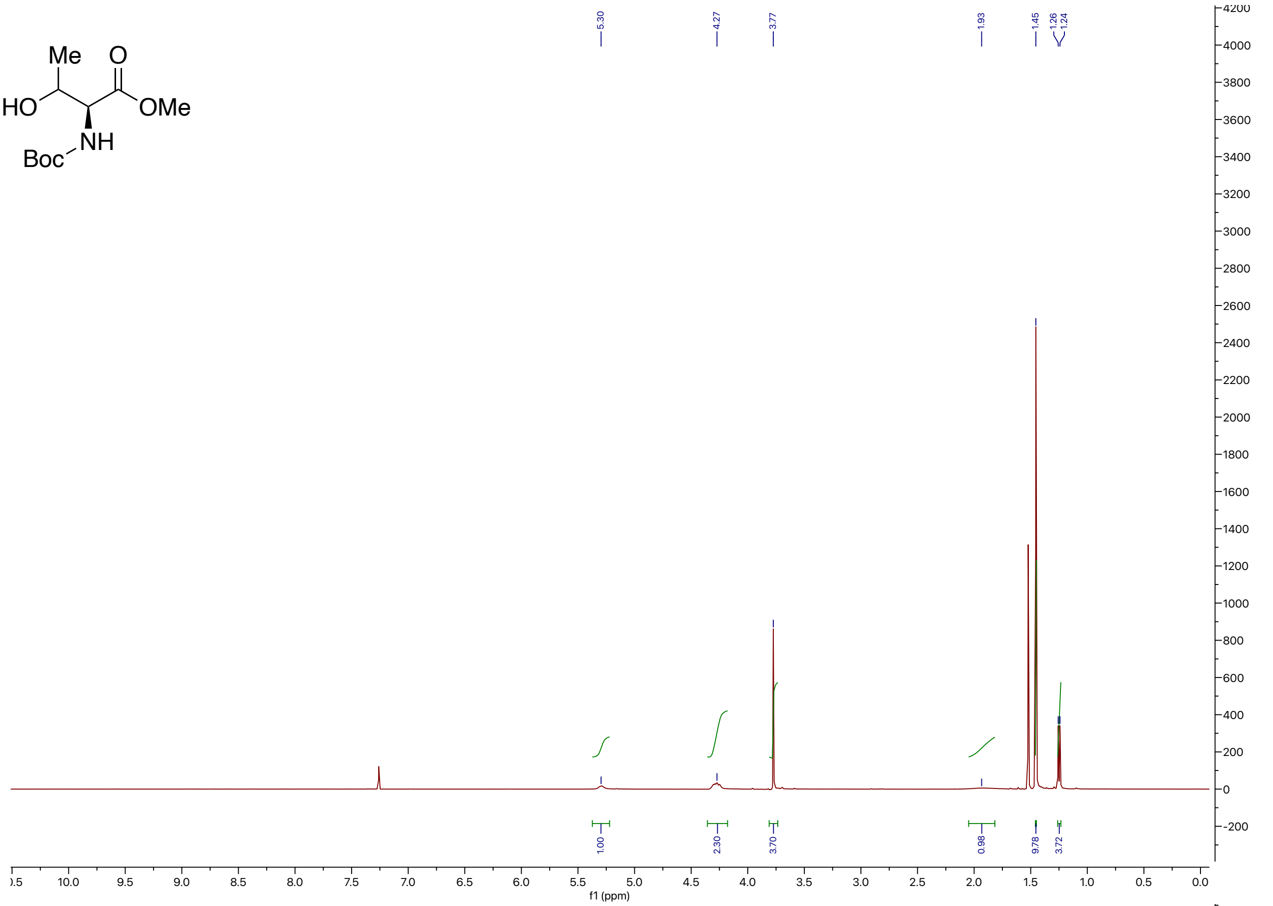

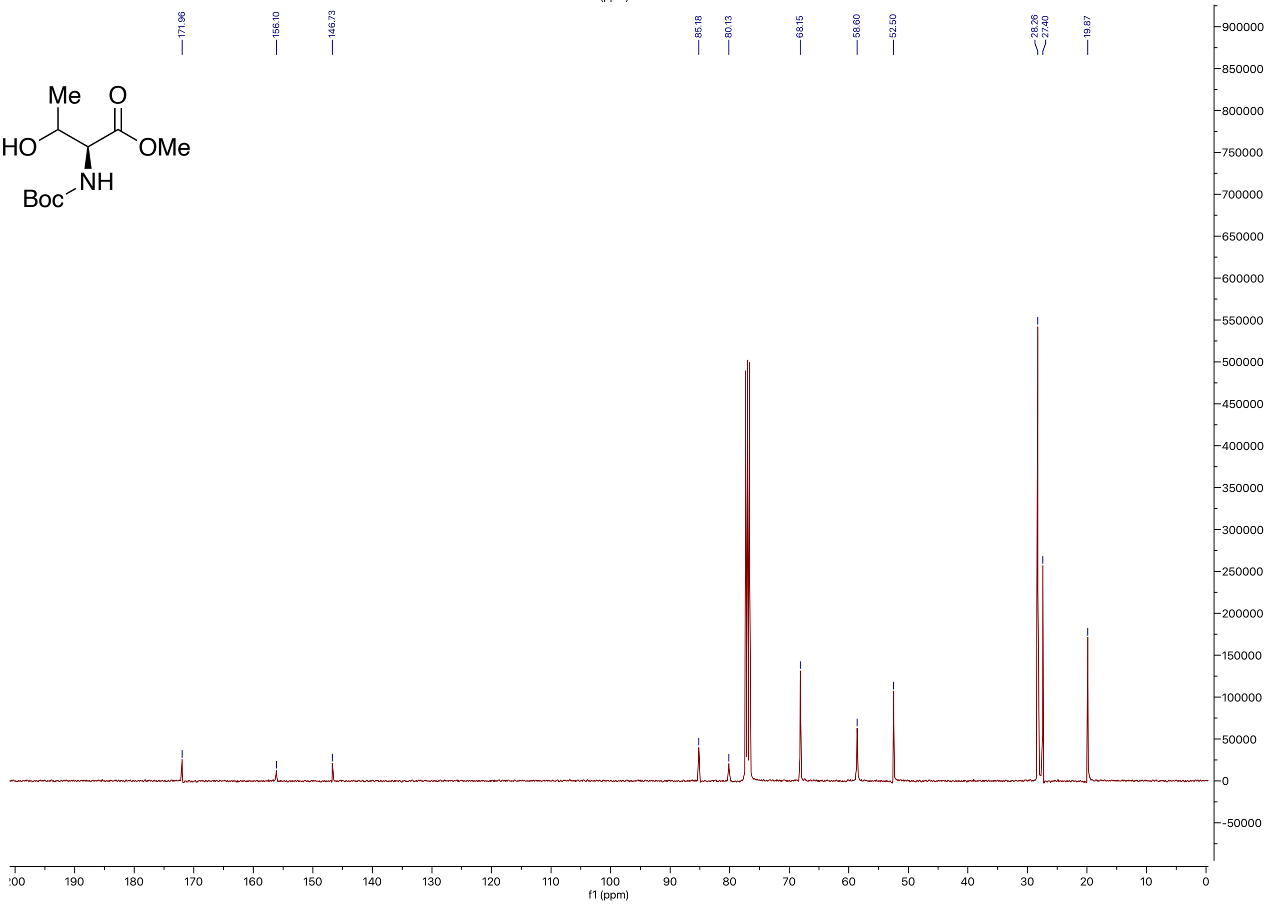

^{1}H-NMR (300 MHz),^{13}C{^{1}H}-NMR (101 MHz) of *Methyl-2-(di(tert-butoxycarbonyl)amino)but-2-enoate (2b)* - *(CDCl$_3$).*

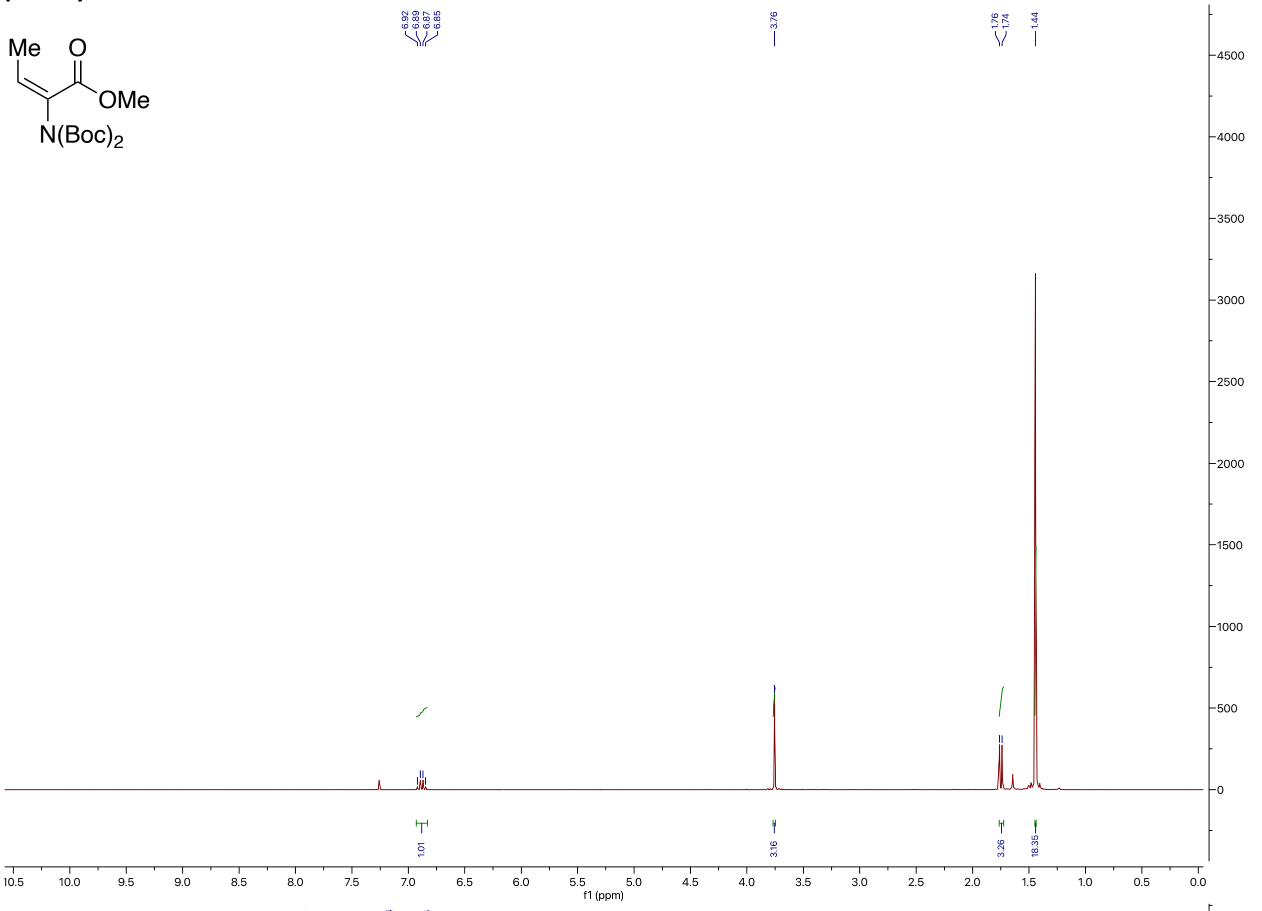

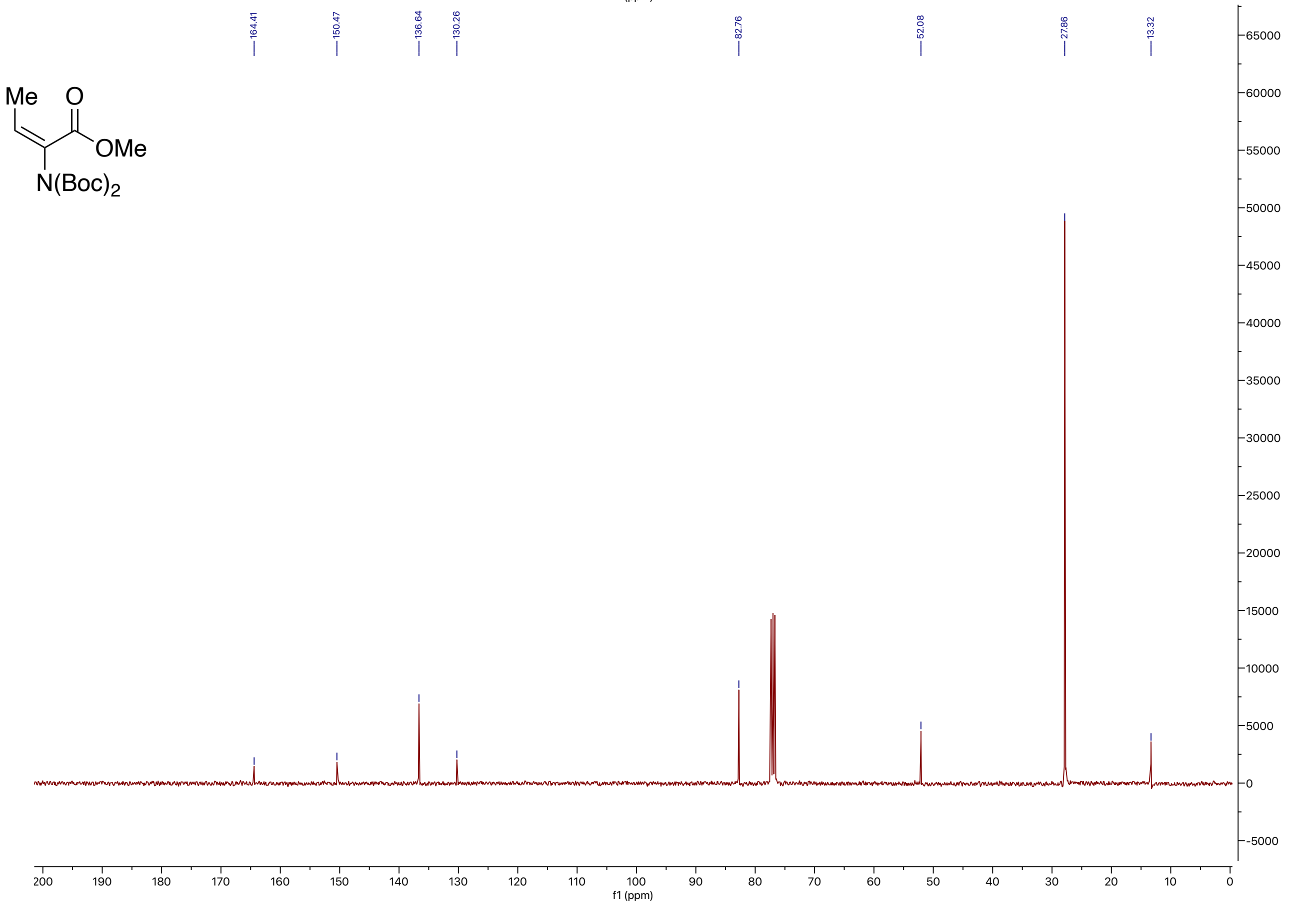

^{1}H-NMR (300 MHz),^{13}C{^{1}H}-NMR (101 MHz) of *Methyl 2-(phenylamino)but-2-enoate (2c) - (CDCl$_3$).*

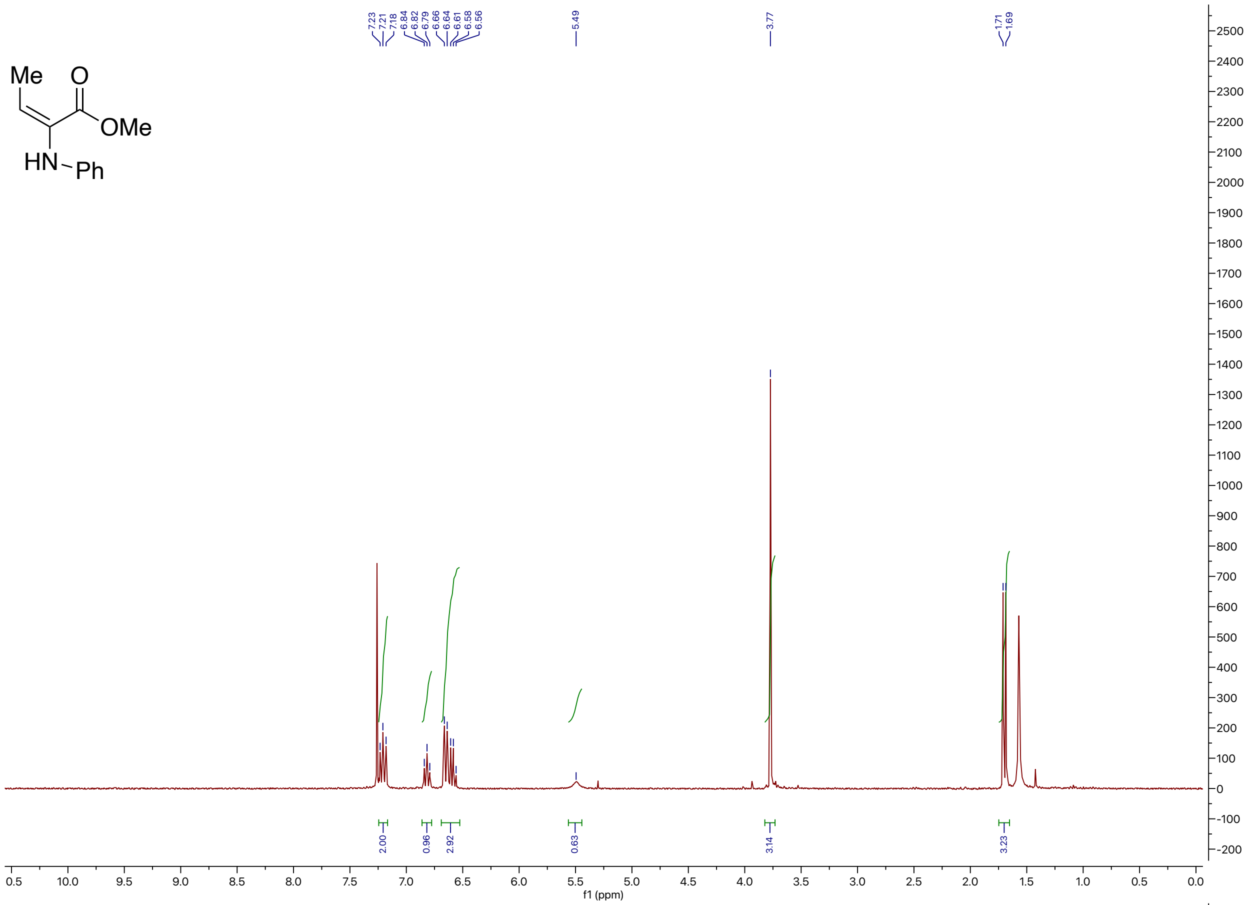

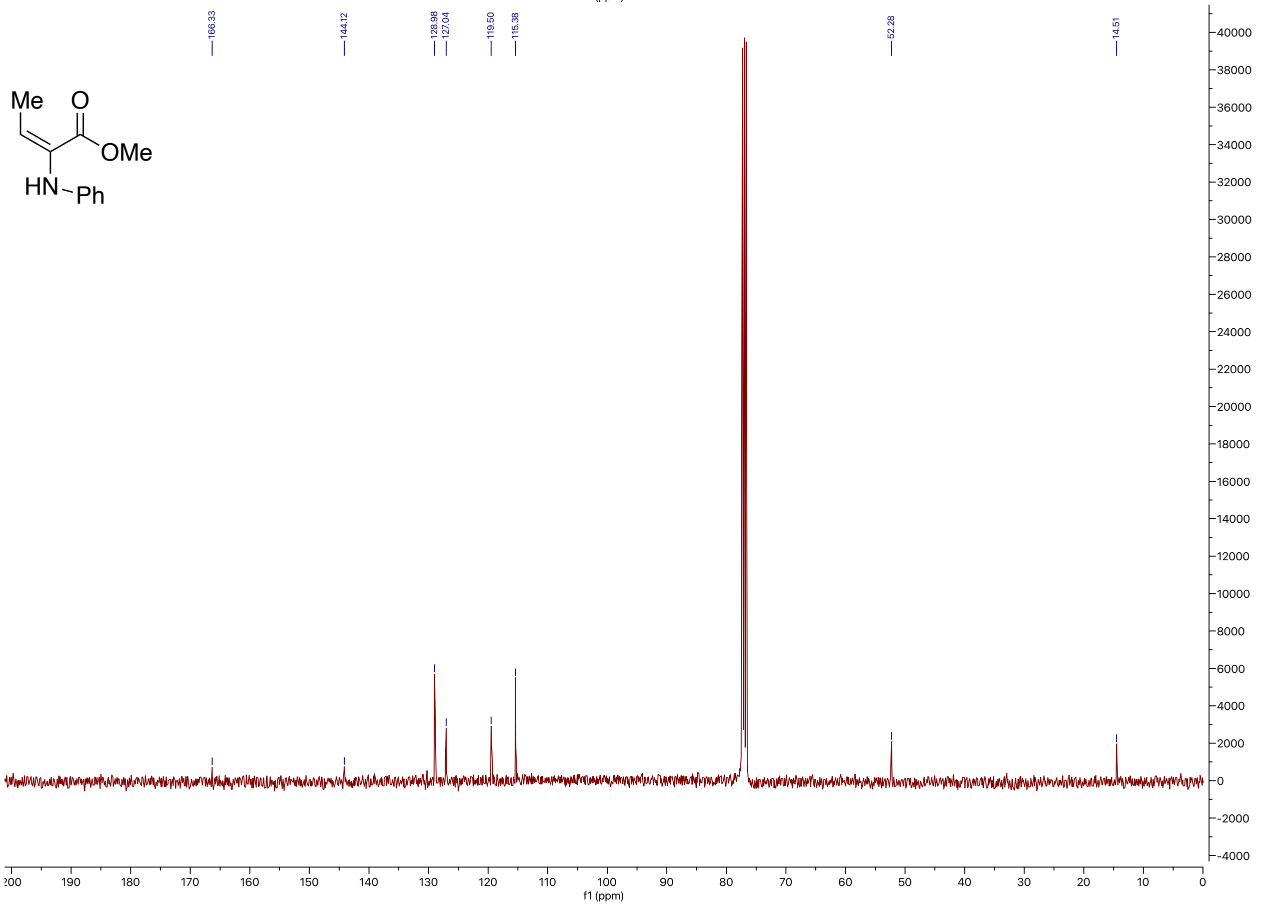

^{1}H-NMR (300 MHz),^{13}C{^{1}H}-NMR (101 MHz) of *2-aminoacrylate (2e) - ($CDCl_3$).*

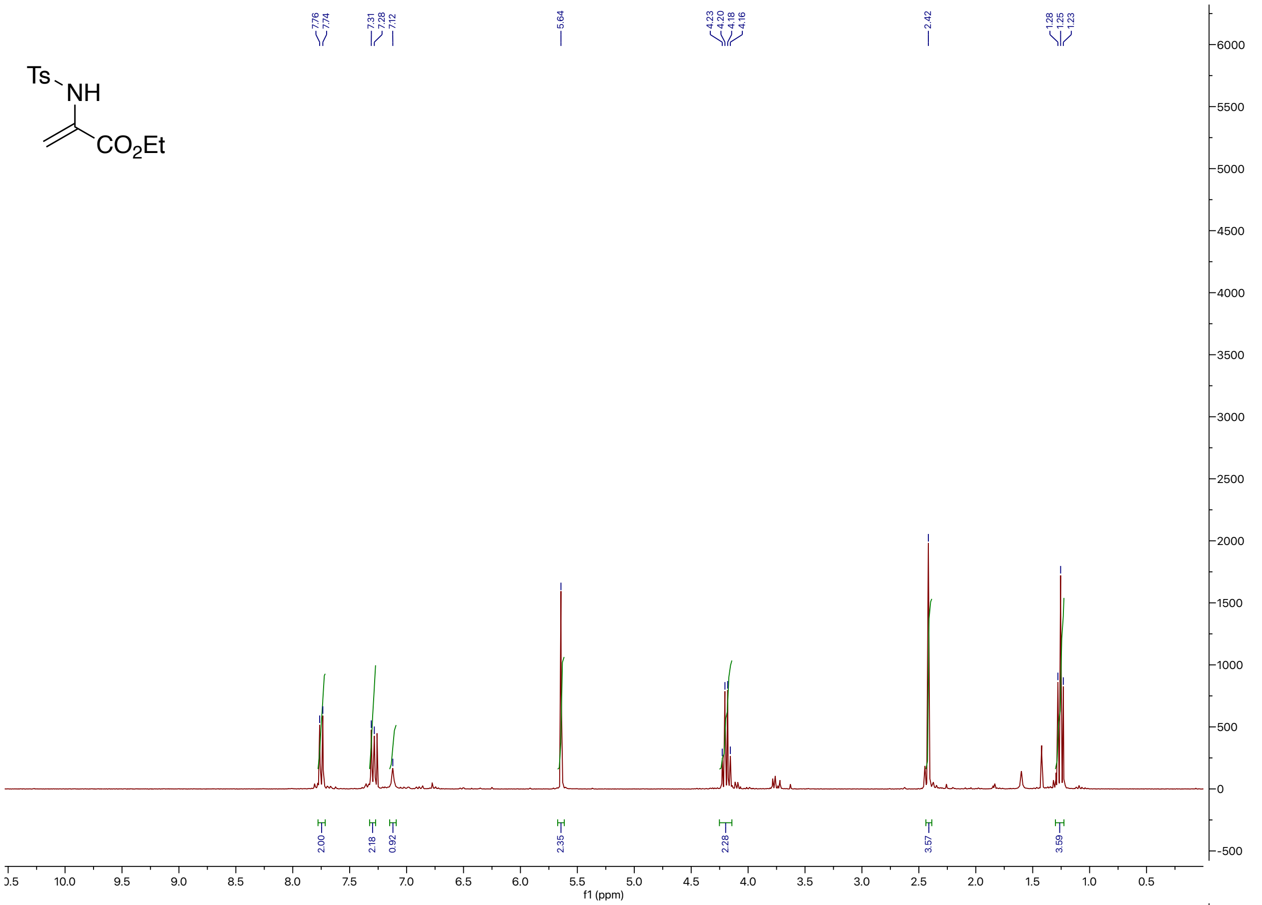

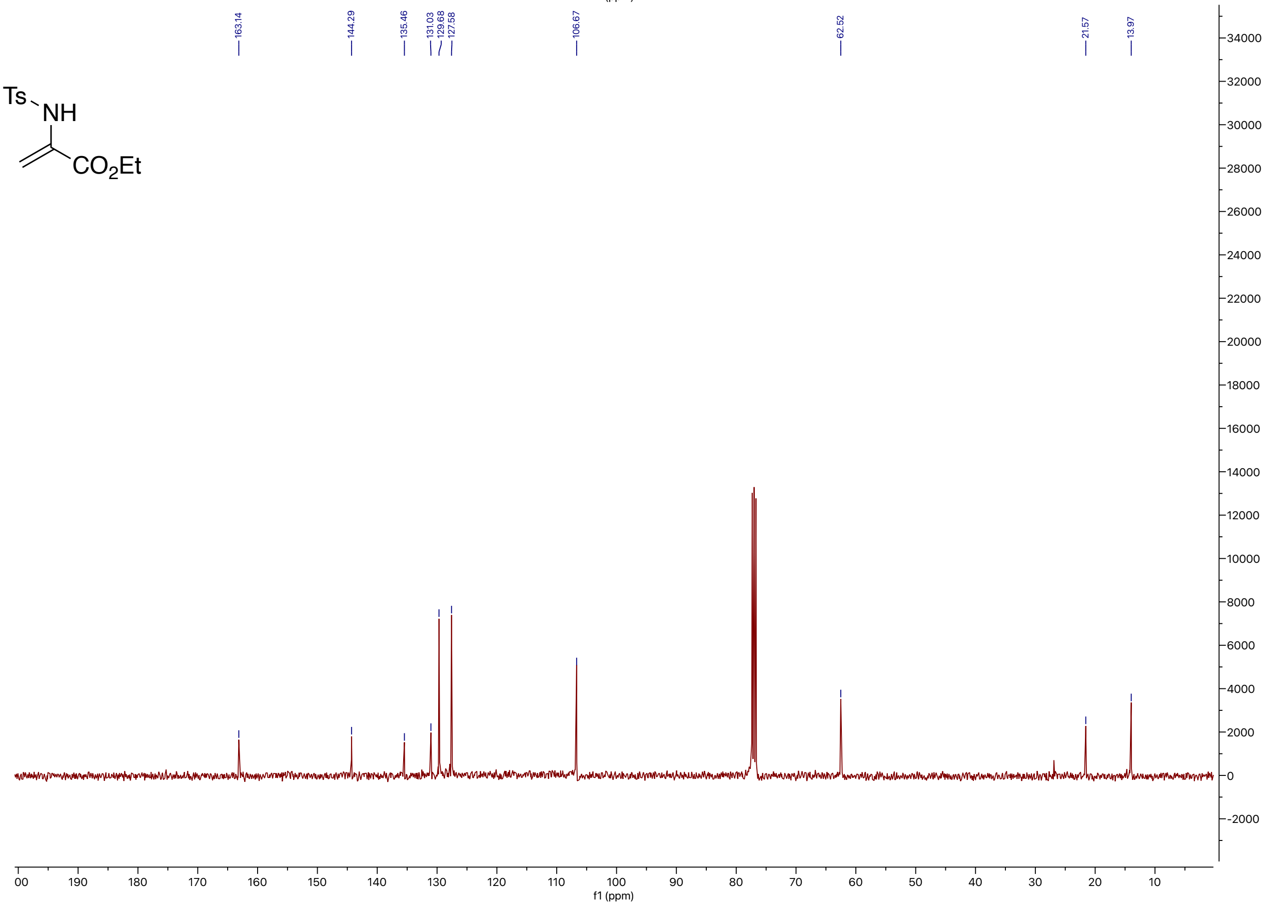

^{1}H-NMR (400 MHz), ^{13}C{^{1}H}-NMR (101 MHz) of *Ethyl 2-(Acetylamino)acrylate (2f)* - ($CDCl_3$).

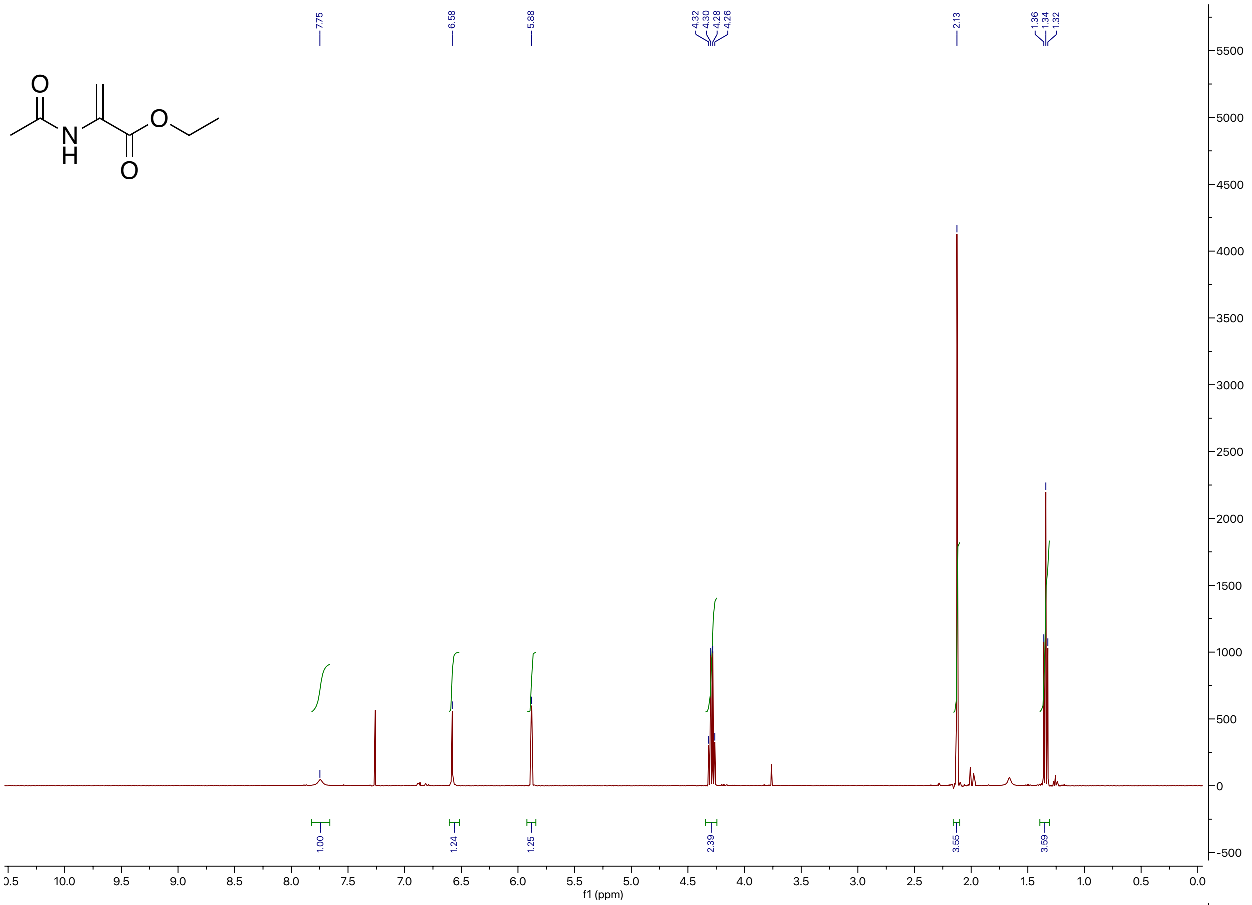

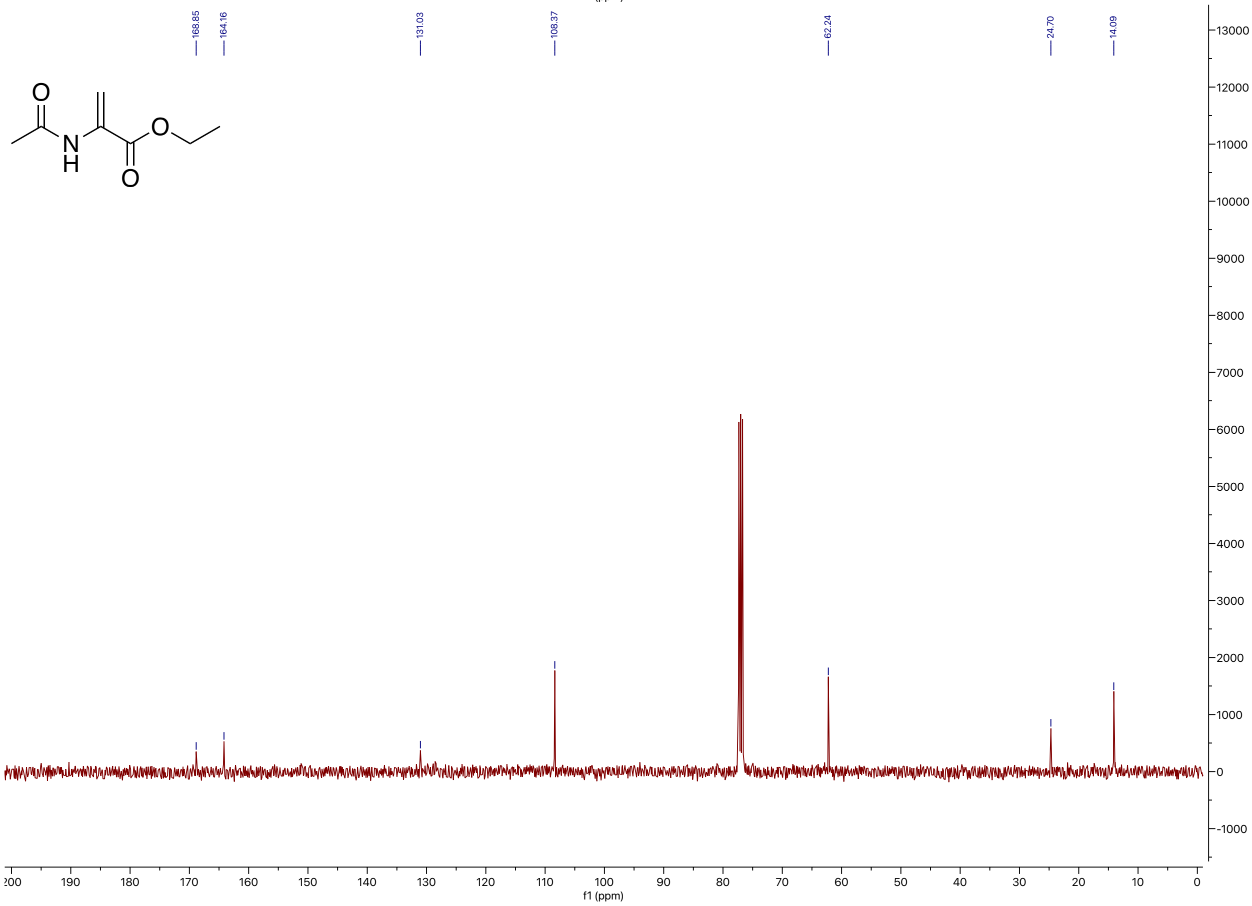

^{1}H-NMR (300 MHz),$^{13}C\{^{1}H\}$-NMR (101 MHz) of *Diethyl 2,6-dimethyl-1,4-dihydropyridine-3,5-dicarboxylate (HE-1) - (CDCl$_3$).*

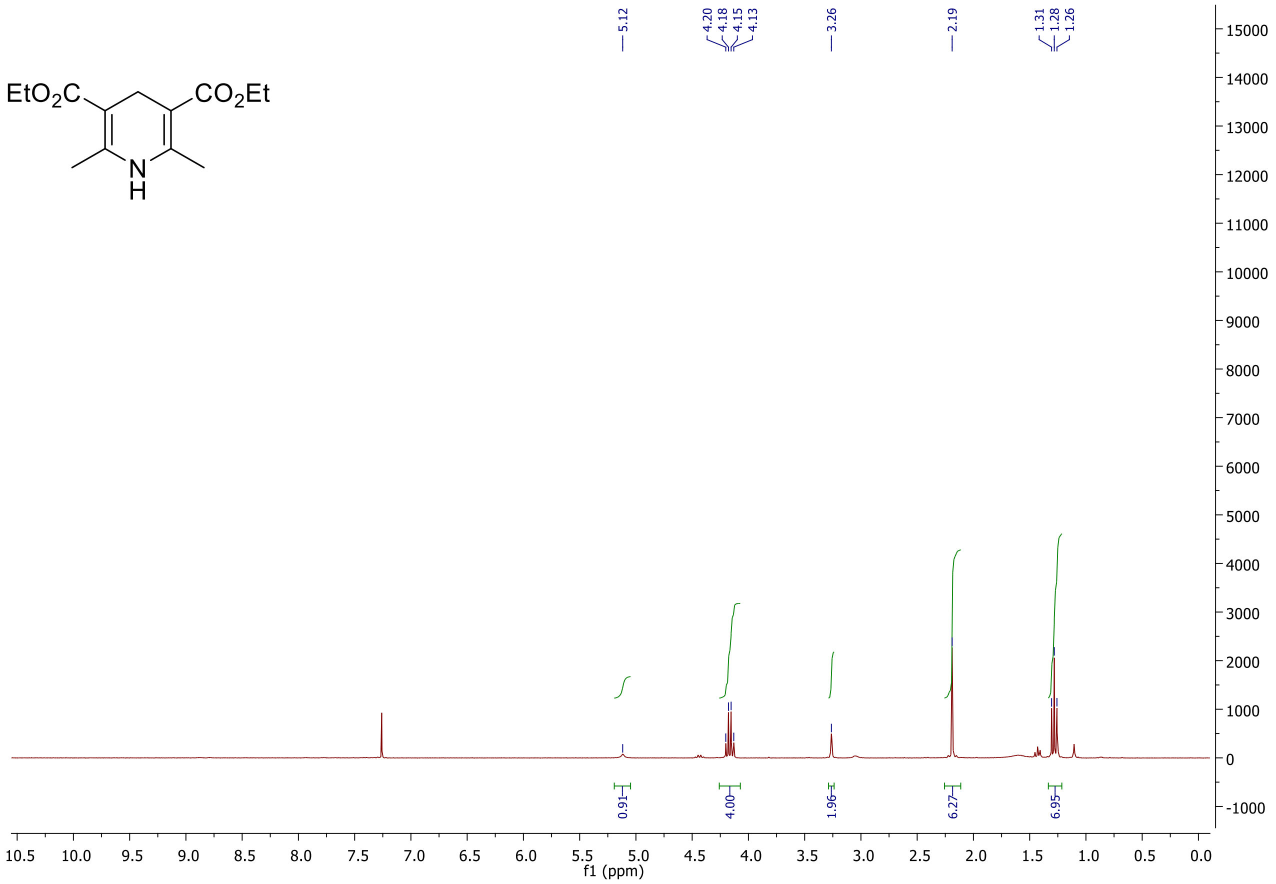

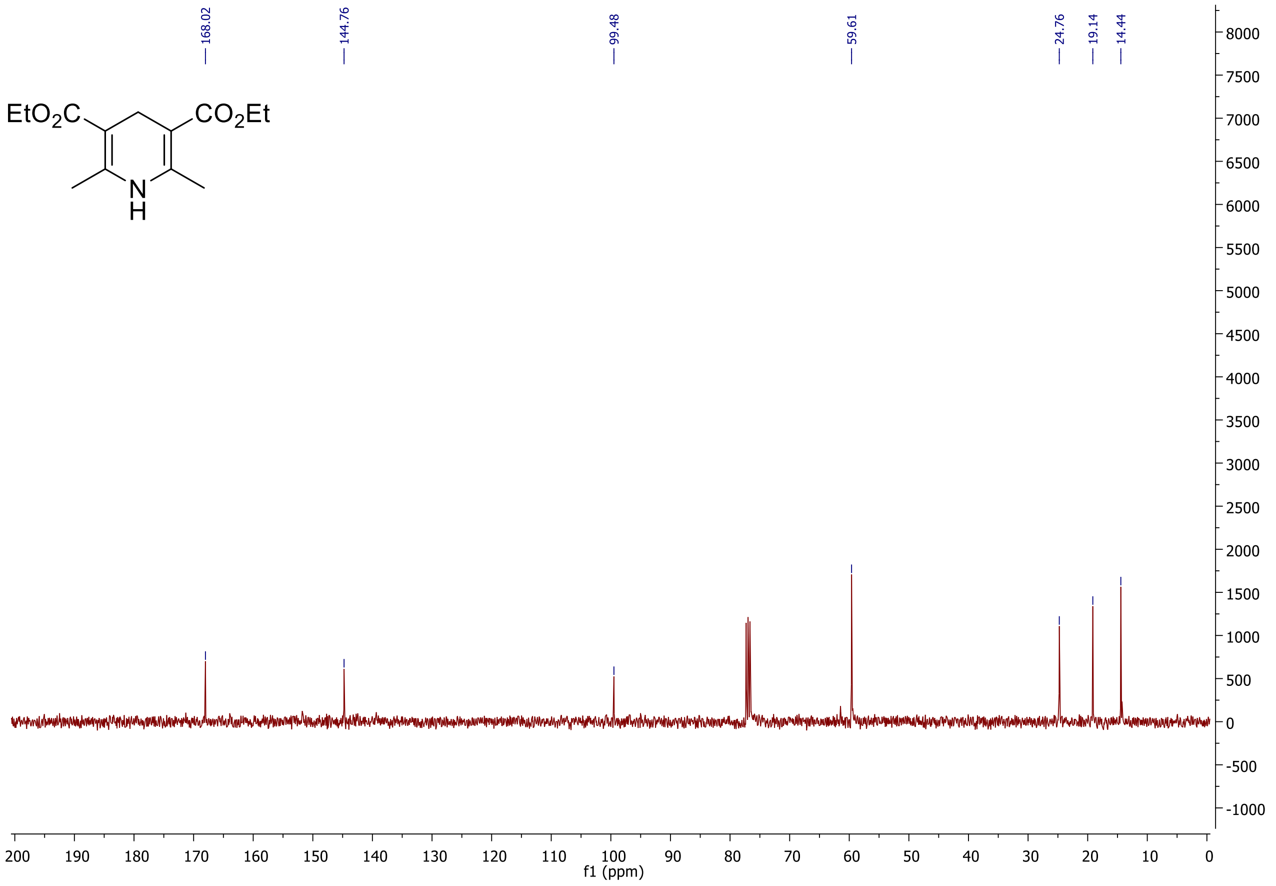

^{1}H-NMR (300 MHz),^{13}C{^{1}H}-NMR (101 MHz) of *Diethyl 2,6-dimethyl-4-phenyl-1,4-dihydropyridine-3,5-dicarboxylate (HE-2) - ($CDCl_3$).*

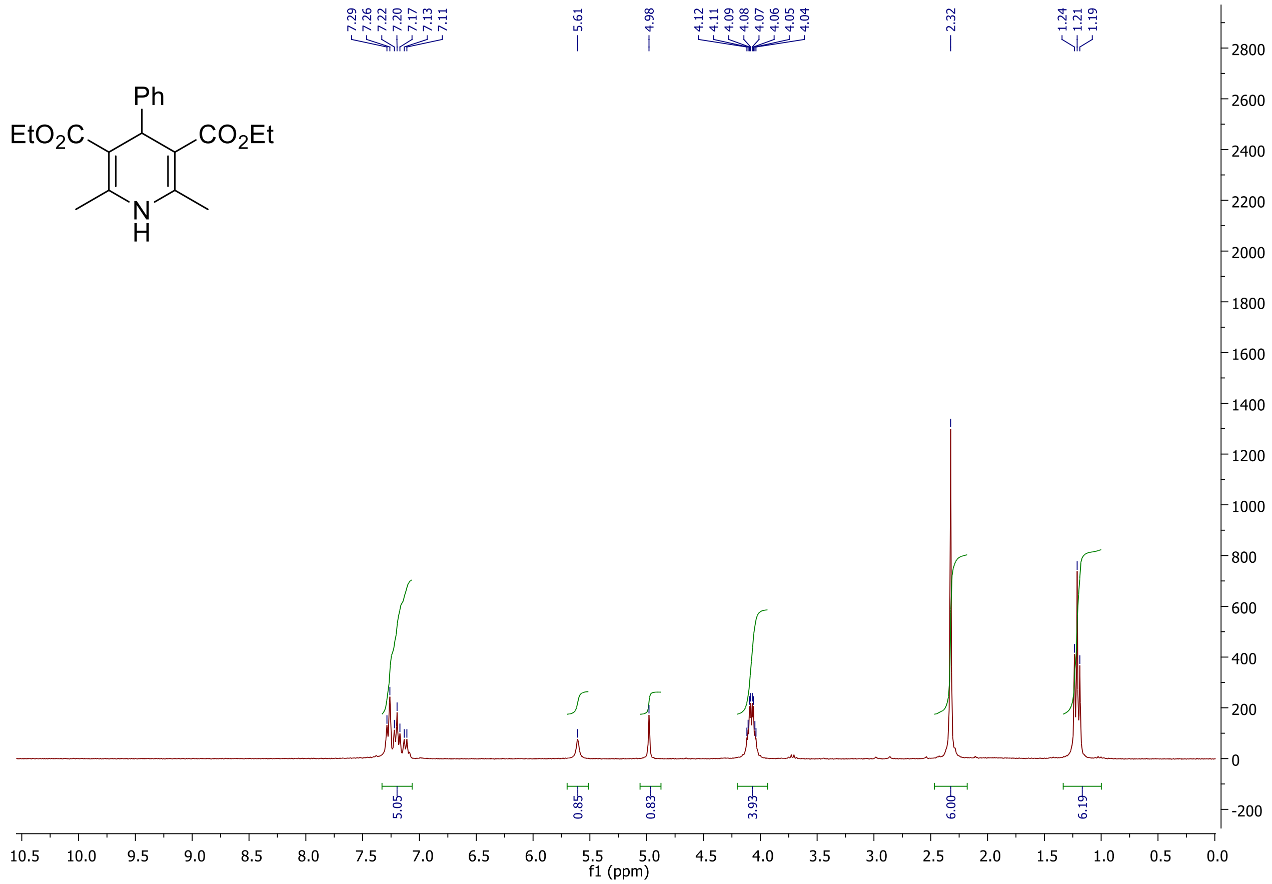

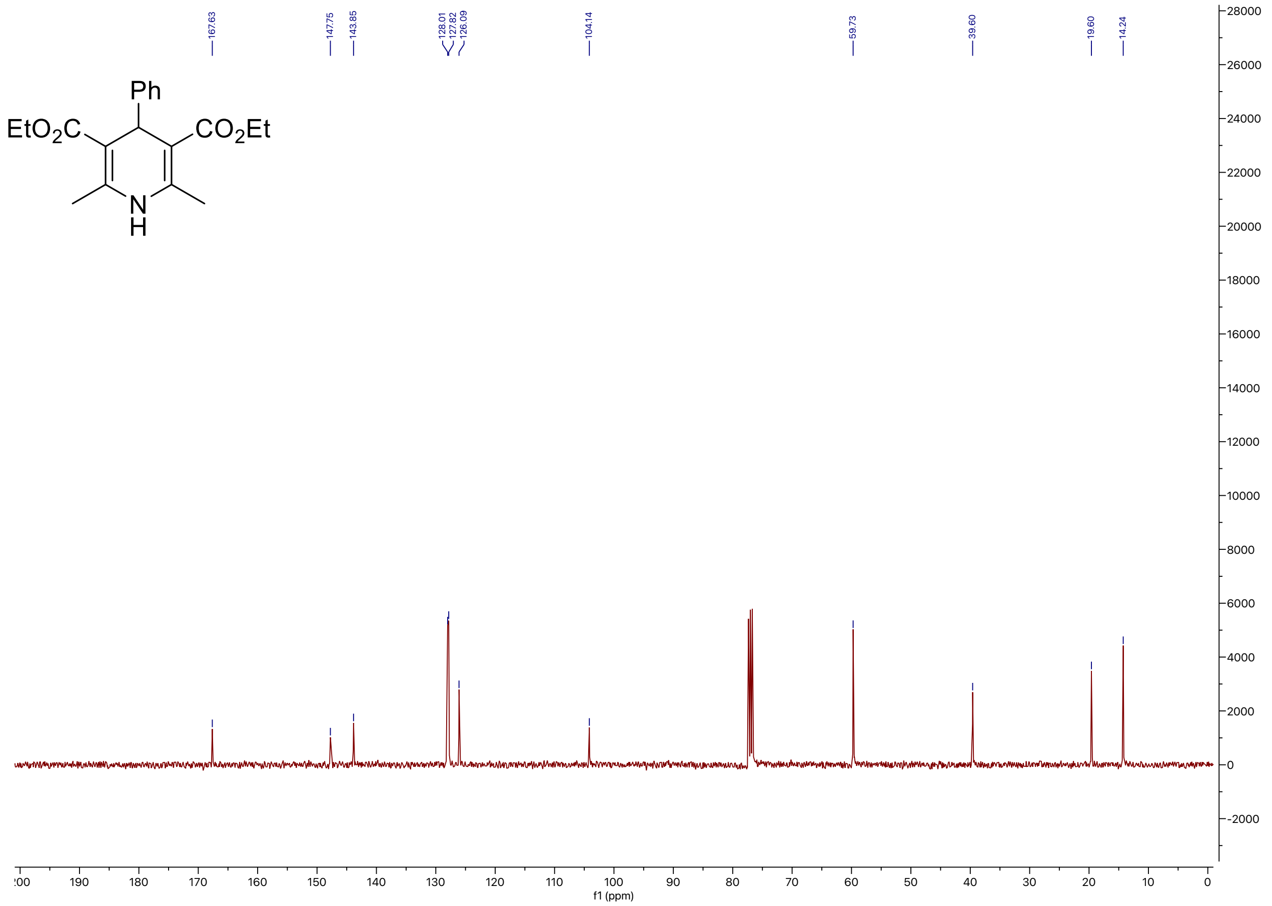

^{1}H-NMR (300 MHz),^{13}C{^{1}H}-NMR (101 MHz) of Diethyl 2,6-dimethyl-4-(o-tolyl)-1,4-dihydropyridine-3,5-dicarboxylate (HE-3) - *($CDCl_3$).*

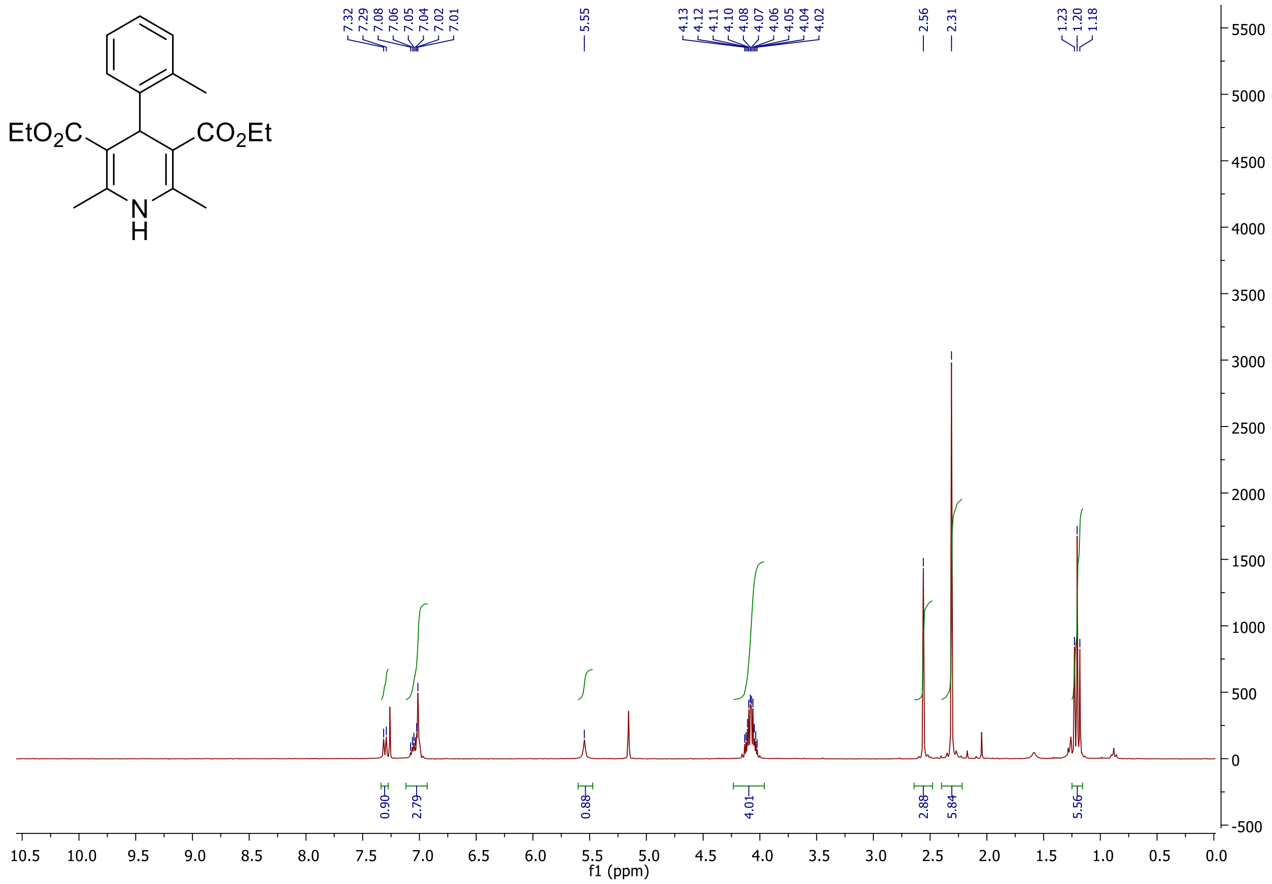

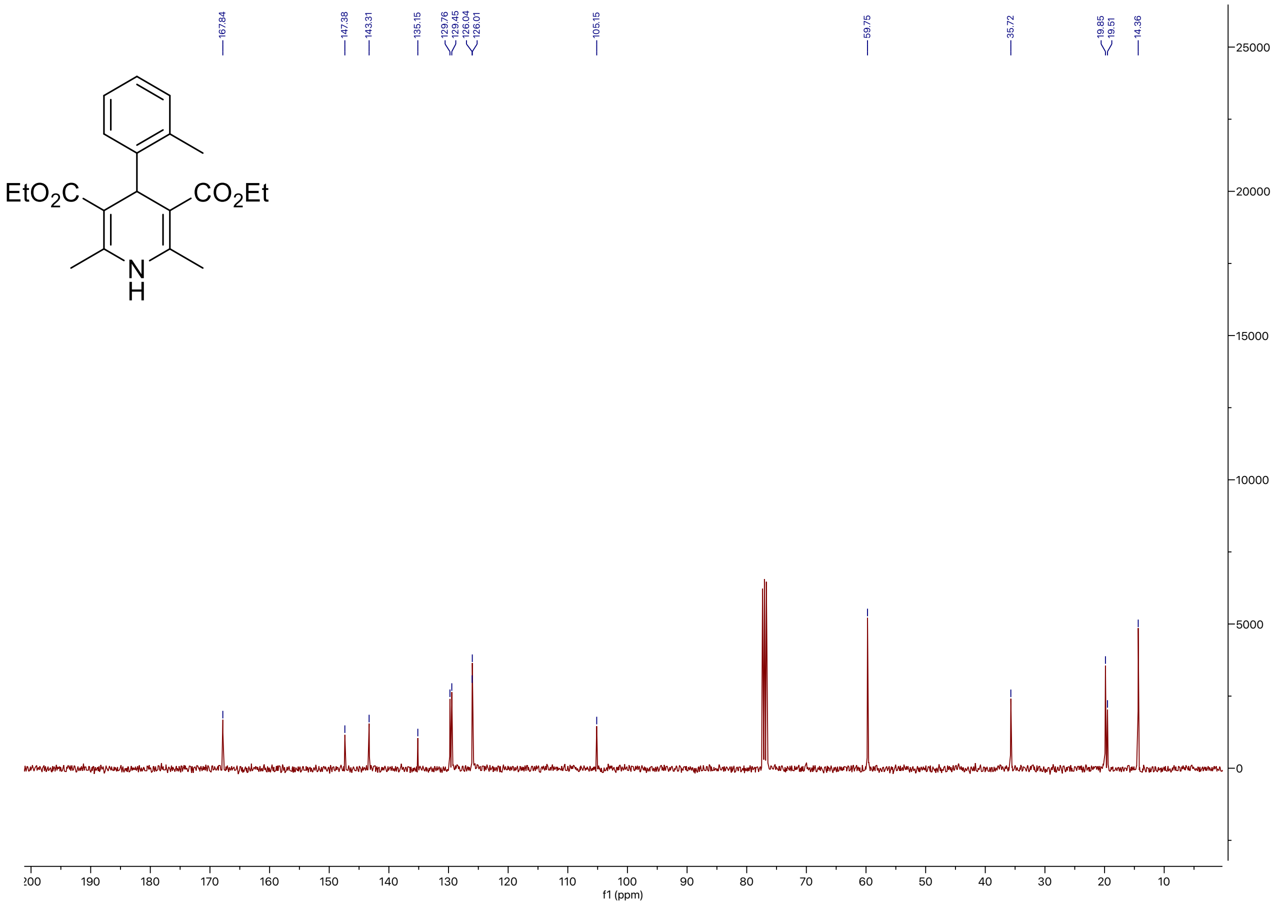

NMR OF PRODUCTS

^{1}H-NMR (400 MHz),^{13}C{^{1}H}-NMR (101 MHz) of *Methyl[3-α-(2,3,4,6-tetra-O-Benzoyl-α-D-glucopyranosyl)]-(2S/2R)-N,N-di-tert-buthoxycarbonyl-alanine (3aa) - (CDCl₃).*

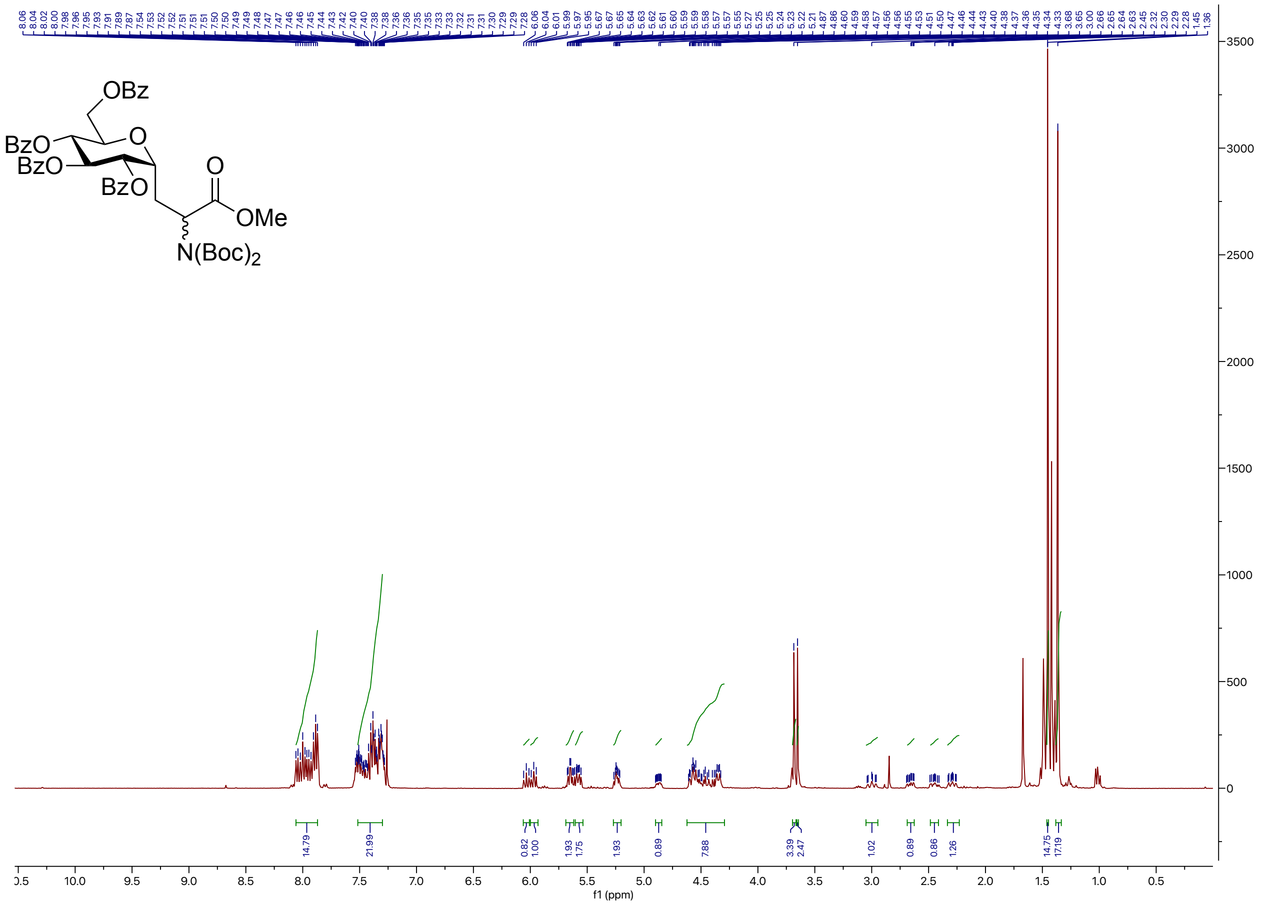

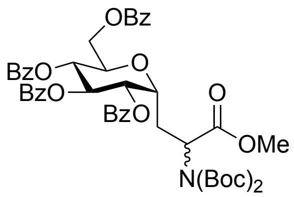
OBz
BzO
BzO
BzO
O
O
OMe
N(Boc)2

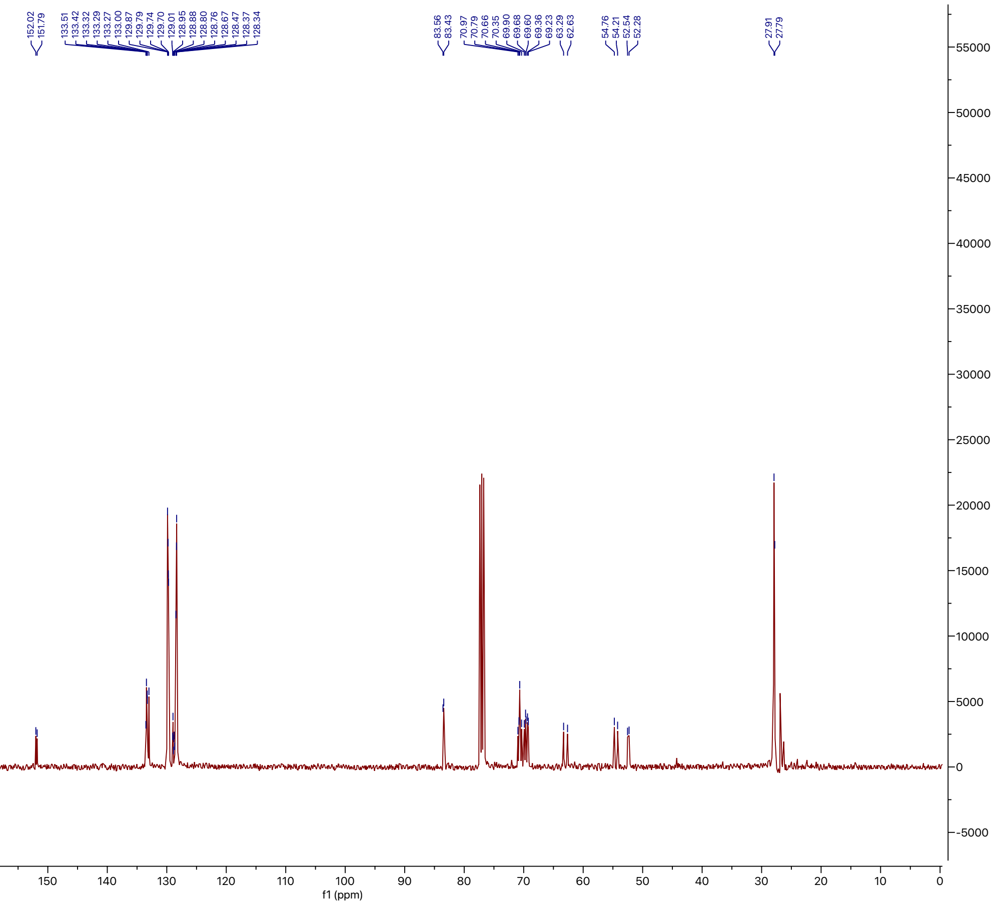
171.29
170.57
166.22
166.08
165.75
165.24
165.19
165.11
152.02
151.79
133.51
133.42
133.32
133.29
133.27
133.00
129.87
129.79
129.74
129.70
129.01
128.95
128.88
128.80
128.76
128.67
128.47
128.37
128.34
83.56
83.43
70.97
70.79
70.66
70.35
69.90
69.68
69.60
69.36
69.23
63.29
62.63
54.76
54.21
52.54
52.28
27.91
27.79
f1 (ppm)

^{1}H-NMR (400 MHz),^{13}C{^{1}H}-NMR (101 MHz)of *Methyl[3-α-(2,3,4,6-tetra-O-Benzoyl-α-D-mannopyranosyl)]-(2S/2R)-N,N-di-tert-buthoxycarbonyl-alanine (3ba) - (CDCl$_3$).*

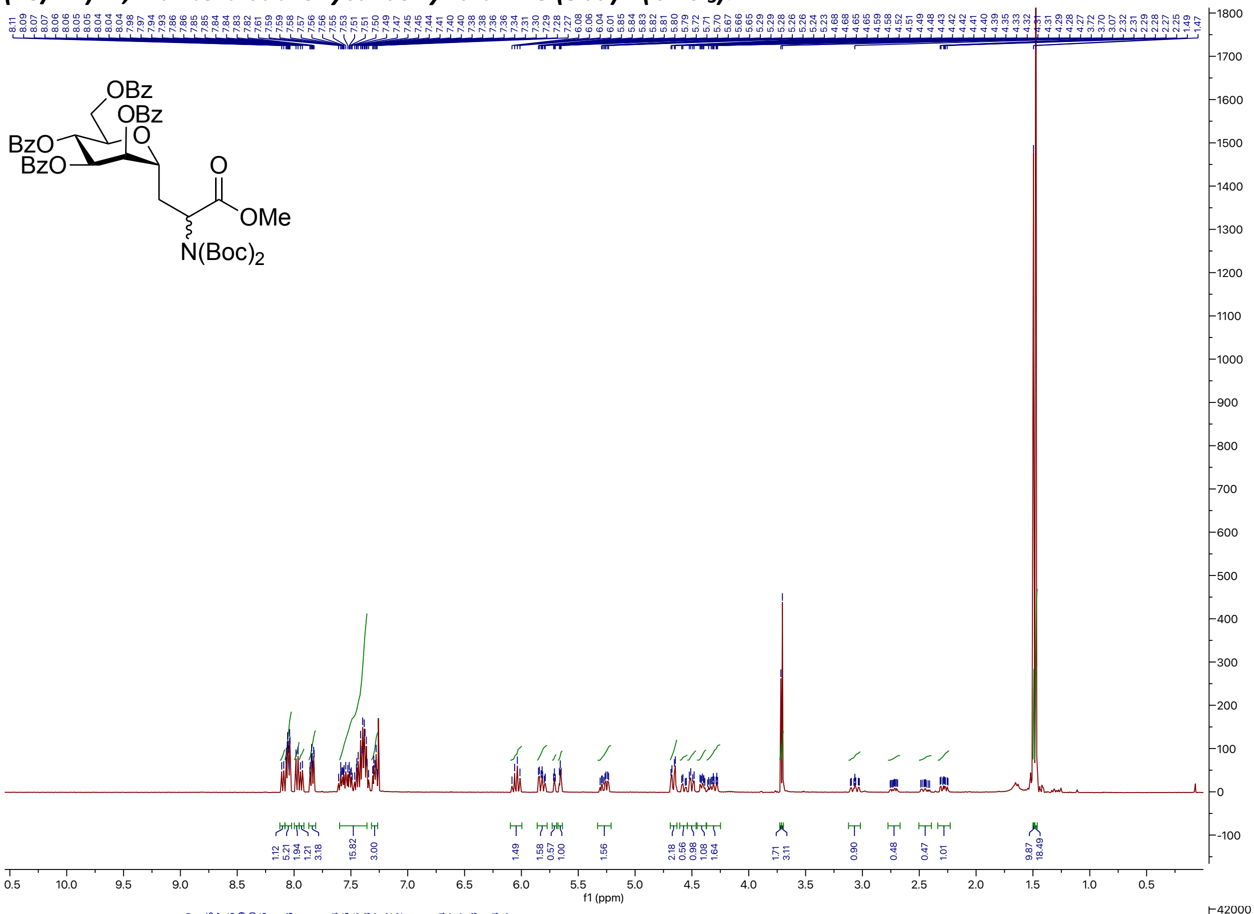

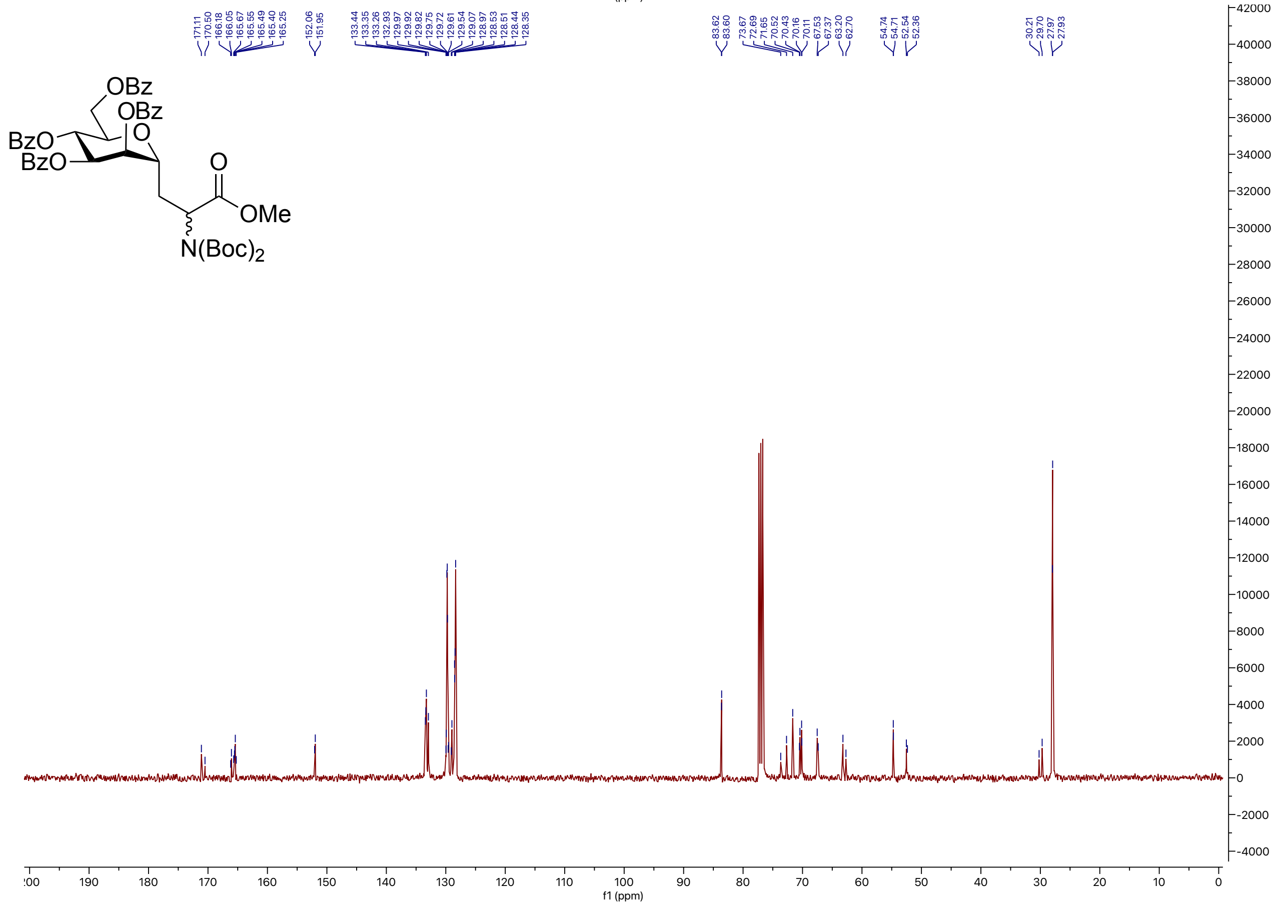

^{1}H-NMR (400 MHz),^{13}C{^{1}H}-NMR (101 MHz)of *Methyl[3-α-(2,3,4,6-tetra-O-Benzoyl-α-D-galactopyranosyl)]-(2S/2R)-N,N-di-tert-buthoxycarbonyl-alanine (3ca) - (CDCl$_3$).*

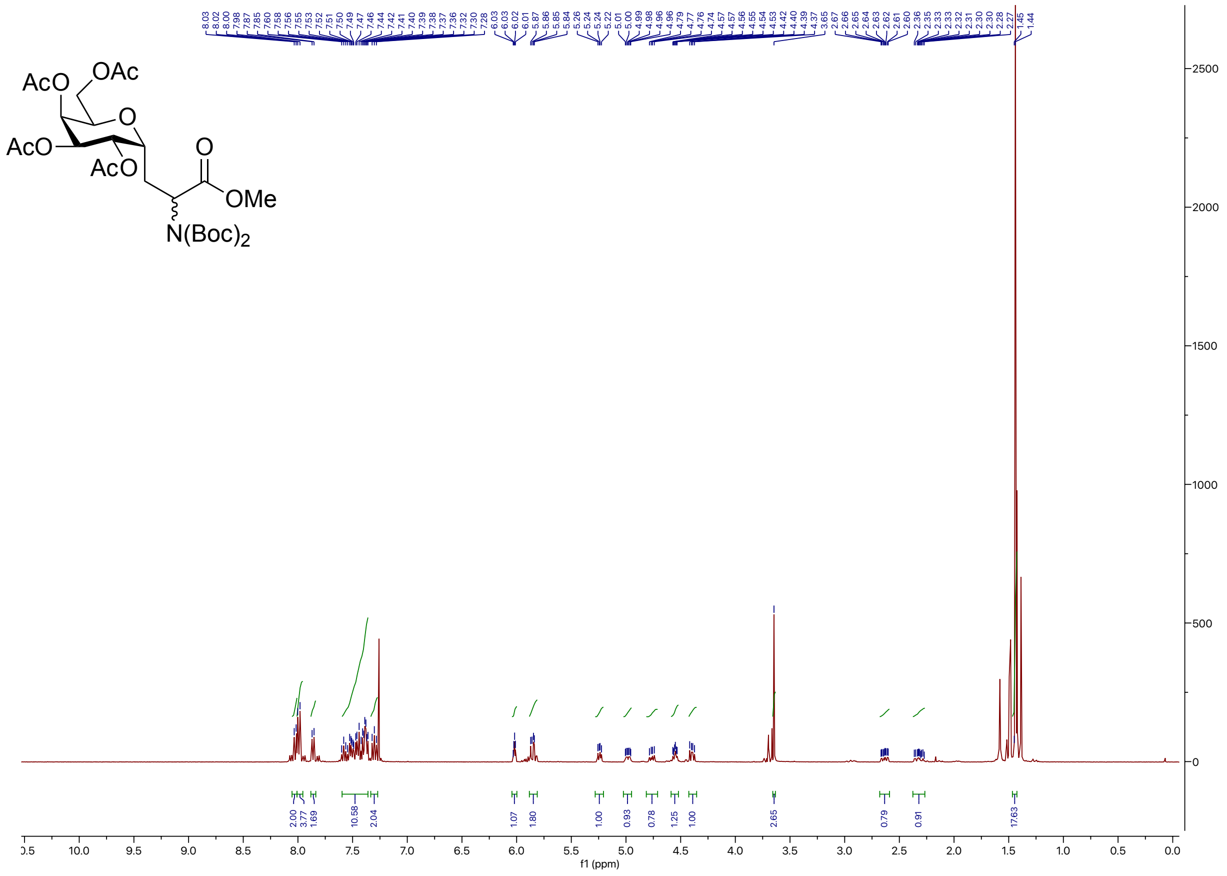

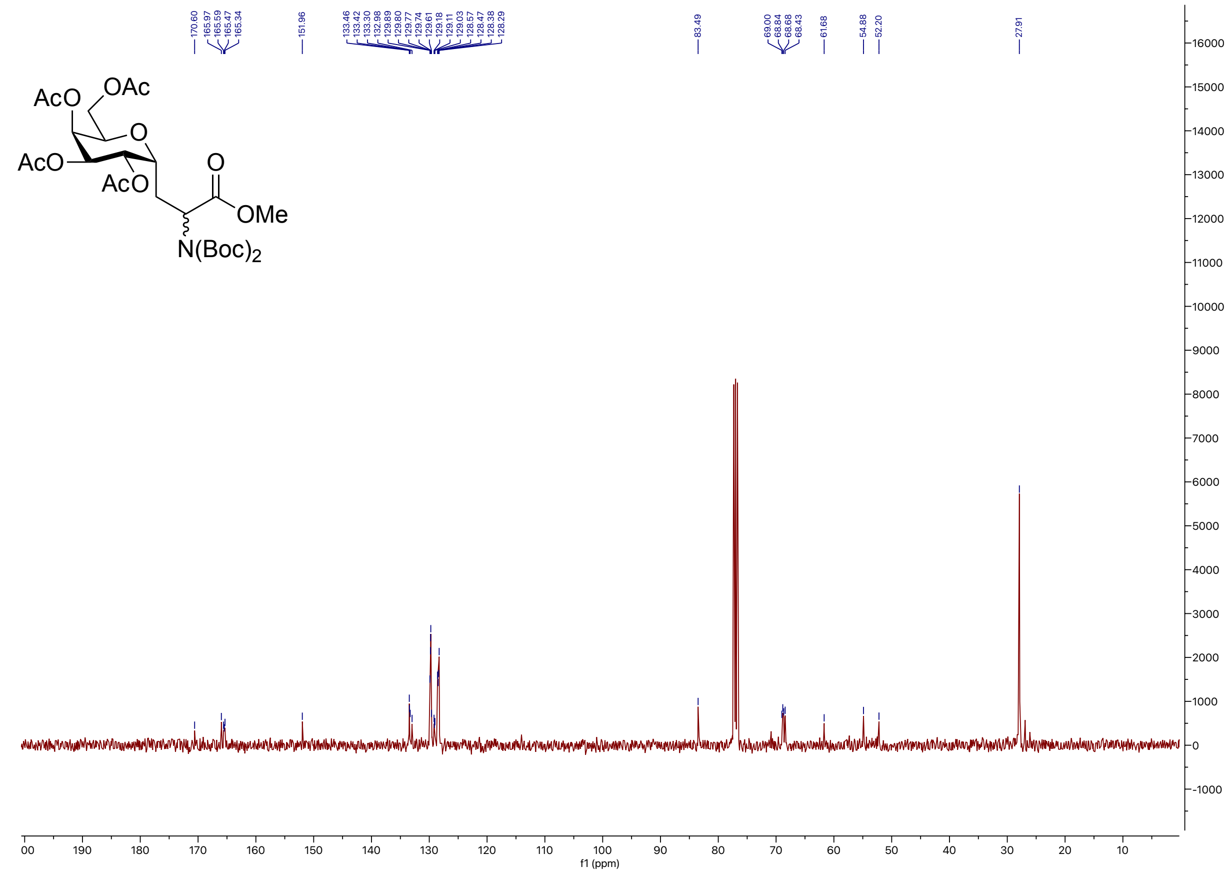

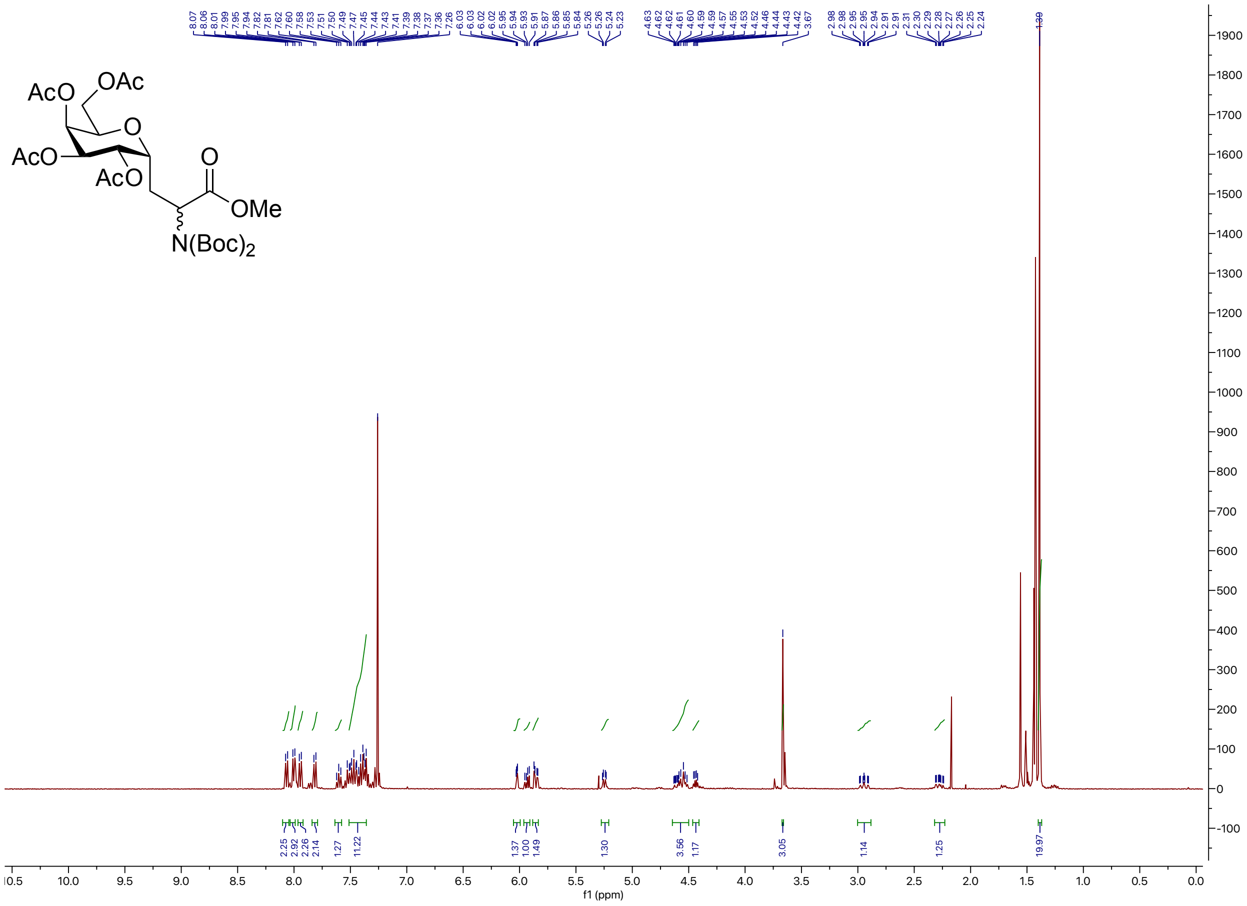
AcO
OAc
O
AcO
AcO
O
OMe
N(Boc)2
f1 (ppm)

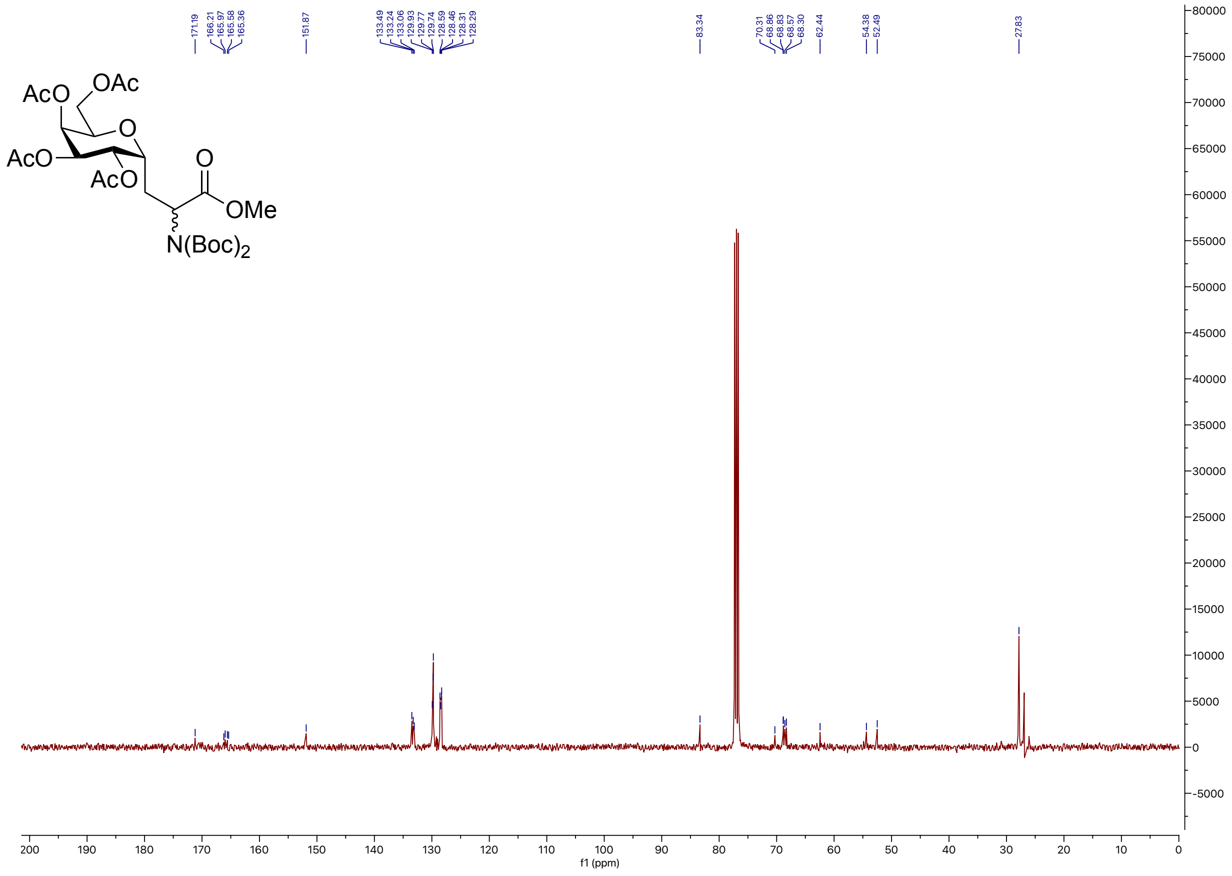
AcO
OAc
O
AcO
AcO
O
OMe
N(Boc)2
f1 (ppm)

^{1}H-NMR (400 MHz),^{13}C{^{1}H}-NMR (101 MHz)of *Methyl[3-α(2,3,4,6-tetra-O-Benzoyl-α-D-galactopyranosyl)]-(2S/2R)-N,N-di-tert-buthoxycarbonyl-(3S/3R)-methyl-alanine (3ab) - (CDCl$_3$).*

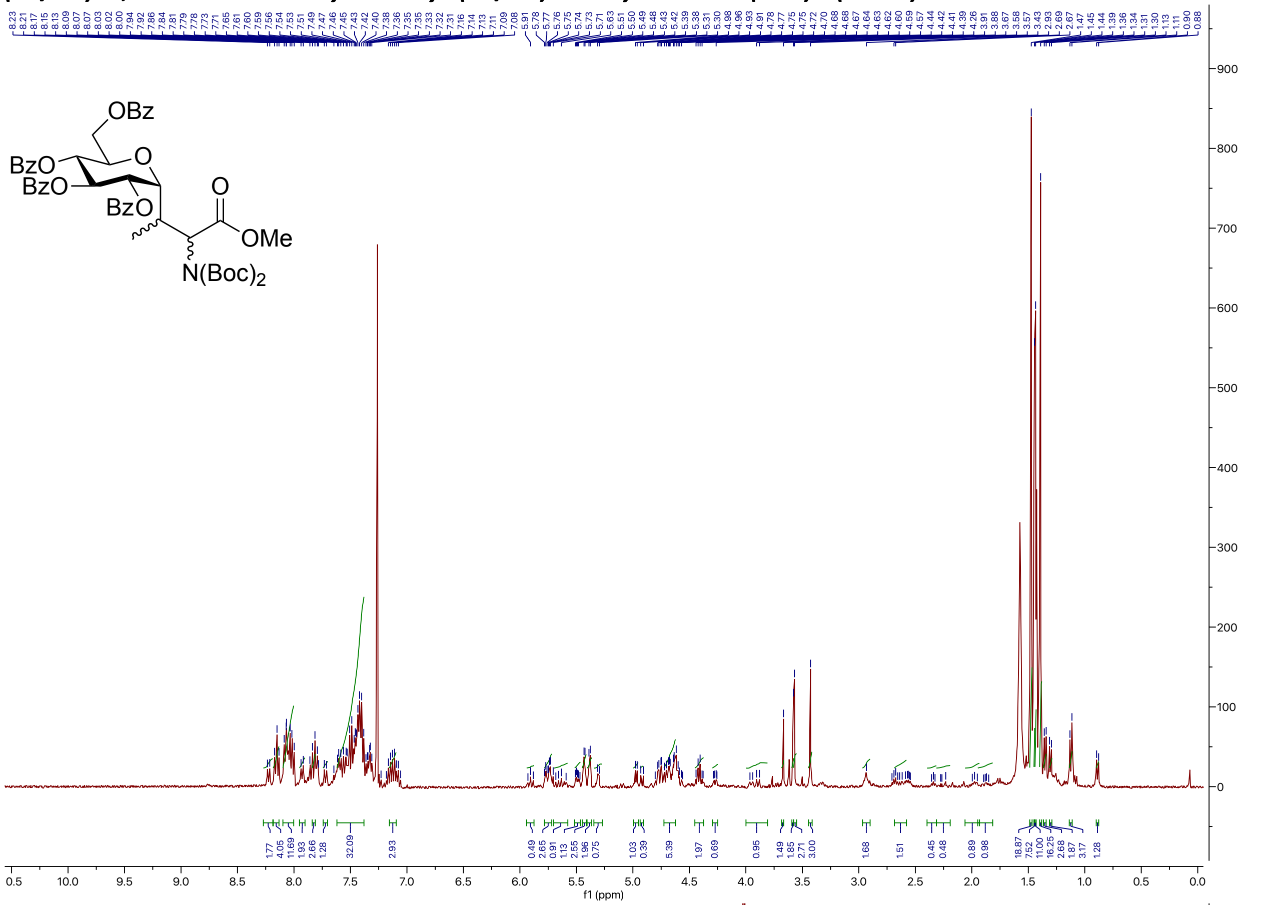

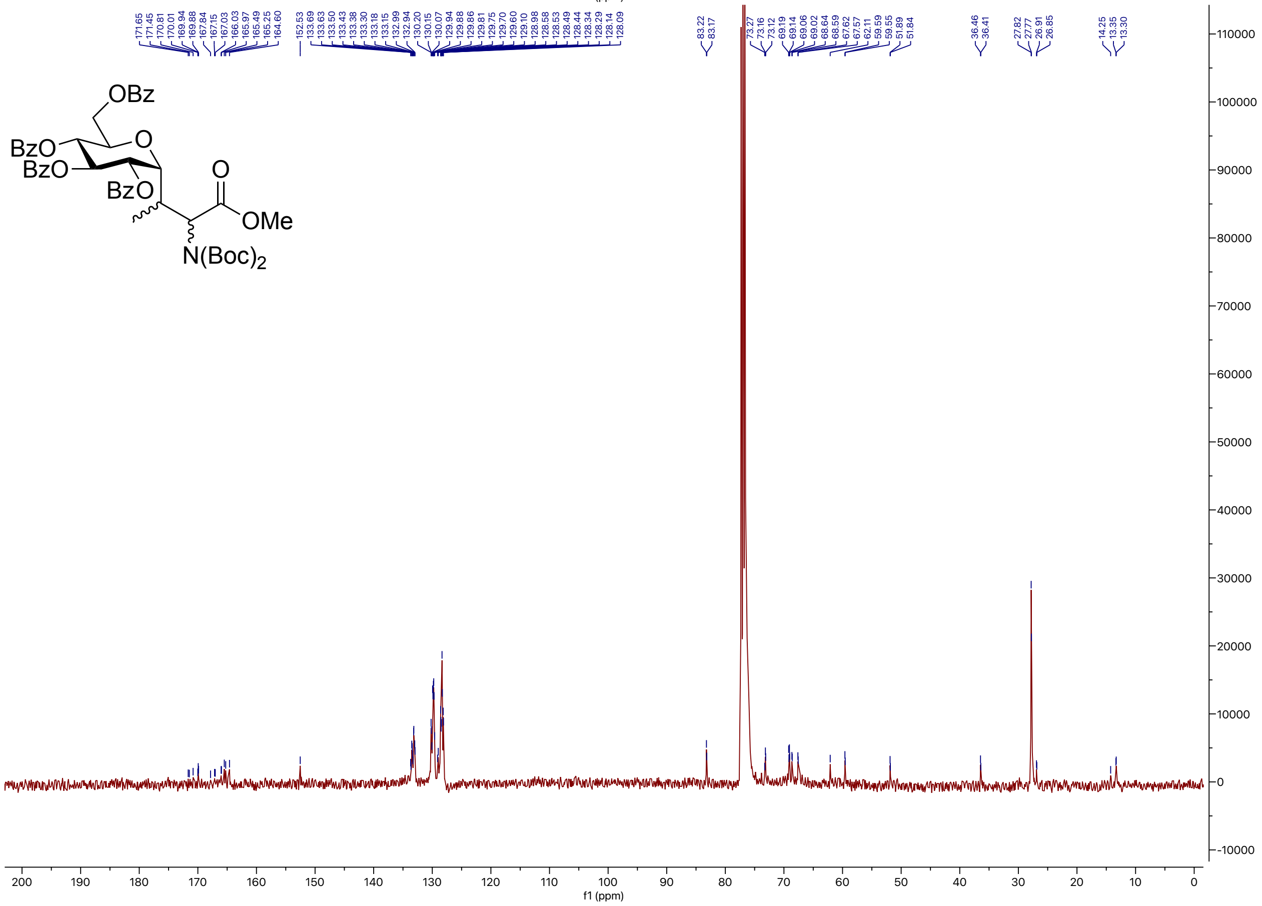

^{1}H-NMR (400 MHz),^{13}C{^{1}H}-NMR (101 MHz)of *Methyl[3-α(2,3,4,6-tetra-O-Benzoyl-α-D-galactopyranosyl]-(2S/2R)-N-phenyl-(3S/3R)-methyl-alanine (3ac) - (CDCl$_3$).*

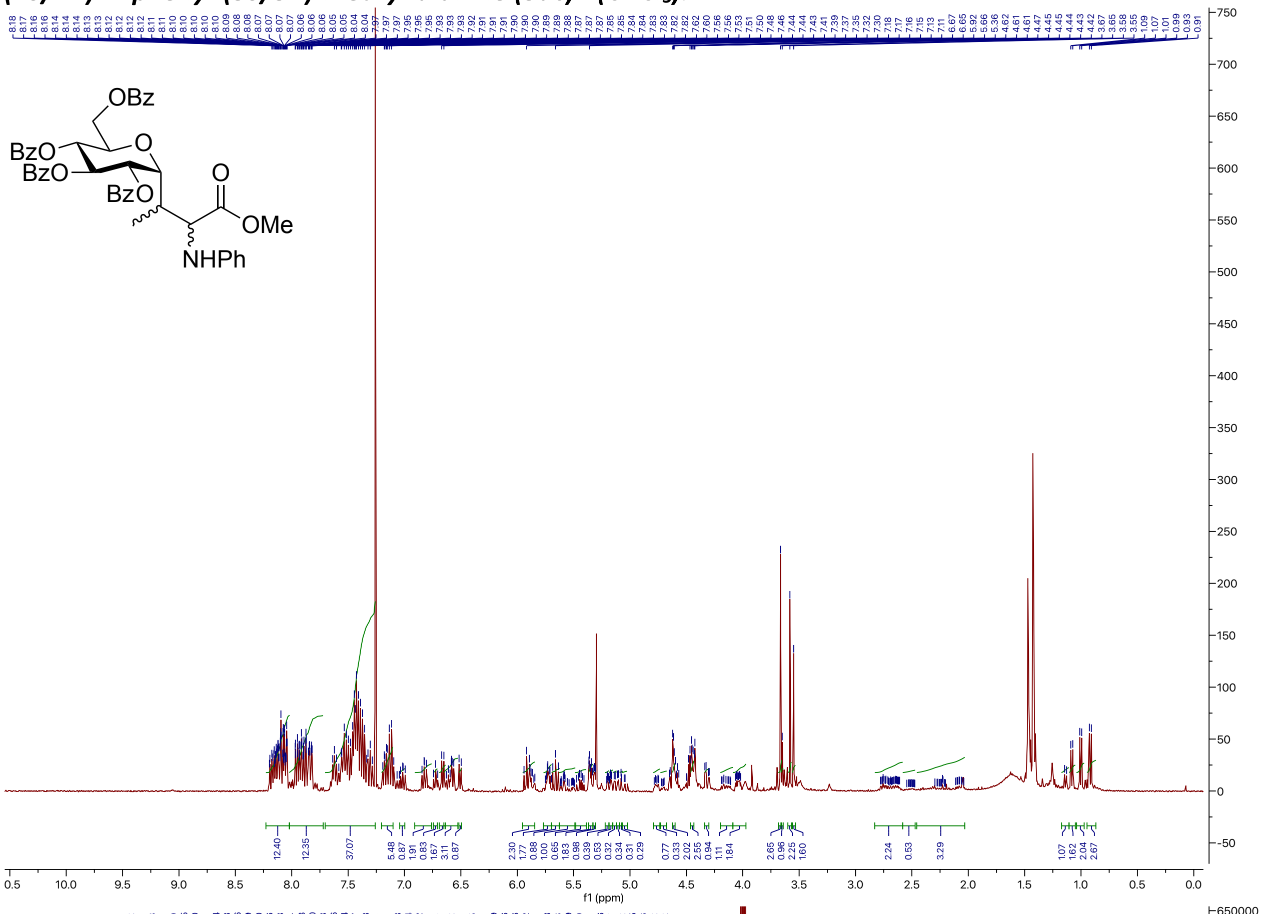

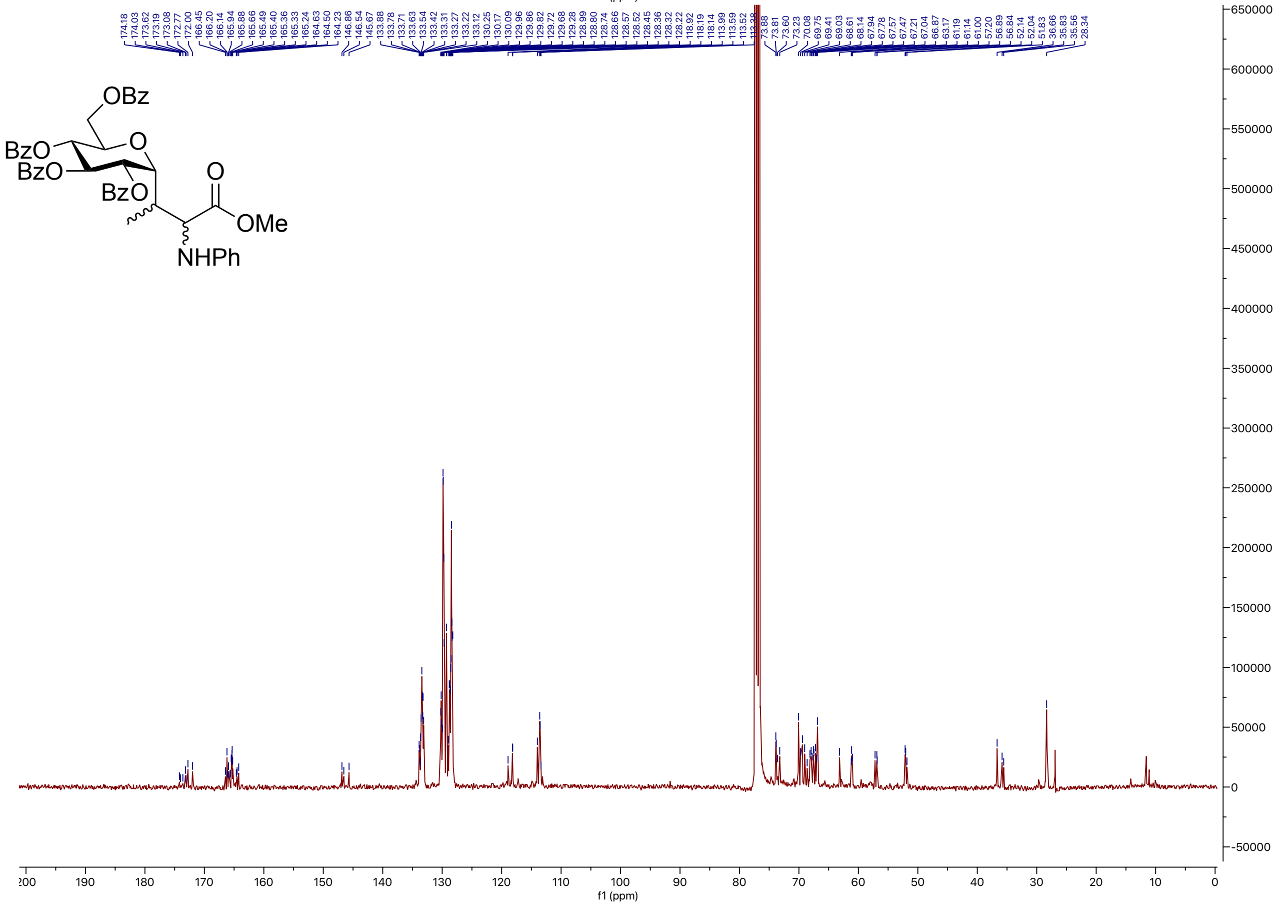

^{1}H-NMR (400 MHz),^{13}C{^{1}H}-NMR (101 MHz) of *Methyl 3-(2,3,4,6-tetra-O-benzoyl-α-D-glucopyranosyl)propanoate (3ad)* - ($CDCl_3$).

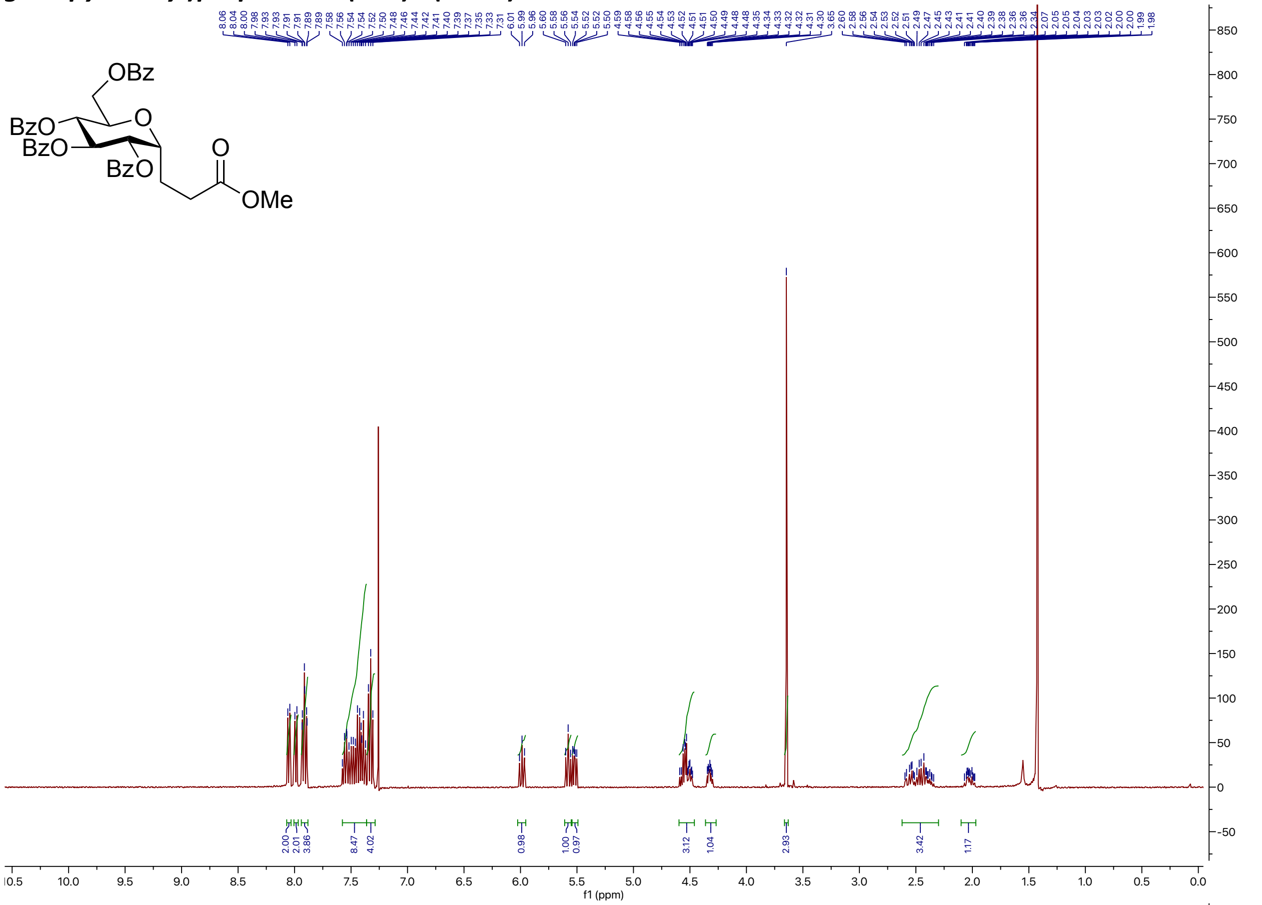

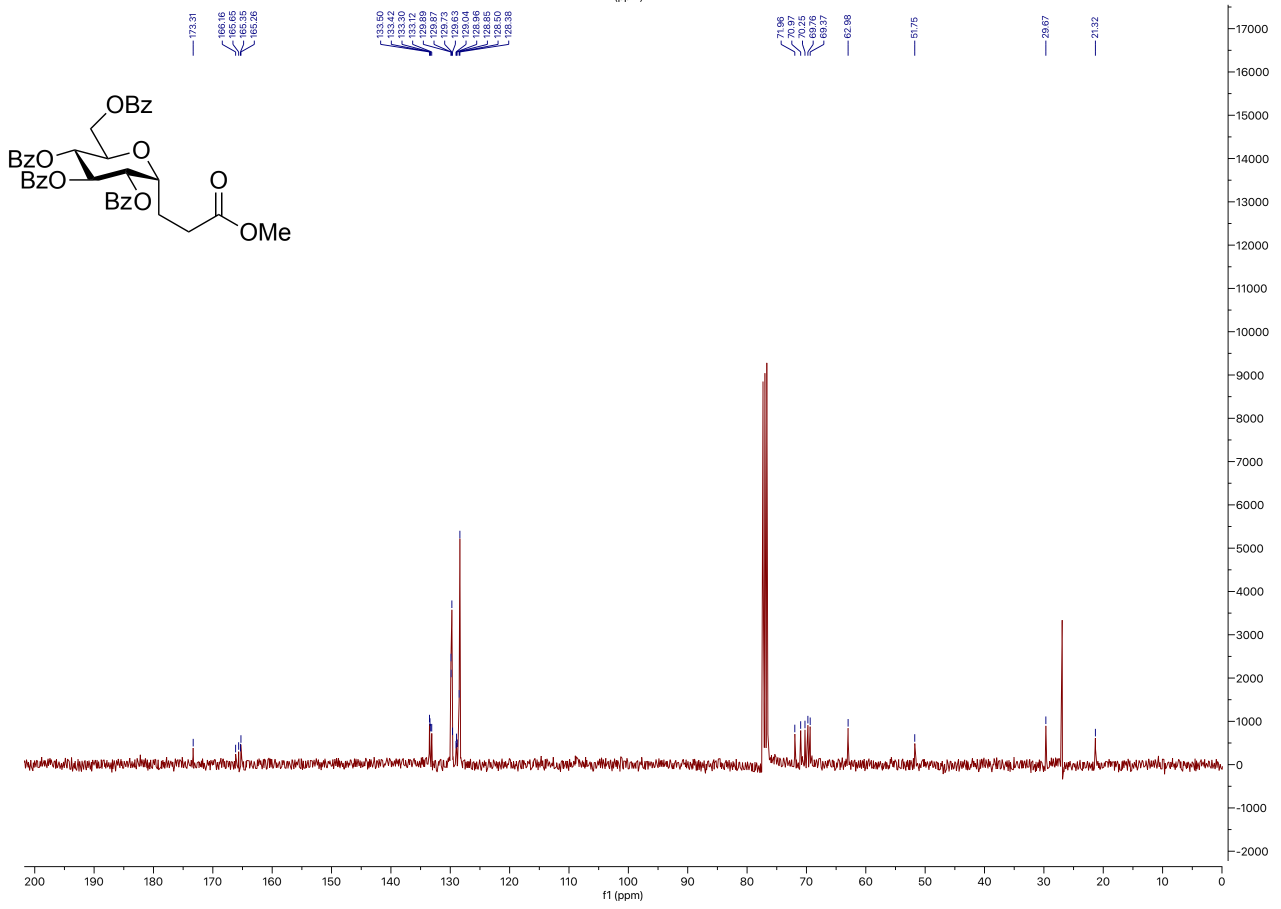

^{1}H-NMR (400 MHz),^{13}C{^{1}H}-NMR (101 MHz) of *Methyl[3-α(2,3,4,6-Tetra-O-benzoyl-β-D-galactopyranosyl-(1→4)-2,3,6-tri-O-benzoyl-α-D-glucopyranosyl]-(2S/2R)-N,N-di-tert-buthoxycarbonyl-alanine (3da) - ($CDCl_3$).*

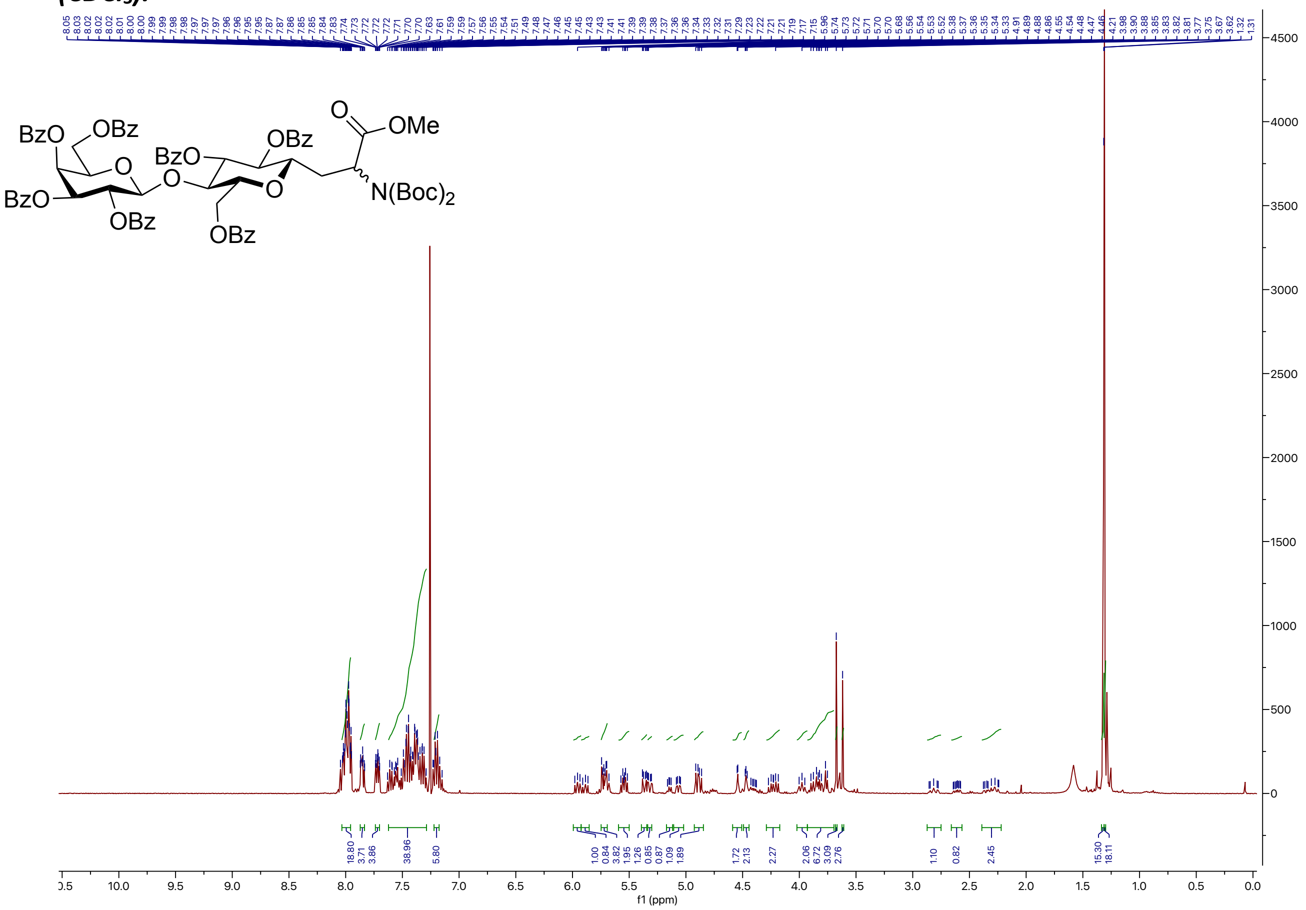

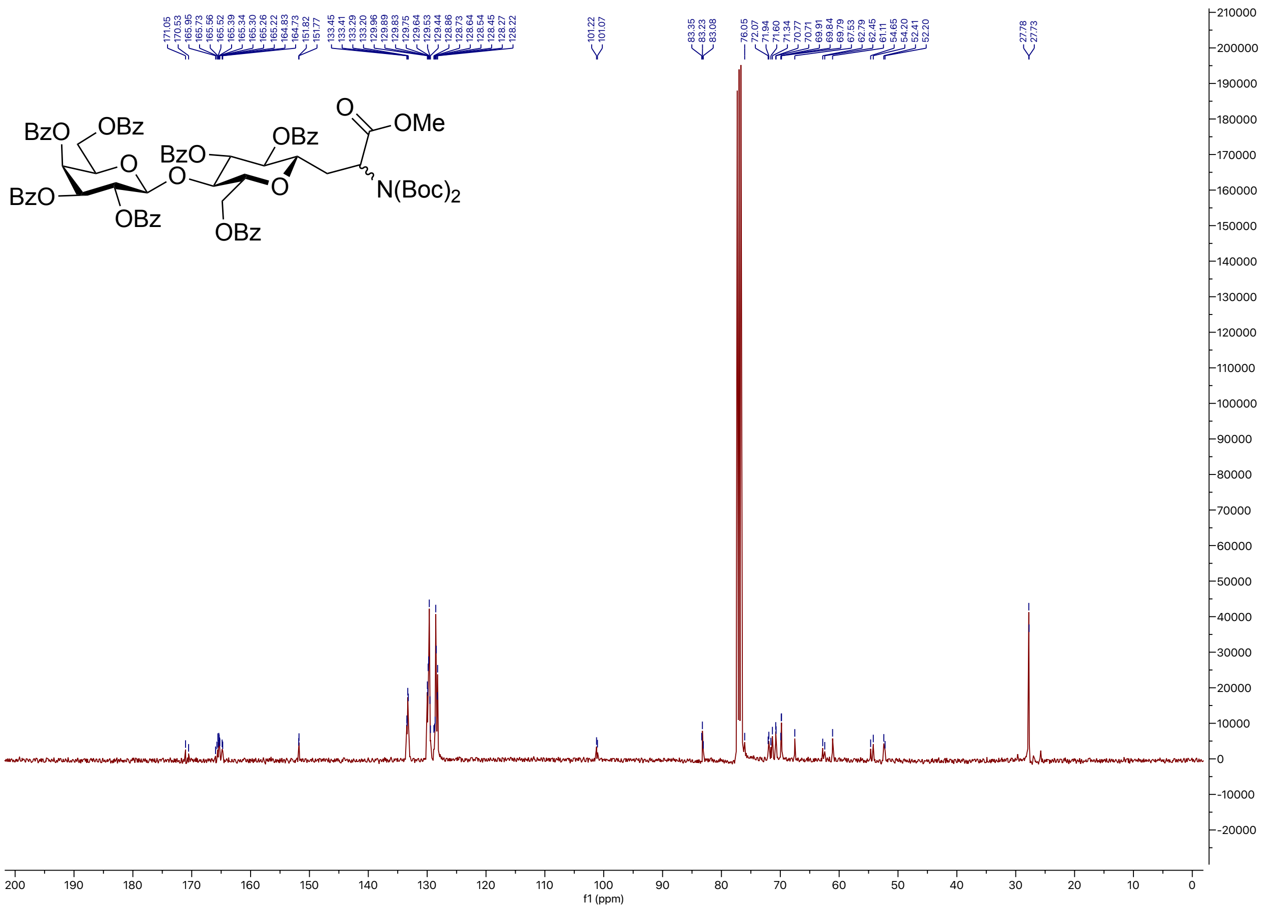

^{1}H-NMR (400 MHz),^{13}C{^{1}H}-NMR (101 MHz)of Methyl[3-α-(2,3:5,6-Di-*O*-isopropylidene-α-D-mannofuranosyl)]-(2S/2R)-N,N-di-*tert*-buthoxycarbonyl-alanine (3ea).

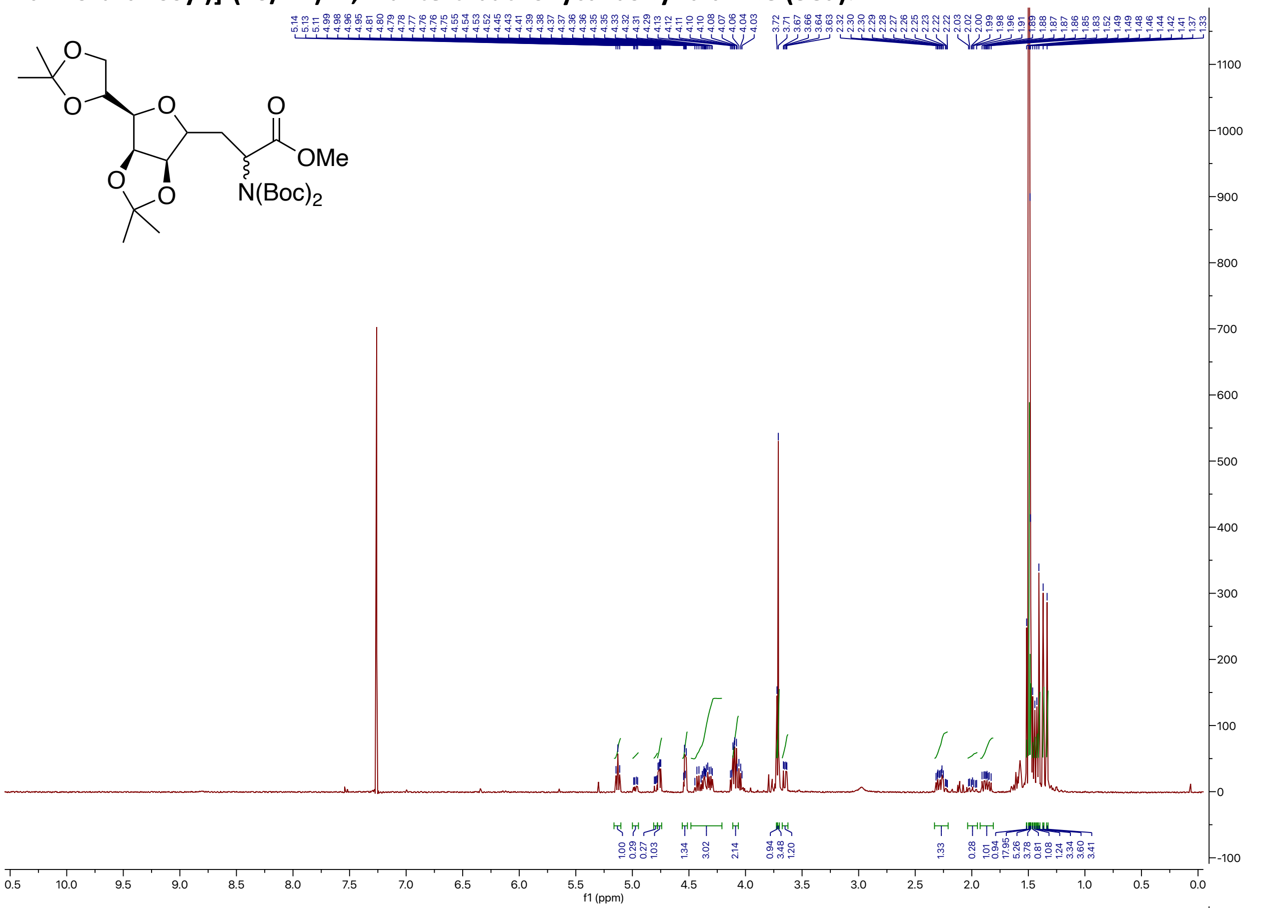

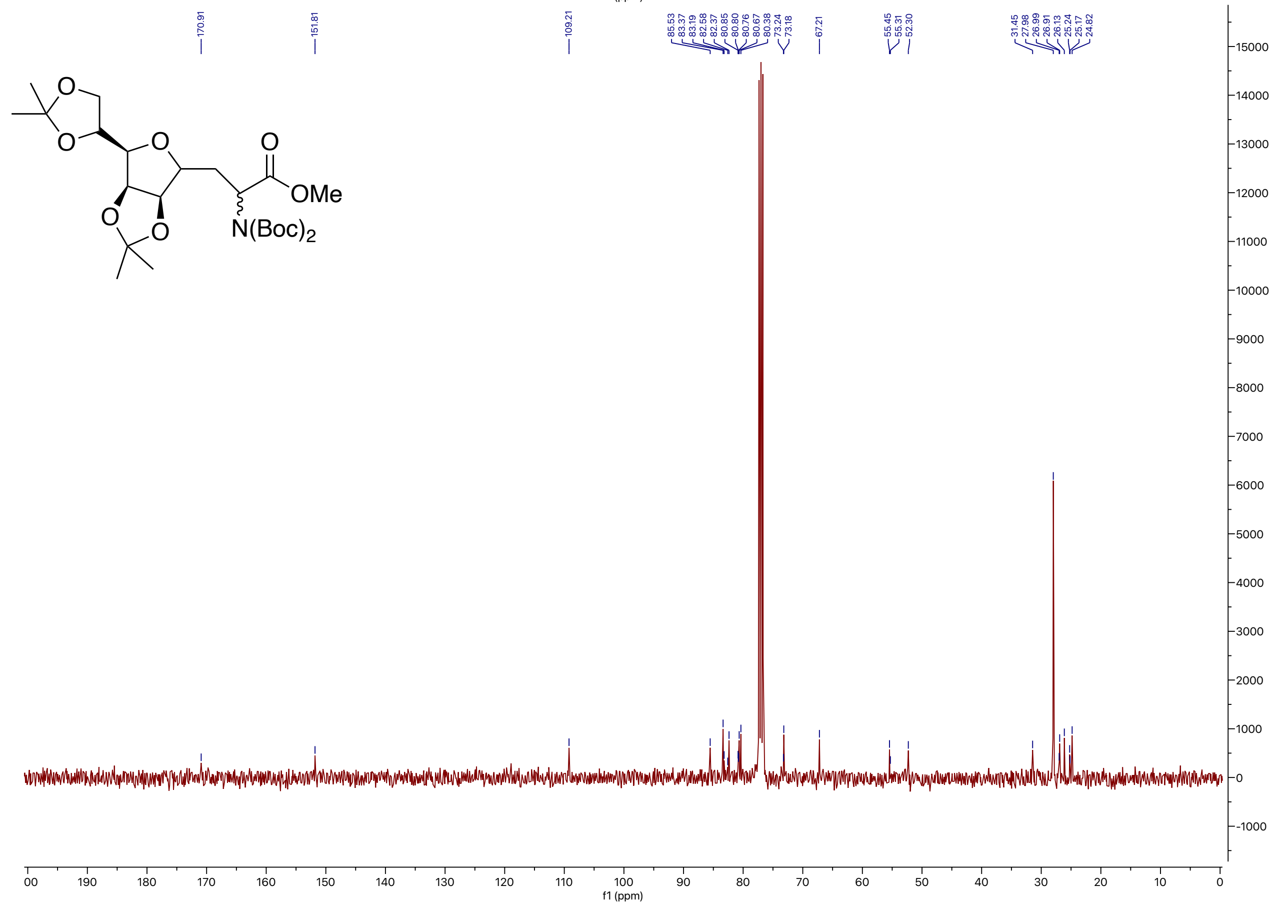

^{1}H-NMR (400 MHz),^{13}C{^{1}H}-NMR (101 MHz) of *Methyl[3-α(2,3,4,6-Tetra-O-acetyl-α-glucopyranosyl)]-(2S/2R)-N,N-di-tert-buthoxycarbonyl-alanine (3fa) - ($CDCl_3$).*

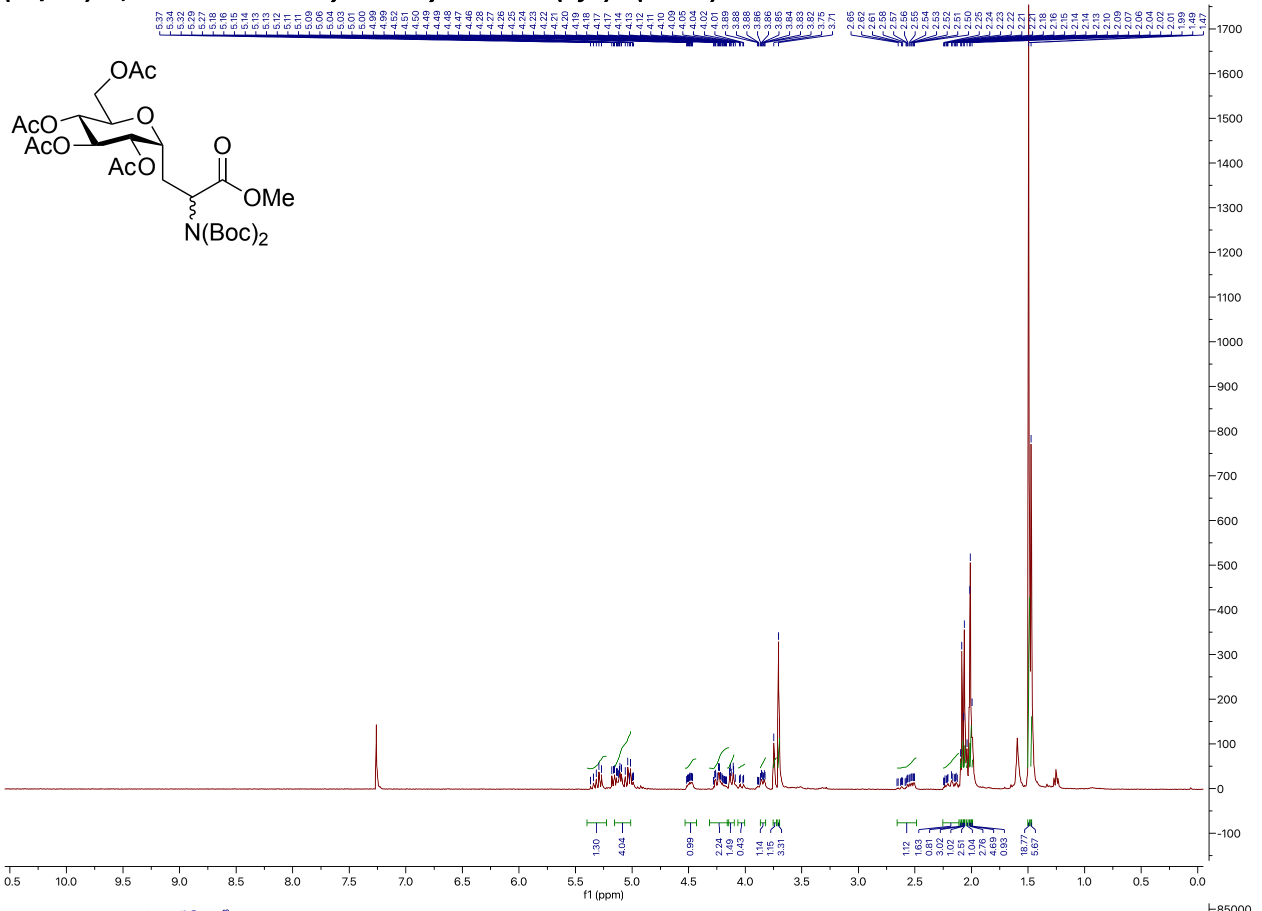

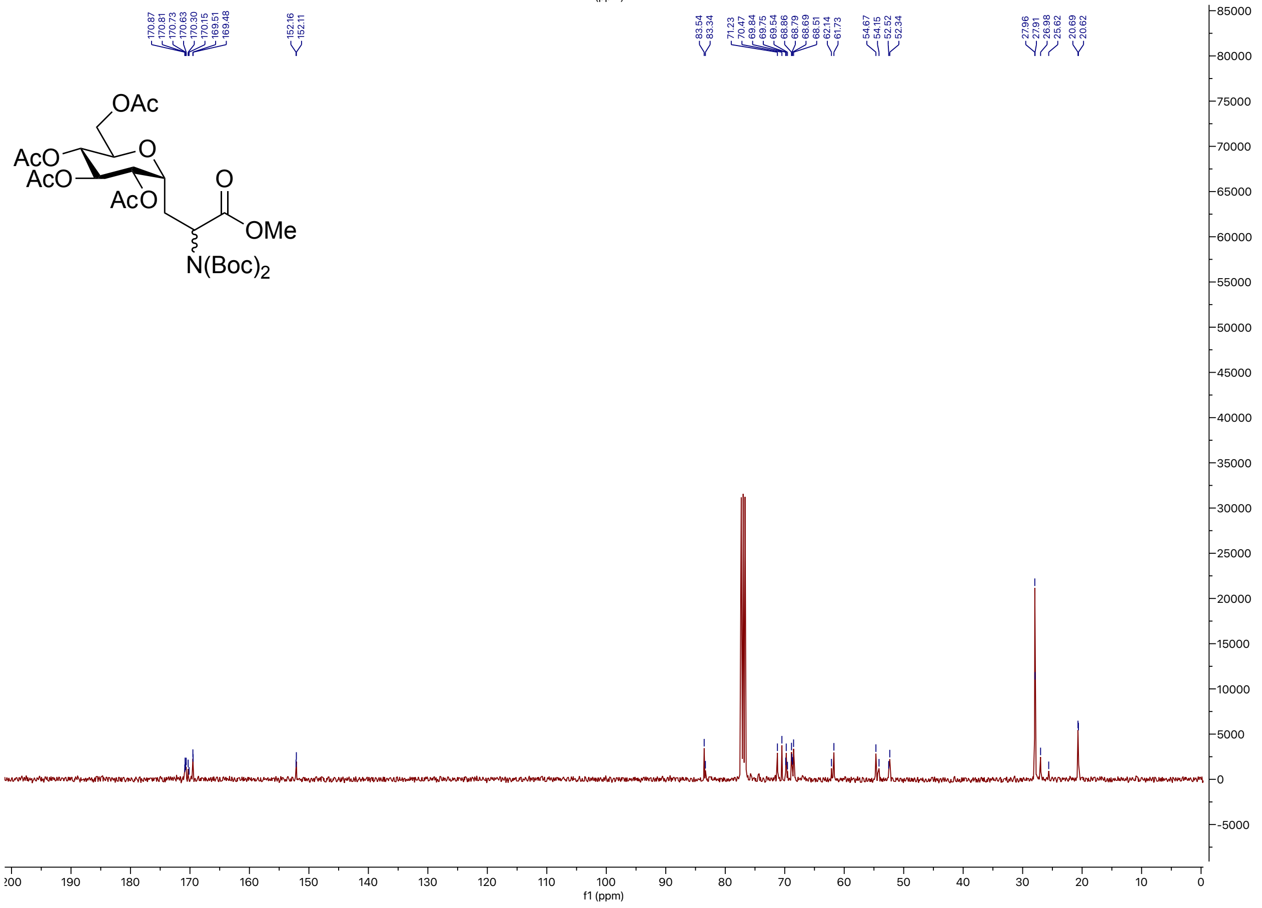

^{1}H-NMR (400 MHz),^{13}C{^{1}H}-NMR (101 MHz) of *Methyl[3-α(2,3,4,6-Tetra-O-acetyl-α-mannopyranosyl)]-(2S/2R)-N,N-di-tert-buthoxycarbonyl-alanine (3ga) - (CDCl$_3$).*

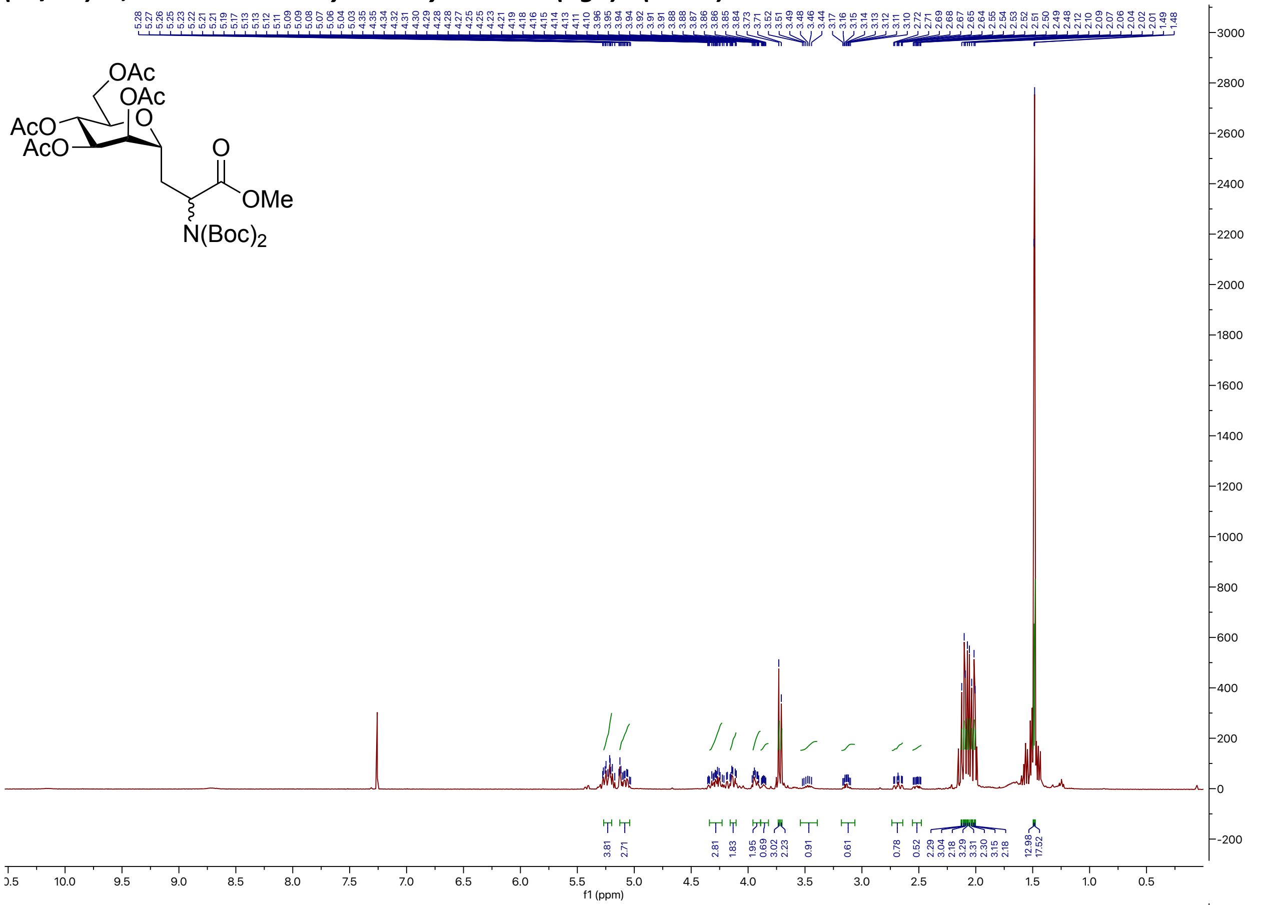

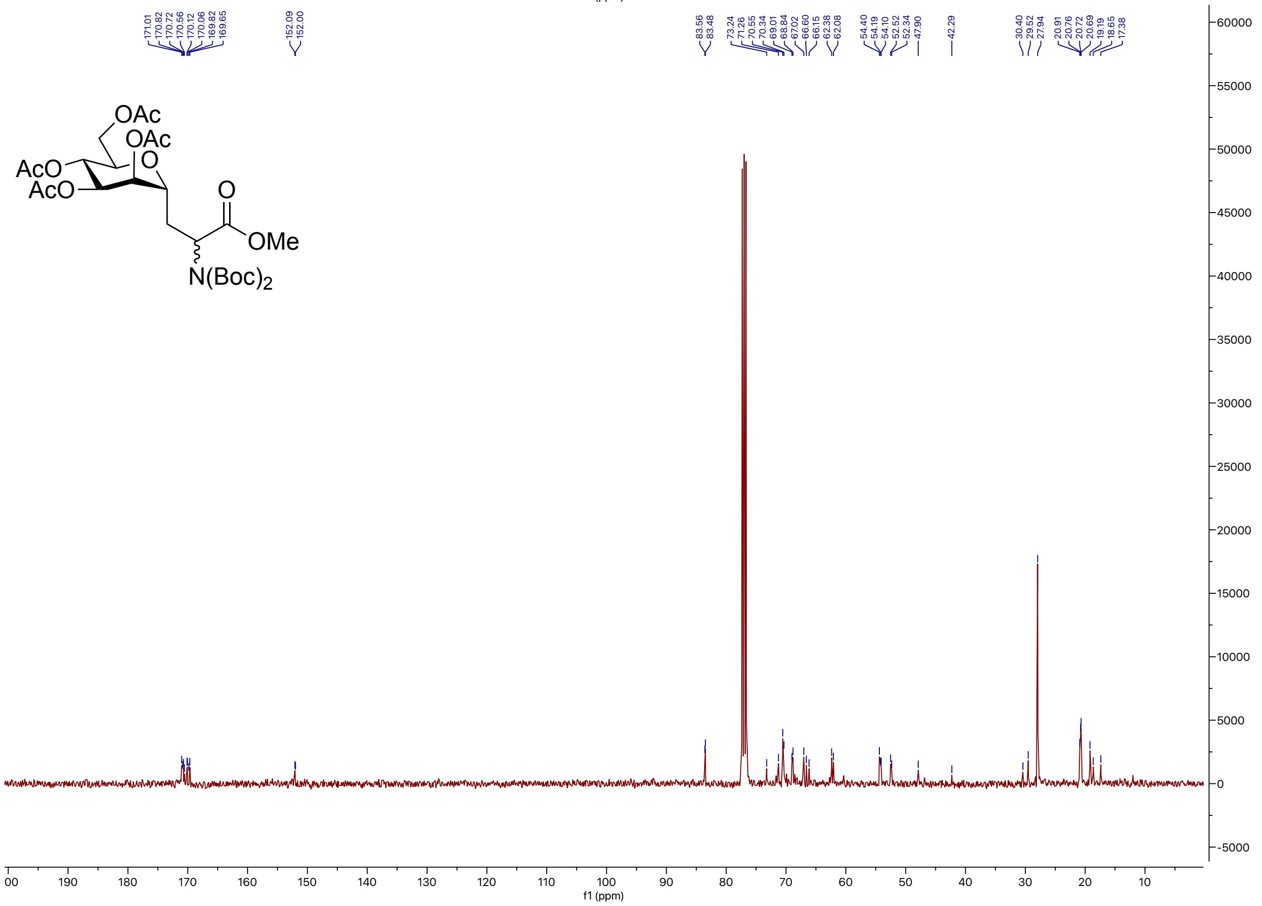

^{1}H-NMR (400 MHz) ,^{13}C{^{1}H}-NMR (101 MHz) of *Methyl[3-α(2,3,4,6-Tetra-O-acetyl-α-galactopyranosyl)]-(2S/2R)-N,N-di-tert-buthoxycarbonyl-alanine (3ha) - (CDCl$_3$).*

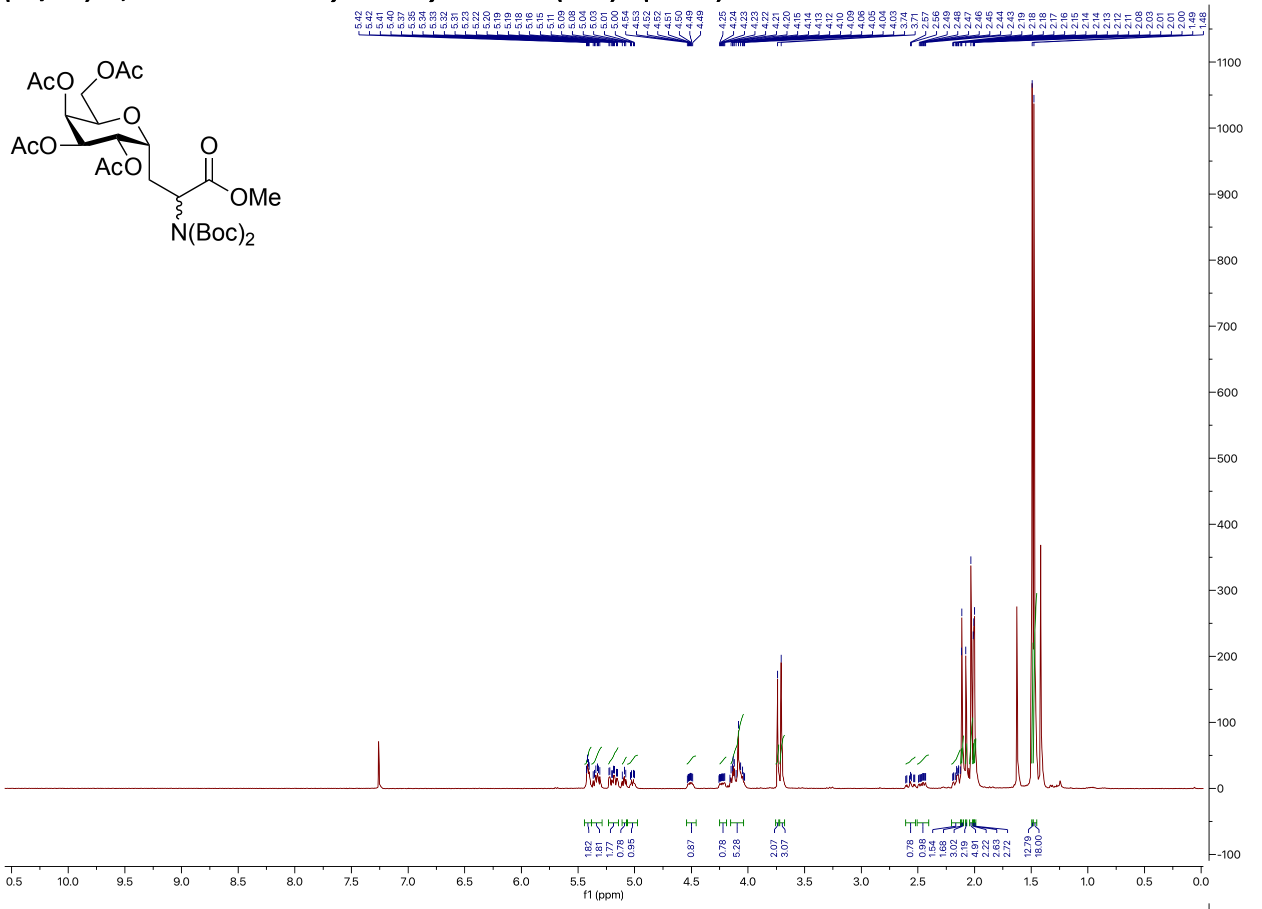

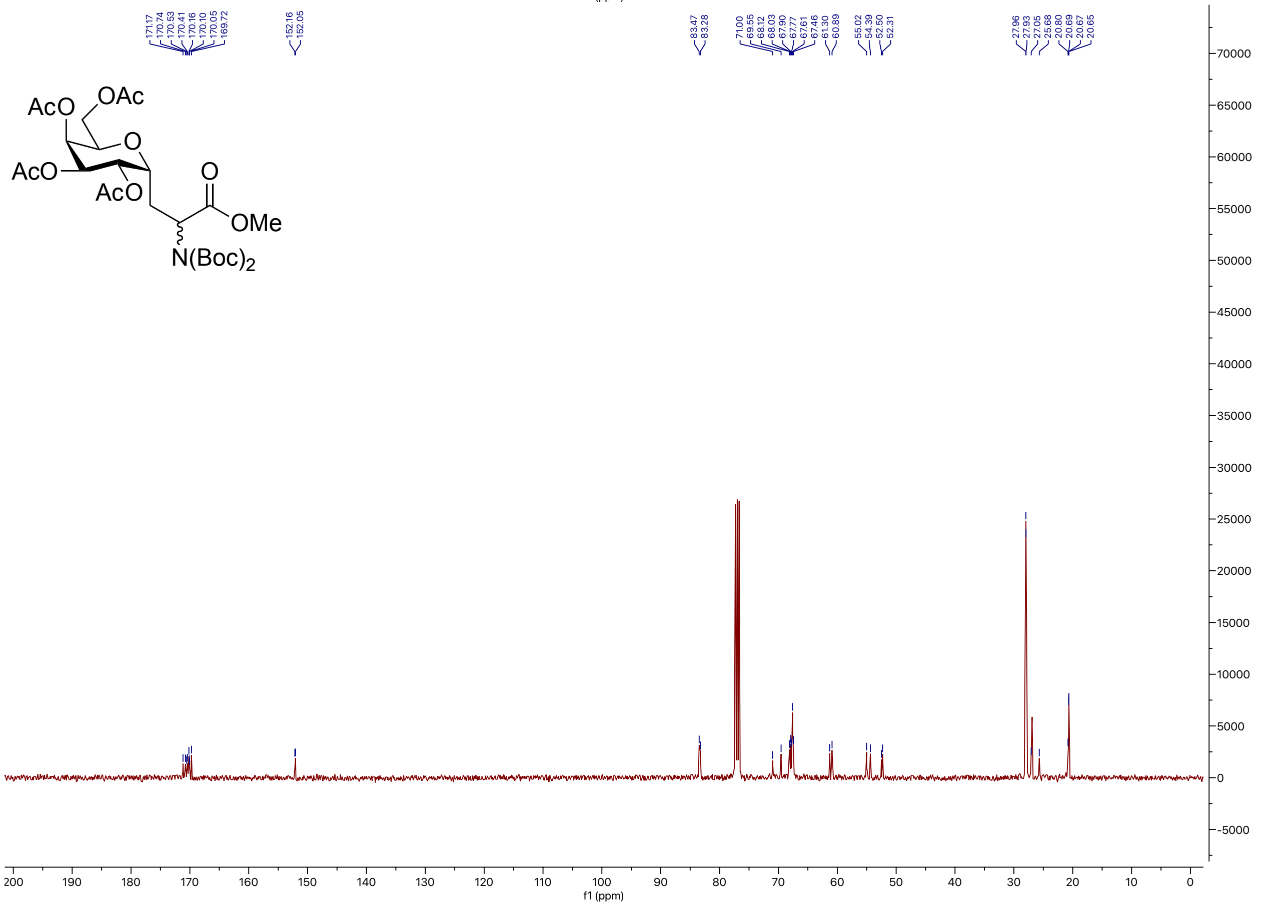

^{1}H-NMR (300 MHz),^{13}C{^{1}H}-NMR (101 MHz) of *Ethyl[3-α(2,3,4,6-Tetra-O-benzoyl-α-glucopyranosyl)]-(2S/2R)-N-acetyl-alanine (3af) – (CDCl₃).*

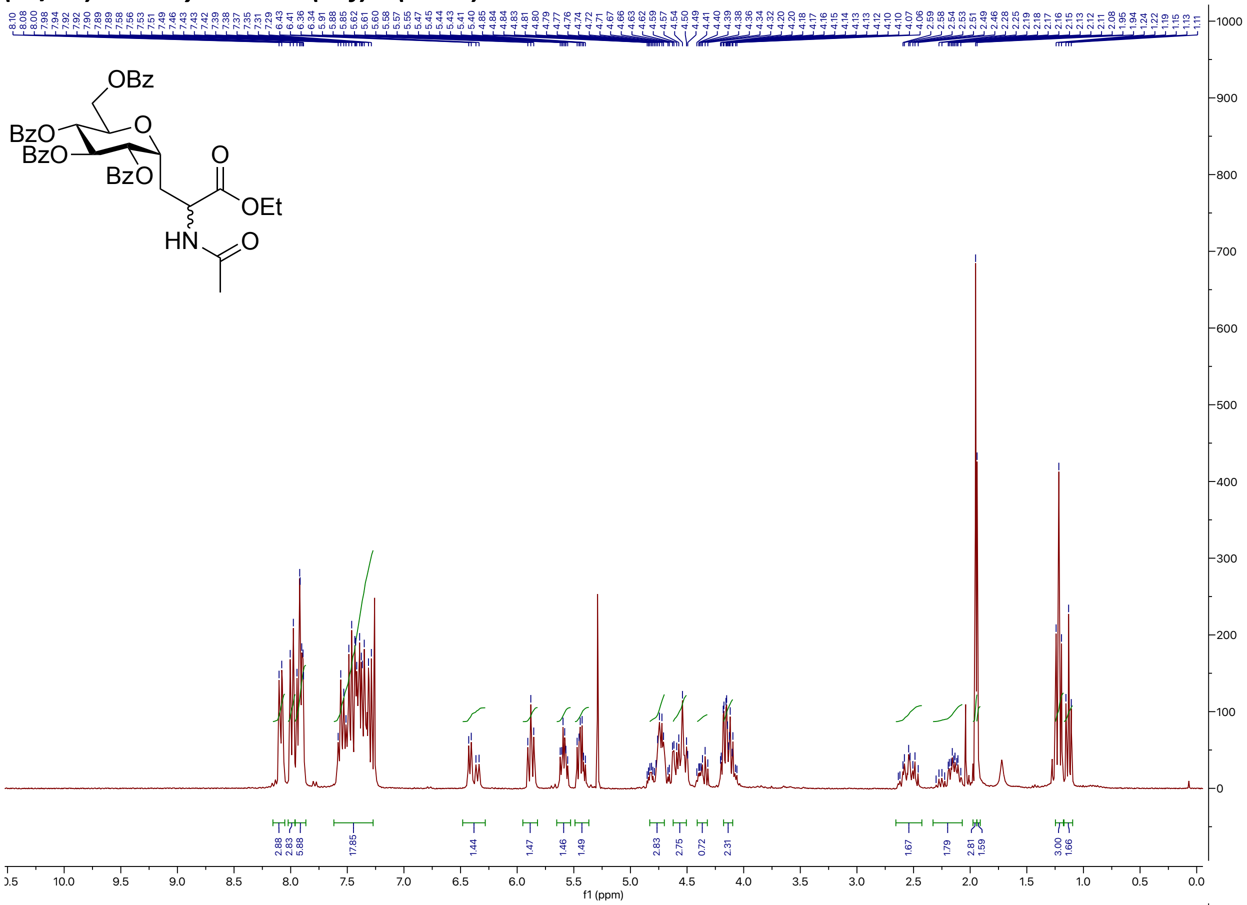

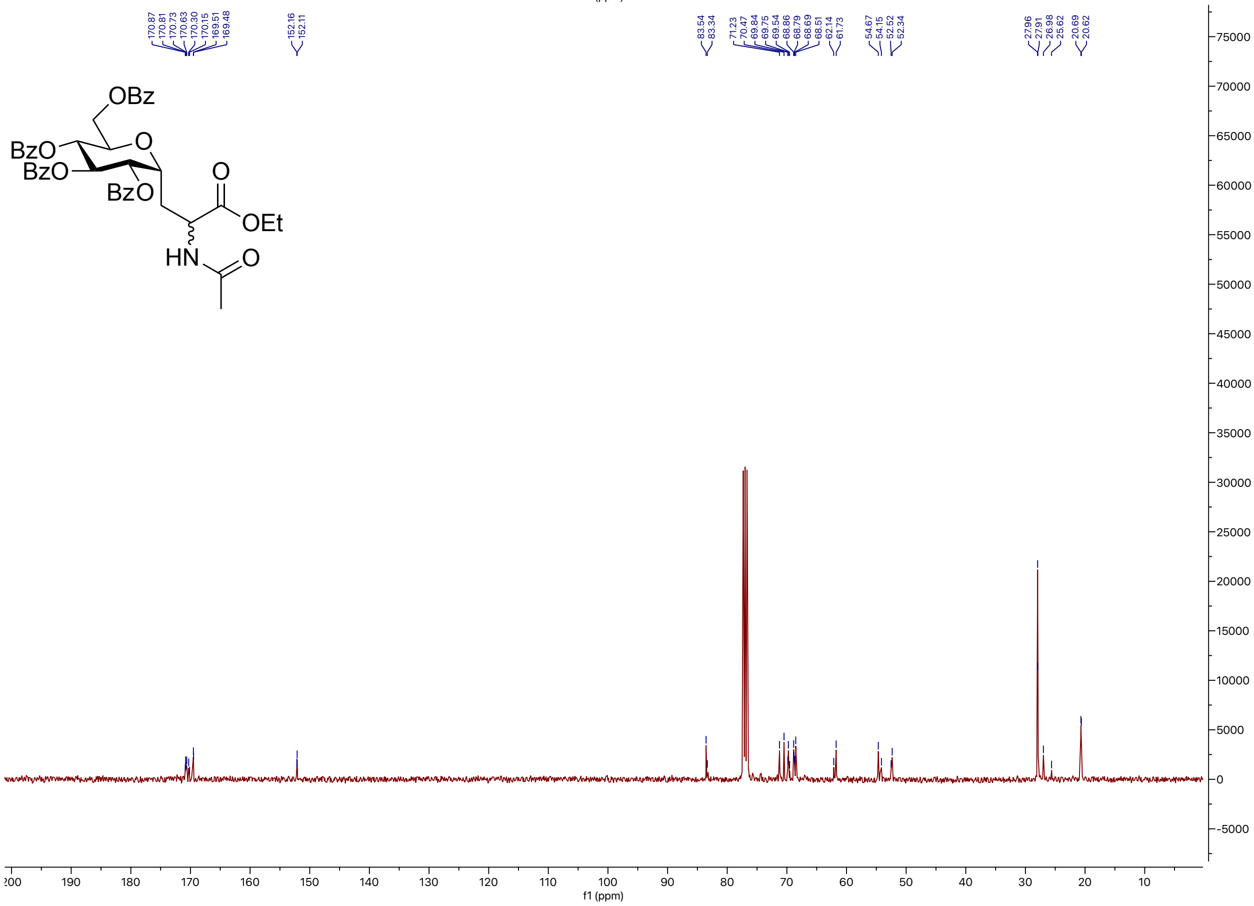

^{1}H-NMR (300 MHz),^{13}C{^{1}H}-NMR (101 MHz)of *Methyl[3-α(2,3,4,6-Tetra-O-benzoyl-β-D-glucopyranosyl-(1→4)-2,3,6-tri-O-benzoyl-α-D-glucopyranosyl]-(2S/2R)-N,N-di-tert-buthoxycarbonyl-alanine (3ia) – (CDCl$_3$).*

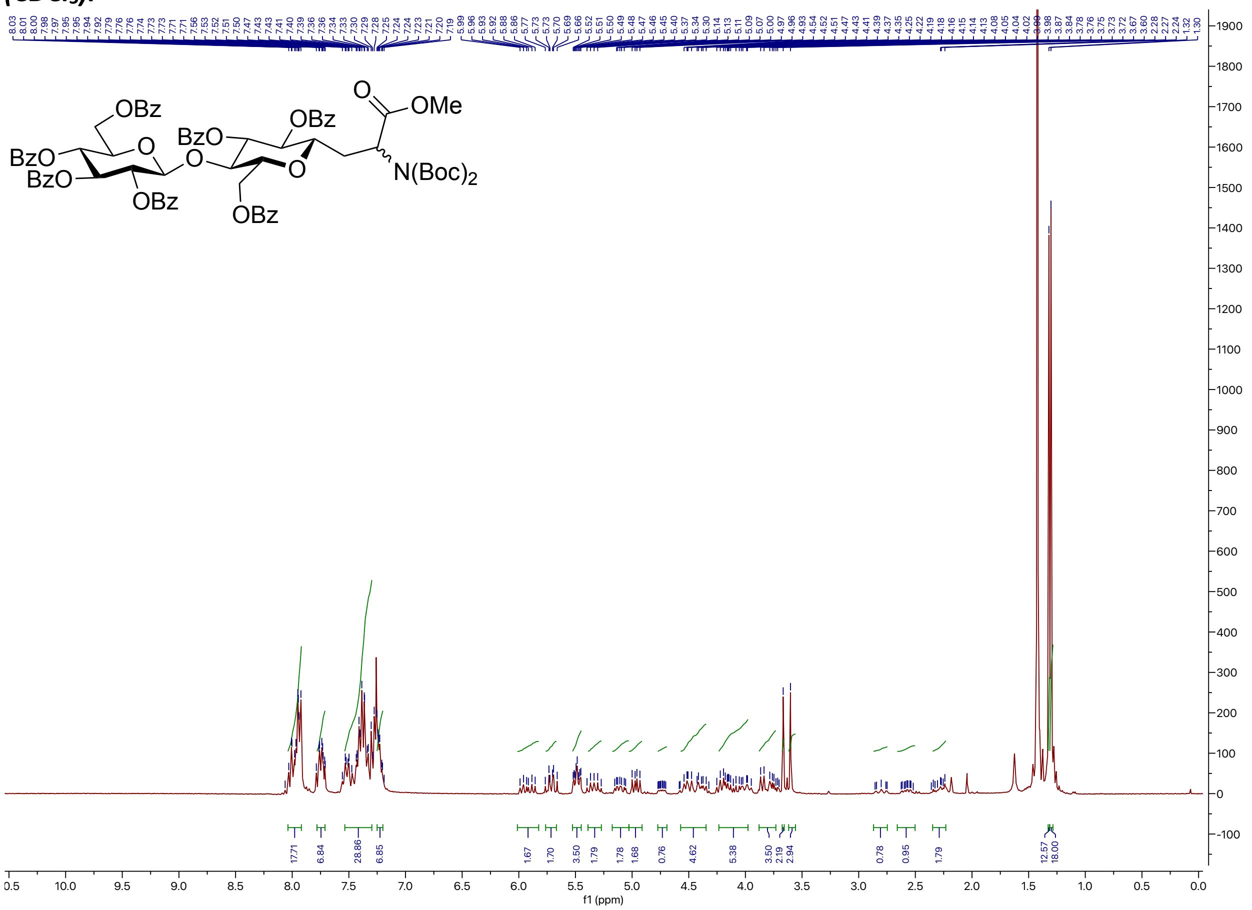

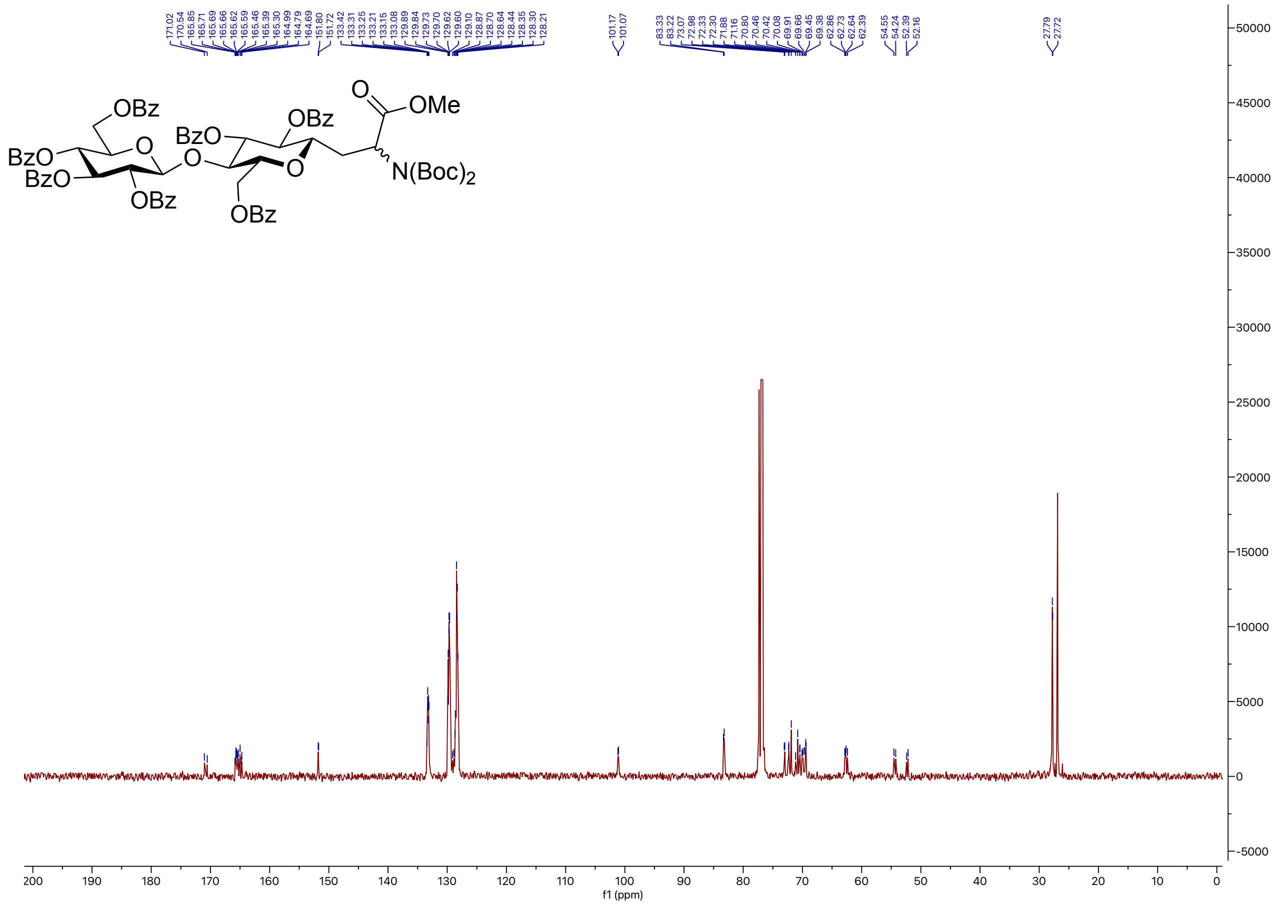

Mechanistic studies by optical spectroscopy

The reaction between the excited state of the photocatalyst (PC*) with DIPEA (eqs. S1,S2) was studied by luminescence techniques and treated using Stern-Volmer analysis (Figure S29, S32, and S36 for photocatalysts **A**, **B**, and **D**, respectively). Formation of PC^{-} according to the photochemical reaction (eq. S2) and the following thermal reaction involving the photogenerated $DPEA^{\bullet+}$ radical (eqs S3-S5) was confirmed by transient absorption spectroscopy (Figure 1a and S33 for photocatalysts **A** and **B**, respectively). Formation of PC^{-} was instead not appreciable under flash photolysis conditions for photocatalyst **D** due to the low excited state quenching yield (Figure S36).

PC + hν → PC* (S1)
PC* + DIPEA → PC^{-} + $DIPEA^{\bullet+}$ (S2)
$DIPEA^{\bullet+}$ + DIPEA → $DIPEA^{\bullet}$ + $DIPEAH^{+}$ (S3)
$DIPEA^{\bullet}$ + PC → PC^{-} + $DIPEA^{+}$ (S4)
$DIPEA^{+}$ → degradation products (S5)

The subsequent reaction between the photogenerated PC^{-} and sugar **1a** (eq. S6) was studied by transient absorption spectroscopy under pseudo-first order kinetic conditions (eqs. S7-S9). The bimolecular rate constant of eq. S6 was estimated from a single-exponential fitting of the kinetic traces according to eqs. S7,S8 (Figure 1b and S34a for photocatalysts **A** and **B**, respectively) and subsequent linear correlation according to eq. S9 (Figure S30 and S34b for photocatalysts **A** and **B**, respectively).

PC^{-} + **1a** → PC + **1a**$^{-}$ (S6)

$$-\frac{d[\boldsymbol{PC^{-}}]}{dt} = k[\boldsymbol{PC^{-}}][\boldsymbol{1a}] \approx k'[\boldsymbol{PC^{-}}] \quad (S7)$$

$$[\boldsymbol{PC^{-}}] = [\boldsymbol{PC^{-}}]_0 \exp(-k't) \quad (S8)$$

$$k' = k[\boldsymbol{1a}] \quad (S9)$$

The reaction between the photogenerated DIPEA radical and sugar **1a** was finally monitored from the disappearance of the delayed component in the formation of PC^{-} (Figure S31 and S35 for photocatalysts **A** and **B**, respectively) due to the favourable competition of eq. S10 with eq. S4.

$DIPEA^{\bullet}$ + **1a** → **1a**$^{-}$ + $DIPEA^{+}$ (S10)

We also checked for additional reactivity pathways of PC* by luminescence measurements in the presence of HE, **1a**, and **2a**. In the case of photocatalyst **A**, no emission quenching is detected with any reagents. In the case of photocatalysts **B** and **D**, excited state quenching is observed in the presence of HE due to a reductive electron transfer process (eqs. S1,S11) and the resulting Stern-Volmer analysis is reported in Figure S33 and S38 for **B** and **D**, respectively.

PC* + HE → PC^{-} + $HE^{\bullet+}$ (S11)

These data suggest the parallel participation of the reaction in eq. S11 in the photogeneration of PC^{-} when photocatalysts **B** and **D** are used.

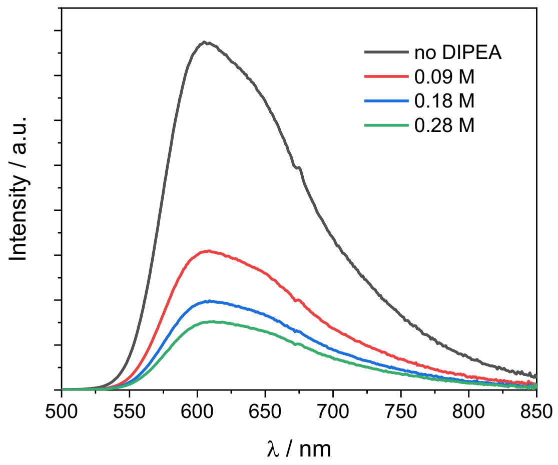

a)

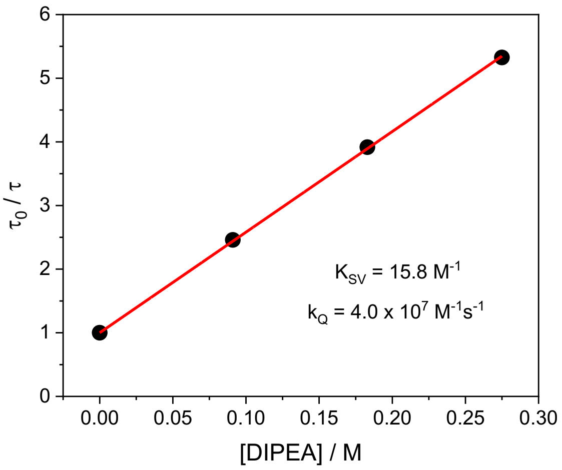

b)

*Figure S29: a) Luminescence spectra (excitation at 450 nm) of CH_2Cl_2 solution containing photocatalyst **A** and 0-0.36 M DIPEA and b) Stern-Volmer analysis obtained by time-resolved emission.*

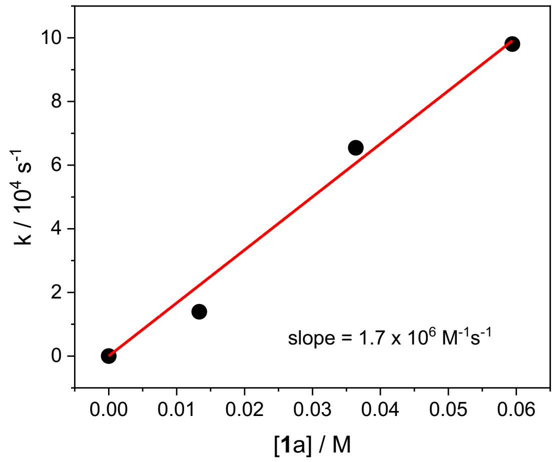

*Figure S30: Plot of the pseudo-first order rates, from the single-exponential fitting of the kinetics in Figure 1b of the main article, vs. [**1a**] to estimate the bimolecular rate constant of the electron transfer from photogenerated **A**⁻ to **1a**.*

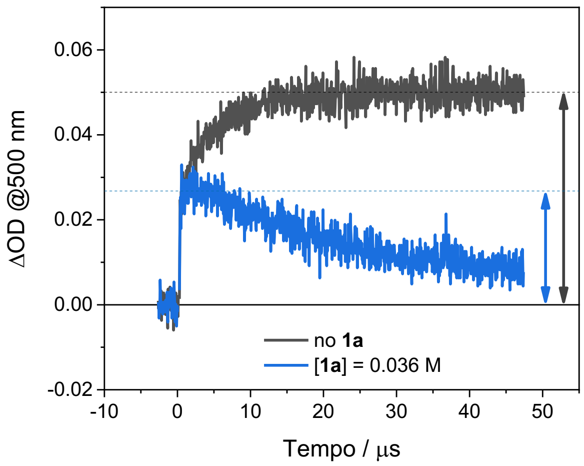

*Figure S31: Detail at short time-scales of the kinetic traces at 500 nm obtained by flash photolysis (excitation at 355 nm, FWHM = 10 ns) of a DCM solution containing **A**, 0.37 M DIPEA and 0-0.036 M **1a**. The arrows on the right-hand side explicitly measure the maximum ΔOD signal at 500 nm associated with the maximum concentration of photogenerated **A**⁻ in the presence and absence of **1a**.*

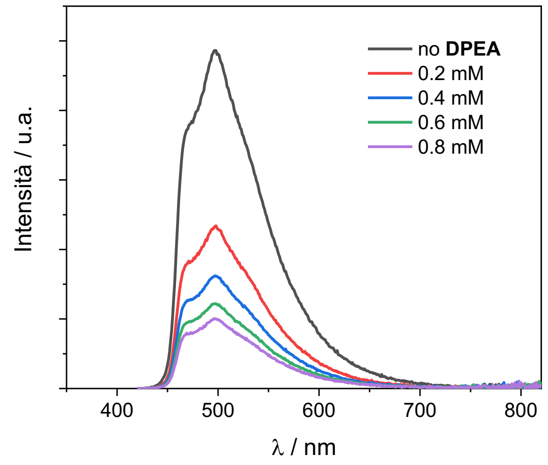

a)

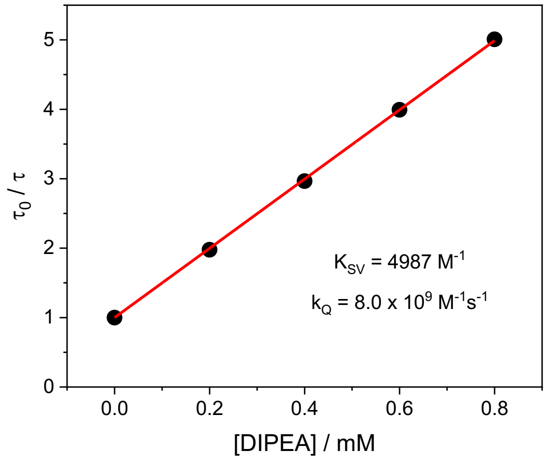

b)

*Figure S32: a) Luminescence spectra (excitation at 400 nm) of CH_2Cl_2 solution containing photocatalyst **B** and 0-0.8 mM DIPEA and b) Stern-Volmer analysis obtained by time-resolved emission.*

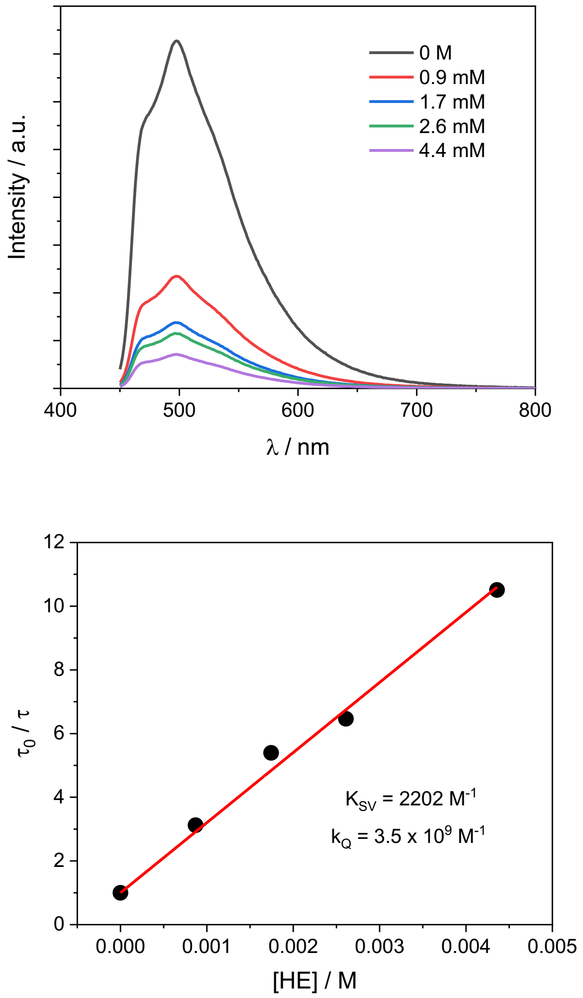

b)

*Figure S33: a) Luminescence spectra (excitation at 400 nm) of CH_2Cl_2 solution containing photocatalyst **B** and 0-4.4 mM HE and b) Stern-Volmer analysis obtained by time-resolved luminescence data.*

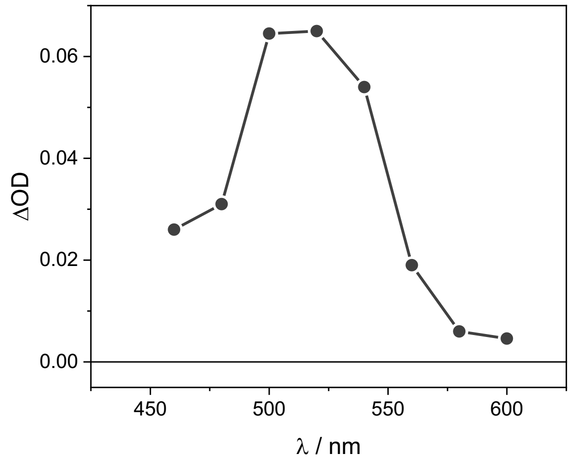

a)

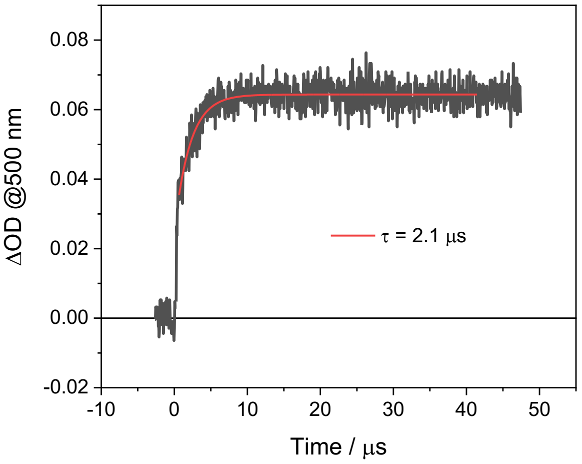

b)

*Figure S34: a) Transient absorption spectrum of the photogenerated **B**⁻ species and b) kinetic trace at 500 nm obtained by flash photolysis (excitation at 355 nm, FWHM = 10 ns) of a DCM solution containing **B** and 0.37 M DIPEA.*

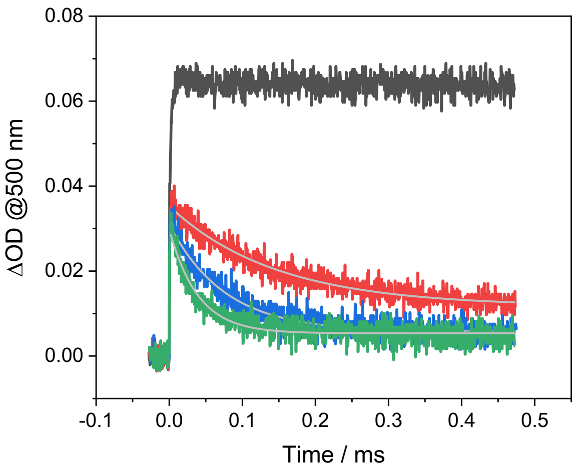

a)

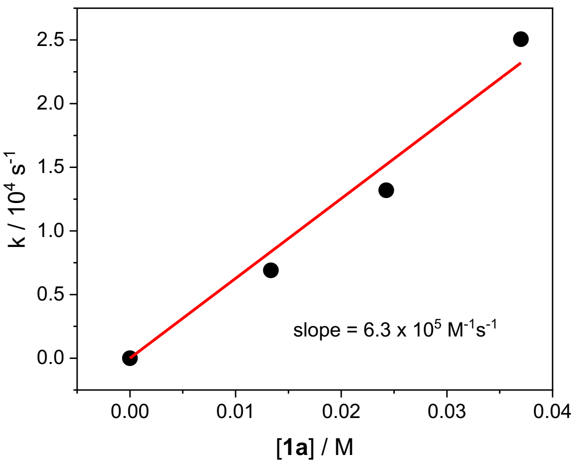

b)

*Figure S35: a) Kinetic traces at 500 nm obtained by flash photolysis (excitation at 355 nm, FWHM = 10 ns) of a DCM solution containing **B**, 0.37 M DIPEA and 0 (black trace), 0.013 M (red trace), 0.024 M (blue trace), and 0.037 M (green trace) sugar **1a** and b) plot of the pseudo-first order rates, from the single-exponential fitting of the kinetics traces, vs. [**1a**] to estimate the bimolecular rate constant of the electron transfer from photogenerated **B**⁻ to **1a**.*

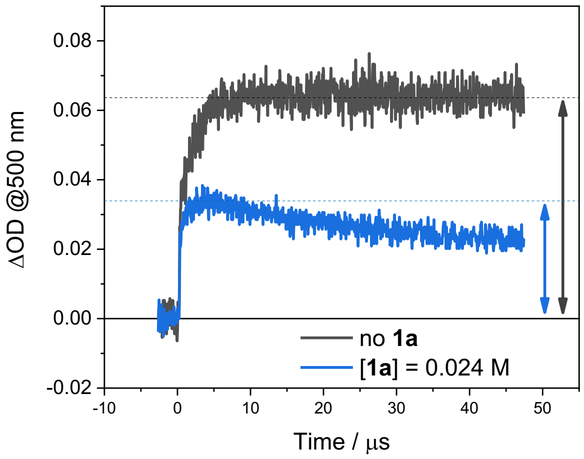

*Figure S36: Detail at short time-scales of the kinetic traces at 500 nm obtained by flash photolysis (excitation at 355 nm, FWHM = 10 ns) of a DCM solution containing **B** 0.37 M DIPEA and 0-0.024 M **1a**. The arrows on the right-hand side explicitly measure the maximum ΔOD signal at 500 nm associated with the maximum concentration of photogenerated $\mathbf{B}^{-}$ in the presence and absence of **1a**.*

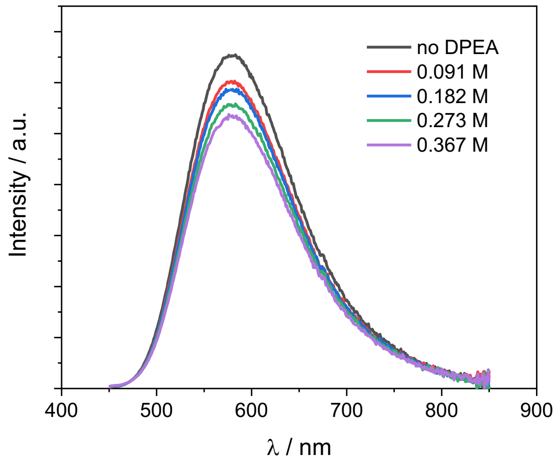

a)

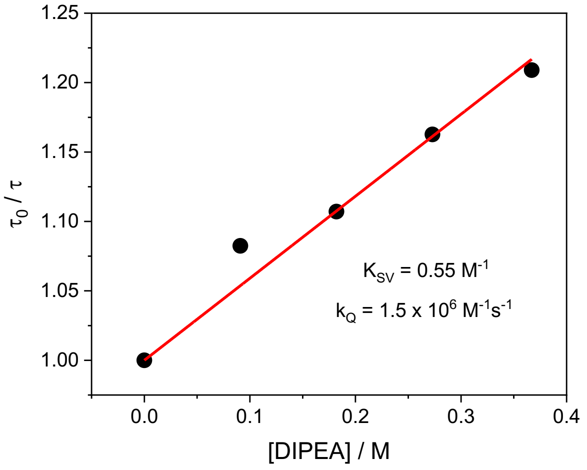

b)

Figure S37: a) Luminescence spectra (excitation at 390 nm) of CH_2Cl_2 solution containing photocatalyst ***D*** *and 0-0.36 M DIPEA and b) Stern-Volmer analysis obtained by time-resolved emission.*

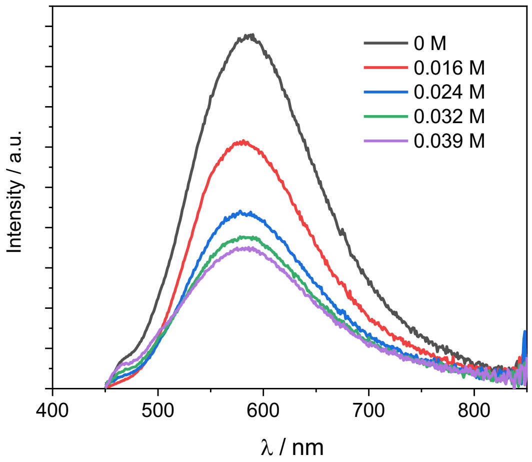

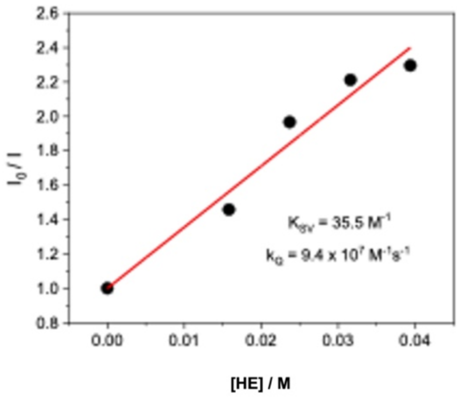

b)

*Figure S38: a) Luminescence spectra (excitation at 410 nm) of CH_2Cl_2 solution containing photocatalyst **D** and 0-0.04 M HE corrected considering the inner filter effect by HE and b) Stern-Volmer analysis obtained from corrected steady-state luminescence data (time-resolved emission is hampered by competitive absorption of HE at the 355 nm excitation wavelength; the value of the bimolecular rate constant should be taken with uncertainty due to the strong inner filter effect by HE and the correction applied).*

References:

(1) J. A. Broomhead, C. G. Young, *Inorganic Syntheses*, **1990**, *28*, 338.
(2) C. Deldaelea, B. Micheleta, H. Baguiaa, S. Kajoujb, E. Romeroa, C. Moucheronb, G. Evano, *Chimia*, **2018**, *72*, 621.
(3) L. M. Doyle, S. O'Sullivan, C. Di Salvo, M. McKinney, P. McArdle, P. V. Murphy, *Org. Lett.*, **2017**, *19*, 5802.
(4) P. Thumbs, T. T. Ensfelder, M. Hillmeier, M. Wagner, M. Heiss, C. Scheel, A. Schçn, M. Müller, S. Michalakis, S. Kellner, T. Carell, *Angew. Chem. Int. Ed.*, **2020**, *59*, 12352.
(5) M. Li, Y. Qiu, C. Wang, X. Li, W. Wei, Y. Wang, Q. Bao, Y. Ding, W. Shi, and Y. Liang, *Org. Lett.*, **2020**, *22*, 6288.
(6) R. A. Aycock, D. B. Vogt and N. T. Jui, **2017**, *8*, 7998.
(7) L. Zhao, X. Li, L. Cao, R. Zhang, X. Shia, J. Qi, *Chem. Commun.*, **2017**, *53*, 5985.
(8) F. Crestey, V. Collot, S. Stiebing, S. Rault, *Synthesis*, **2006**, *20*, 3506.
(9) J. A. Leitch, A. L. Fuentes de Arriba, J. Tan, O. Hoff, C. M. Martinez, D. J. Dixon, *Chem. Sci.*, **2018**, *9*, 6653.
