# Supplementary material for: Modulable Photocatalyzed Strategies for the Synthesis of α-C-Glycosyl Alanine Analogues via the Giese Reaction with Dehydroalanine Derivates
Source: Org Lett. 2023 Jun 22;25(26):4862–7. doi: 10.1021/acs.orglett.3c01660 (PMC10334469; doi:10.1021/acs.orglett.3c01660)
Supplement: Supplementary file 1 — ol3c01660_si_001.zip [file ol3c01660_si_001.zip › Spectra_Photoglyco_def/Spectra Sugars/8/13C - 8/plots/CARBON_01_plot01.pdf]

NC_L_17

Sample Name	NC_L_17	Pulse sequence	CARBON	Temperature	25	Study owner	vnmr1
Date collected	2023-04-12	Solvent	cdcl3	Spectrometer	nmr400.unife.it-mercury400	Operator	vnmr1

SAMPLE

date	Apr 12 2023
solvent	cdcl3
file	/vnmr/fidlib/utenti/lorenzo_poletti400/NC_L_17_20230412_02/CARBON_01.fid

ACQUISITION

sw	25125.6
at	0.700
np	35176
fb	13800
bs	32
d1	0
nt	1024
ct	1024

TRANSMITTER

tn	C13
sfrq	100.583
tof	1544.6
tpwr	60
pw	3.600

DECOUPLER

dn	H1
dof	0
dm	yyy
decwave	w
dpwr	42
dmf	8500

PRESATURATION

satmode	n
wet	n

SPECIAL

temp	25.0
gain	30
spin	20
hst	0.008
pw90	10.800
alfa	10.000

FLAGS

il	n
in	n
dp	y
hs	nn

PROCESSING

lb	3.00
fn	65536

DISPLAY

sp	-1499.9
wp	25125.6
rfl	1499.9
rfp	0
rp	161.0
lp	-430.8

PLOT

wc	375
sc	8
vs	615
th	10

ai cdc ph

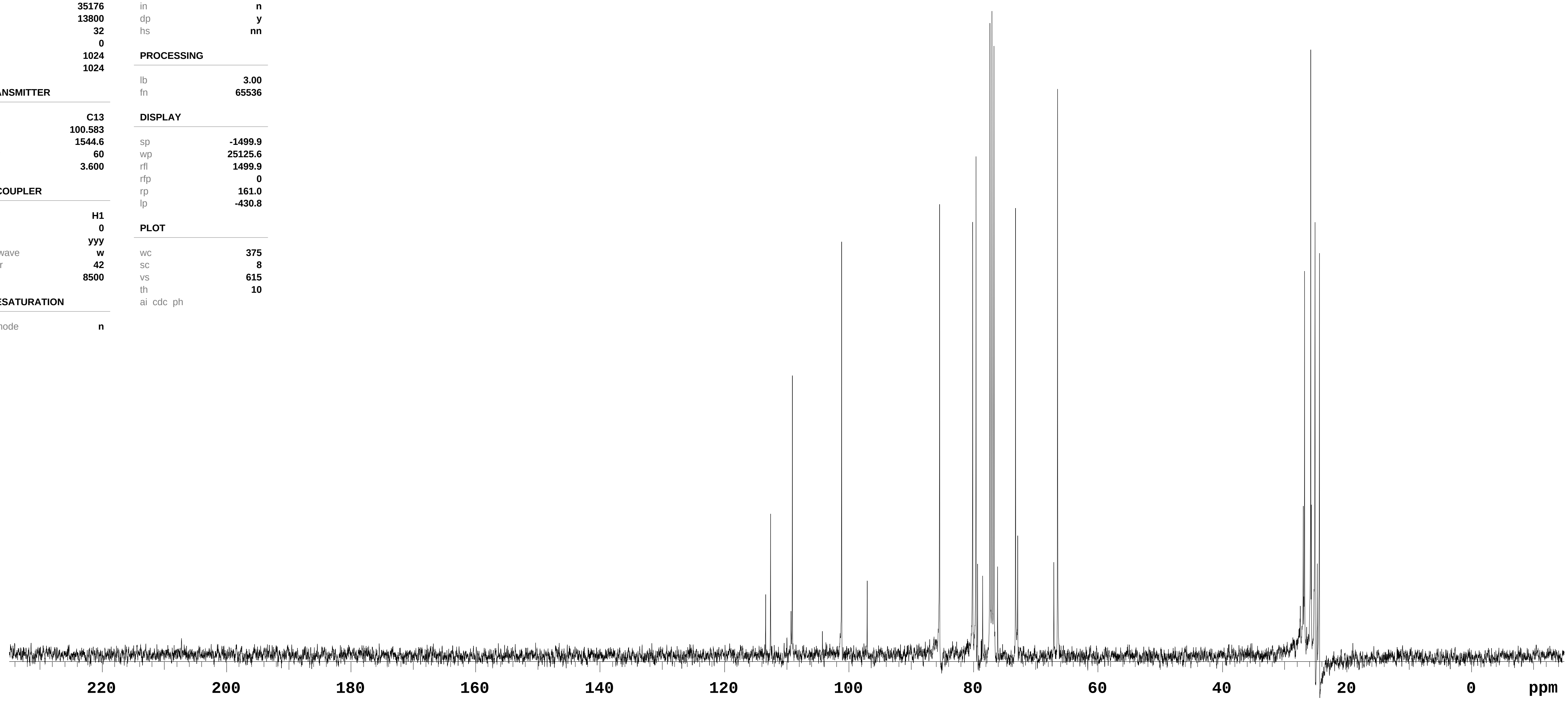

Plotname: CARBON_01_plot01

Data file /vnmr/fidlib/utenti/lorenzo_poletti400/NC_L_17_20230412_02/CARBON_01.fid

Plot date 2023-04-12
