# Supplementary material for: Modulable Photocatalyzed Strategies for the Synthesis of α-C-Glycosyl Alanine Analogues via the Giese Reaction with Dehydroalanine Derivates
Source: Org Lett. 2023 Jun 22;25(26):4862–7. doi: 10.1021/acs.orglett.3c01660 (PMC10334469; doi:10.1021/acs.orglett.3c01660)
Supplement: Supplementary file 1 — ol3c01660_si_001.zip [file ol3c01660_si_001.zip › Spectra_Photoglyco_def/Spectra Dehydroalanine/2f/1H - 2f/plots/PROTON_01_plot01.pdf]

LP_R_241

Sample Name	LP_R_241	Pulse sequence	PROTON	Temperature	25	Study owner	vnmr1
Date collected	2023-04-13	Solvent	cdcl3	Spectrometer	nmr400.unife.it-mercury400	Operator	vnmr1

SAMPLE

date	Apr 13 2023
solvent	cdcl3
file	/vnmr/fidlib/utenti/lorenzo_poletti400/LP_R_241_20230413_01/PROTON_01.fid

ACQUISITION

sw	6398.0
at	3.000
np	38388
fb	3600
bs	8
d1	0
nt	16
ct	16

TRANSMITTER

tn	H1
sfrq	399.971
tof	452.5
tpwr	62
pw	6.000

DECOUPLER

dn	C13
dof	0
dm	nnn
decwave	g
dpwr	45
dmf	17100

PRESATURATION

satmode	n
wet	n

SPECIAL

temp	25.0
gain	30
spin	20
hst	0.008
pw90	12.000
alfa	10.000

FLAGS

il	n
in	n
dp	y
hs	nn

PROCESSING

lb	0.10
fn	32768

DISPLAY

sp	-798.8
wp	6397.6
rfl	799.2
rfp	0
rp	-15.8
lp	-114.2

PLOT

wc	375
sc	8
vs	24
th	62

ai cdc ph

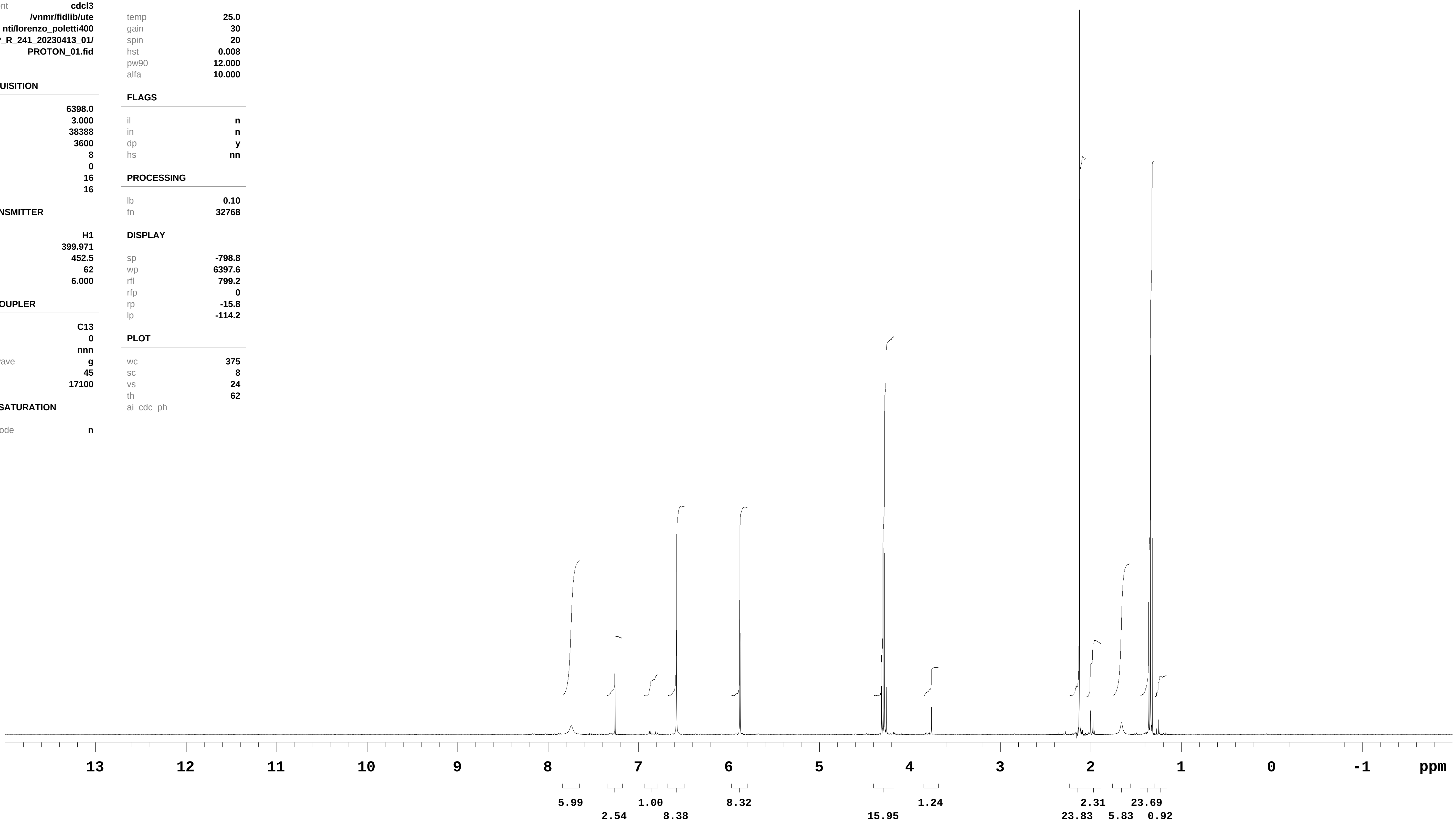

Plotname: PROTON_01_plot01

Data file /vnmr/fidlib/utenti/lorenzo_poletti400/LP_R_241_20230413_01/PROTON_01.fid

Plot date 2023-04-13
